# Supplementary material for: Genome and epigenome wide studies of neurological protein biomarkers in the Lothian Birth Cohort 1936
Source: Nat Commun. 2019 Jul 18;10:3160. doi: 10.1038/s41467-019-11177-x (PMC6639385; doi:10.1038/s41467-019-11177-x)
Supplement: Supplementary file 1 — Supplementary Information [file 41467_2019_11177_MOESM1_ESM.pdf]

Genome and epigenome wide studies of neurological protein biomarkers in the Lothian Birth Cohort 1936

Hillary *et al.*

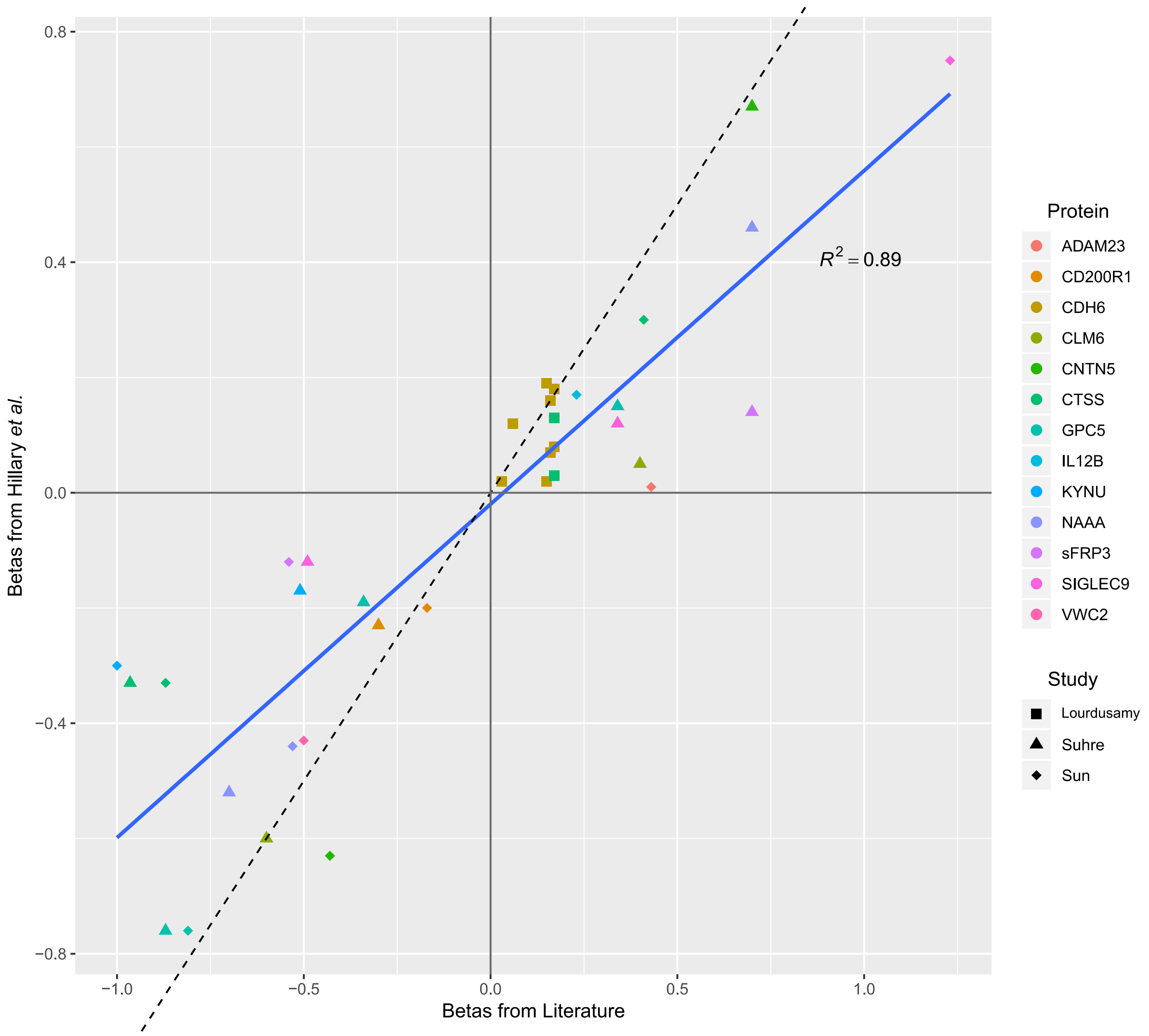

Supplementary Figure 1. The correlation of effect sizes from the present study, Hillary *et al.* and those from pre-existing literature. Blue line indicates correlation between effect sizes (r^2 = 0.89), black dotted line indicates hypothetical perfect correlation (r^2 = 1.0) for visual comparison.

Genome-wide association studies on Olink® neurology proteins

LBC study participants recruited and blood samples drawn at wave 1

(mean age = 70; n = 1,091)

Genotyping performed on LBC1936 participants (n = 1,005). Exclusion criteria: disagreement between genetic and reproted gender (n = 12), relatendess (n = 8), samples with call rate ≤ 0.95 (n = 16) and those showing evidence of non-European descent by multidimensional scaling (n = 1)

Blood samples assayed by Olink® using neurology panel at wave 2 (n = 814). Following quality control, proteomic and genotyping data combined for GWAS (n = 750)

Epigenome-wide association studies on Olink® neurology proteins

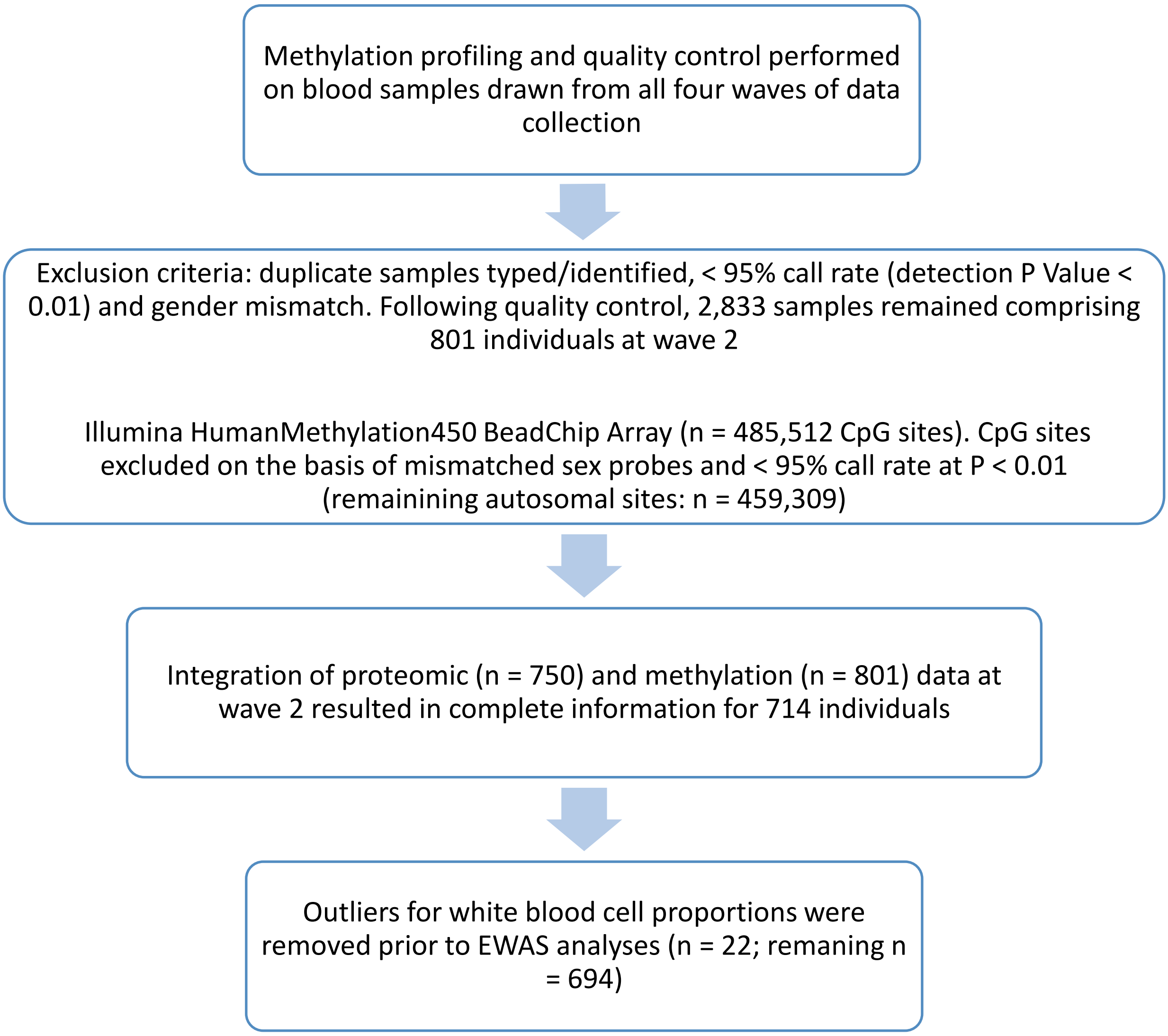

Supplementary Figure 2. Flow chart for inclusion/exclusion of study participants in Lothian Birth Cohort 1936. The Lothian Birth Cohort of 1936 (LBC1936) represents a sample of healthy older adults. For inclusion, participants must not have had a neurodegenerative disease at Wave 1. Genotypic and DNA methylation data were obtained from individuals and integrated with proteomic data measured at Wave 2 of data collection (mean age: 73). A flow chart describing the inclusion and exclusion criteria for the genome- and epigenome-wide association studies (GWAS/EWAS) on Olink® neurological protein levels in LBC1936 participants is presented above.

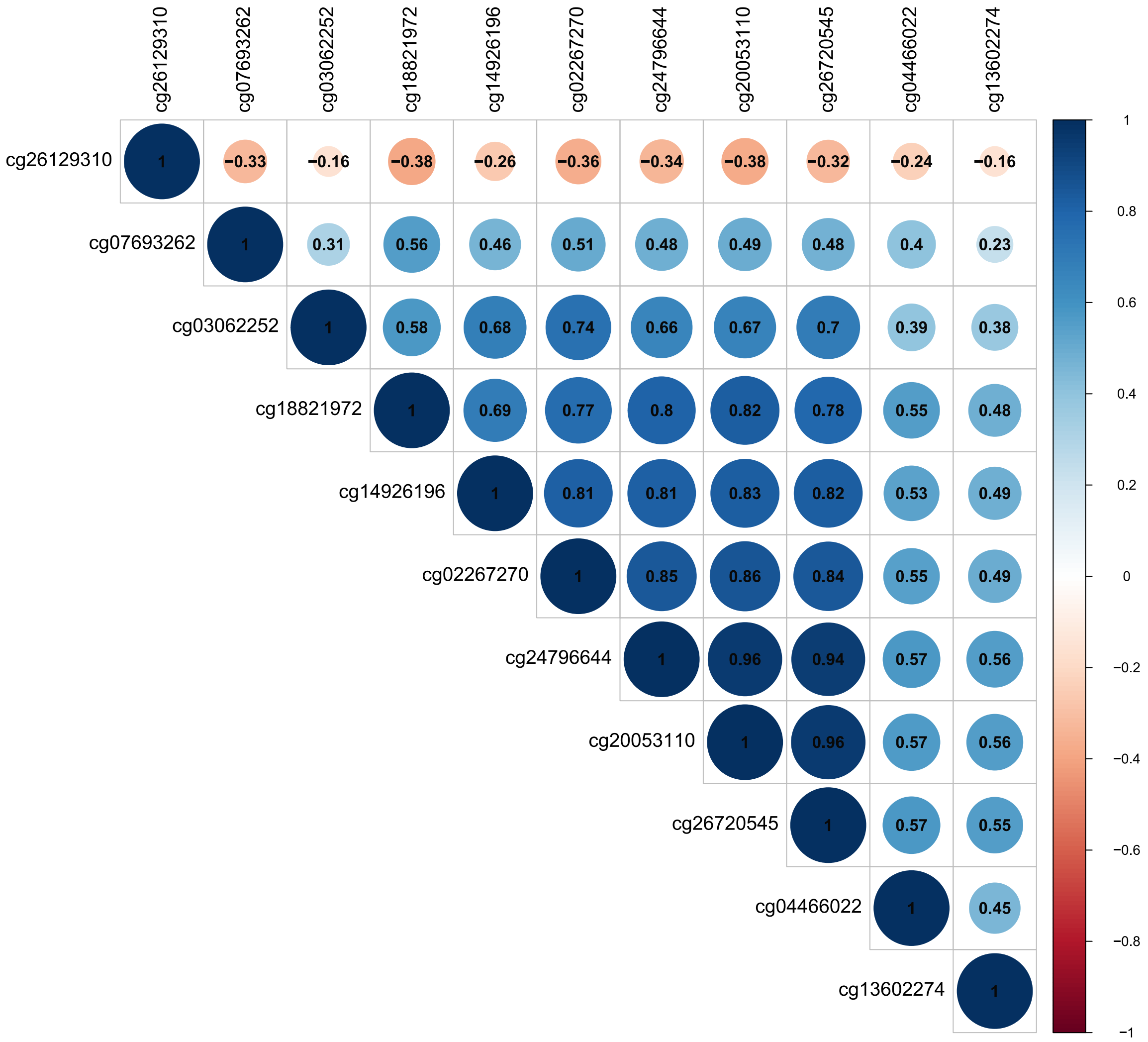

Supplementary Figure 3. The correlation between eleven CpG sites significantly associated with normalised MDGA1 levels in the Lothian Birth Cohort of 1936. Blue values indicate positive correlations, red values indicate negative correlations.

Supplementary Table 1. R2 measures of linkage disequilibrium between non-overlapping pQTLs (discordant between COJO/FUMA) identified by COJO and FUMA (mapped to British population)

Protein	COJO	FUMA	R2
ADAM 23	rs1448903	rs13429599	0.05
ADAM22	rs12535512	rs13233308	**0.78**
CD200R1	rs12233417	rs79834152	**1.00**
CD200R1	rs12233417	rs7622812	0.02
CD200R1	rs12233417	rs12493830	0.01
CD200R1	rs4857414	rs12493830	**0.91**
CD200R1	rs4857414	rs79834152	0.01
CD200R1	rs4857414	rs7622812	0.01
CD200R1	rs7622812	rs79834152	0.01
CD200R1	rs7622812	rs12493830	0.01
CDH6	rs1921086	rs4406147	**0.87**
CLEC10A	rs444207	rs2002664	0.24
CLEC10A	rs444207	rs12941354	0.04
CLM-1	rs10512597	rs9903991	**0.97**
CLM-1	rs10512597	rs62084910	0.12
CLM-1	rs10512597	rs11655369	0.09
CLM-1	rs10512597	rs34074270	0.09
CLM-1	rs10512597	rs112607667	0.04
CLM-1	rs10512597	rs16978165	0.04
CLM-6	rs1171196	rs708601	**1.00**
CLM-6	rs1171196	rs2706505	0.01
CNTN5	rs1461674	rs1461677	**1.00**
CTSC	rs217116	rs217053	0.67
CTSC	rs217116	rs17756204	0.01
CTSS	rs2228099	rs2867296	**0.89**
CTSS	rs4970986	rs2867296	**1.00**
DRAXIN	rs12139487	rs12047253	**1.00**
FcRL2	rs12568320	rs2065883	**1.00**
FLRT2	rs4904262	rs2746995	**1.00**
gal-8	rs495828	rs507666	0.71
GDNF	rs17386472	rs11747340	**0.94**
GPC5	rs2352029	rs1929922	**1.00**
GPC5	rs2352029	rs138994828	0.10
IL12	rs10045431	rs6556416	**0.90**
KYNU	rs16858172	rs78201785	**1.00**
LAIR	rs17606864	rs111825282	0.04
LAIR	rs17606864	rs2277972	0.04
LAIR	rs17606864	rs73070113	0.03
LAIR	rs17606864	rs2042290	0.01
LAIR	rs2042290	rs17606864	**1.00**
LAIR	rs2042290	rs111825282	0.04
LAIR	rs2042290	rs2277972	0.04
LAIR	rs2042290	rs73070113	0.03
LXN	rs2228243	rs72625023	0.43
MATN3	rs3731663	rs1147118	0.29
MDGA1	rs6458011	rs9349050	0.48
MDGA1	rs6458011	rs3846881	0.22
MDGA1	rs6458011	rs36086366	0.10
MDGA1	rs6458011	rs114253244	0.09
MDGA1	rs6458011	rs146513610	0.09
MDGA1	rs6458011	rs73415453	0.04
MDGA1	rs6938061	rs9349050	**0.92**
MDGA1	rs6938061	rs36086366	0.20
MDGA1	rs6938061	rs73415453	0.08
MDGA1	rs6938061	rs3846881	0.07
MDGA1	rs6938061	rs114253244	0.05
MDGA1	rs6938061	rs146513610	0.05
N2DL2	rs1853665	rs9383621	**1.00**
NAAA	rs1857821	rs112197434	**0.75**
N-CDase	rs10508921	rs11597071	0.00
N-CDase	rs10508921	rs146075547	0.00
N-CDase	rs1898198	rs11597071	0.16
N-CDase	rs1898198	rs146075547	0.01
NEP	rs4687657	rs35004449	**1.00**
SCARF2	rs361603	rs9610447	**0.82**
sFRP-3	rs1561369	rs288326	**1.00**
sFRP-3	rs1561369	rs143674995	0.00
sFRP-3	rs1561369	rs72890325	0.00
Siglec_9	rs4857414	rs12496730	**0.91**
Siglec_9	rs4857414	rs2673908	0.01
Siglec-9	rs2075803	rs2673908	**1.00**
Siglec-9	rs2075803	rs12496730	0.00
TMPRSS5	rs2465651	rs7110738	**0.96**
VWC2	rs481076	rs482968	**1.00**

Supplementary Table 2. Bayesian tests of colocalisation for *cis* pQTLs and *cis* eQTLs

	Protein	Gene	PP.H0	PP.H1	PP.H2	PP.H3	PP.H4
1	DRAXIN	*DRAXIN*	0.00	0.00	0.00	0.00	**1.00**
2	KYNU	*KYNU*	0.00	0.00	0.00	0.02	**0.97**
3	MDGA1	*MDGA1*	0.00	0.00	0.00	0.05	**0.95**
4	SIGLEC9	*SIGLEC9*	0.00	0.06	0.00	0.04	**0.89**
5	LAIR	*LAIR2*	0.00	0.09	0.00	0.14	**0.77**
6	CLM_6	*CD300C*	0.00	0.00	**0.76**	0.19	0.04
7	NAAA	*NAAA*	0.00	0.00	0.00	**1.00**	0.00
8	CLEC10A	*CLEC10A*	0.00	0.00	0.00	**1.00**	0.00
9	FcRL2	*FCRL2*	0.00	0.00	0.00	**1.00**	0.00
10	CTSS	*CTSS*	0.00	0.00	0.00	**1.00**	0.00
11	CLM_1	*CD300LF*	0.00	0.00	0.00	**1.00**	0.00
12	CTSC	*CTSC*	0.00	0.00	0.00	**0.83**	0.17

H0 (no causal variant), H1 (causal variant for protein only), H2 (causal variant for expression only), H3 (two distinct causal variants), H4 (common causal variant)

Supplementary Table 3. Genome-wide significant CpG sites associated with circulating levels of Olink neurological biomarkers as identified using limma

Biomarker	CpG site	CHR of CpG	CpG Position	Annotation of CpG	Feature	CpG Island	log FC	Ave Expression	t statistic	P Value	B	Chromosome of Biomarker	Gene Start	Gene End	Type
CRTAM	cg02305850	11	126152462	TIRAP	TSS1500	N_Shore	0.01	0.11	6.50	1.65E-10	10.99	11	122838500	122872639	Cis
CRTAM	cg15617814	11	131780492	NTM	TSS1500	Island	0.02	0.11	6.61	8.02E-11	11.70	11	122838500	122872639	Cis
CRTAM	cg04983516	11	79151719	ODZ4	TSS200	Island	0.01	0.06	6.56	1.15E-10	11.35	11	122838500	122872639	Trans
G_CSF	cg09349128	22	50327986			N_Shore	-0.01	0.29	-7.10	3.25E-12	14.81	17	40015361	40017813	Trans
MATN3	cg24416238	2	20211868	MATN3	Body	N_Shore	0.01	0.48	7.30	8.45E-13	16.12	2	20012694	19992111	Cis
MDGA1	cg20053110	6	37617864	MDGA1	Body	Island	0.14	0.56	29.19	1.70E-119	261.18	6	37699306	37630679	Cis
MDGA1	cg24796644	6	37617956	MDGA1	Body	Island	0.15	0.54	27.79	7.25E-112	243.61	6	37699306	37630679	Cis
MDGA1	cg26720545	6	37618009	MDGA1	Body	Island	0.10	0.54	27.52	2.16E-110	240.21	6	37699306	37630679	Cis
MDGA1	cg14926196	6	37616482	MDGA1	Body	Island	0.13	0.55	23.74	1.06E-89	192.57	6	37699306	37630679	Cis
MDGA1	cg18821972	6	37660403	MDGA1	Body	N_Shelf	0.06	0.64	22.79	1.75E-84	180.57	6	37699306	37630679	Cis
MDGA1	cg02267270	6	37616410	MDGA1	Body	Island	0.09	0.54	22.66	8.52E-84	178.98	6	37699306	37630679	Cis
MDGA1	cg03062252	6	37616598	MDGA1	Body	Island	0.05	0.19	15.58	1.33E-46	93.45	6	37699306	37630679	Cis
MDGA1	cg07693262	6	37625029	MDGA1	Body	N_Shore	0.02	0.75	15.32	2.60E-45	90.49	6	37699306	37630679	Cis
MDGA1	cg04466022	6	37618123	MDGA1	Body	Island	0.01	0.95	12.22	5.42E-31	57.64	6	37699306	37630679	Cis
MDGA1	cg13602274	6	37667518			S_Shore	0.02	0.36	11.75	5.26E-29	53.09	6	37699306	37630679	Cis
MDGA1	cg26129310	6	37664451	MDGA1	Body	Island	0.00	0.03	-9.25	3.35E-19	30.68	6	37699306	37630679	Cis
N_CDase	cg22645355	10	52002547	ASAH2	Body	OpenSea	0.06	0.84	7.60	1.08E-13	18.16	10	50248610	50182778	Cis
N_CDase	cg16435686	10	47964834			N_Shelf	-0.07	0.53	-7.53	1.78E-13	17.67	10	50248610	50182778	Cis
NEP	cg06690548	4	139162808	SLC7A11	Body	OpenSea	-0.02	0.82	-7.42	3.77E-13	16.93	3	155024124	155183729	Trans
NEP	cg11645453	3	52864694	ITIH4	5'UTR	OpenSea	0.02	0.64	6.67	5.68E-11	12.00	3	155024124	155183729	Trans
NEP	cg18404041	3	52824283	ITIH1	Body	OpenSea	-0.02	0.42	-6.68	5.31E-11	12.08	3	155024124	155183729	Trans
SIGLEC1	cg05696877	1	79088769	IFI44L	5'UTR	OpenSea	-0.03	0.70	-7.20	1.72E-12	15.44	20	3707128	3686970	Trans
SIGLEC1	cg08122652	3	122281939	PARP9	5'UTR	N_Shore	-0.01	0.79	-6.65	6.51E-11	11.87	20	3707128	3686970	Trans
SMPD1	cg18477969	11	6415462	SMPD1	Body	S_Shelf	0.05	0.82	7.92	1.03E-14	20.46	11	6390431	6394998	Cis
TN_R	cg03636183	19	17000585	F2RL3	Body	N_Shore	0.02	0.63	7.12	2.84E-12	14.93	1	175315194	175743770	Trans
TN_R	cg05575921	5	373378	AHRR	Body	N_Shore	0.02	0.80	6.49	1.68E-10	10.92	1	175315194	175743770	Trans

Supplementary Table 4. Genome-wide significant CpG sites associated with circulating levels of Olink neurological biomarkers as identified using OSCA (CpGs which are discordant with limma are emboldened)

Biomarker	CpG site	CHR of CpG	CpG Position	Annotation of CpG	Strand	CpG Island	B	SE	P Value	Chromsome	Gene Start	Gene End	Type
MATN3	cg24416238	2	20211868	MATN3	+	N_Shore	6.36	0.79	6.76E-16	2	20012694	19992111	Cis
MDGA1	cg02267270	6	37616410	MDGA1	-	Island	5.40	0.22	4.37E-137	6	37699306	37630679	Cis
MDGA1	cg03062252	6	37616598	MDGA1	-	Island	6.18	0.36	5.47E-65	6	37699306	37630679	Cis
MDGA1	cg07693262	6	37625029	MDGA1	+	N_Shore	15.08	0.87	2.53E-67	6	37699306	37630679	Cis
MDGA1	cg14926196	6	37616482	MDGA1	-	Island	3.81	0.16	9.28E-128	6	37699306	37630679	Cis
MDGA1	cg18821972	6	37660403	MDGA1	-	N_Shelf	7.95	0.34	1.32E-119	6	37699306	37630679	Cis
MDGA1	cg20053110	6	37617864	MDGA1	+	Island	4.15	0.14	8.76E-194	6	37699306	37630679	Cis
MDGA1	cg24796644	6	37617956	MDGA1	+	Island	3.87	0.13	5.88E-193	6	37699306	37630679	Cis
MDGA1	cg26720545	6	37618009	MDGA1	+	Island	5.57	0.19	3.96E-188	6	37699306	37630679	Cis
MDGA1	**cg24442454**	6	37616803	MDGA1	+	NA	8.09	0.40	4.73E-89	6	37699306	37630679	Cis
MDGA1	**cg00807871**	6	37617124	MDGA1	+	NA	4.31	0.18	2.51E-127	6	37699306	37630679	Cis
N_CDase	cg16435686	10	47964834		-	N_Shelf	-1.11	0.15	1.32E-13	10	50248610	50182778	Cis
N_CDase	cg22645355	10	52002547	ASAH2;ASAH2	-	NA	1.49	0.20	1.52E-13	10	50248610	50182778	Cis
SMPD1	cg18477969	11	6415462	SMPD1;SMPD1;SMPD1	+	S_Shelf	1.83	0.24	8.95E-15	11	6390431	6394998	Cis
NEP	cg06690548	4	139162808	SLC7A11	-	NA	-3.32	0.51	1.08E-10	3	155024124	155183729	Trans
NEP	cg11645453	3	52864694	ITIH4;ITIH4;ITIH4;ITIH4	+	NA	3.19	0.48	3.98E-11	3	155024124	155183729	Trans
SIGLEC1	cg05696877	1	79088769	IFI44L	+	NA	-2.75	0.43	2.06E-10	20	3707128	3686970	Trans
SIGLEC1	cg08122652	3	122281939	PARP9;PARP9;DTX3L;PARP9;PARP9;PARP9;PARP9	-	N_Shore	-6.97	1.04	2.08E-11	20	3707128	3686970	Trans
TN_R	cg03636183	19	17000585	F2RL3	-	N_Shore	5.21	0.67	5.93E-15	1	175315194	175743770	Trans
TN_R	cg05575921	5	373378	AHRR	+	N_Shore	3.02	0.43	3.73E-12	1	175315194	175743770	Trans
G_CSF	cg09349128	22	50327986		-	N_Shore	-12.77	1.45	1.32E-18	17	40015361	40017813	Trans
G_CSF	**cg12054453**	17	57915717	TMEM49	+	NA	-4.18	0.52	1.05E-15	17	40015361	40017813	Trans
G_CSF	**cg16936953**	17	57915665	TMEM49	+	NA	-4.11	0.57	4.06E-13	17	40015361	40017813	Trans

Supplementary Table 5. Bidirectional Mendelian Randomisation analyses to test for causal relationships between DNA methylation and Olink® protein levels (Wald ratio test)

		DNA methylation affecting protein levels			***Protein levels causally affecting DNA methylation***		
**Instrumental Variable**	**CpG Site**	**Beta**	**SE**	**P Value**	**Beta**	**SE**	**P Value**
MATN3							
rs3731663	cg24416238 (*MATN3*)	0.80	0.09	**6.60E-18**	1.24	0.09	**4.50E-40**
MDGA1							
rs6938061	cg20053110 (*MDGA1*)	1.00	0.05	**1.40E-87**	0.99	0.02	**1.20E-102**
rs6458011	cg20053110 (*MDGA1*)	-1.16	0.06	**1.40E-87**	-0.84	0.04	**8.00E-111**
NEP							
rs4687657	cg11645453 (*ITIH4*)	0.63	0.07	**2.60E-19**	1.58	0.07	**1.90E-111**
rs4687657	cg18404041 (*ITIH1*)	-0.56	0.06	**2.60E-19**	-1.77	0.21	**3.54E-17**
