# Supplementary material for: Genome and epigenome wide studies of neurological protein biomarkers in the Lothian Birth Cohort 1936
Source: Nat Commun. 2019 Jul 18;10:3160. doi: 10.1038/s41467-019-11177-x (PMC6639385; doi:10.1038/s41467-019-11177-x)
Supplement: Supplementary file 5 — Supplementary Dataset 4 [file 41467_2019_11177_MOESM5_ESM.docx]

**Supplementary Data 4.** Tissue-specific patterns of expression for genes associated with Olink neurology proteins exhibiting genome-wide significant CpG sites. Differentially expressed gene sets in 53 tissue types (GTEx v7) with Bonferroni-corrected P values of < 0.05 and an absolute log fold change ≥ 0.58 are shown in red (default settings). Differentially expressed genes (DEG).

*CRTAM*

**
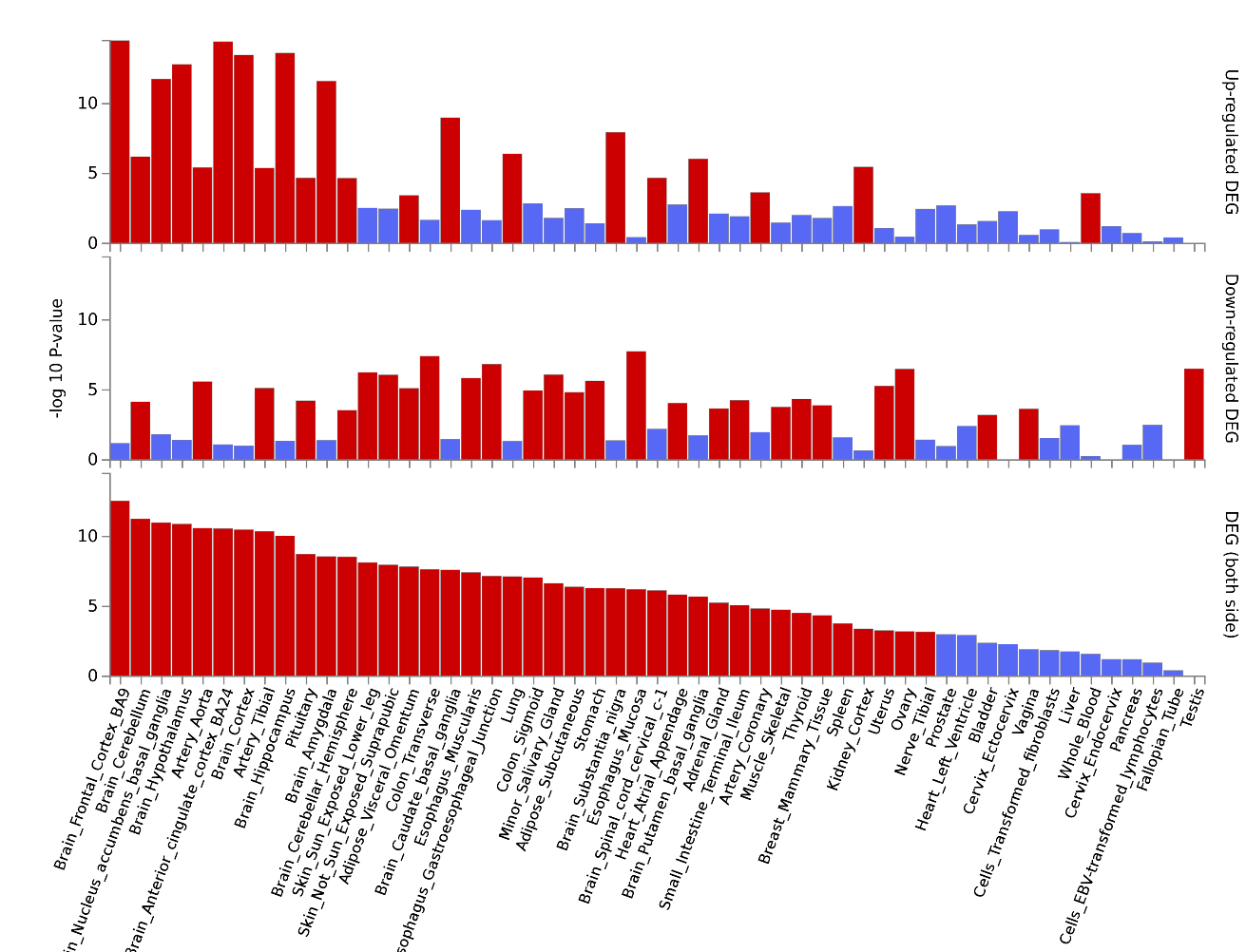
**

*G-CSF*

**
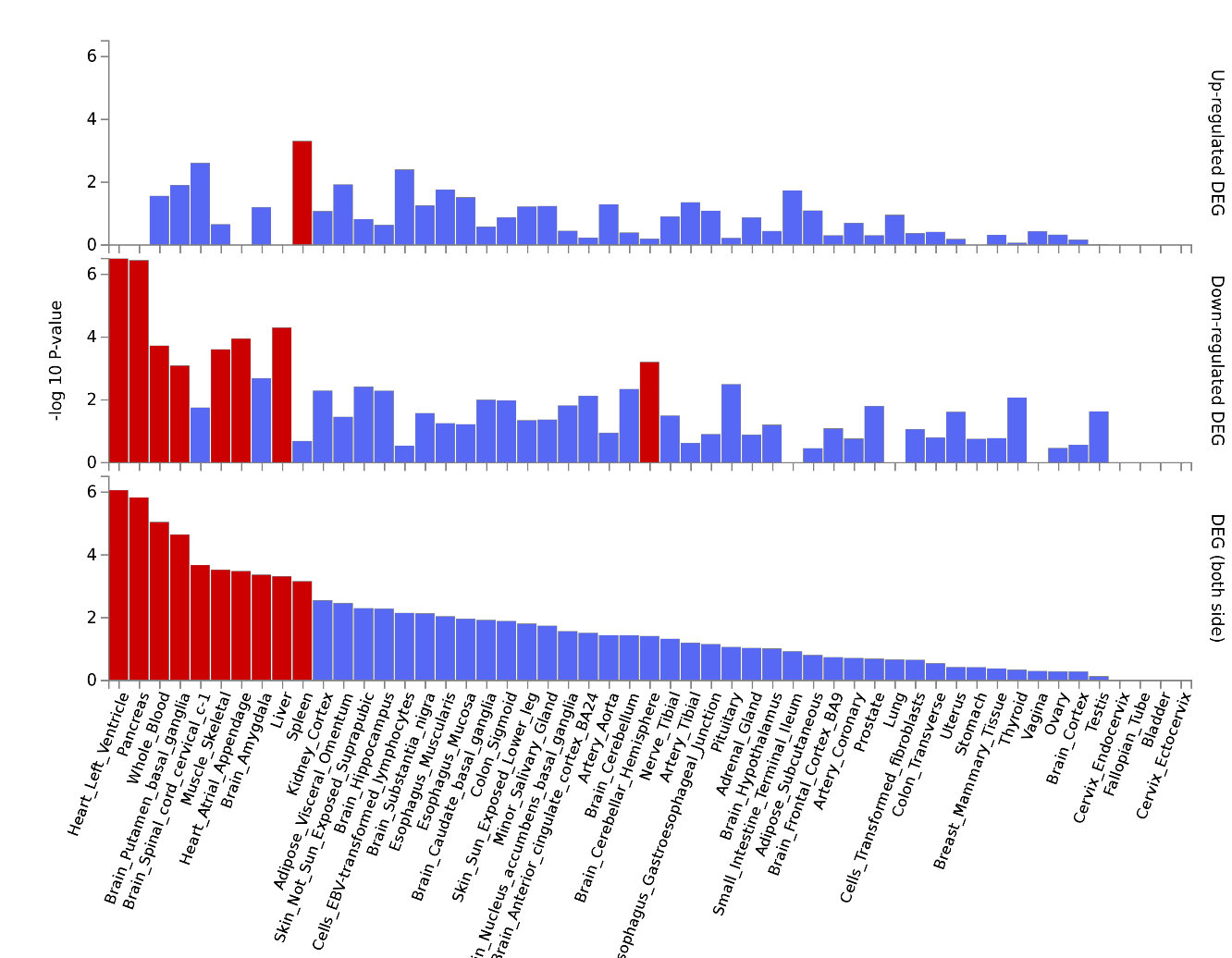
**

*MDGA1*

**
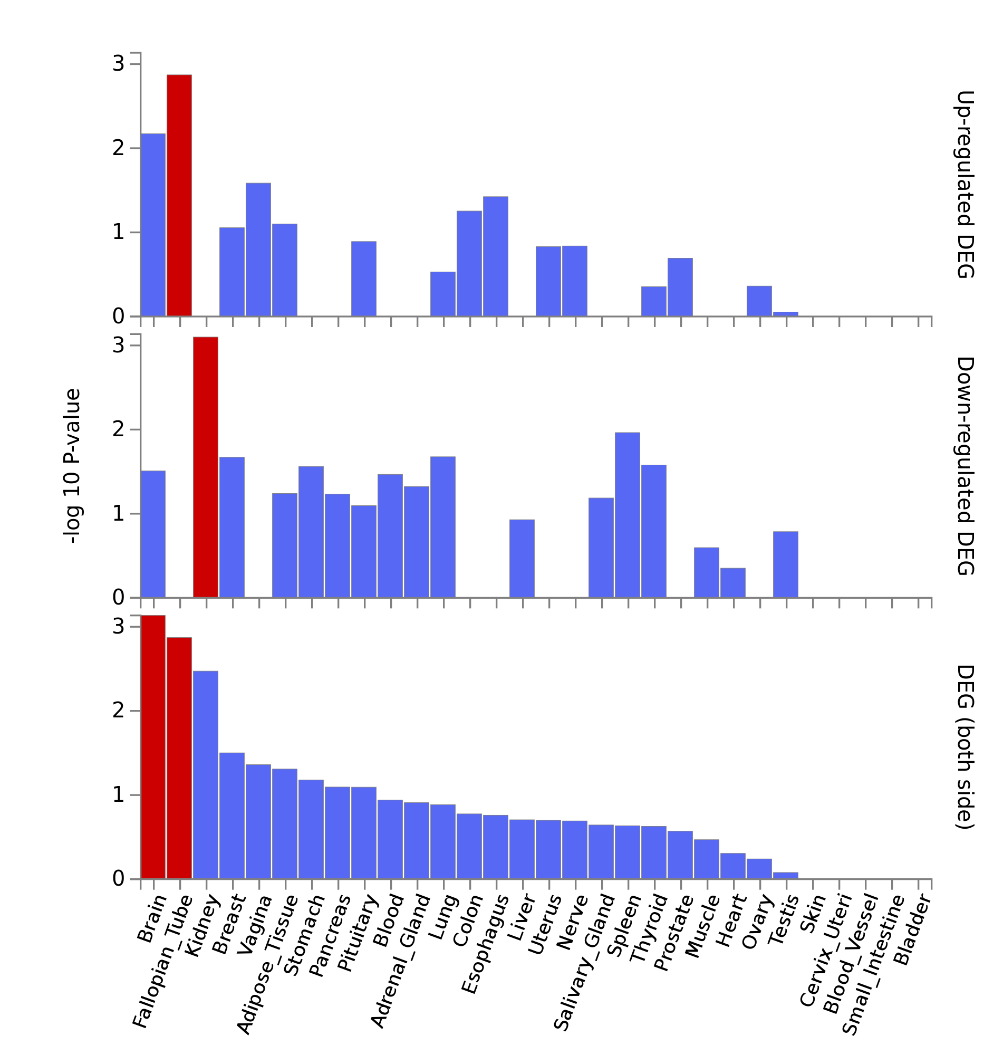
**

*NEP*

**
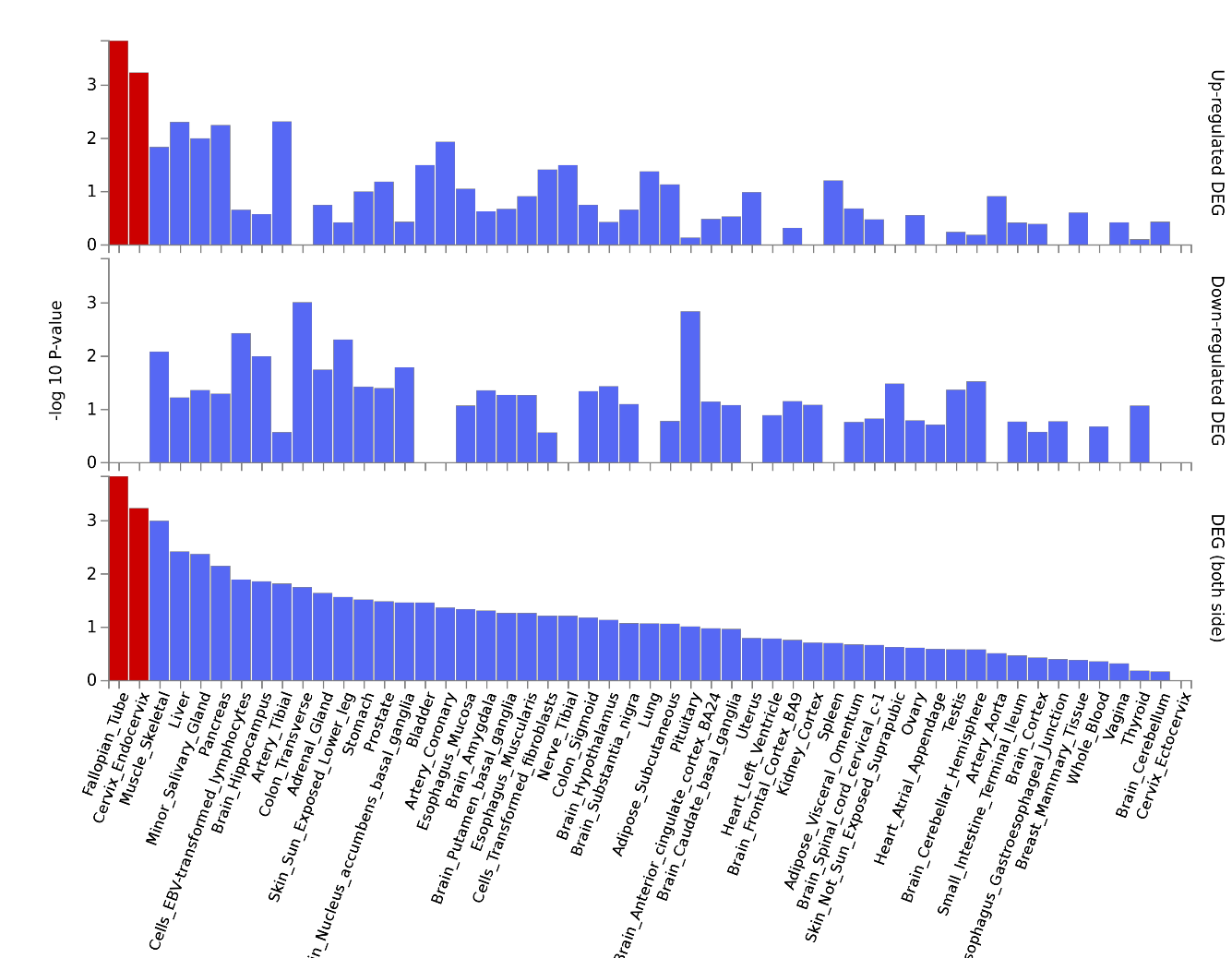
**

*SIGLEC1*

**
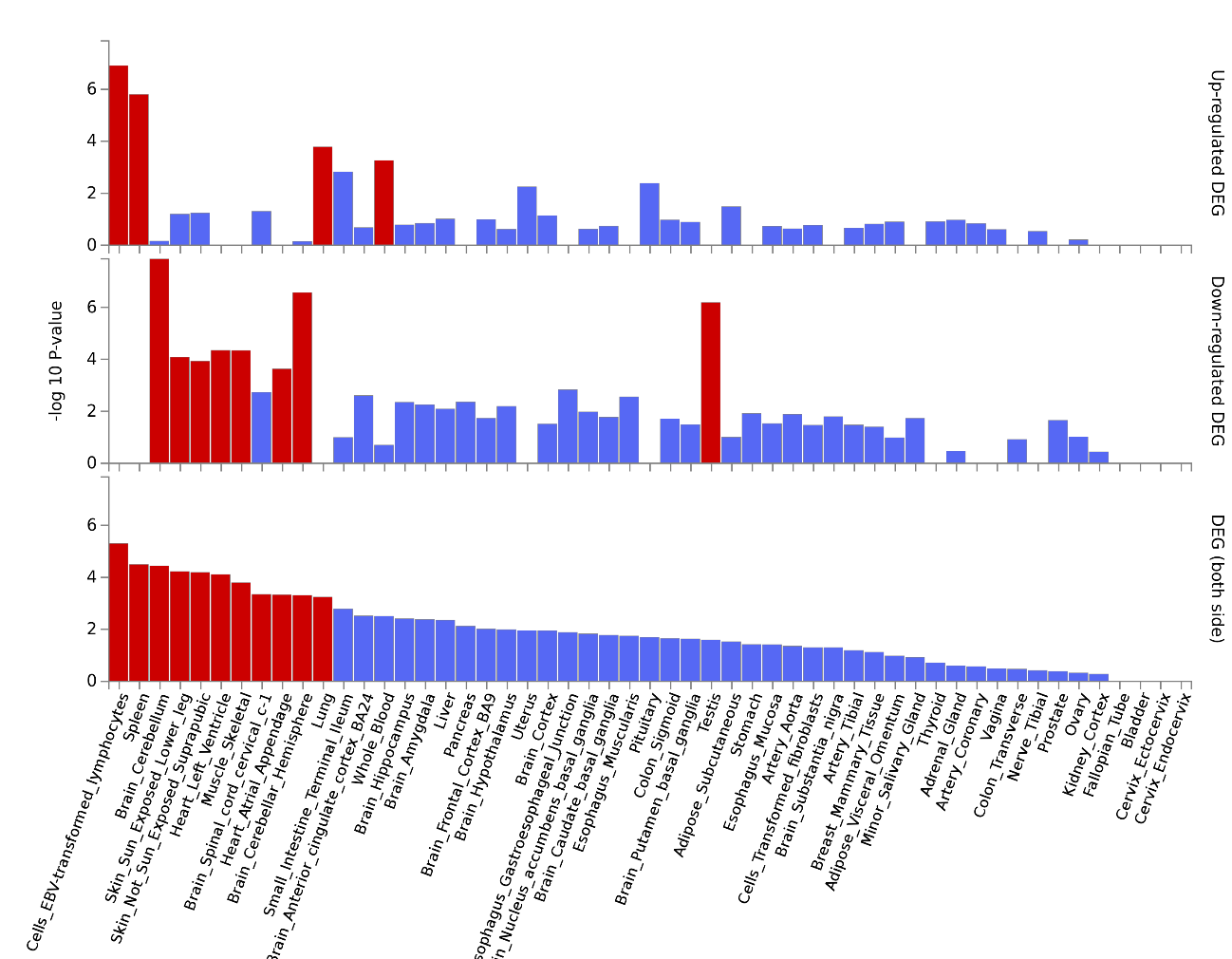
**
