# Supplementary material for: Genome and epigenome wide studies of neurological protein biomarkers in the Lothian Birth Cohort 1936
Source: Nat Commun. 2019 Jul 18;10:3160. doi: 10.1038/s41467-019-11177-x (PMC6639385; doi:10.1038/s41467-019-11177-x)
Supplement: Supplementary file 11 — Supplementary Dataset 10 [file 41467_2019_11177_MOESM11_ESM.pdf]

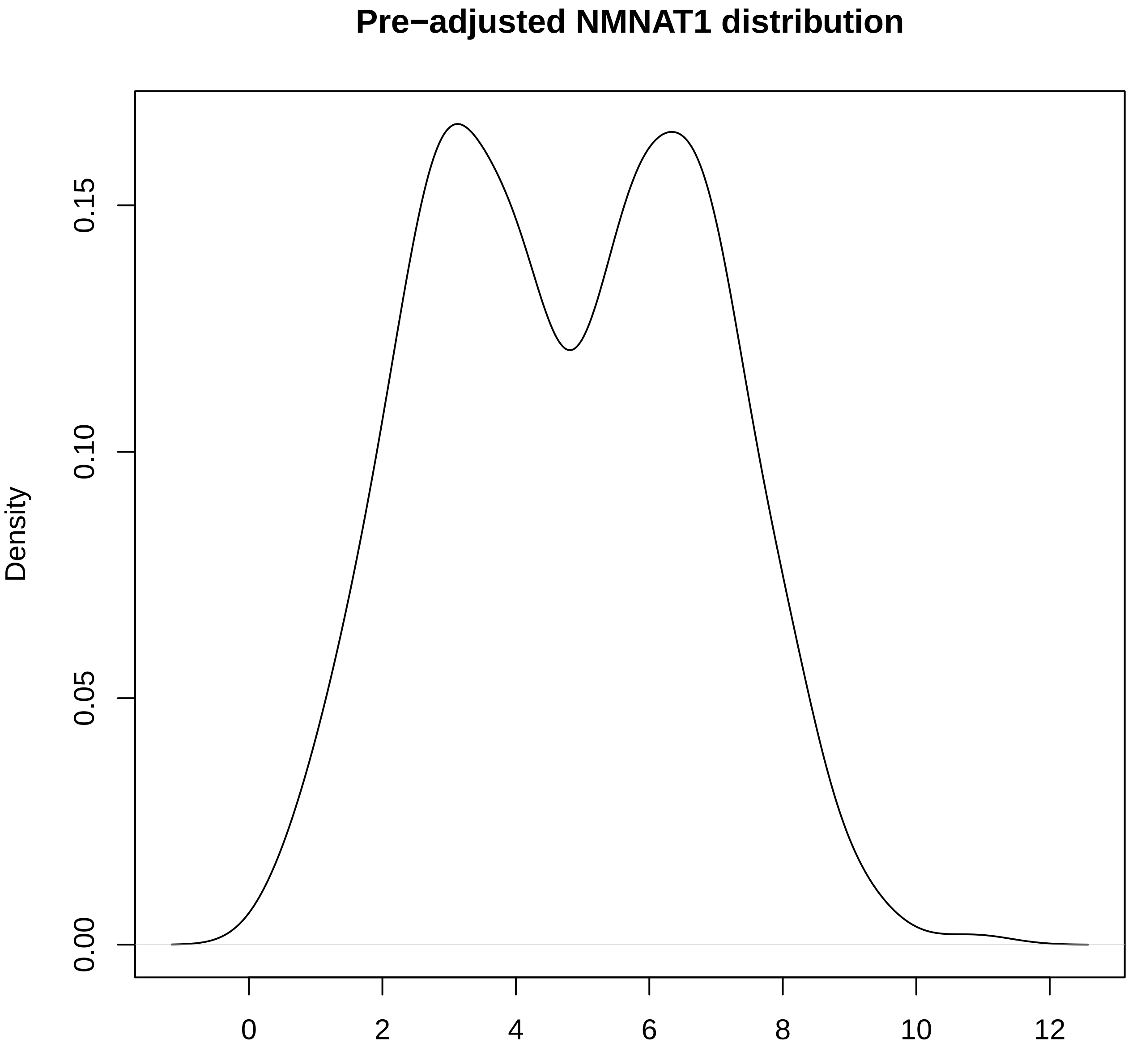

Pre−adjusted NMNAT1 distribution
Density
0.00
0.05
0.10
0.15
0
2
4
6
8
10
12

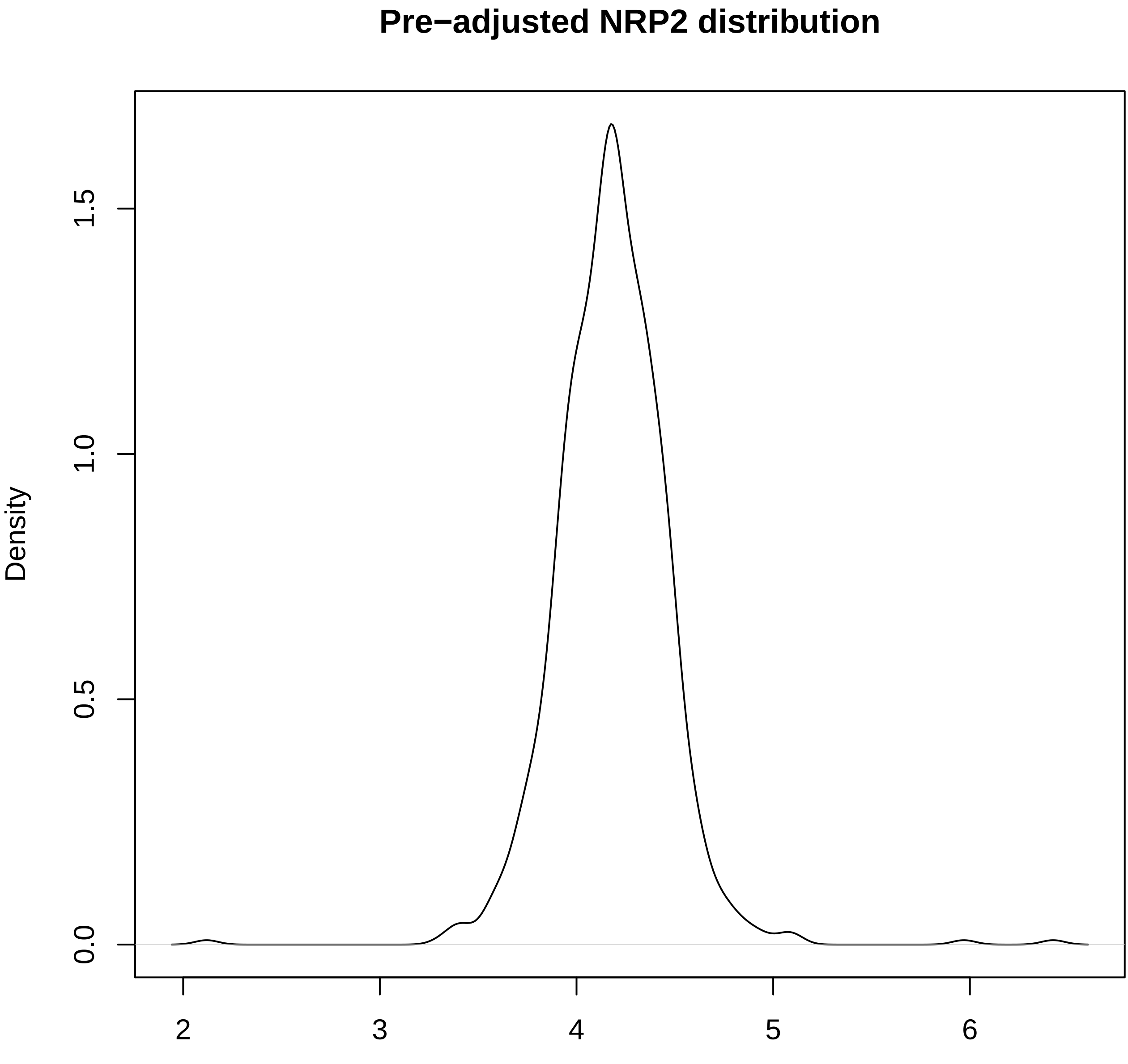

Pre−adjusted NRP2 distribution
Density
0.0
0.5
1.0
1.5
2
3
4
5
6

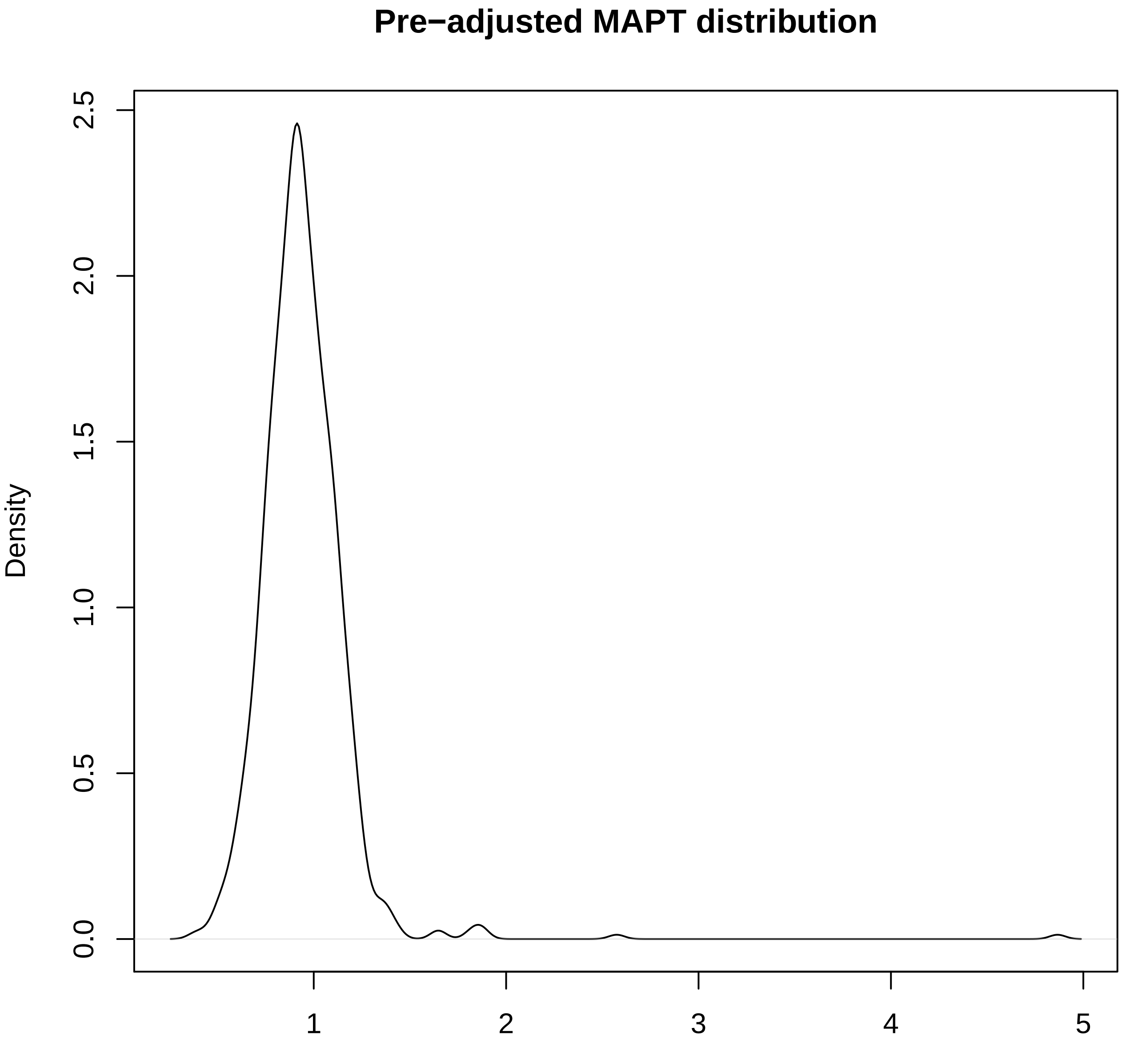
Pre−adjusted MAPT distribution
Density
0.0
0.5
1.0
1.5
2.0
2.5
1
2
3
4
5

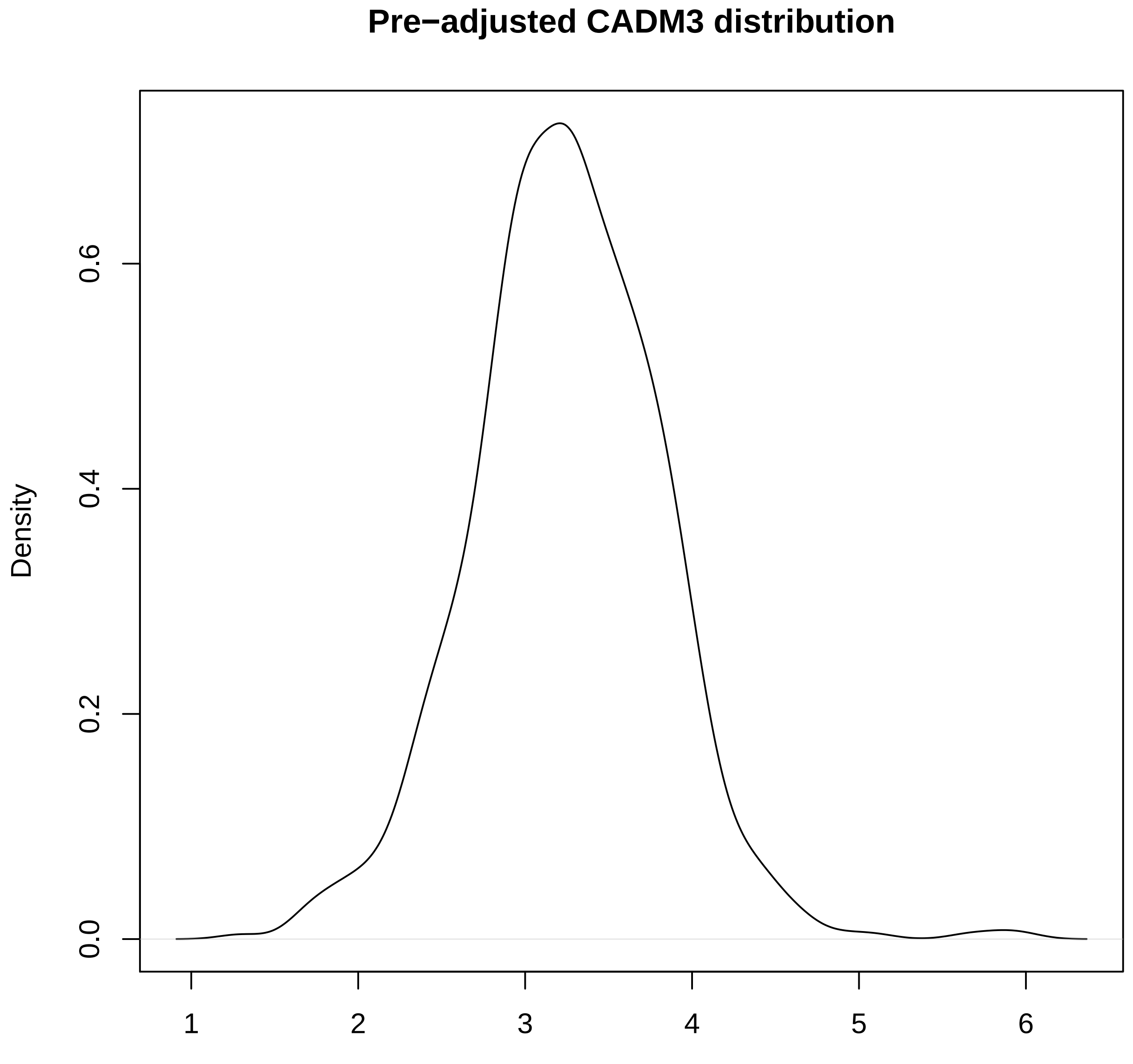
Pre−adjusted CADM3 distribution
Density
0.0
0.2
0.4
0.6
1
2
3
4
5
6

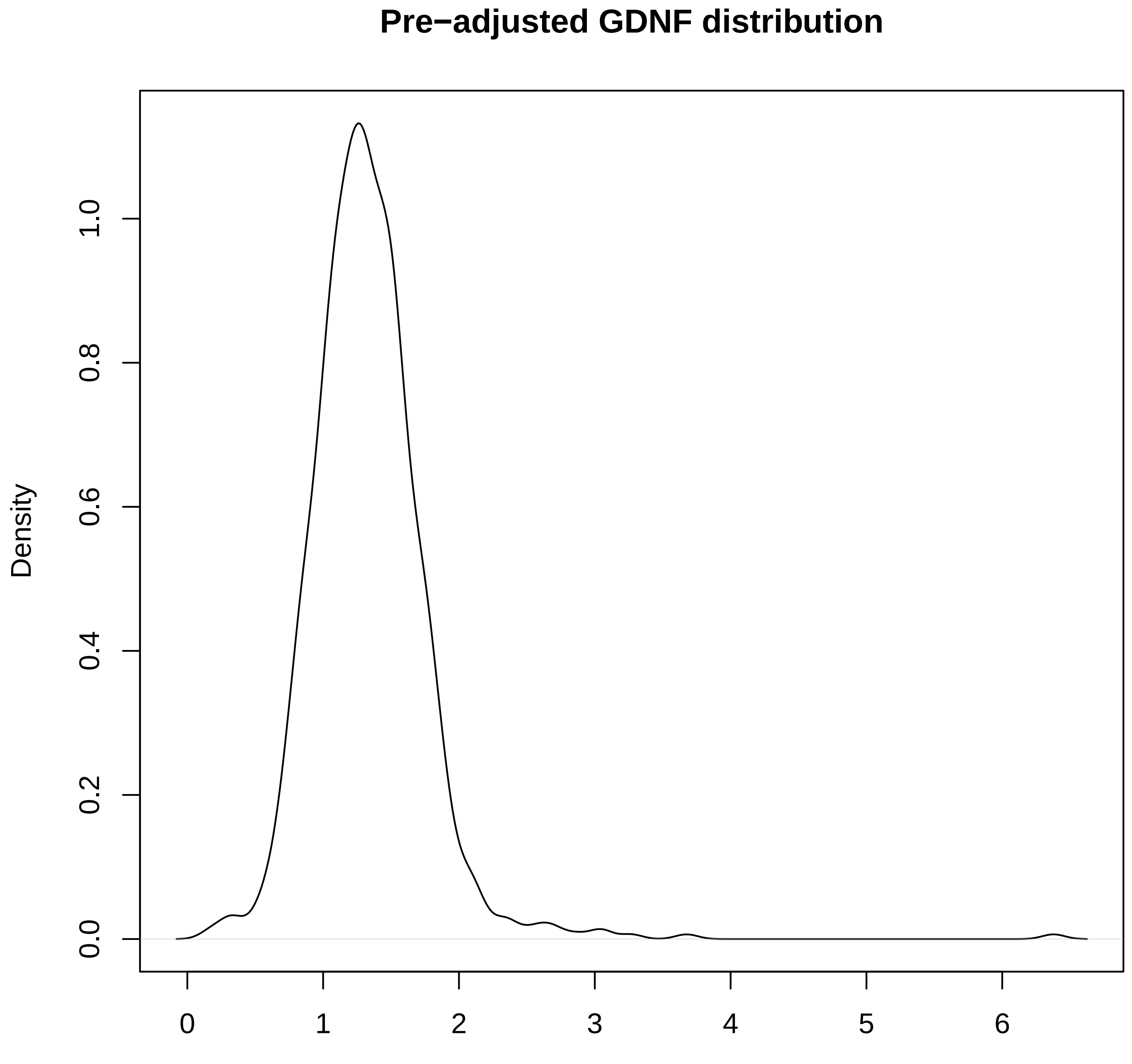
Pre−adjusted GDNF distribution
Density
0.0
0.2
0.4
0.6
0.8
1.0
0
1
2
3
4
5
6

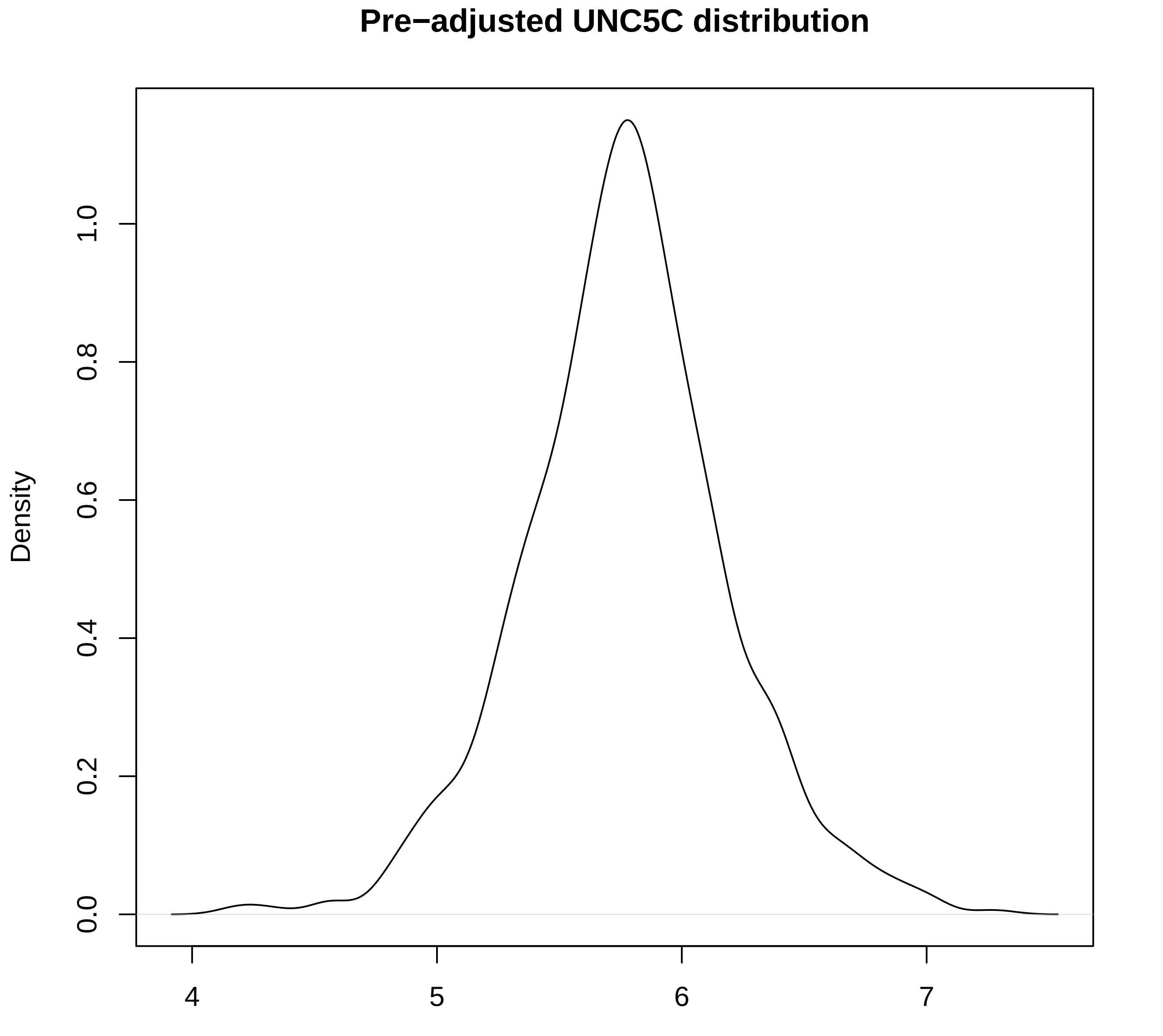
Pre−adjusted UNC5C distribution
Density
0.0
0.2
0.4
0.6
0.8
1.0
4
5
6
7

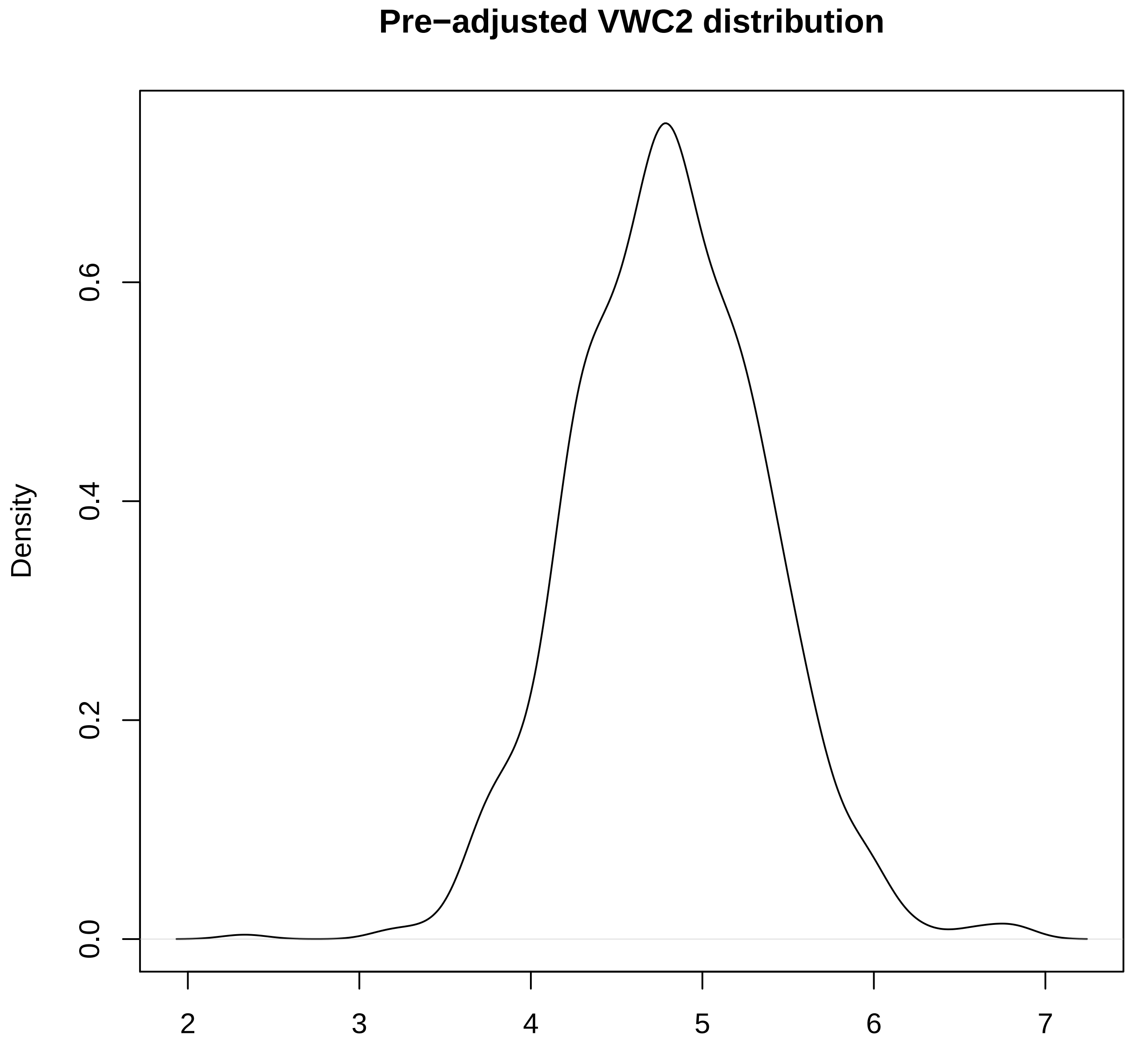
Pre−adjusted VWC2 distribution
Density
0.0
0.2
0.4
0.6
2
3
4
5
6
7

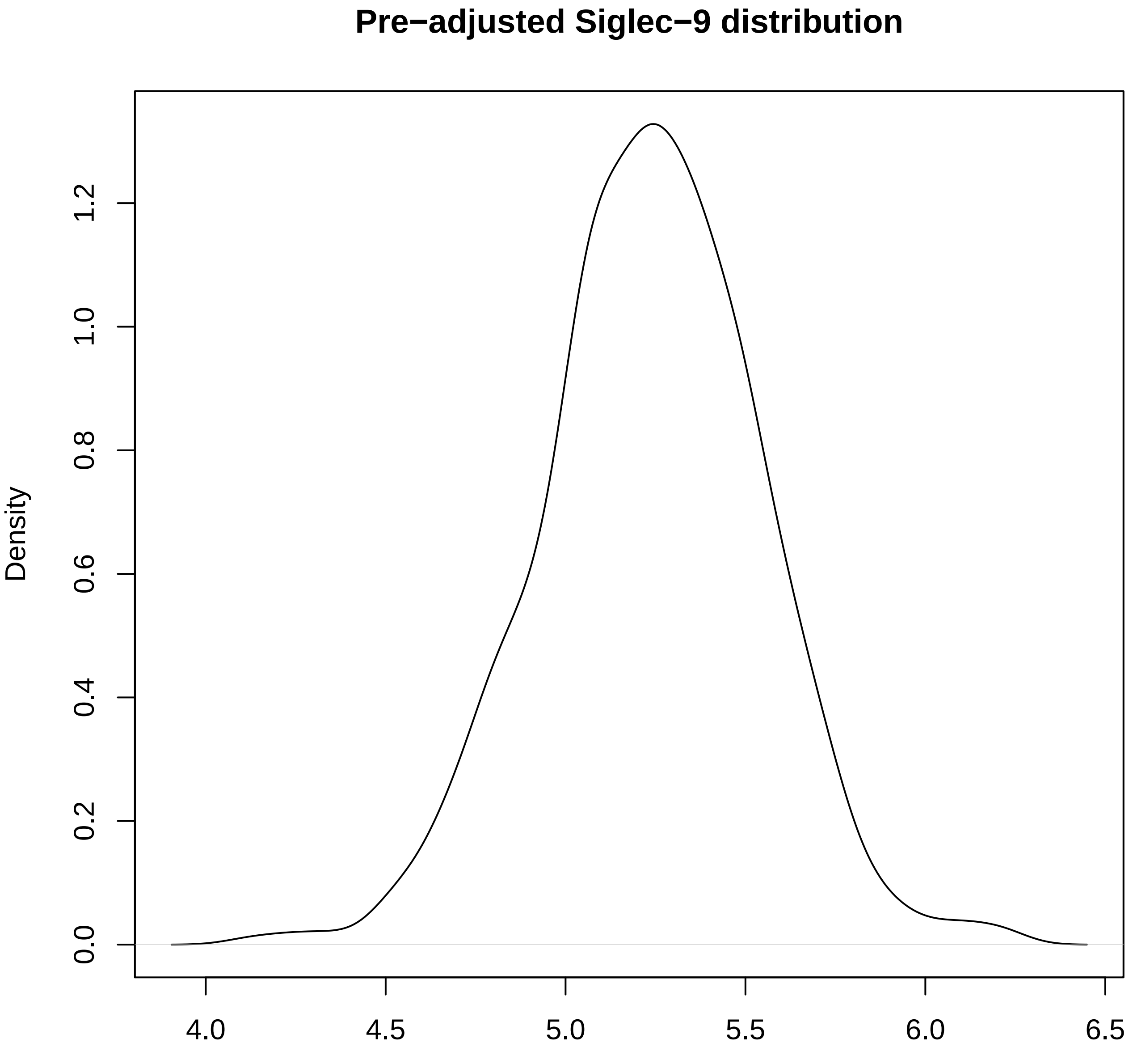
Pre−adjusted Siglec−9 distribution
Density
0.0
0.2
0.4
0.6
0.8
1.0
1.2
4.0
4.5
5.0
5.5
6.0
6.5

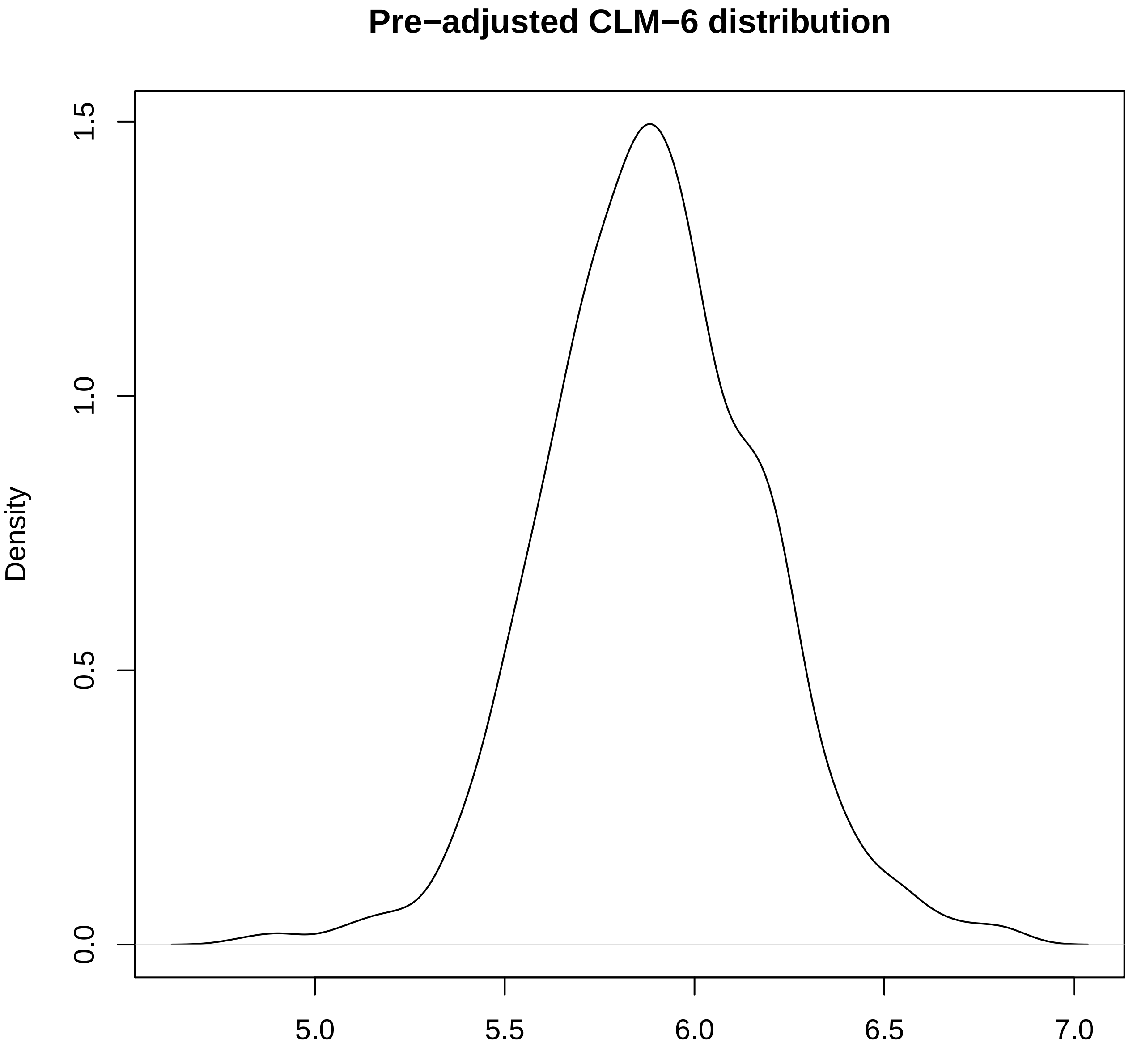

Pre−adjusted CLM−6 distribution
Density
0.0
0.5
1.0
1.5
5.0
5.5
6.0
6.5
7.0

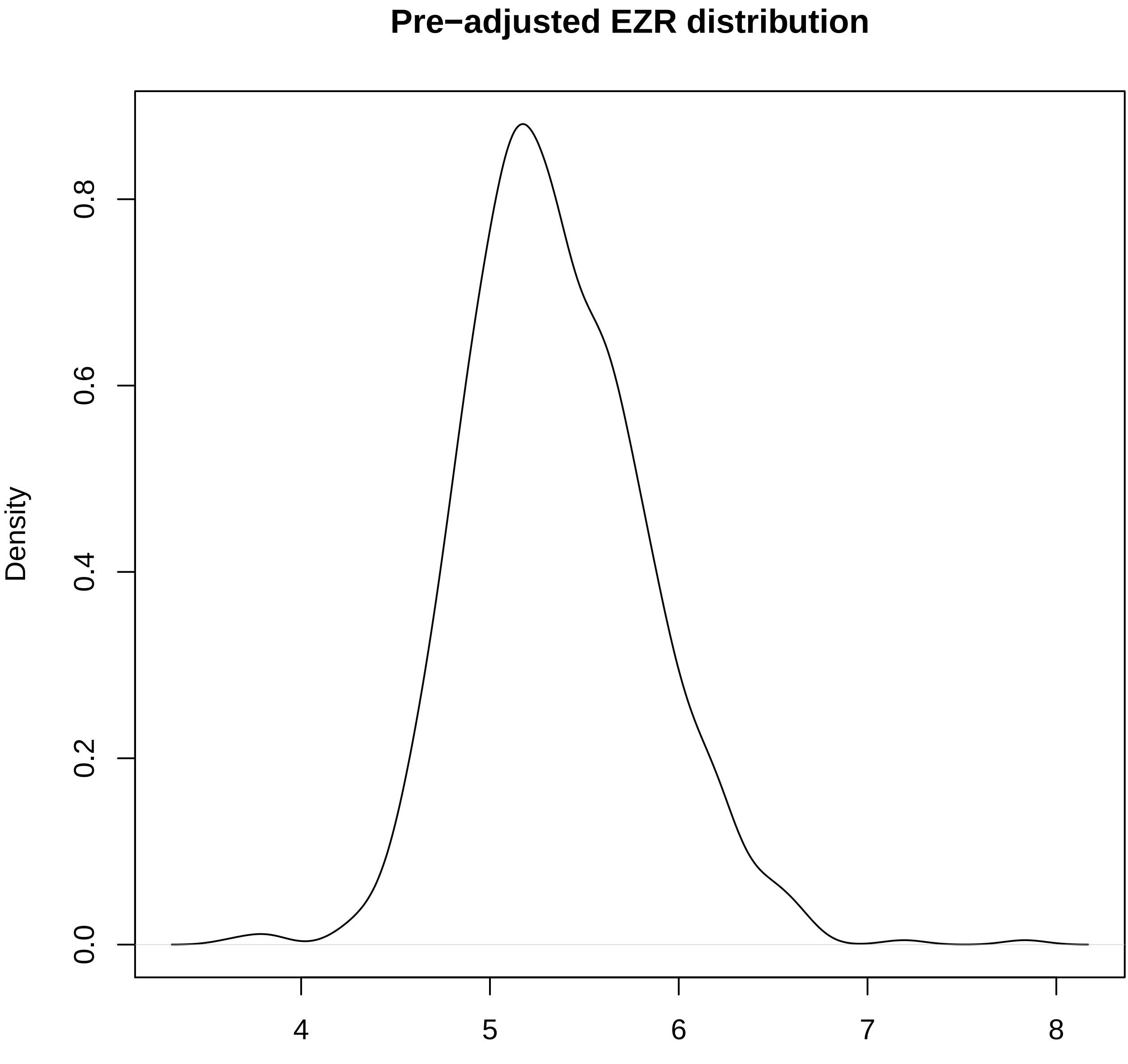
Pre−adjusted EZR distribution
Density
0.0
0.2
0.4
0.6
0.8
4
5
6
7
8

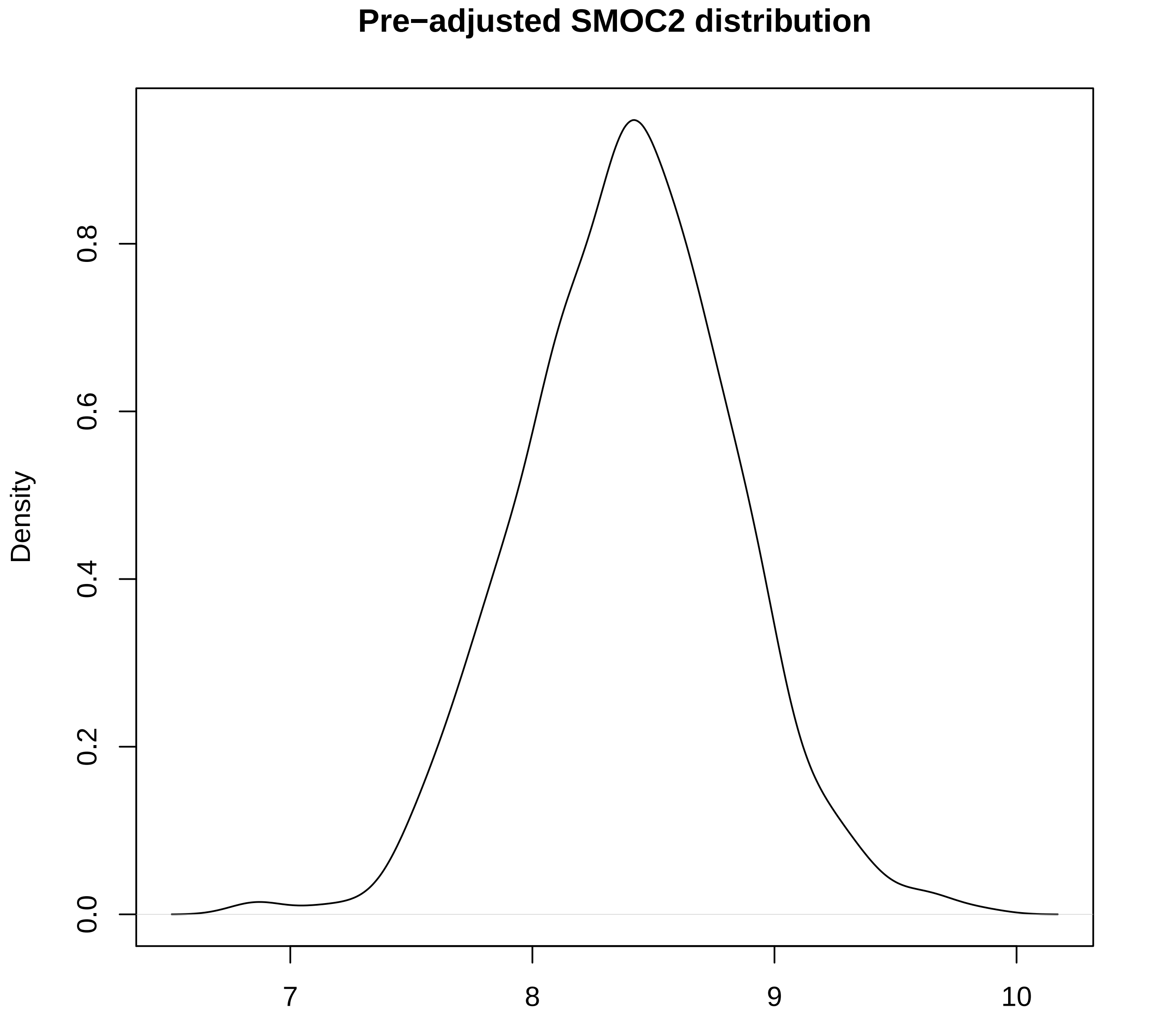
Pre−adjusted SMOC2 distribution
Density
0.0
0.2
0.4
0.6
0.8
7
8
9
10

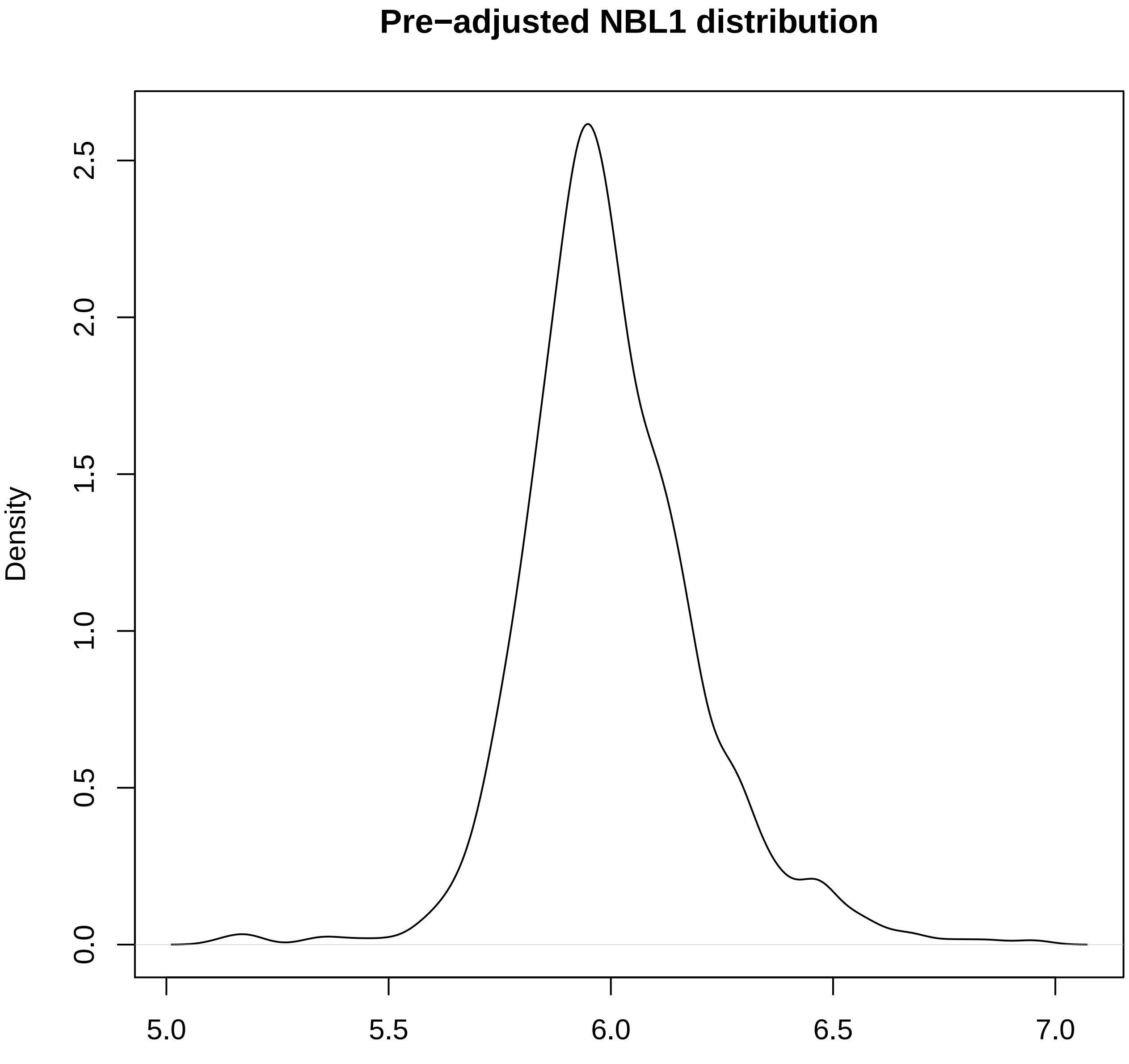
Pre−adjusted NBL1 distribution
Density
0.0
0.5
1.0
1.5
2.0
2.5
5.0
5.5
6.0
6.5
7.0

Pre−adjusted EFNA4 distribution

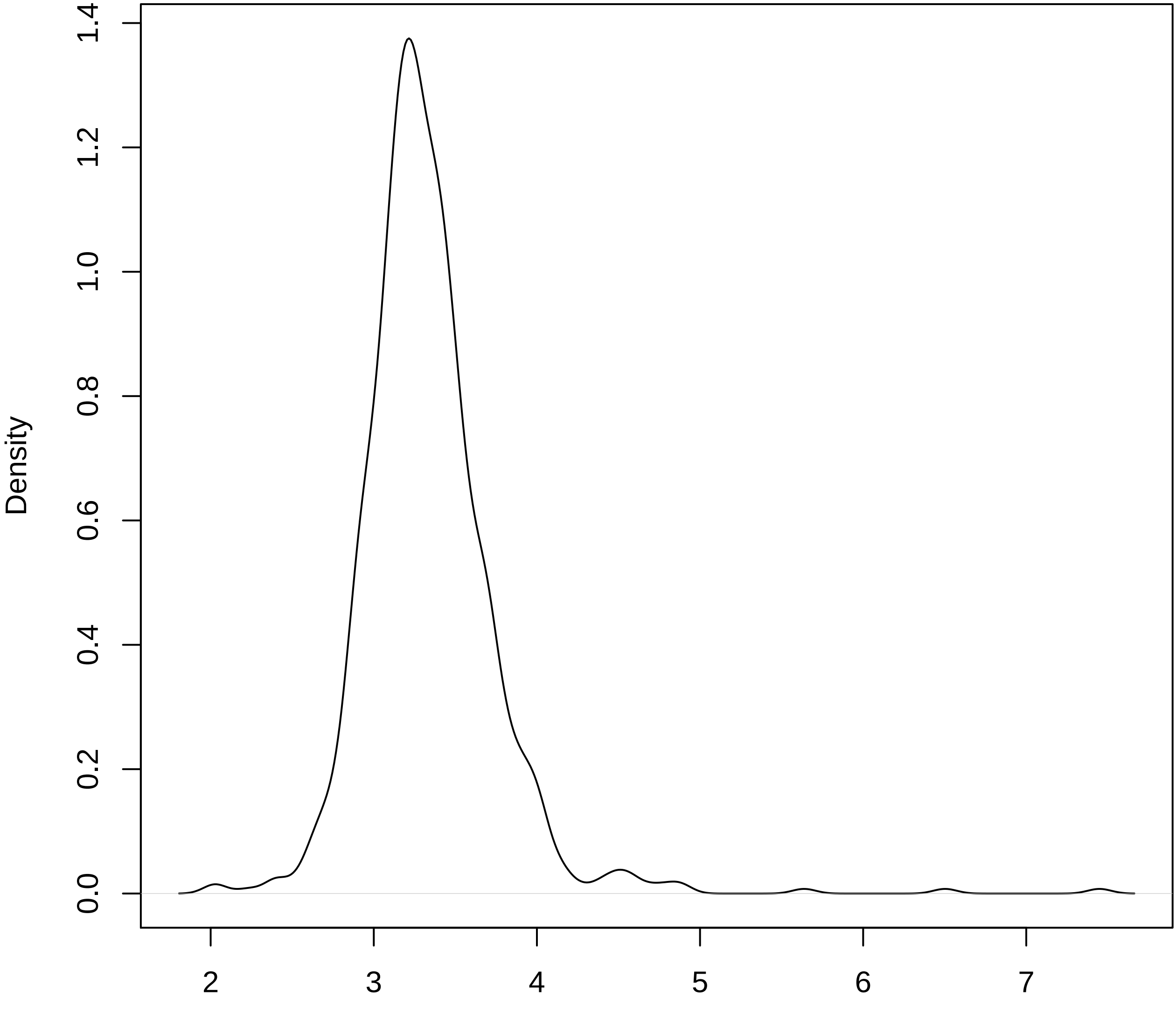

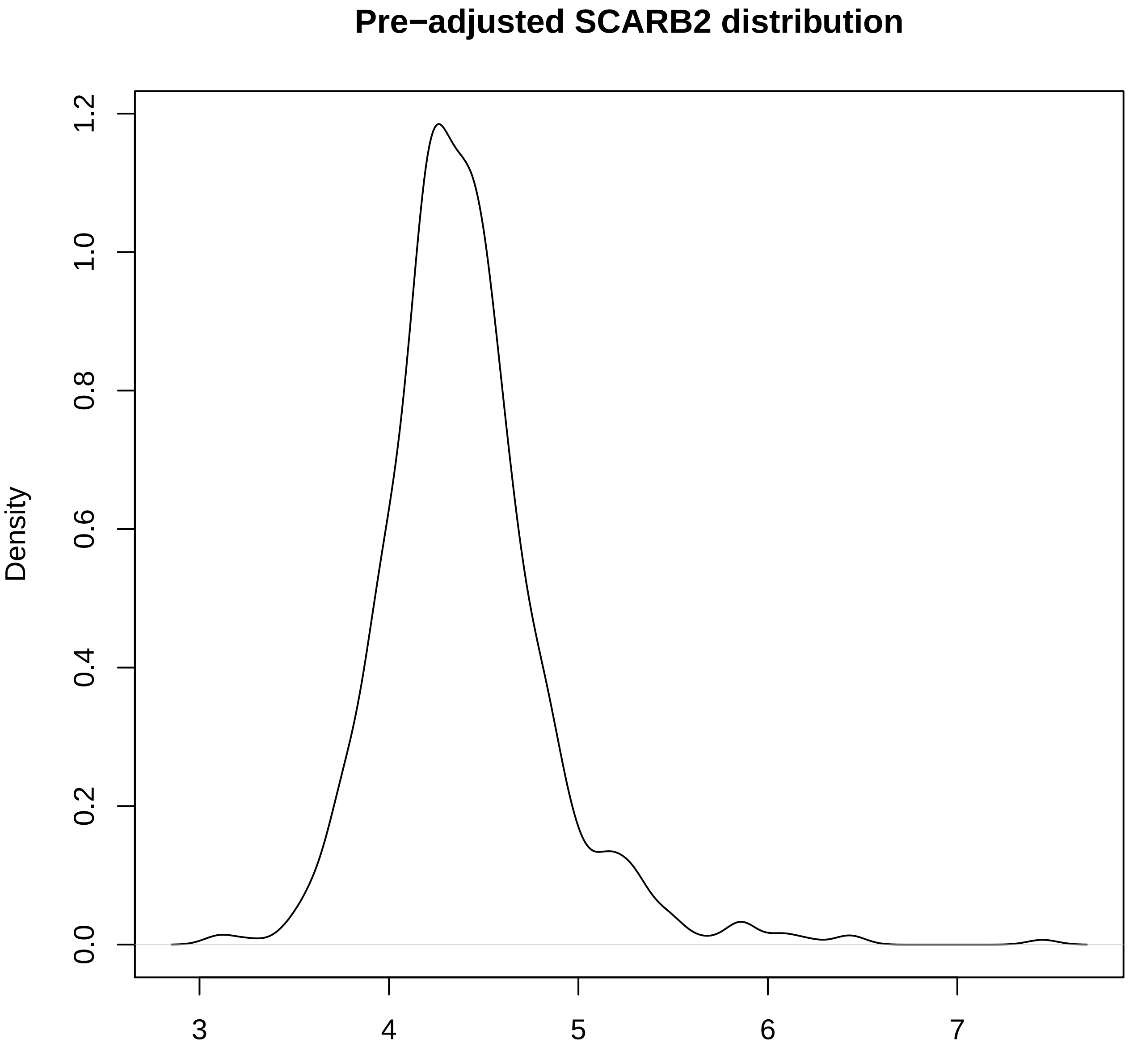
Pre−adjusted SCARB2 distribution
Density
0.0
0.2
0.4
0.6
0.8
1.0
1.2
3
4
5
6
7

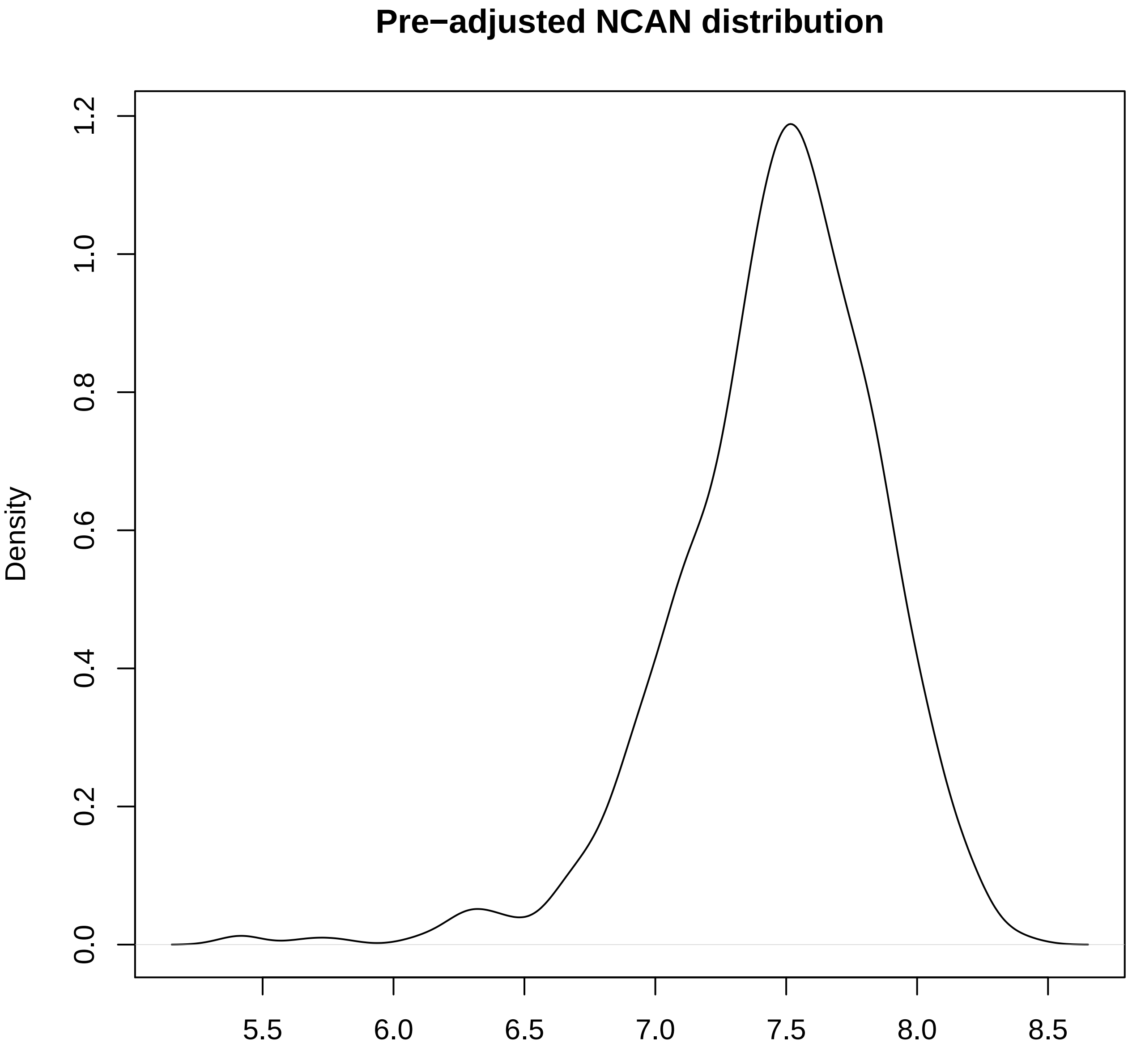

Pre−adjusted NCAN distribution
Density
0.0
0.2
0.4
0.6
0.8
1.0
1.2
5.5
6.0
6.5
7.0
7.5
8.0
8.5

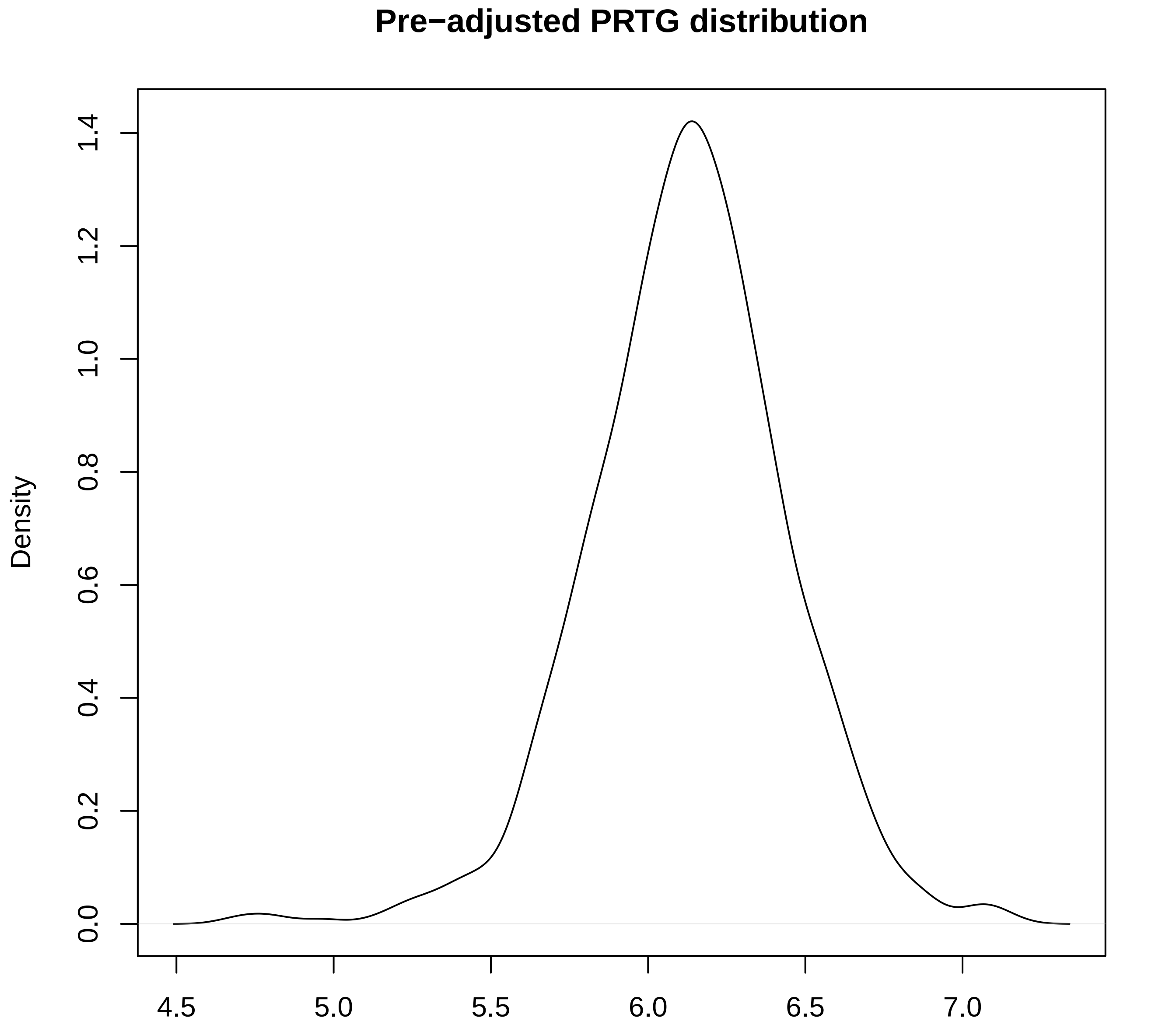
Pre−adjusted PRTG distribution
Density
0.0
0.2
0.4
0.6
0.8
1.0
1.2
1.4
4.5
5.0
5.5
6.0
6.5
7.0

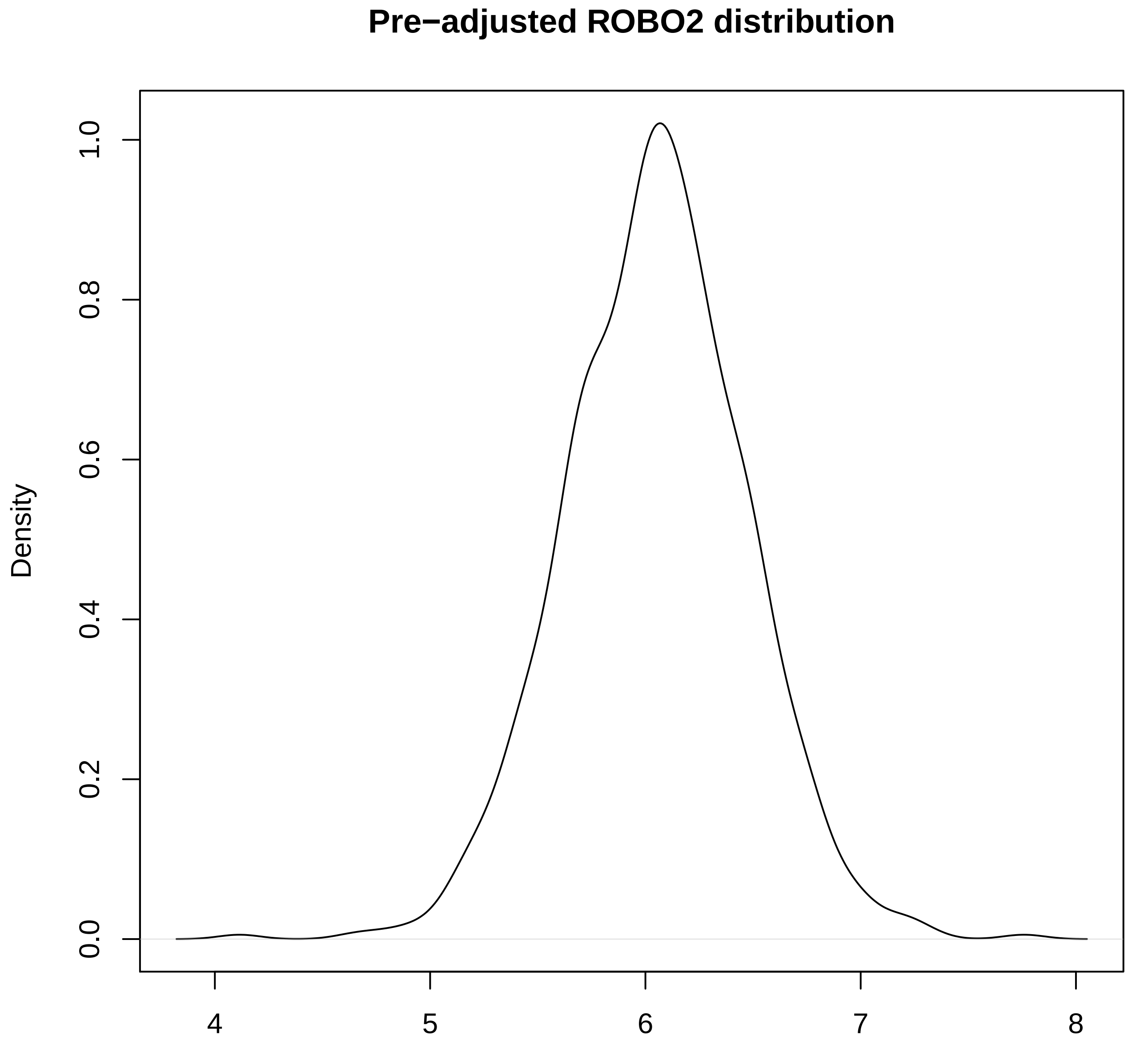

Pre−adjusted ROBO2 distribution
Density
0.0
0.2
0.4
0.6
0.8
1.0
4
5
6
7
8

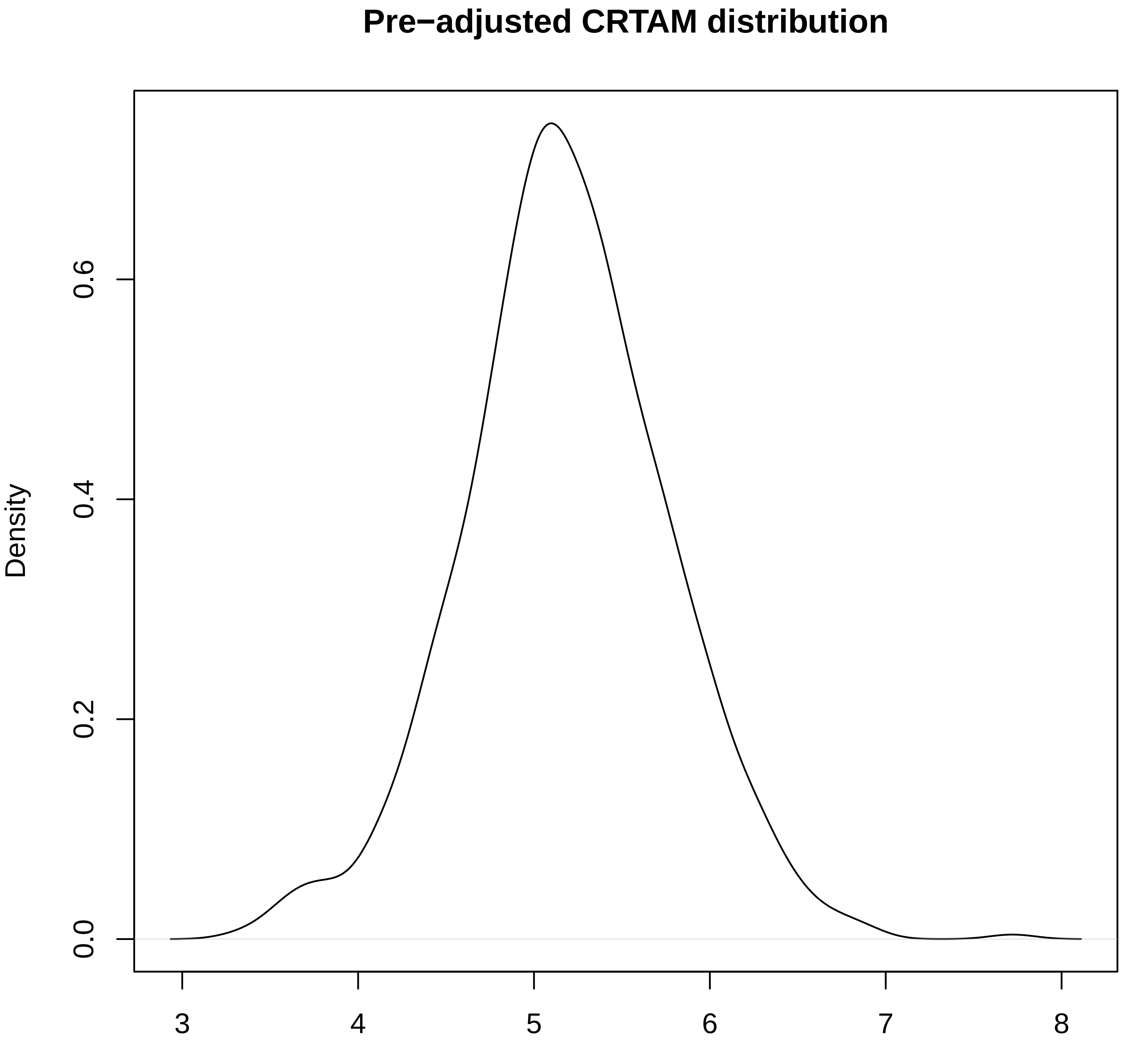
Pre−adjusted CRTAM distribution
Density
0.0
0.2
0.4
0.6
3
4
5
6
7
8

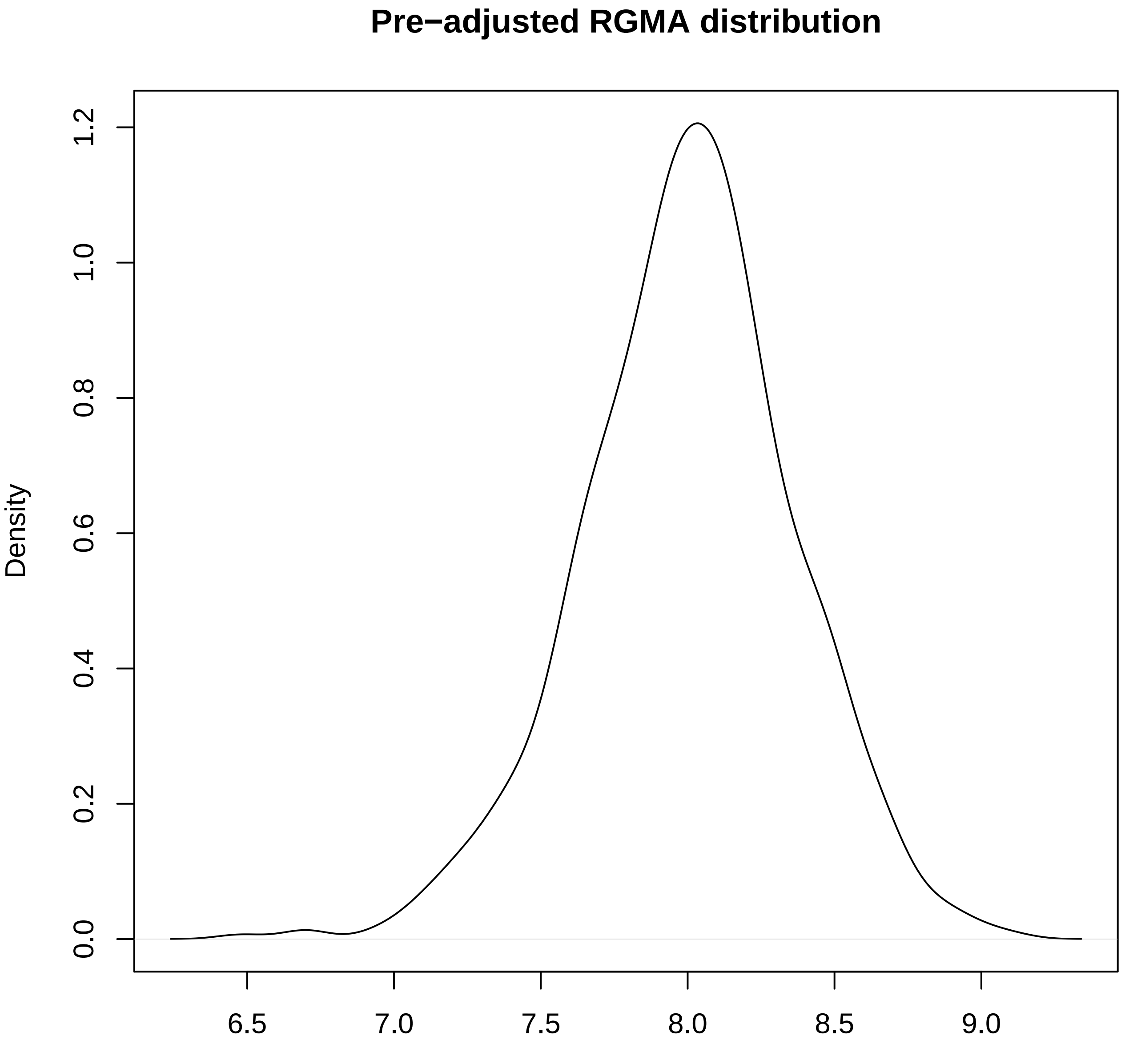

Pre−adjusted RGMA distribution
Density
0.0
0.2
0.4
0.6
0.8
1.0
1.2
6.5
7.0
7.5
8.0
8.5
9.0

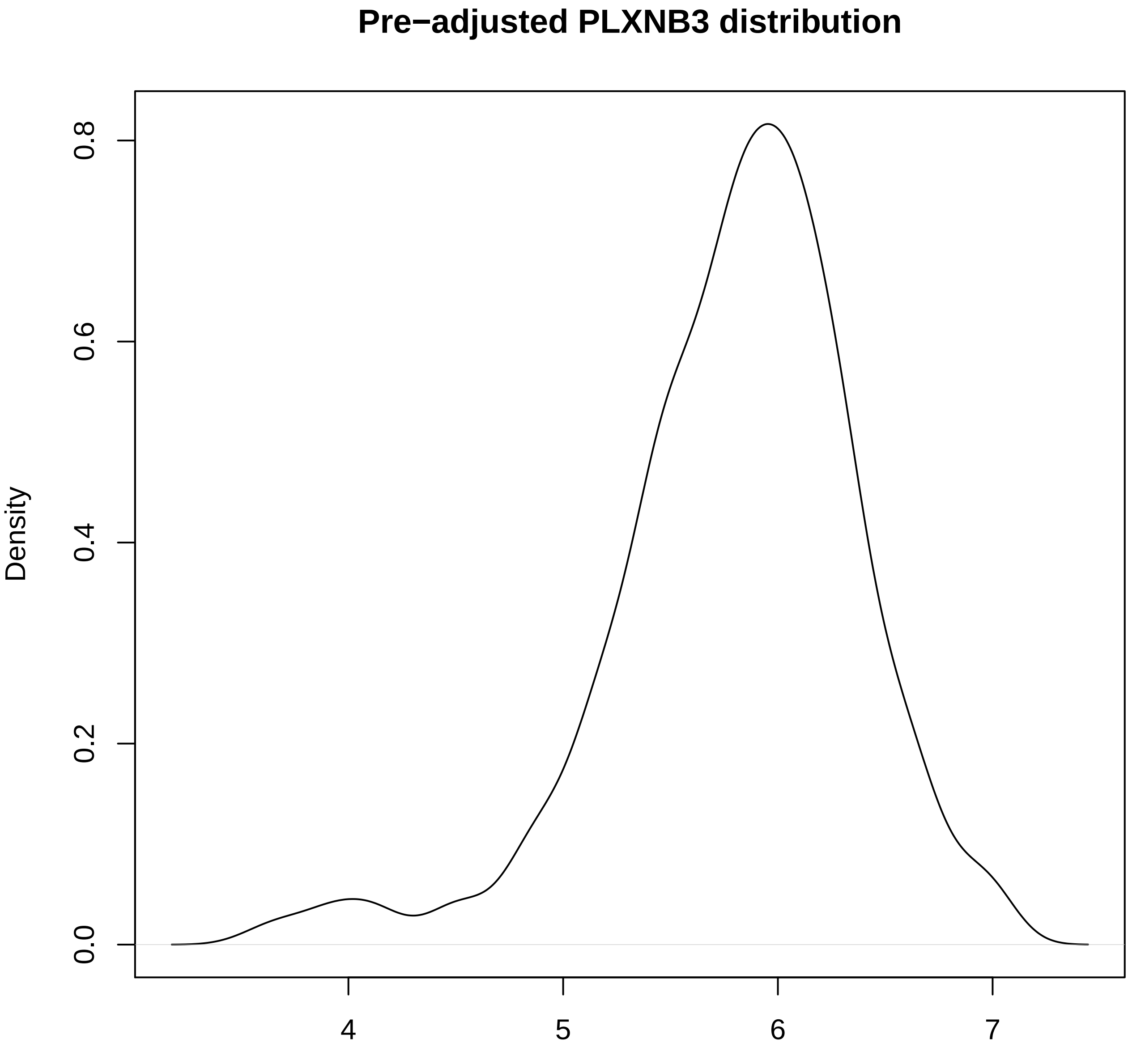
Pre−adjusted PLXNB3 distribution
Density
0.0
0.2
0.4
0.6
0.8
4
5
6
7

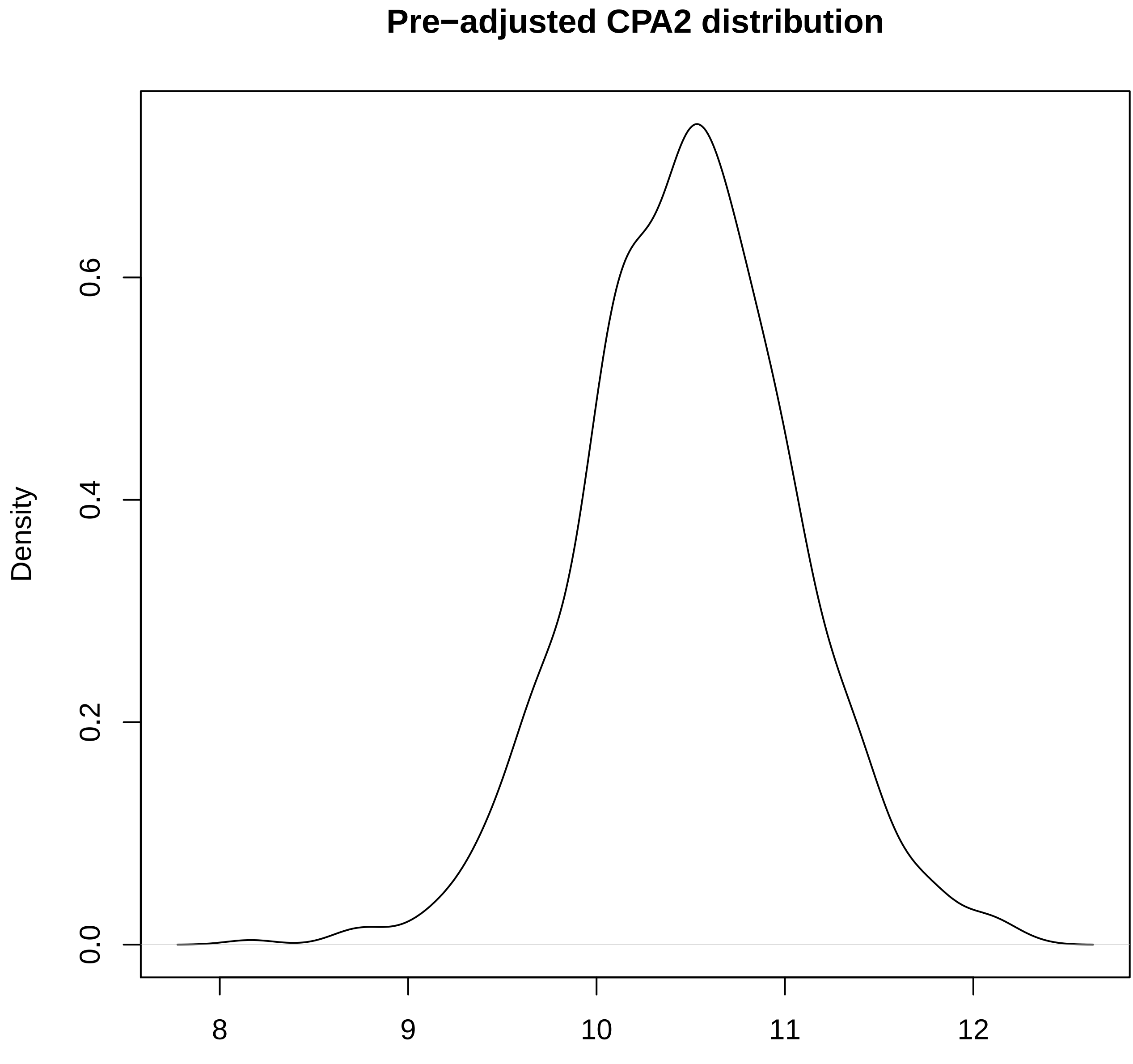
Pre−adjusted CPA2 distribution
Density
0.0
0.2
0.4
0.6
8
9
10
11
12

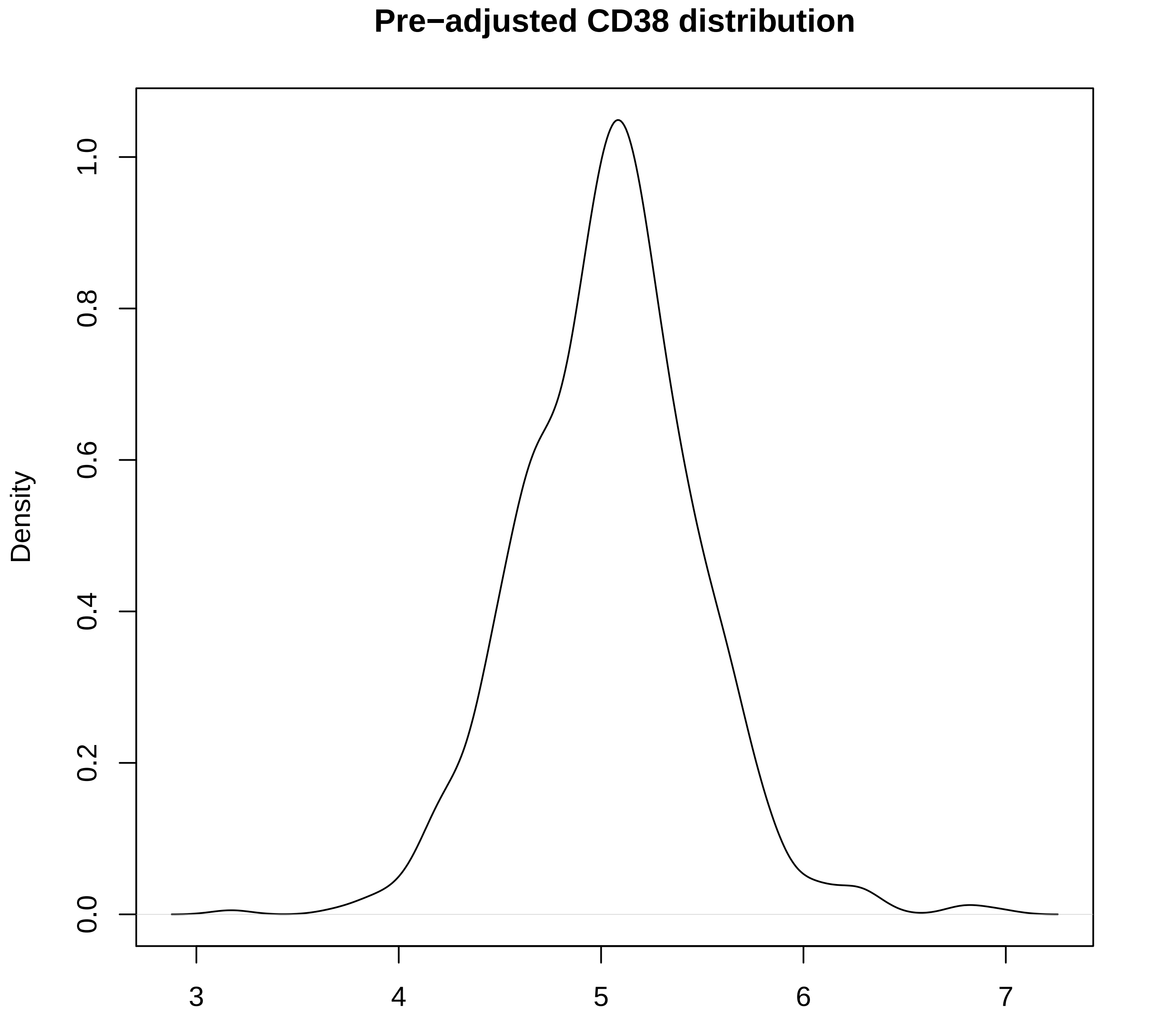
Pre−adjusted CD38 distribution
Density
0.0
0.2
0.4
0.6
0.8
1.0
3
4
5
6
7

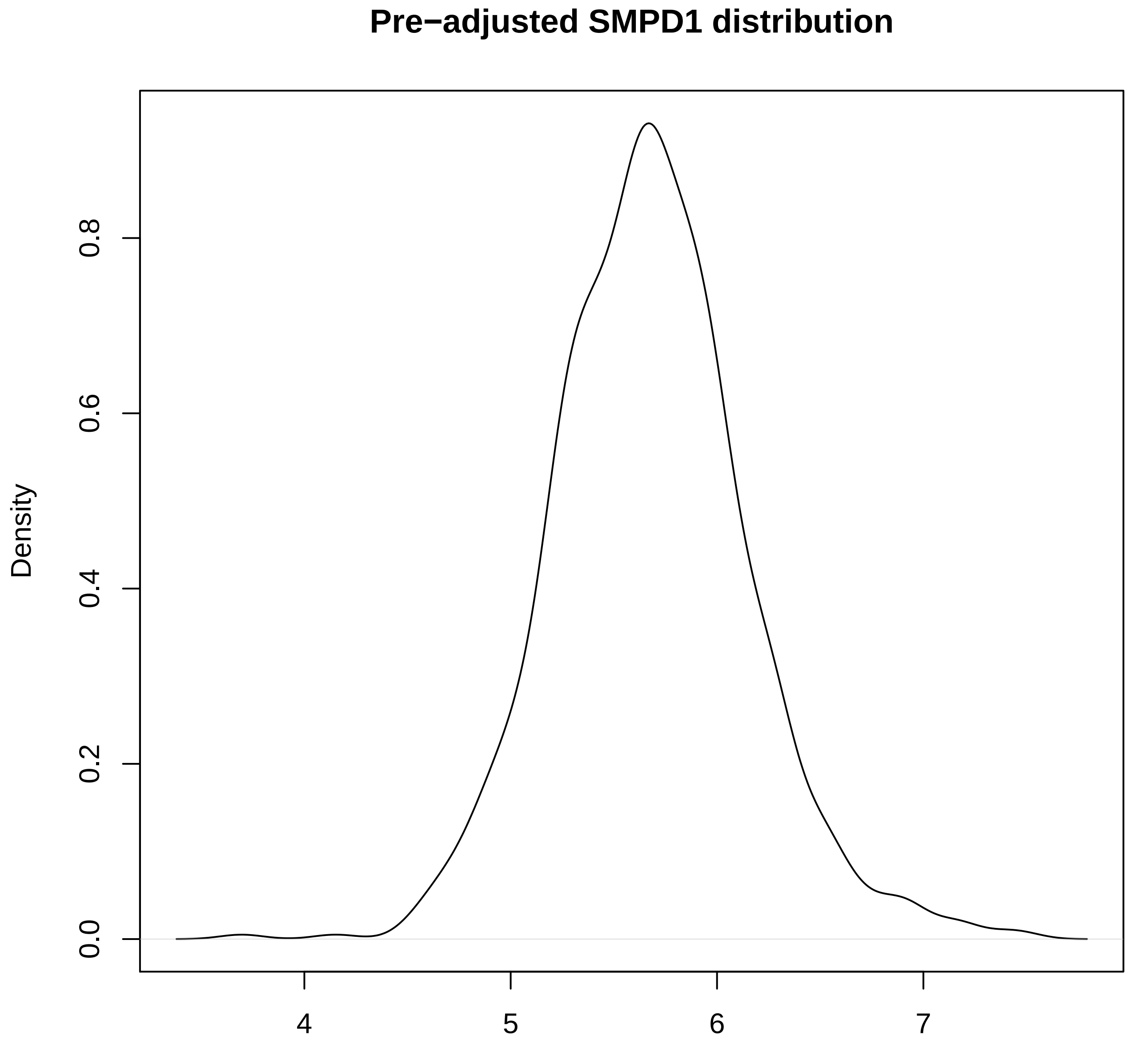
Pre−adjusted SMPD1 distribution
Density
0.0
0.2
0.4
0.6
0.8
4
5
6
7

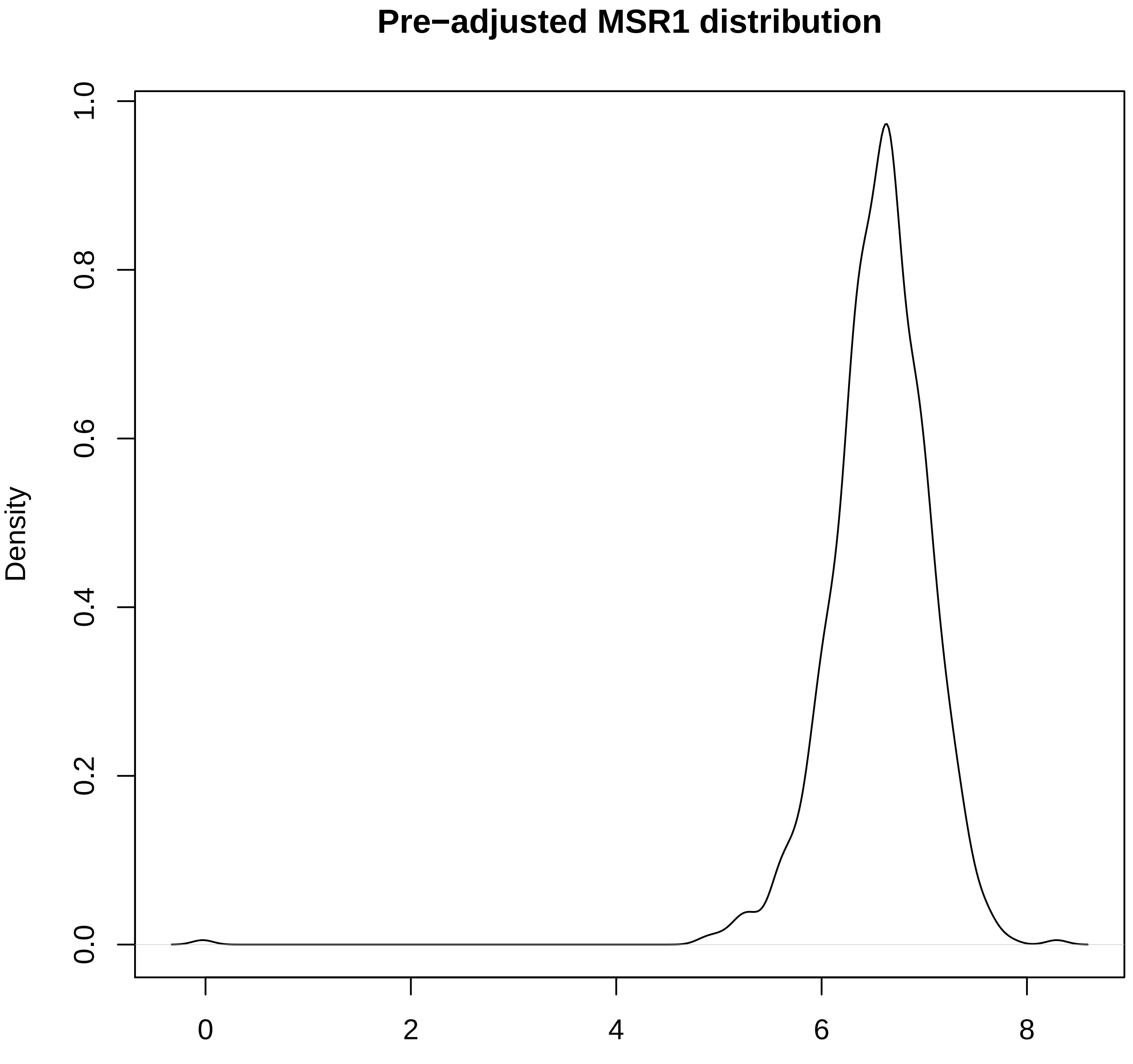
Pre−adjusted MSR1 distribution
Density
0.0
0.2
0.4
0.6
0.8
1.0
0
2
4
6
8

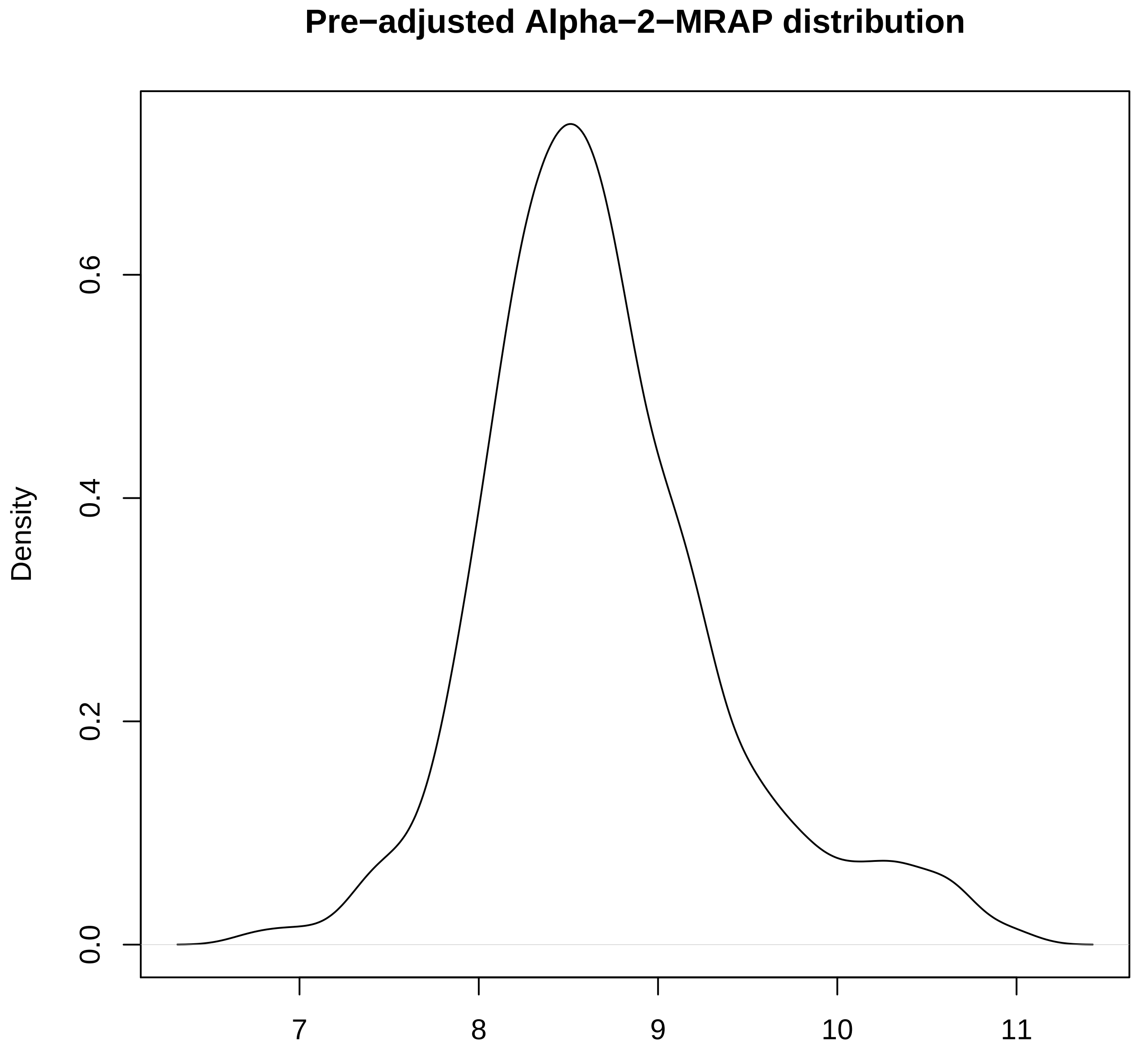
Pre−adjusted Alpha−2−MRAP distribution
Density
0.0
0.2
0.4
0.6
7
8
9
10
11

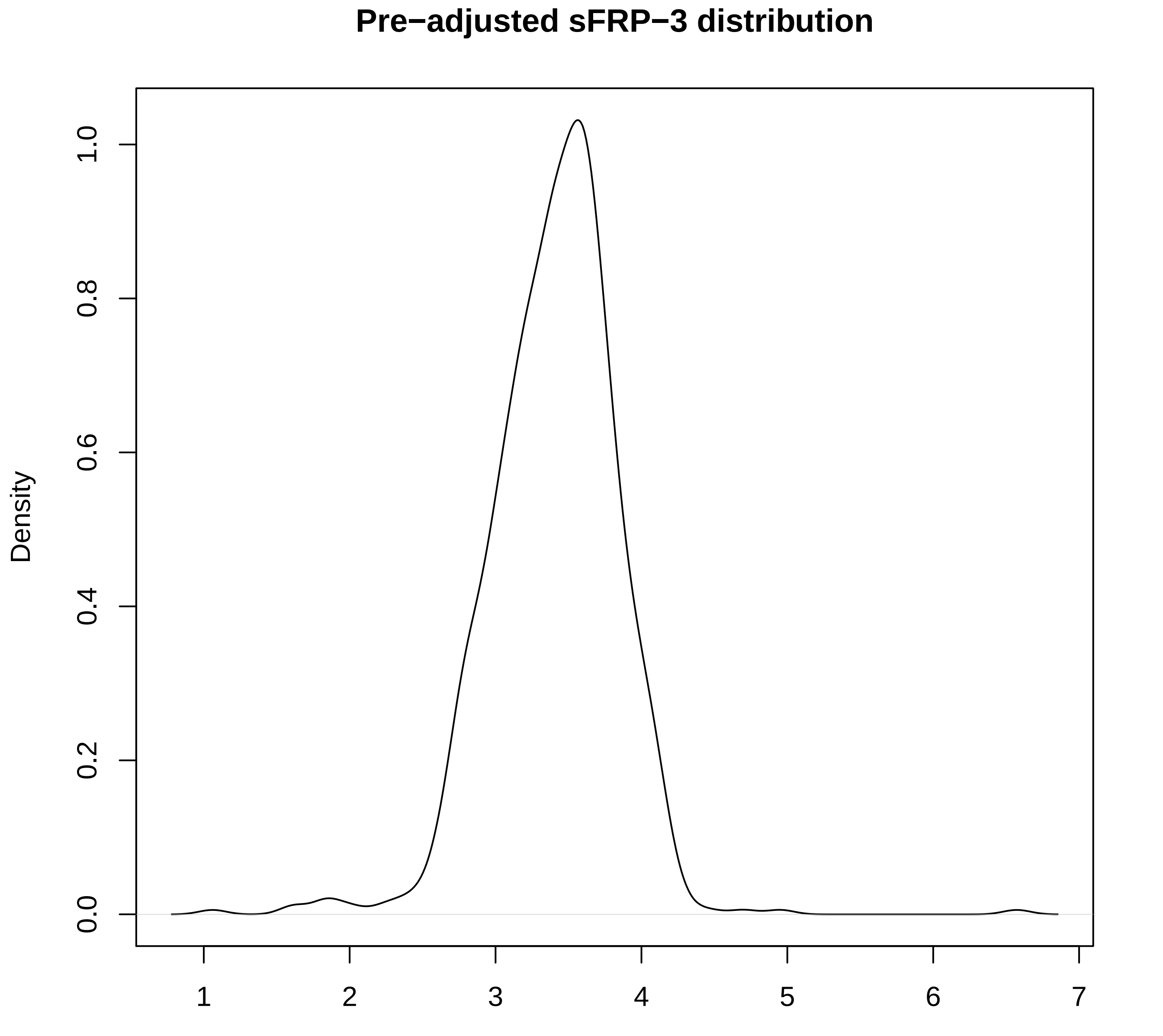

Pre−adjusted sFRP−3 distribution
Density
0.0
0.2
0.4
0.6
0.8
1.0
1
2
3
4
5
6
7

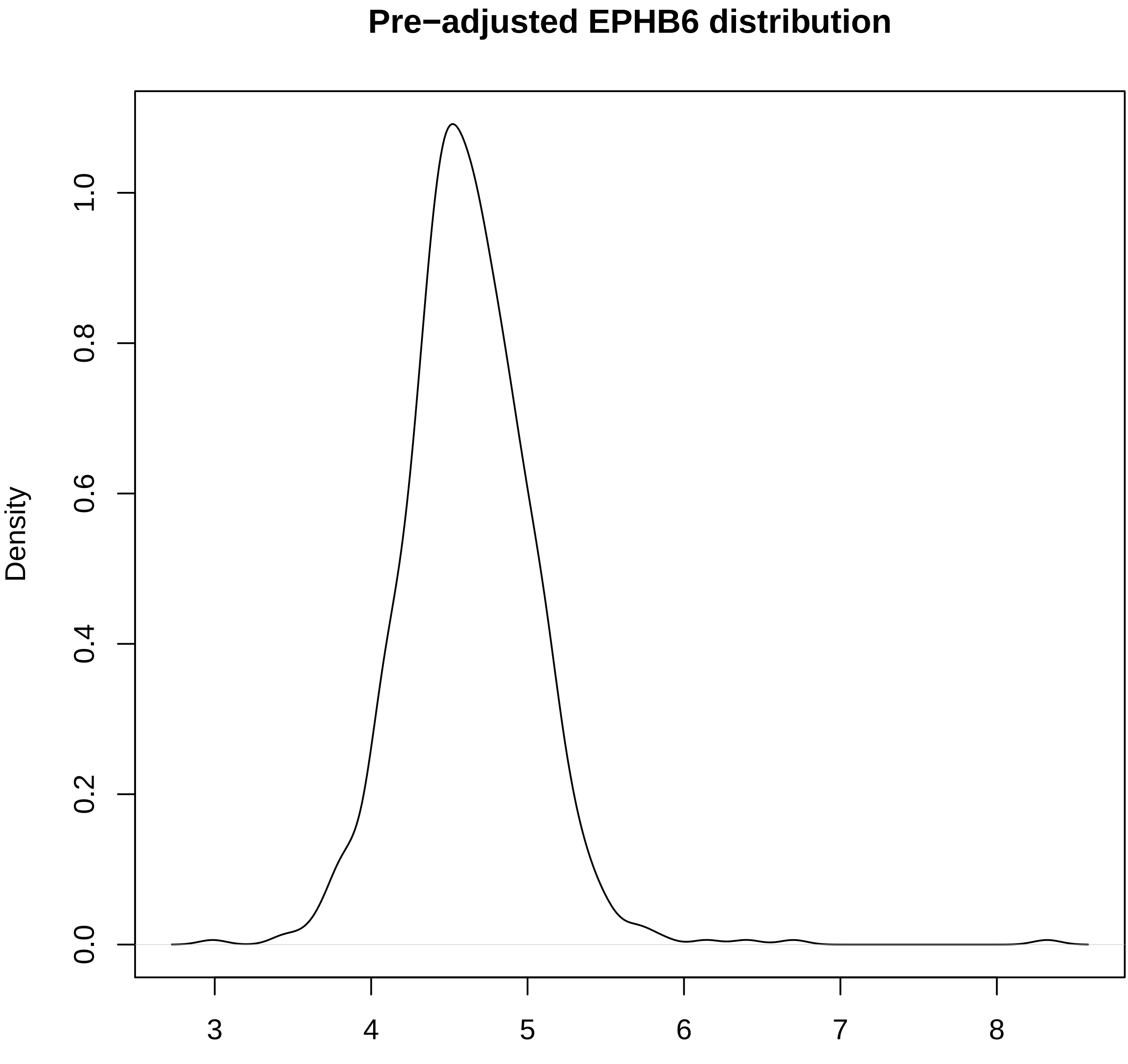
Pre−adjusted EPHB6 distribution
Density
0.0
0.2
0.4
0.6
0.8
1.0
3
4
5
6
7
8

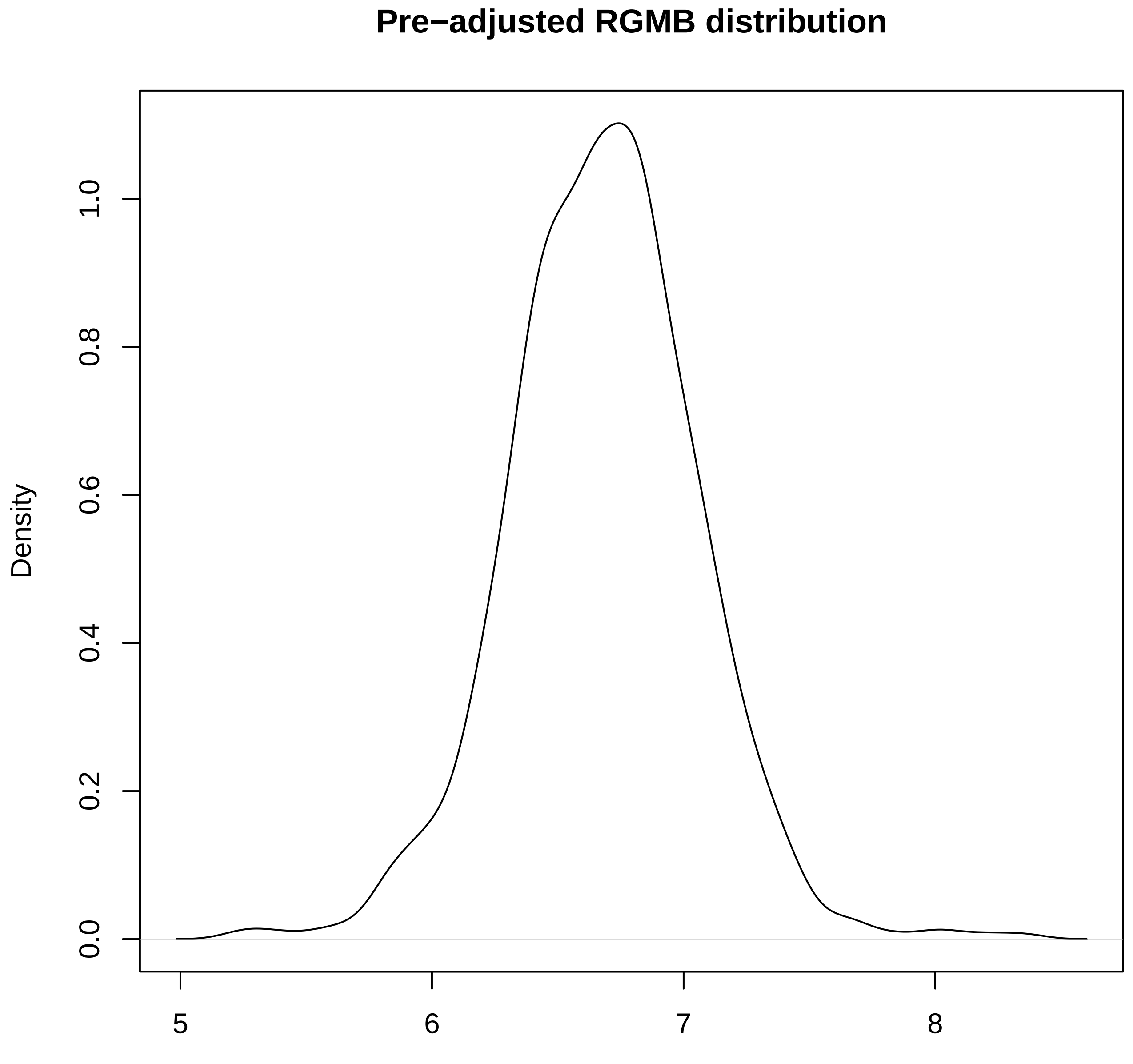
Pre−adjusted RGMB distribution
Density
0.0
0.2
0.4
0.6
0.8
1.0
5
6
7
8

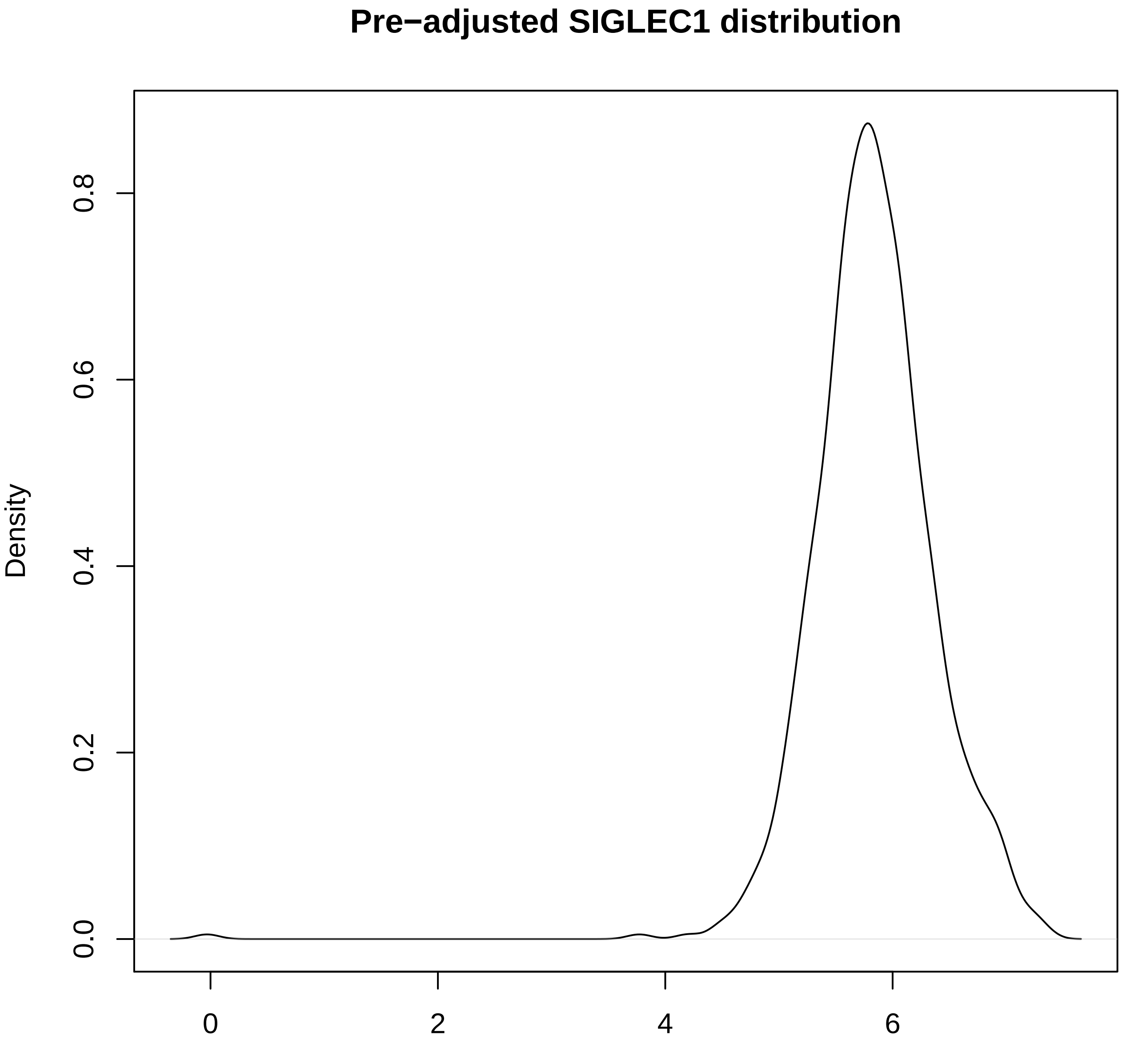

Pre−adjusted SIGLEC1 distribution
Density
0.0
0.2
0.4
0.6
0.8
0
2
4
6

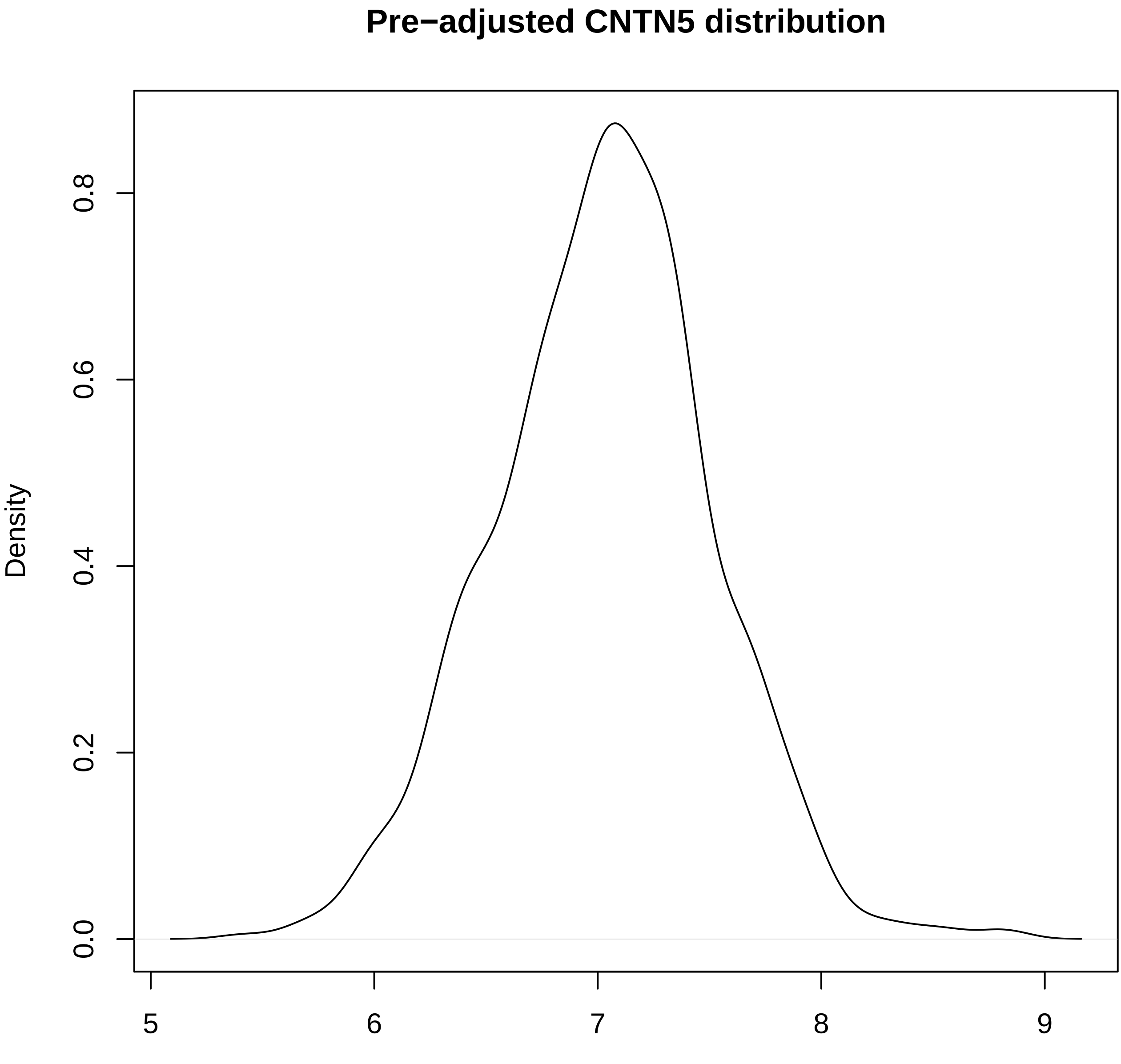
Pre−adjusted CNTN5 distribution
Density
0.0
0.2
0.4
0.6
0.8
5
6
7
8
9

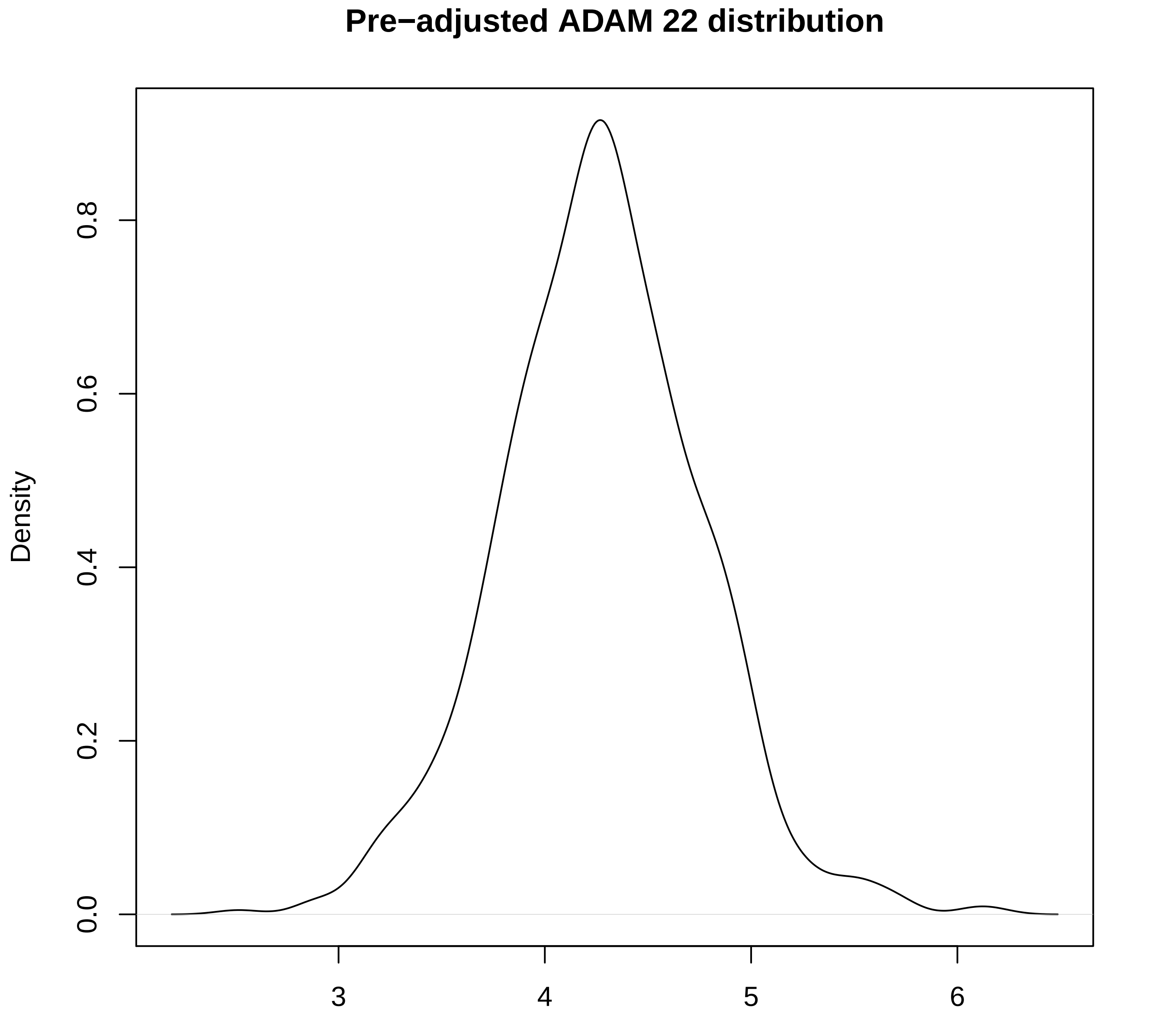
Pre−adjusted ADAM 22 distribution
Density
0.0
0.2
0.4
0.6
0.8
3
4
5
6

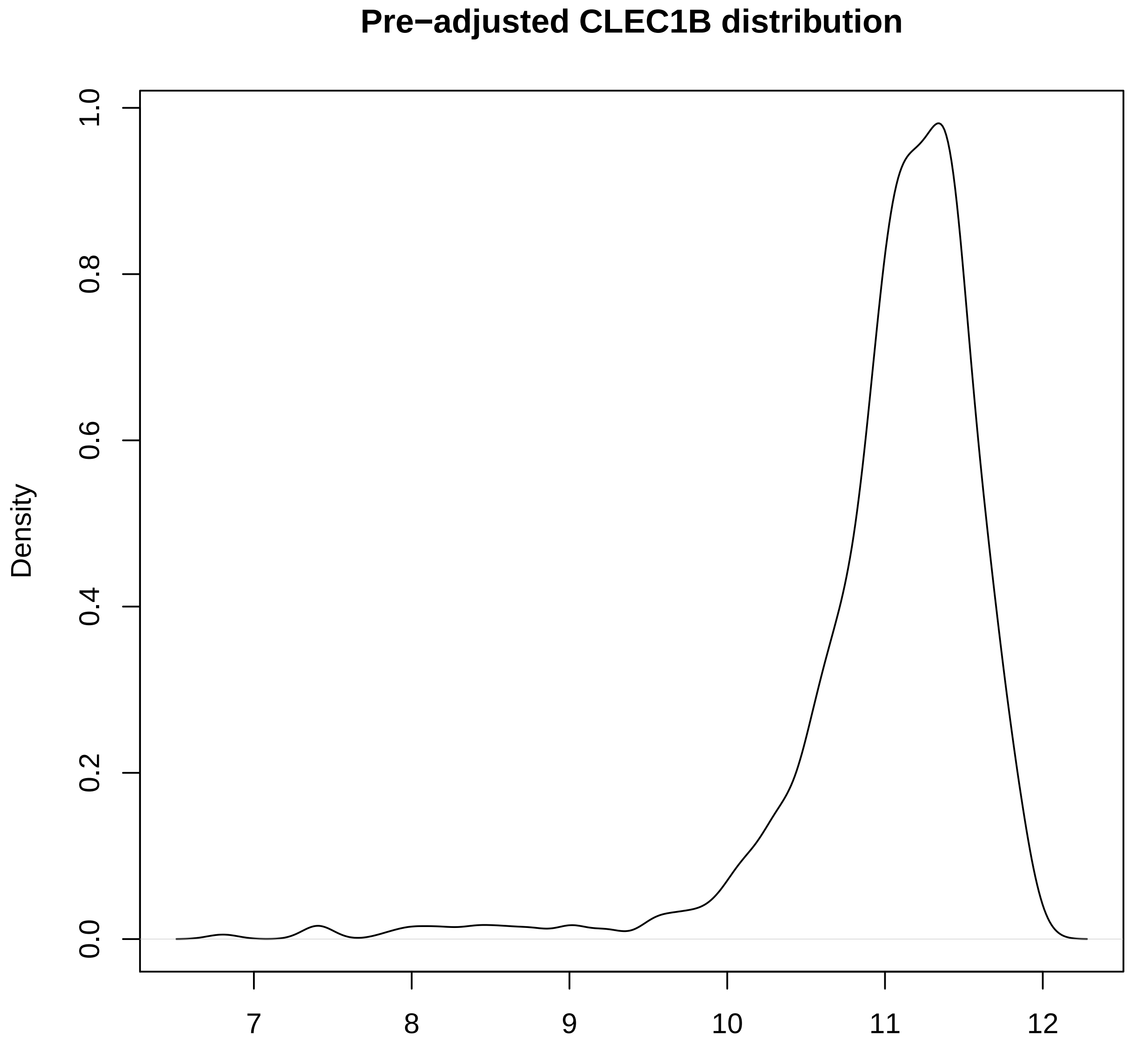
Pre−adjusted CLEC1B distribution
Density
0.0
0.2
0.4
0.6
0.8
1.0
7
8
9
10
11
12

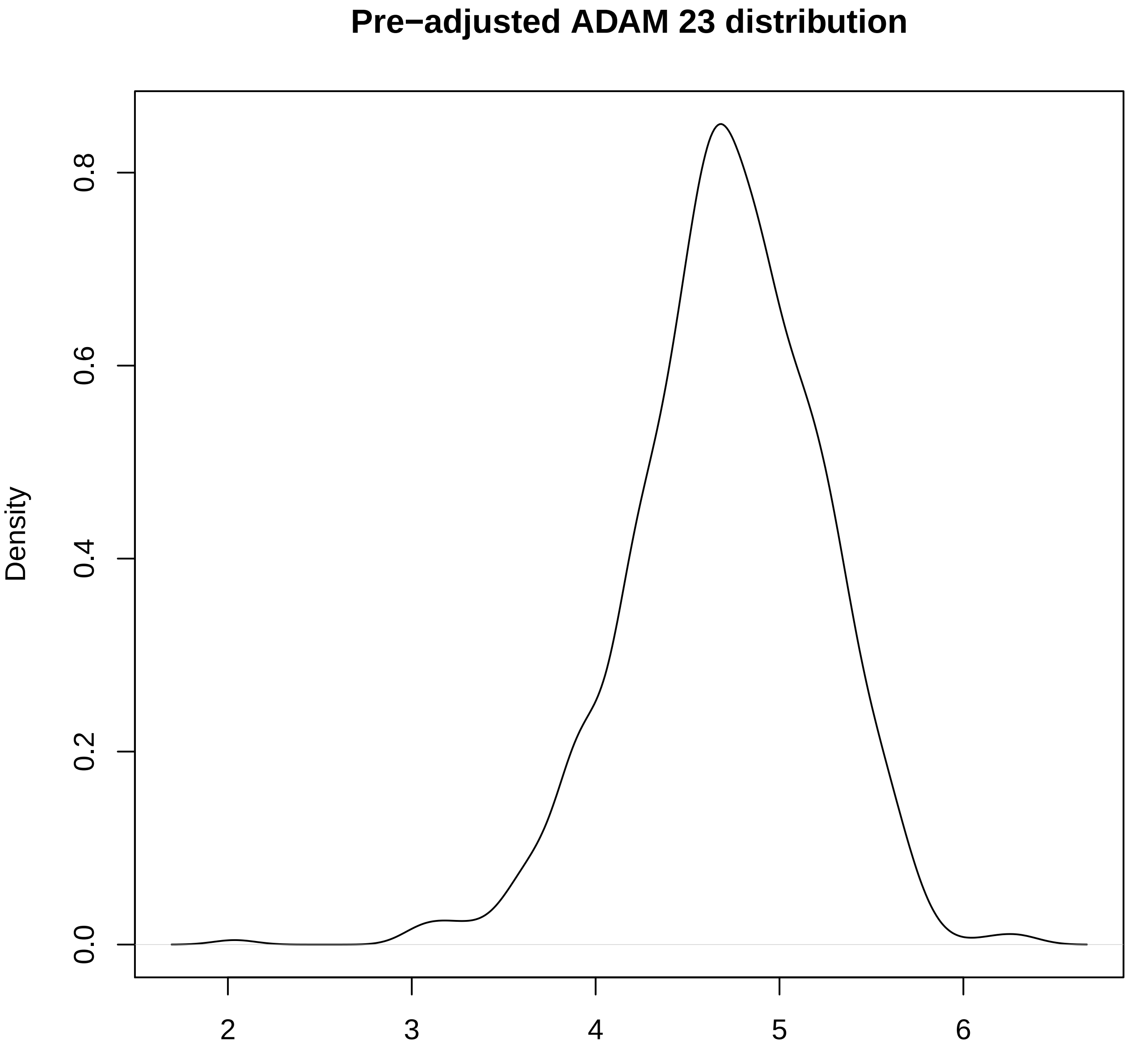
Pre−adjusted ADAM 23 distribution
Density
0.0
0.2
0.4
0.6
0.8
2
3
4
5
6

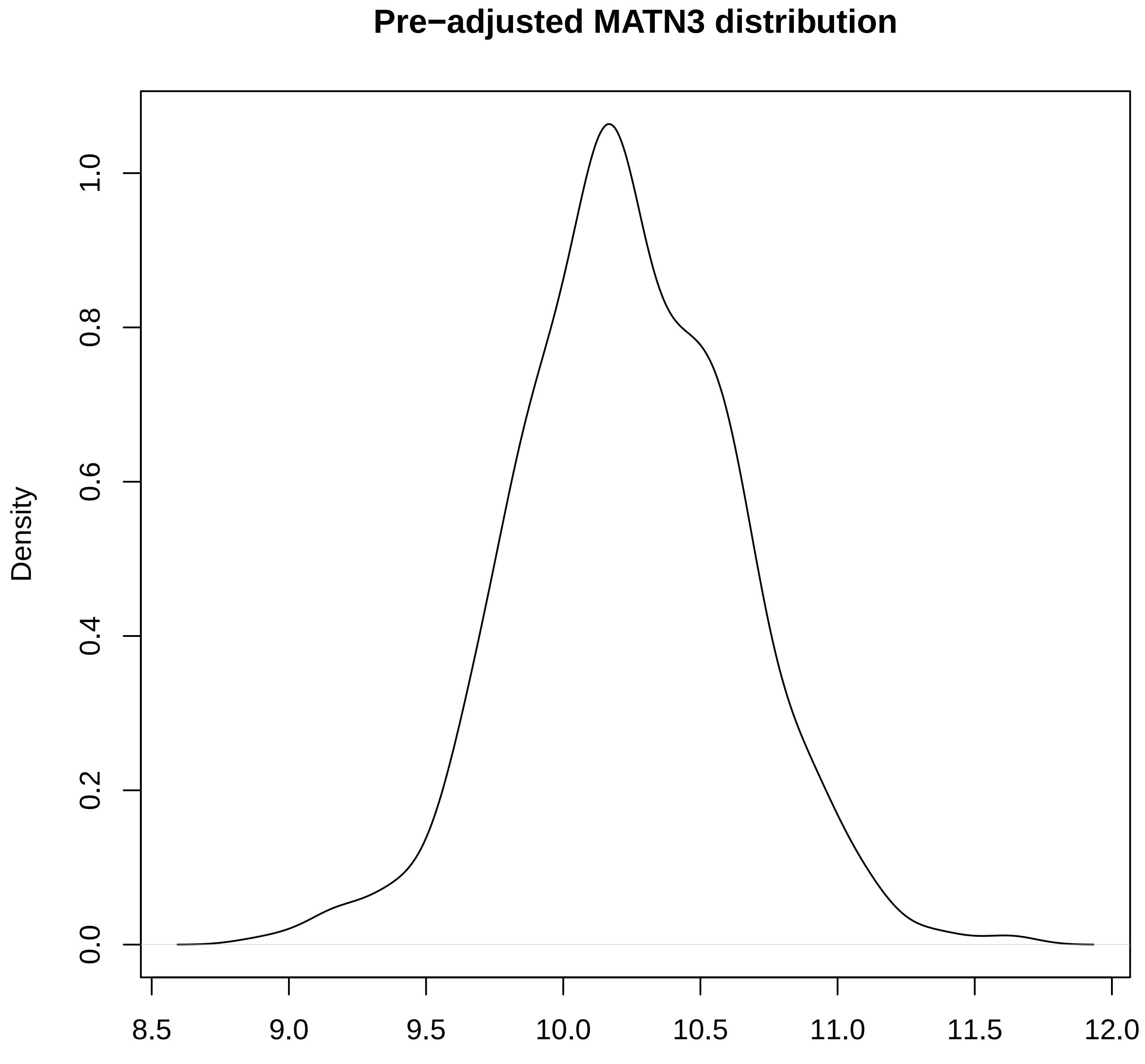
Pre−adjusted MATN3 distribution
Density
0.0
0.2
0.4
0.6
0.8
1.0
8.5
9.0
9.5
10.0
10.5
11.0
11.5
12.0

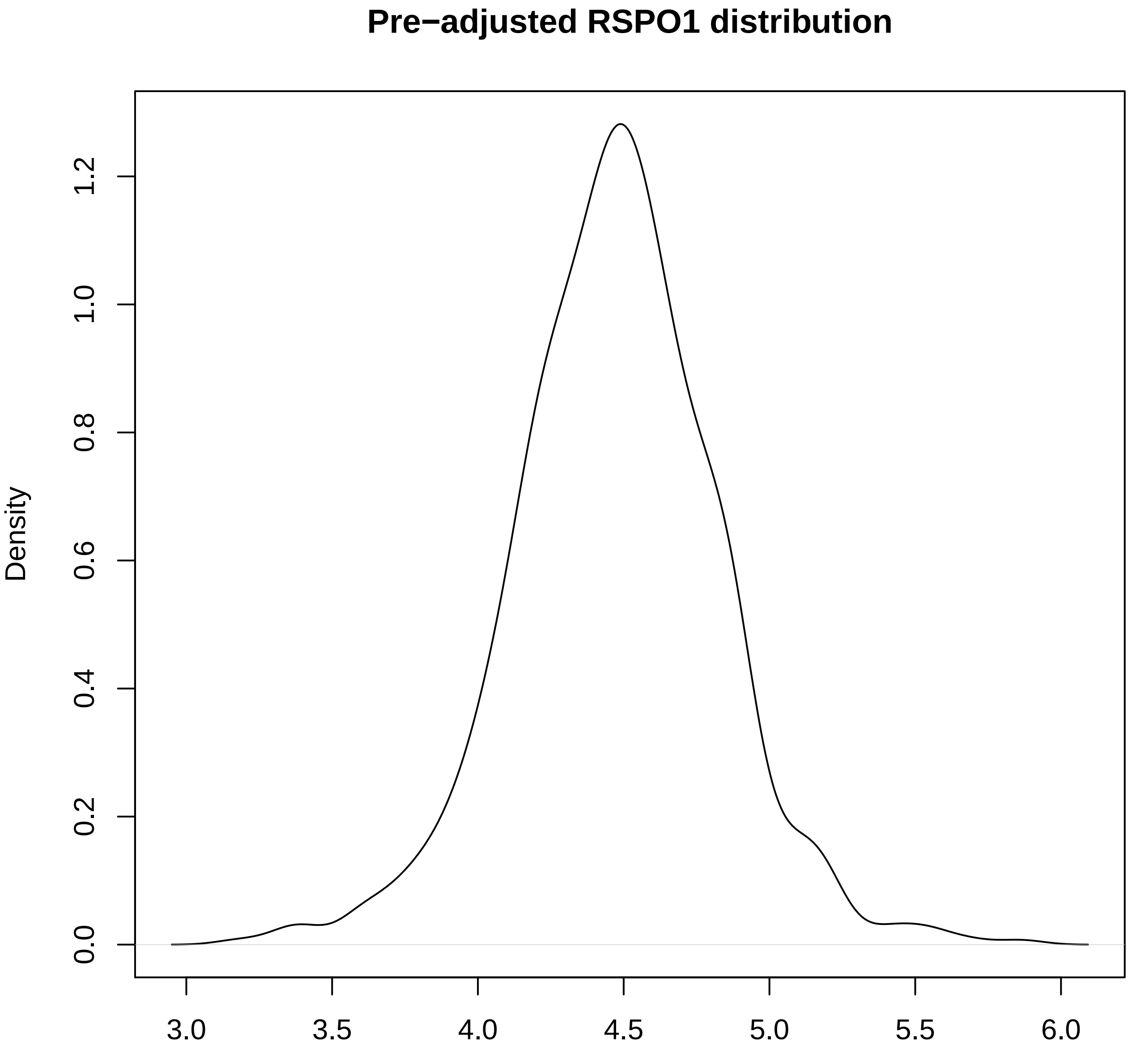
Pre−adjusted RSPO1 distribution
Density
0.0
0.2
0.4
0.6
0.8
1.0
1.2
3.0
3.5
4.0
4.5
5.0
5.5
6.0

Pre−adjusted HAGH distribution

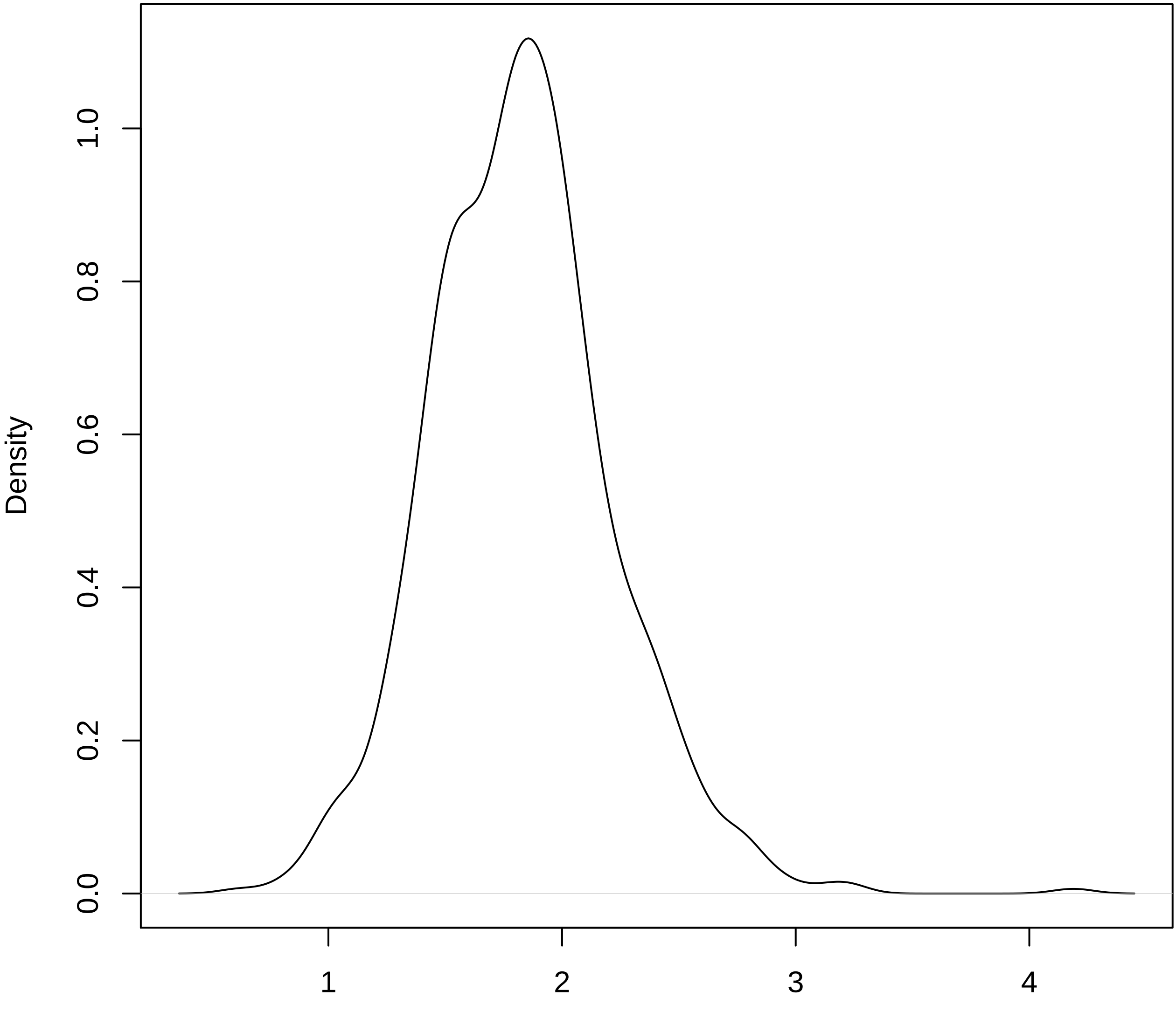

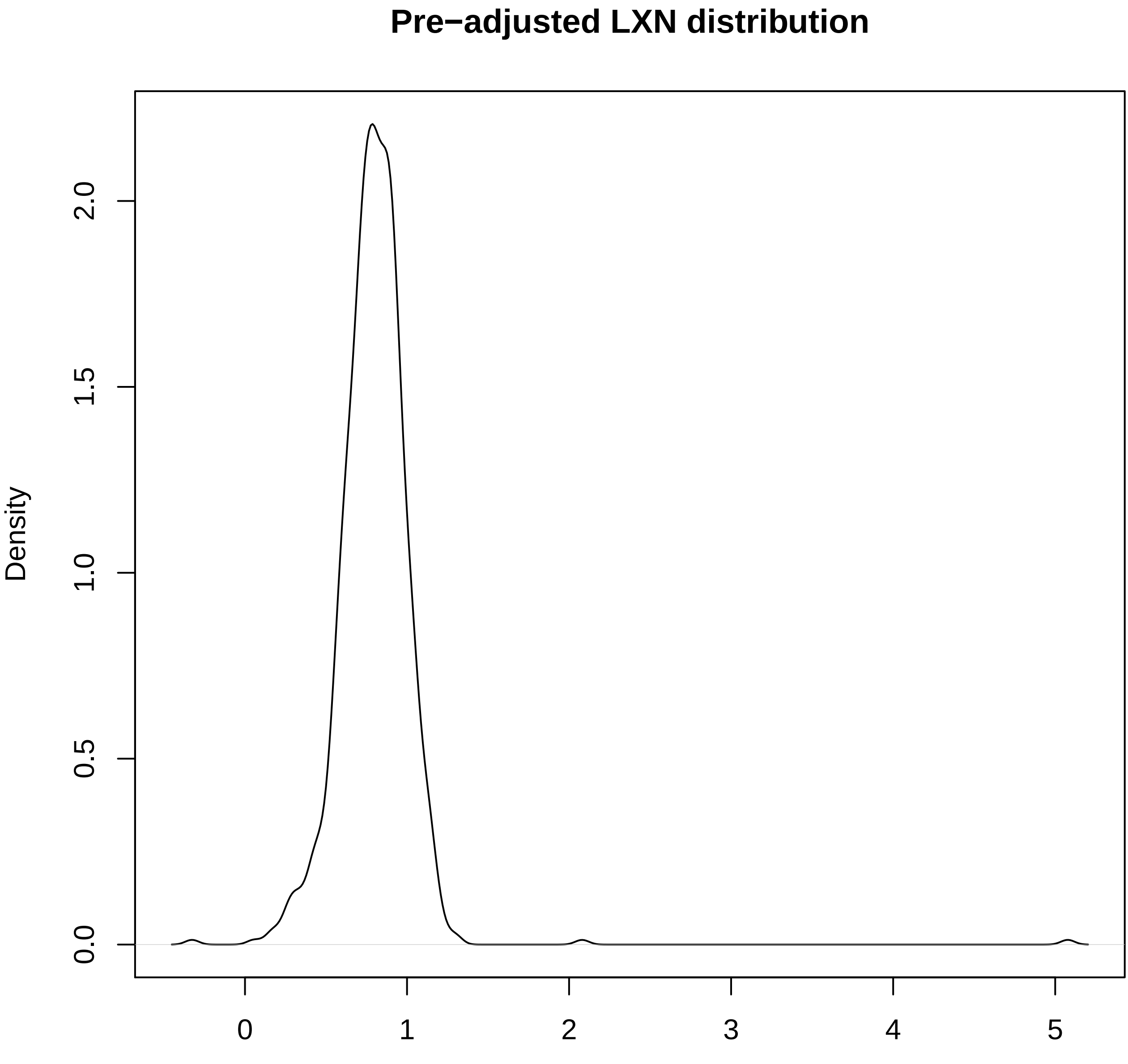
Pre−adjusted LXN distribution
Density
0.0
0.5
1.0
1.5
2.0
0
1
2
3
4
5

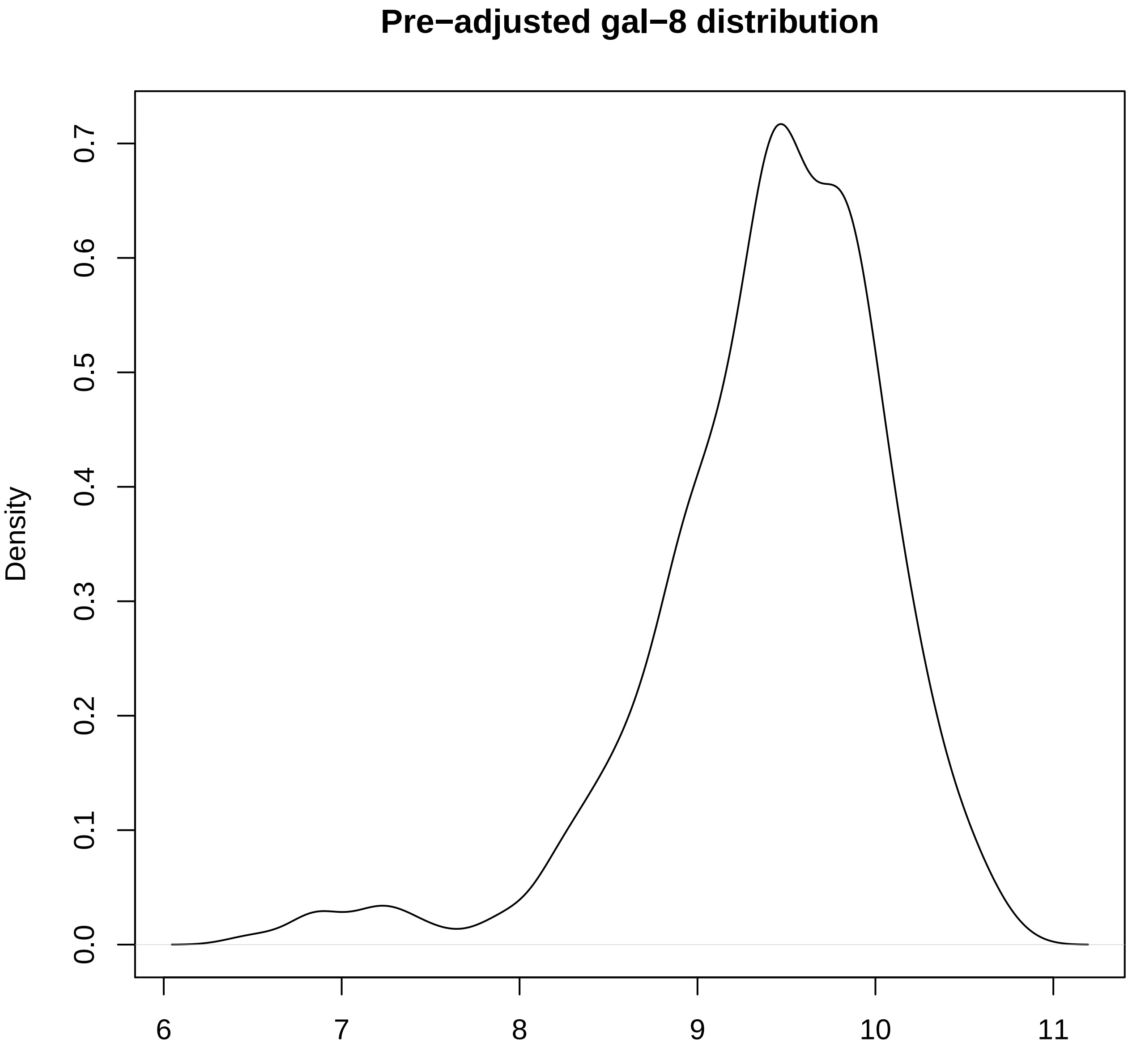
Pre−adjusted gal−8 distribution
Density
0.0
0.1
0.2
0.3
0.4
0.5
0.6
0.7
6
7
8
9
10
11

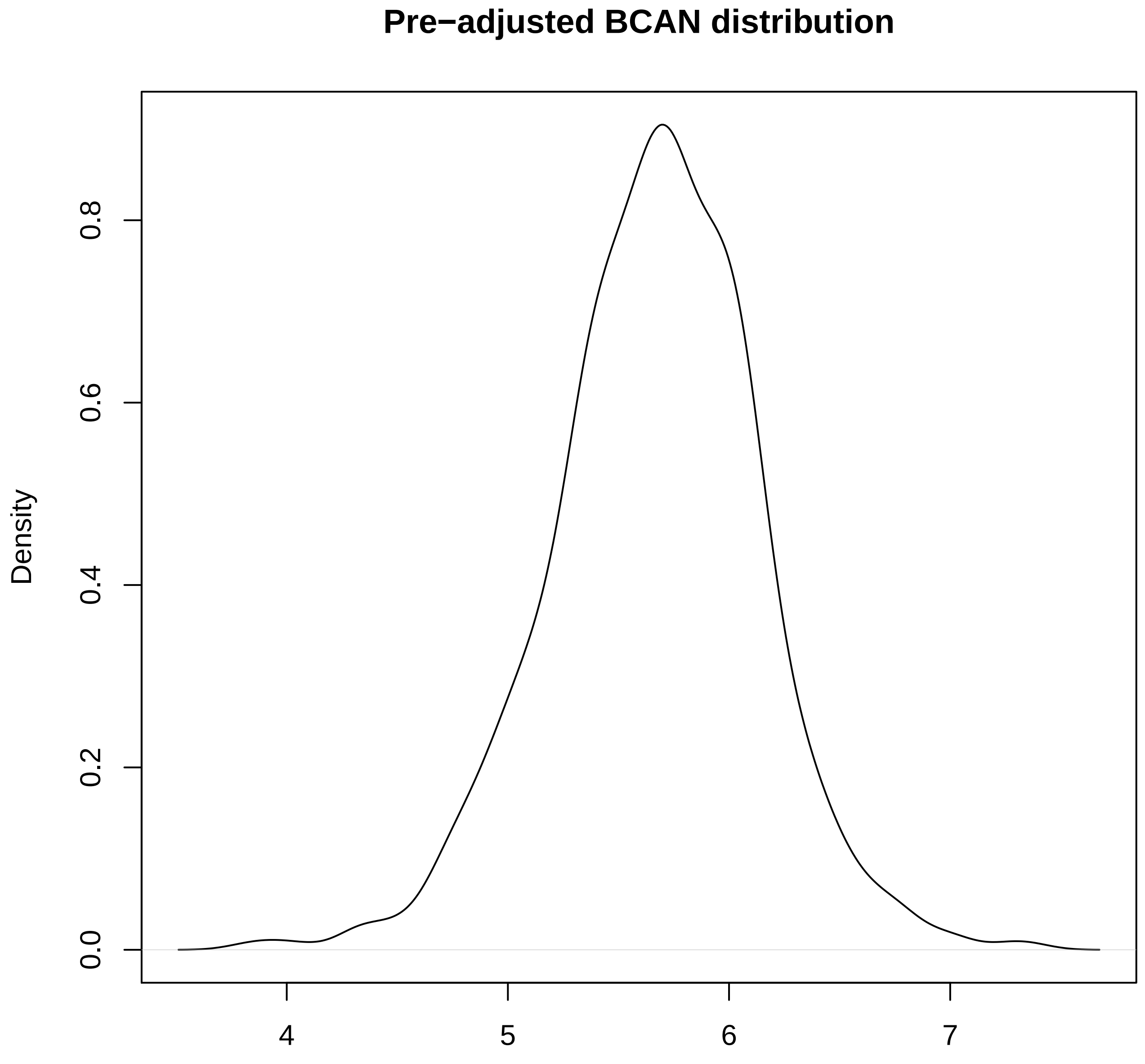
Pre−adjusted BCAN distribution
Density
0.0
0.2
0.4
0.6
0.8
4
5
6
7

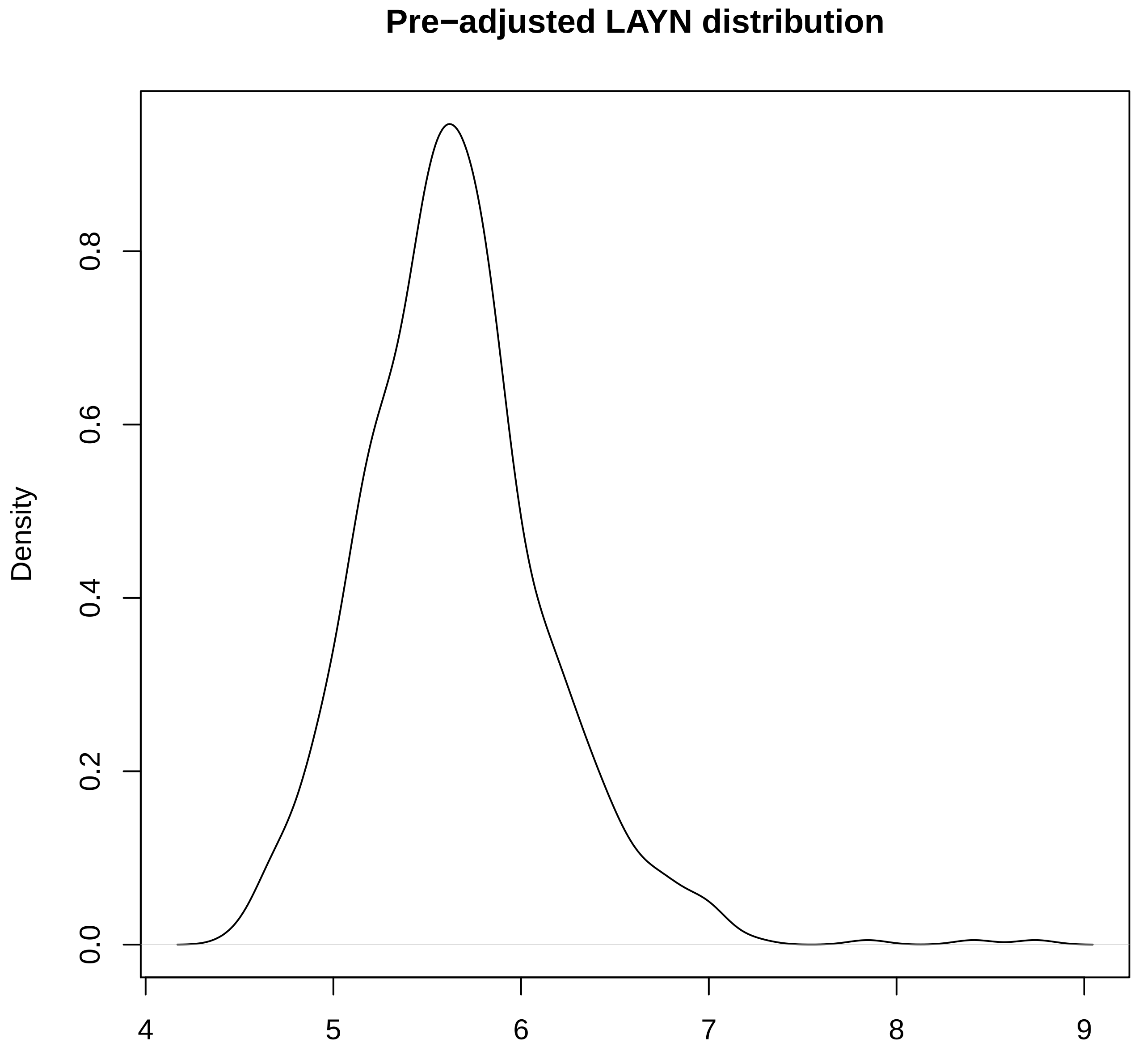
Pre−adjusted LAYN distribution
Density
0.0
0.2
0.4
0.6
0.8
4
5
6
7
8
9

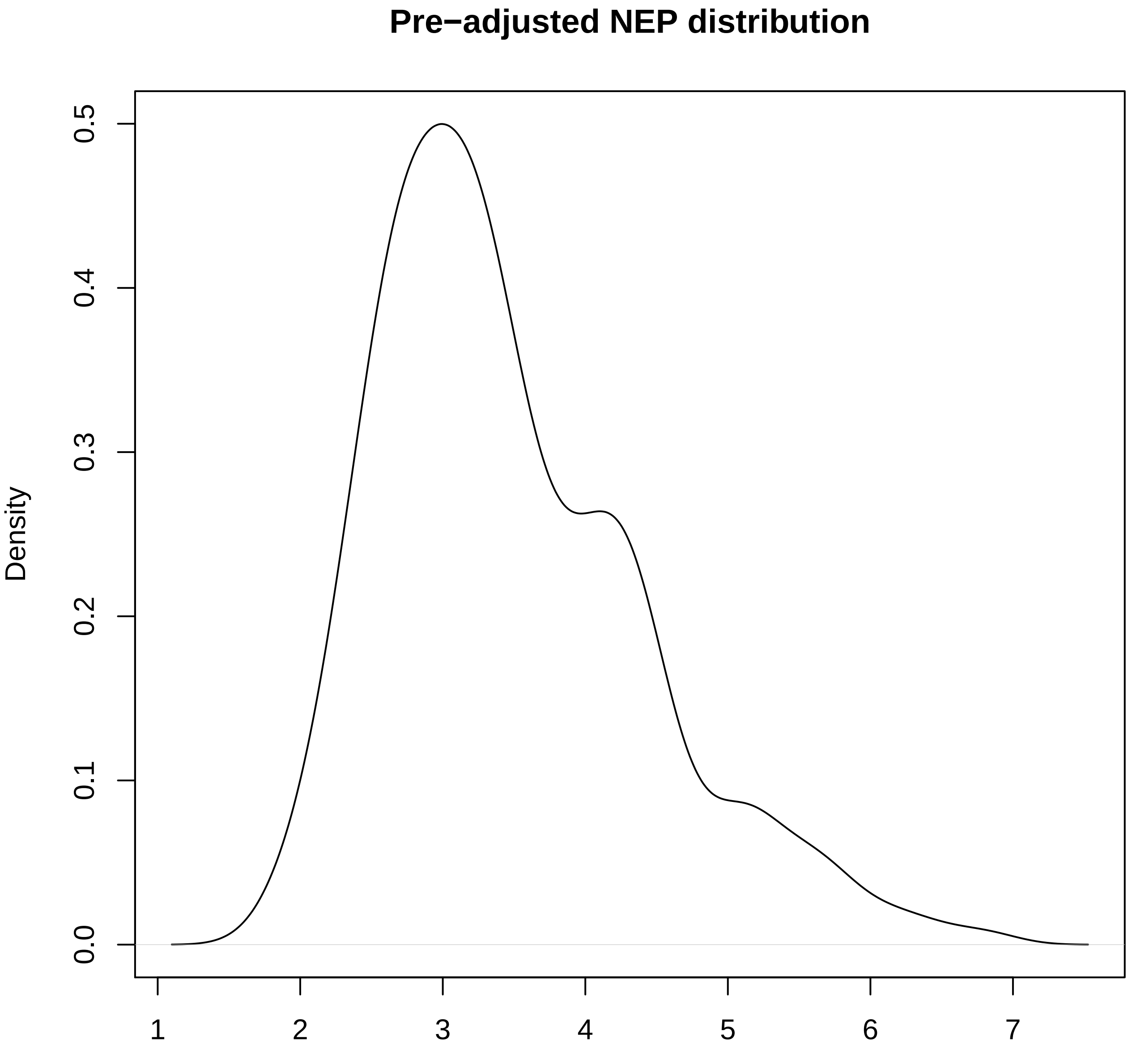
Pre−adjusted NEP distribution
Density
0.0
0.1
0.2
0.3
0.4
0.5
1
2
3
4
5
6
7

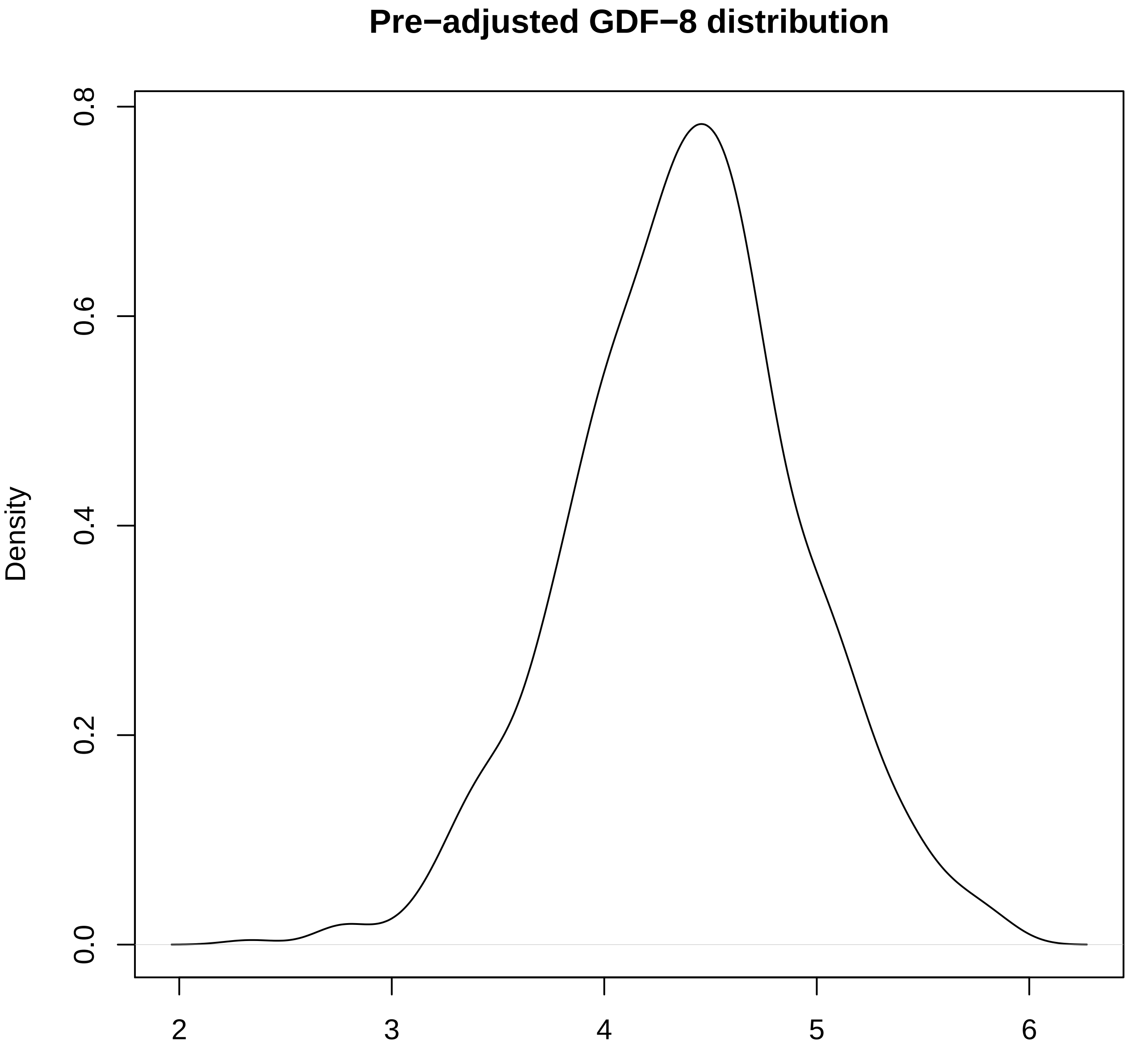

Pre−adjusted GDF−8 distribution
Density
0.0
0.2
0.4
0.6
0.8
2
3
4
5
6

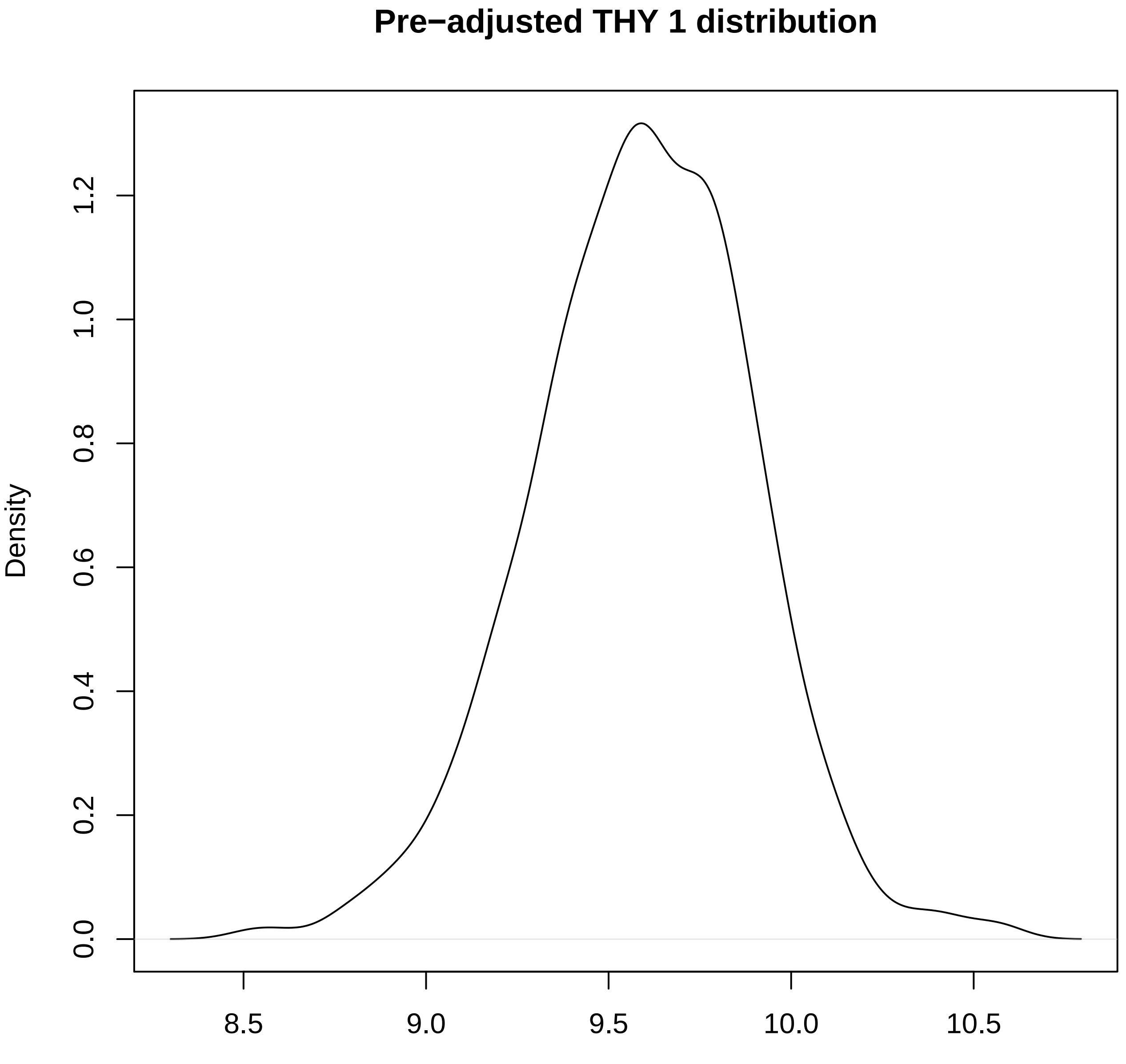

Pre−adjusted THY 1 distribution
Density
0.0
0.2
0.4
0.6
0.8
1.0
1.2
8.5
9.0
9.5
10.0
10.5

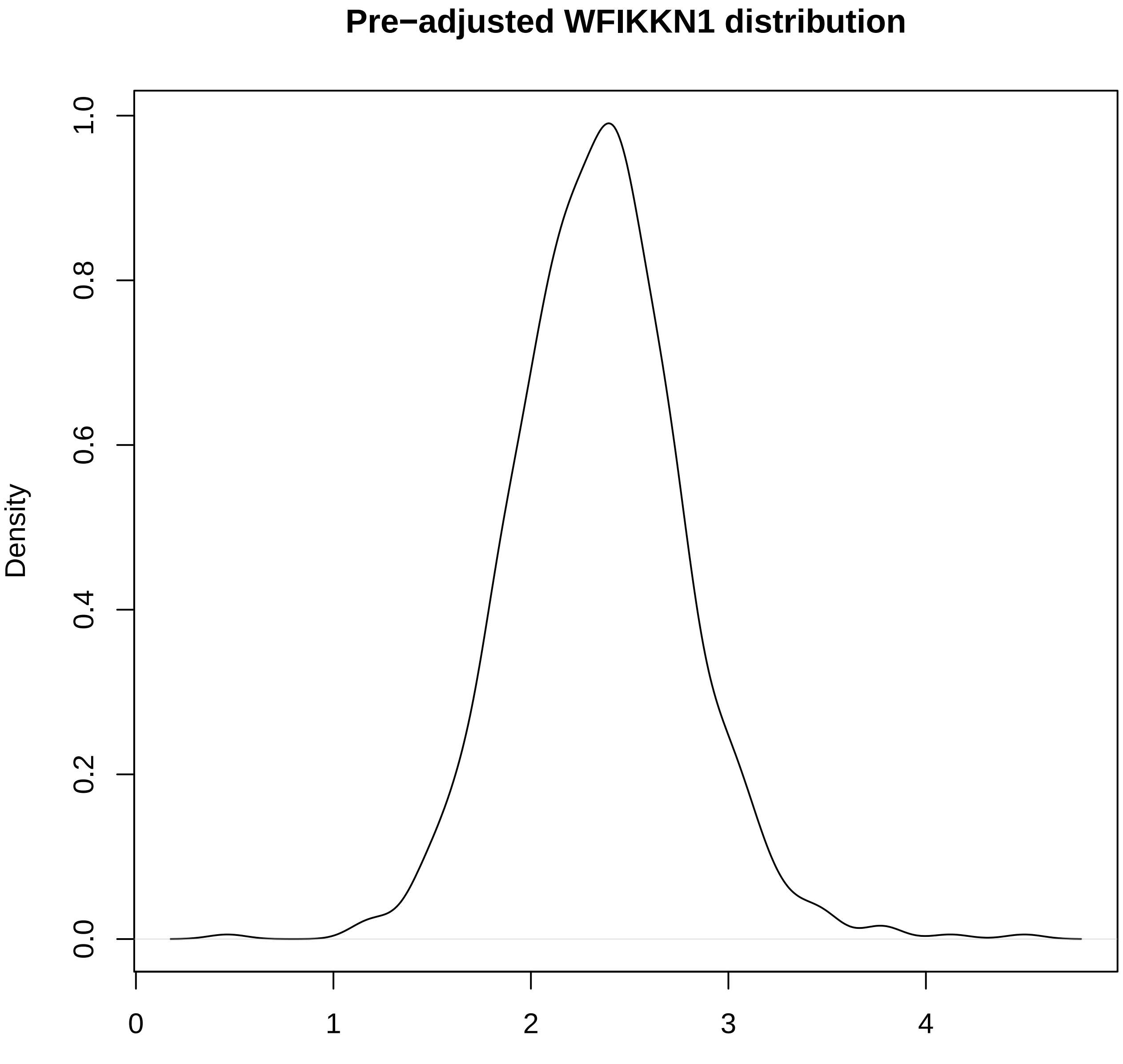
Pre−adjusted WFIKKN1 distribution
Density
0.0
0.2
0.4
0.6
0.8
1.0
0
1
2
3
4

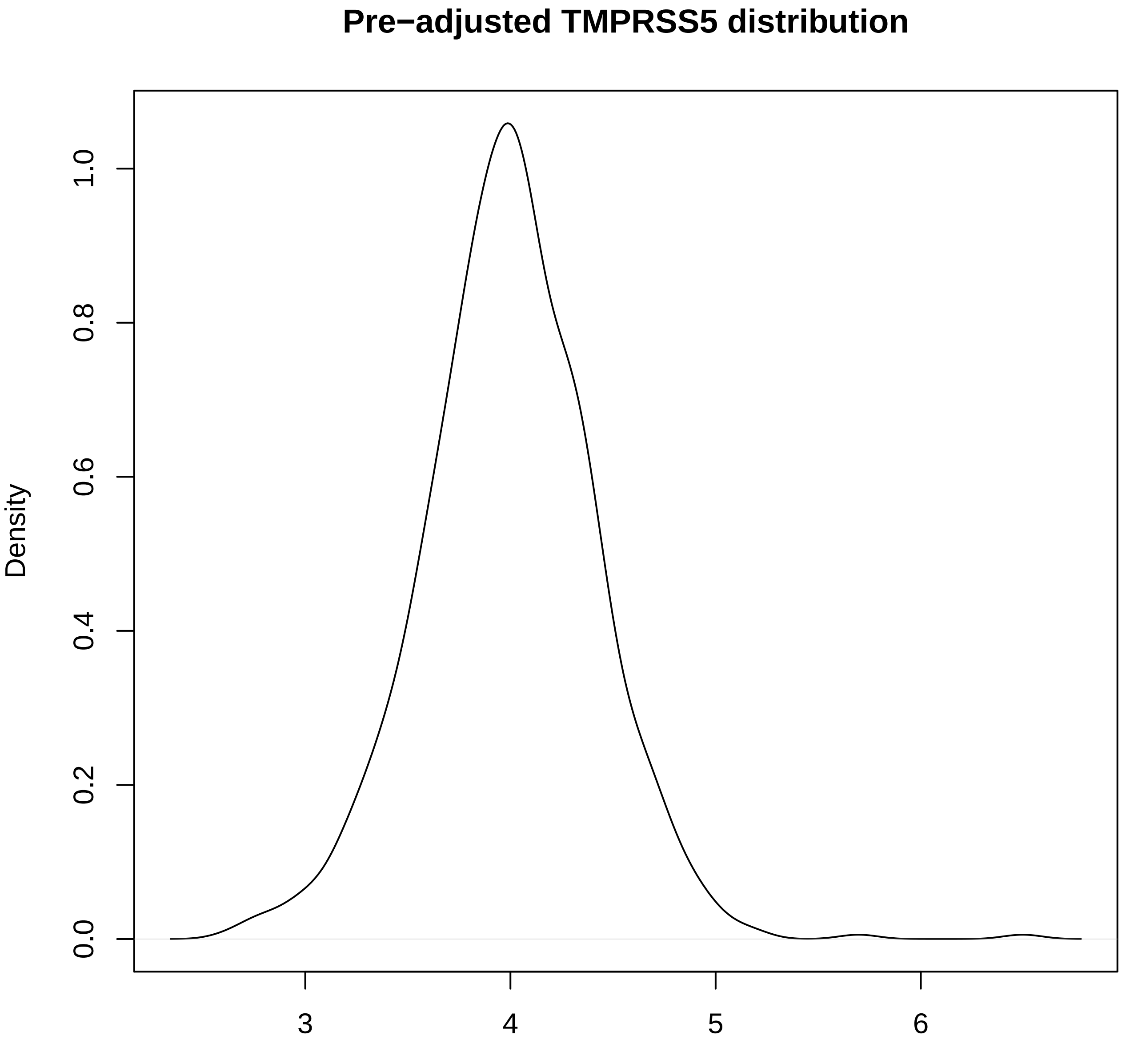
Pre−adjusted TMPRSS5 distribution
Density
0.0
0.2
0.4
0.6
0.8
1.0
3
4
5
6

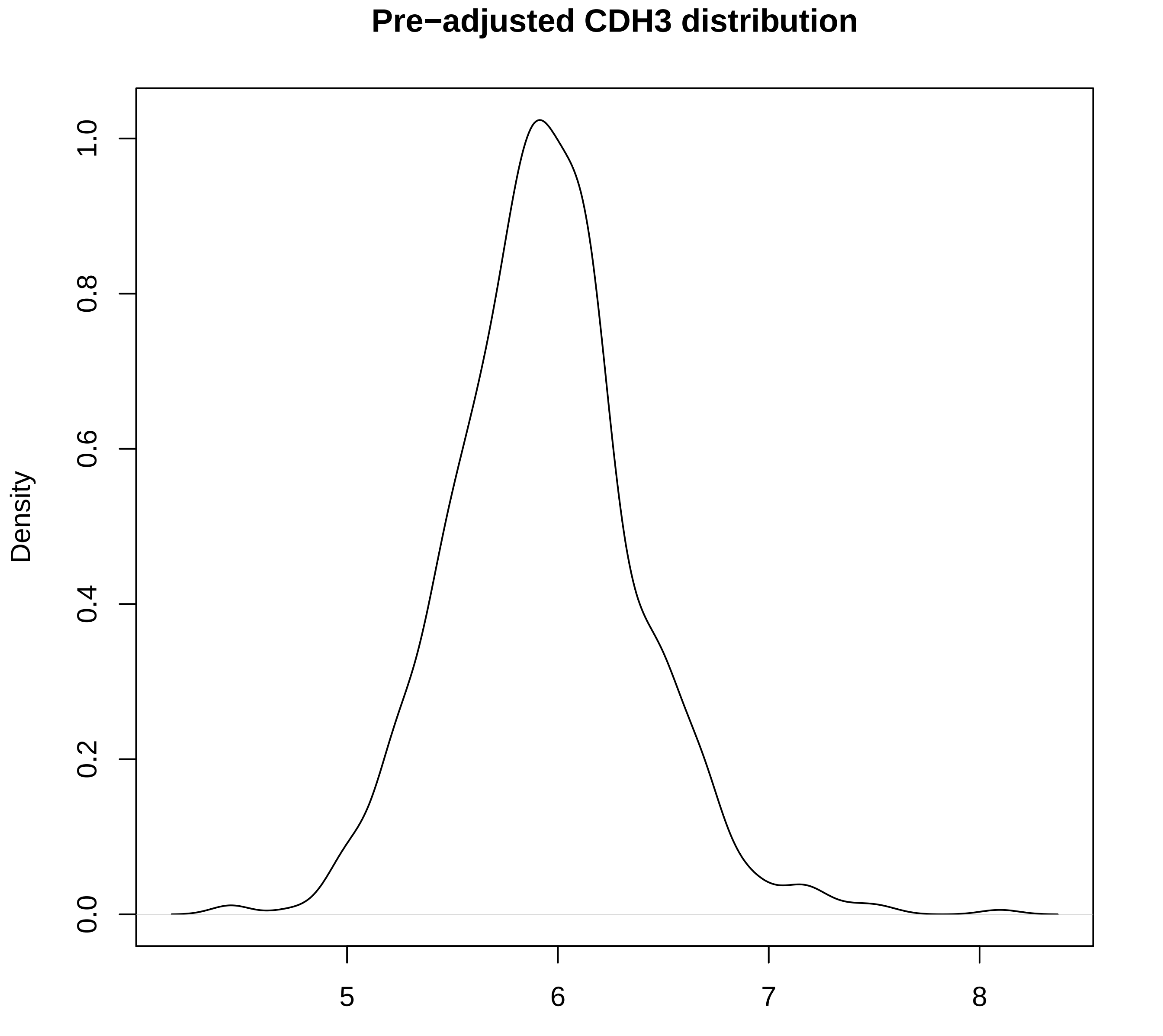
Pre−adjusted CDH3 distribution
Density
0.0
0.2
0.4
0.6
0.8
1.0
5
6
7
8

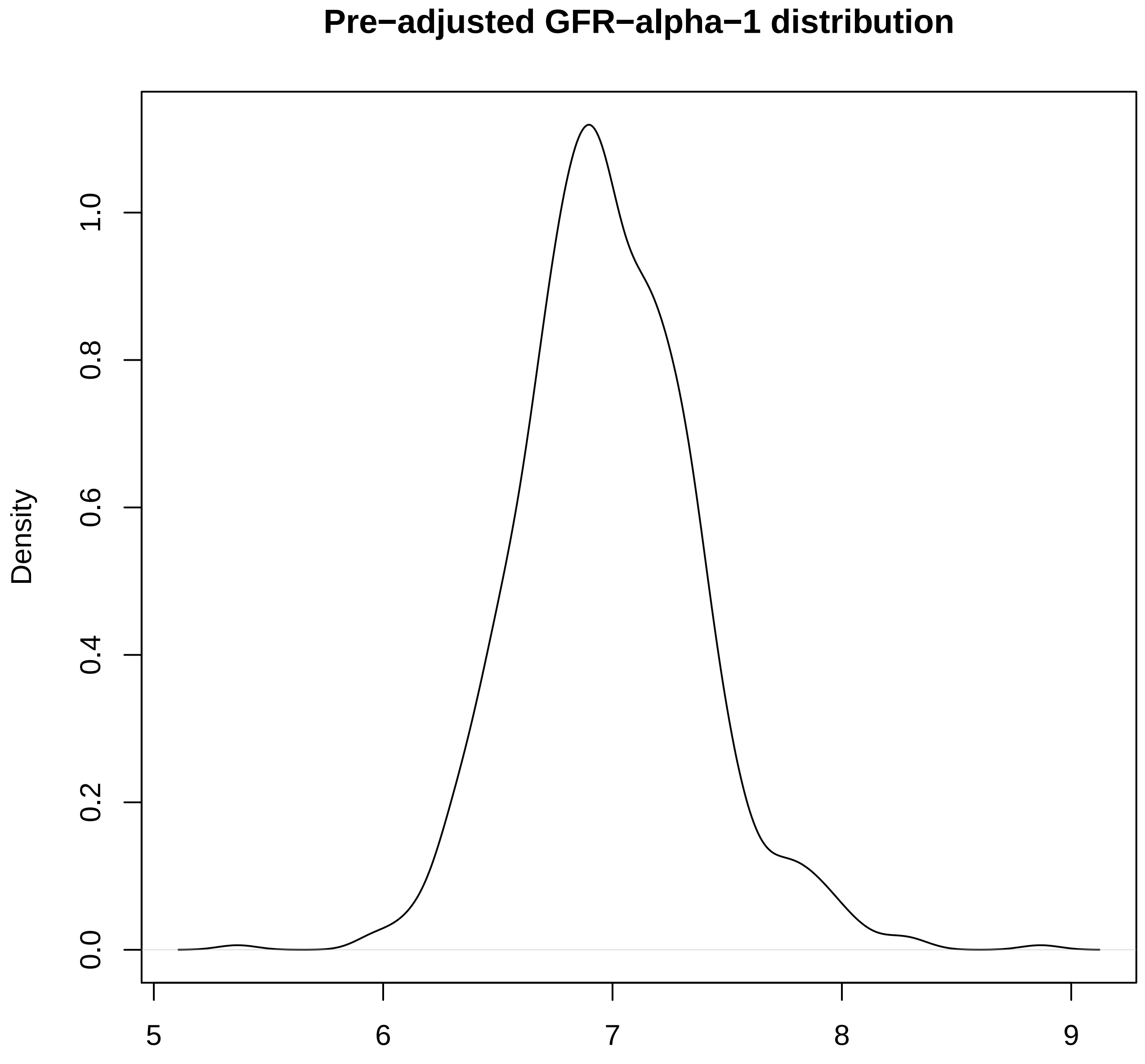
Pre−adjusted GFR−alpha−1 distribution
Density
0.0
0.2
0.4
0.6
0.8
1.0
5
6
7
8
9

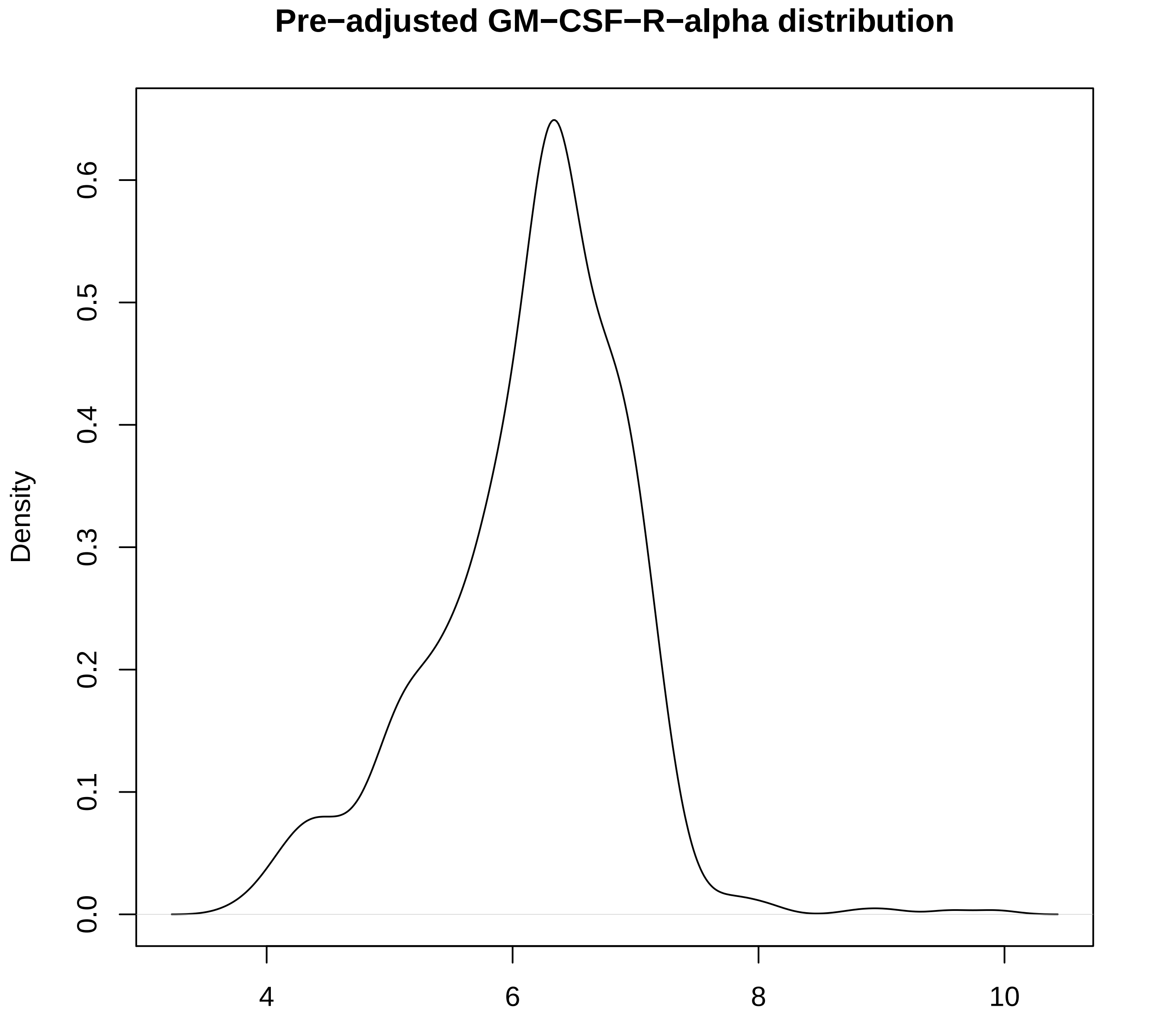
Pre−adjusted GM−CSF−R−alpha distribution
Density
0.0
0.1
0.2
0.3
0.4
0.5
0.6
4
6
8
10

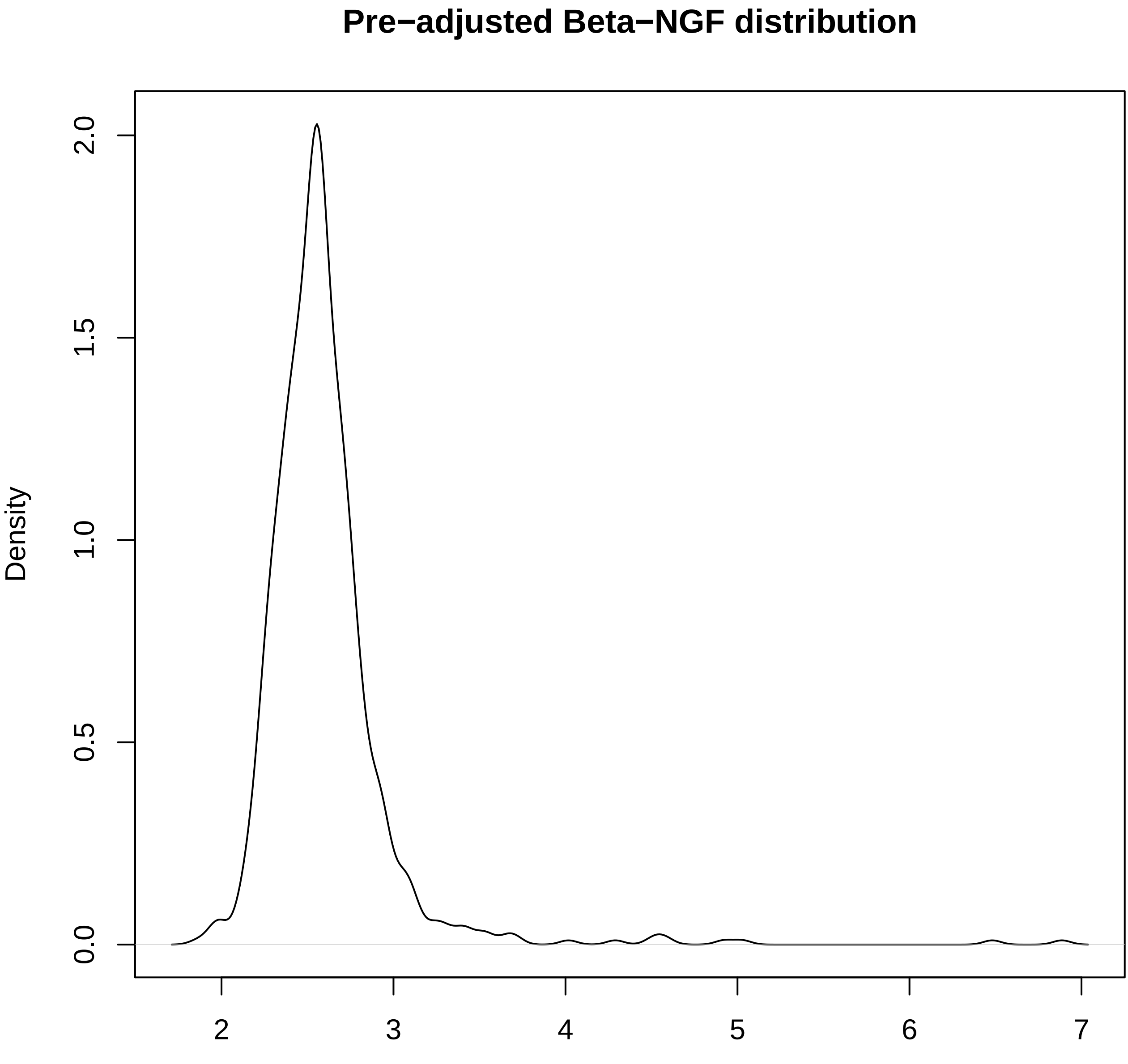
Pre−adjusted Beta−NGF distribution
Density
0.0
0.5
1.0
1.5
2.0
2
3
4
5
6
7

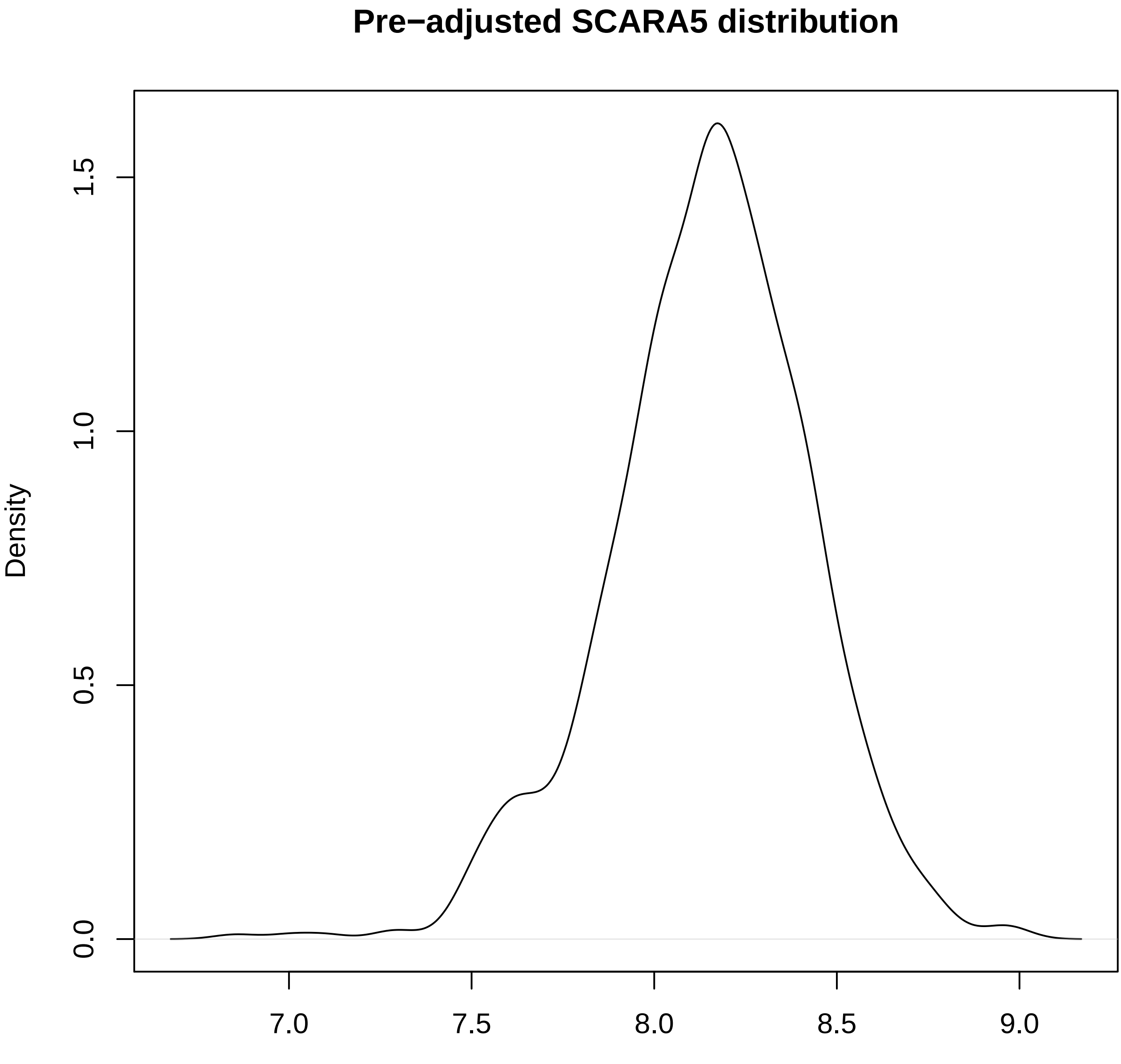

Pre−adjusted SCARA5 distribution
Density
0.0
0.5
1.0
1.5
7.0
7.5
8.0
8.5
9.0

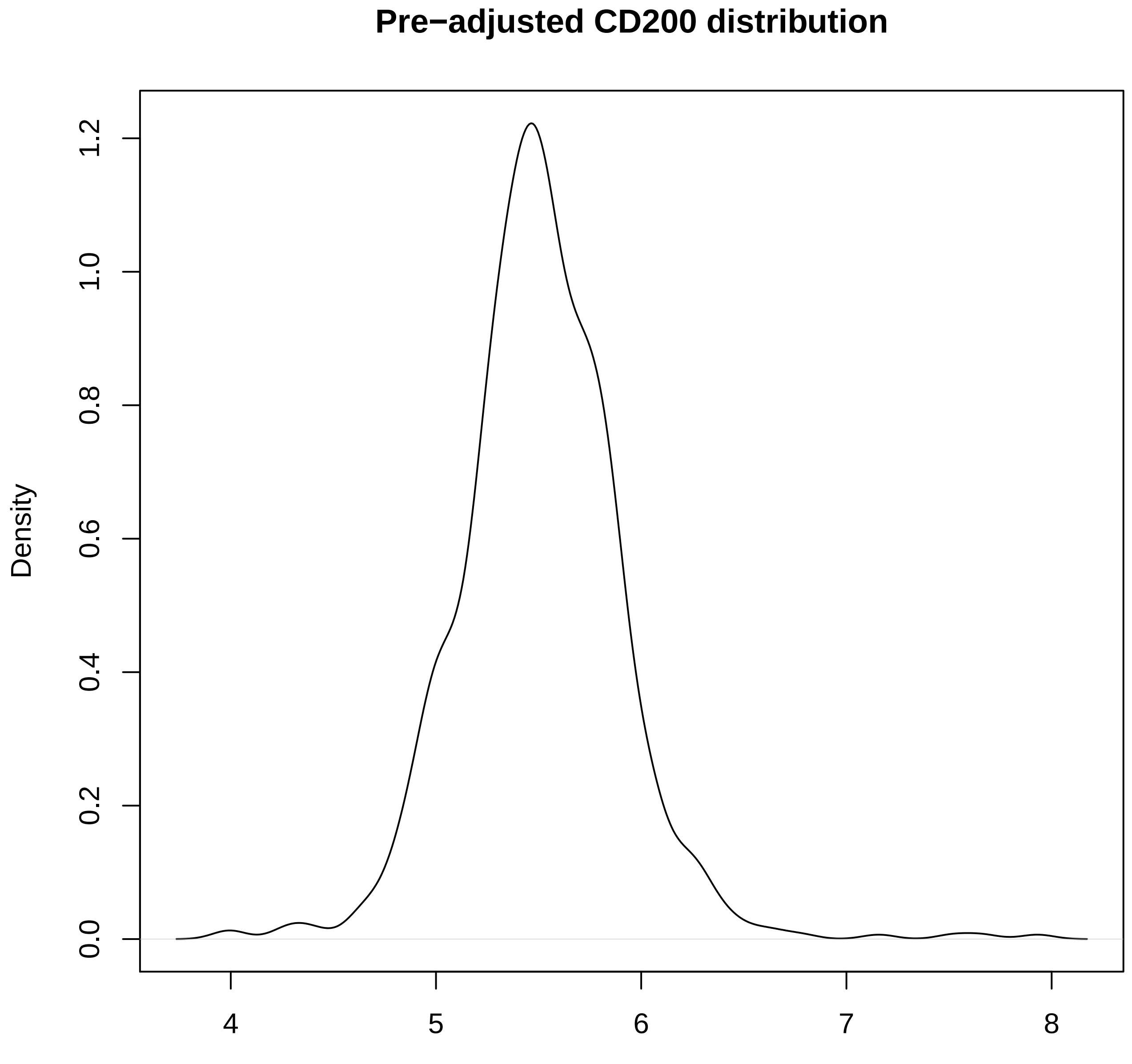
Pre−adjusted CD200 distribution
Density
0.0
0.2
0.4
0.6
0.8
1.0
1.2
4
5
6
7
8

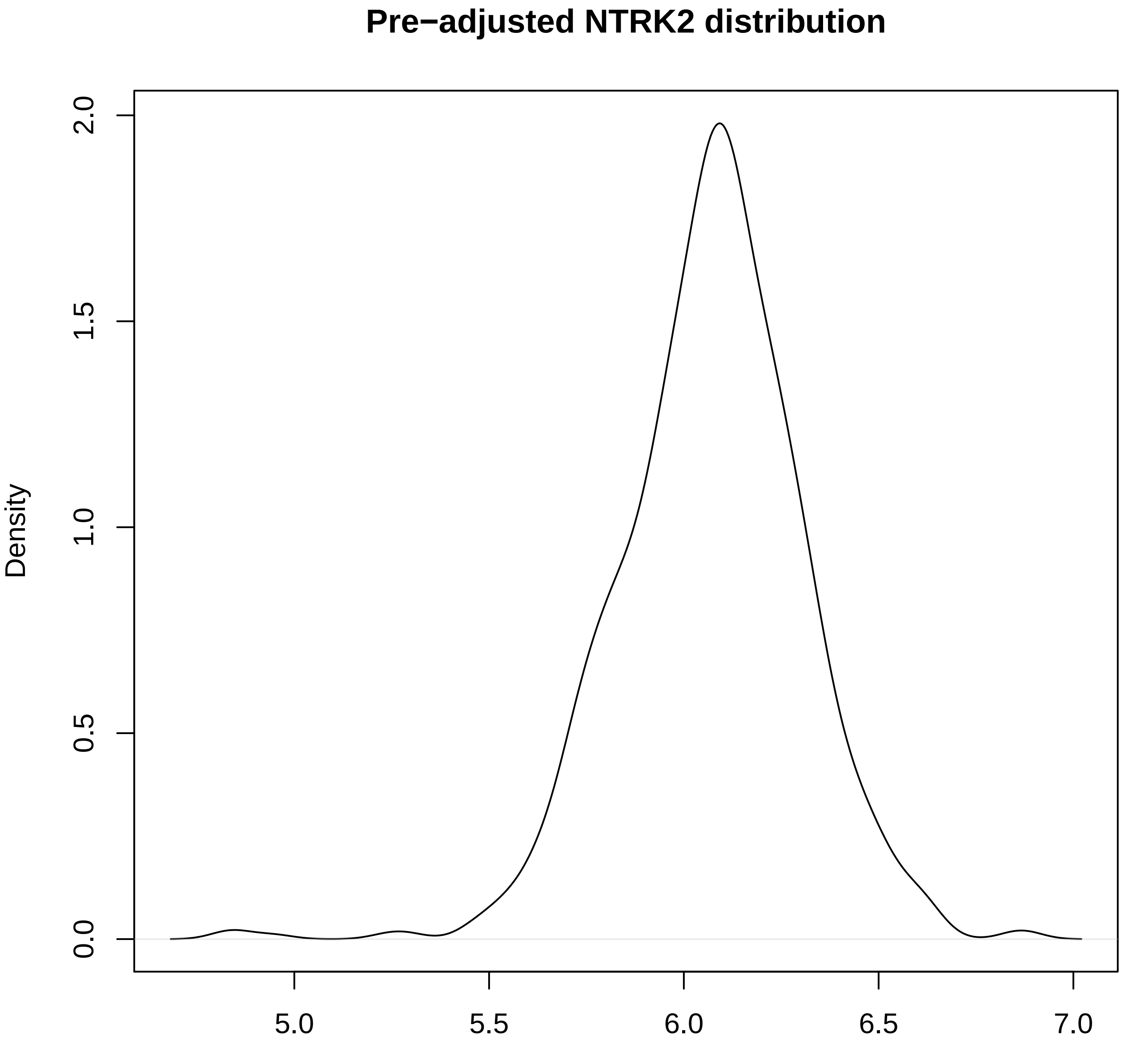

Pre−adjusted NTRK2 distribution
Density
0.0
0.5
1.0
1.5
2.0
5.0
5.5
6.0
6.5
7.0

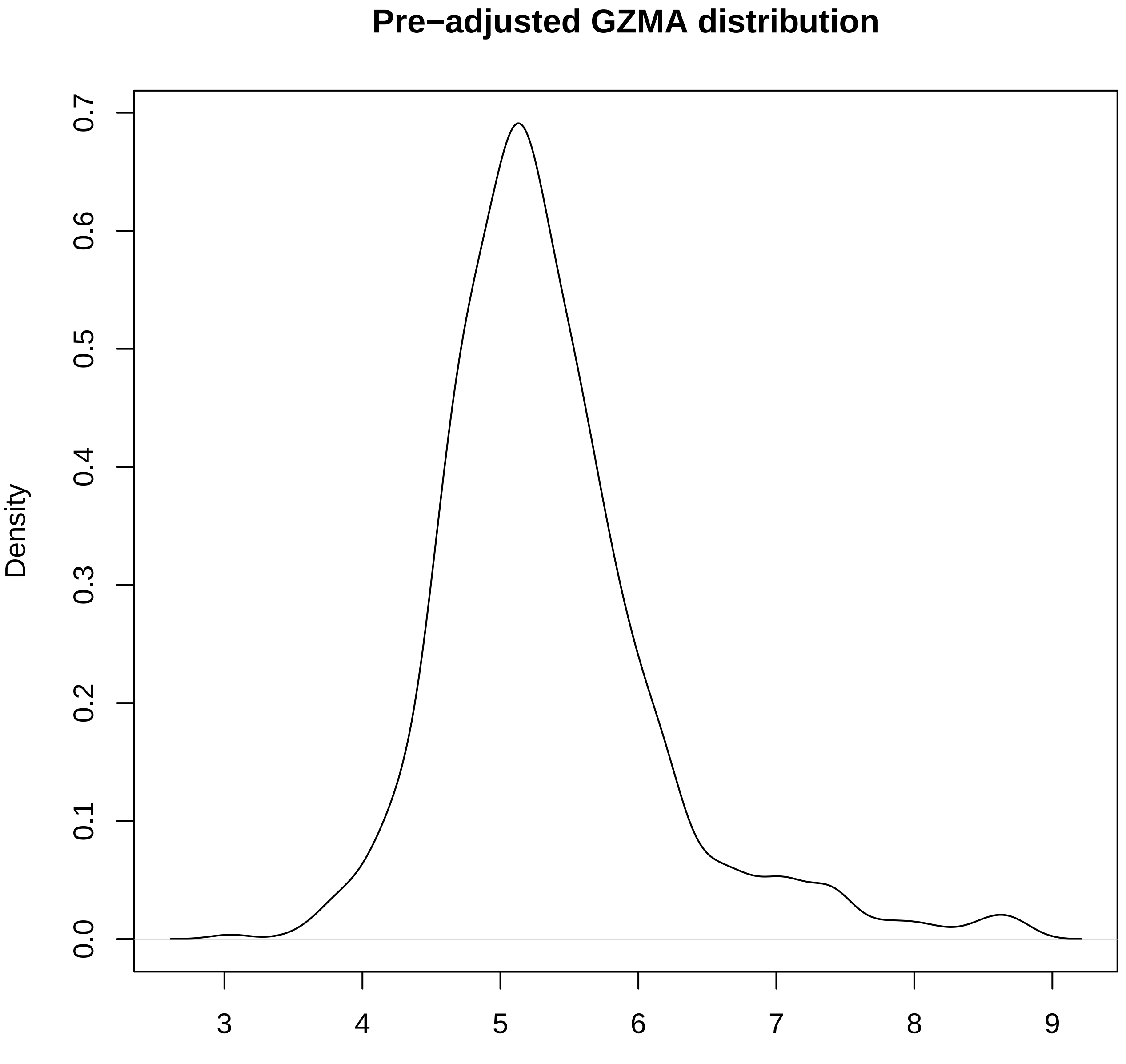
Pre−adjusted GZMA distribution
Density
0.0
0.1
0.2
0.3
0.4
0.5
0.6
0.7
3
4
5
6
7
8
9

Pre−adjusted G−CSF distribution

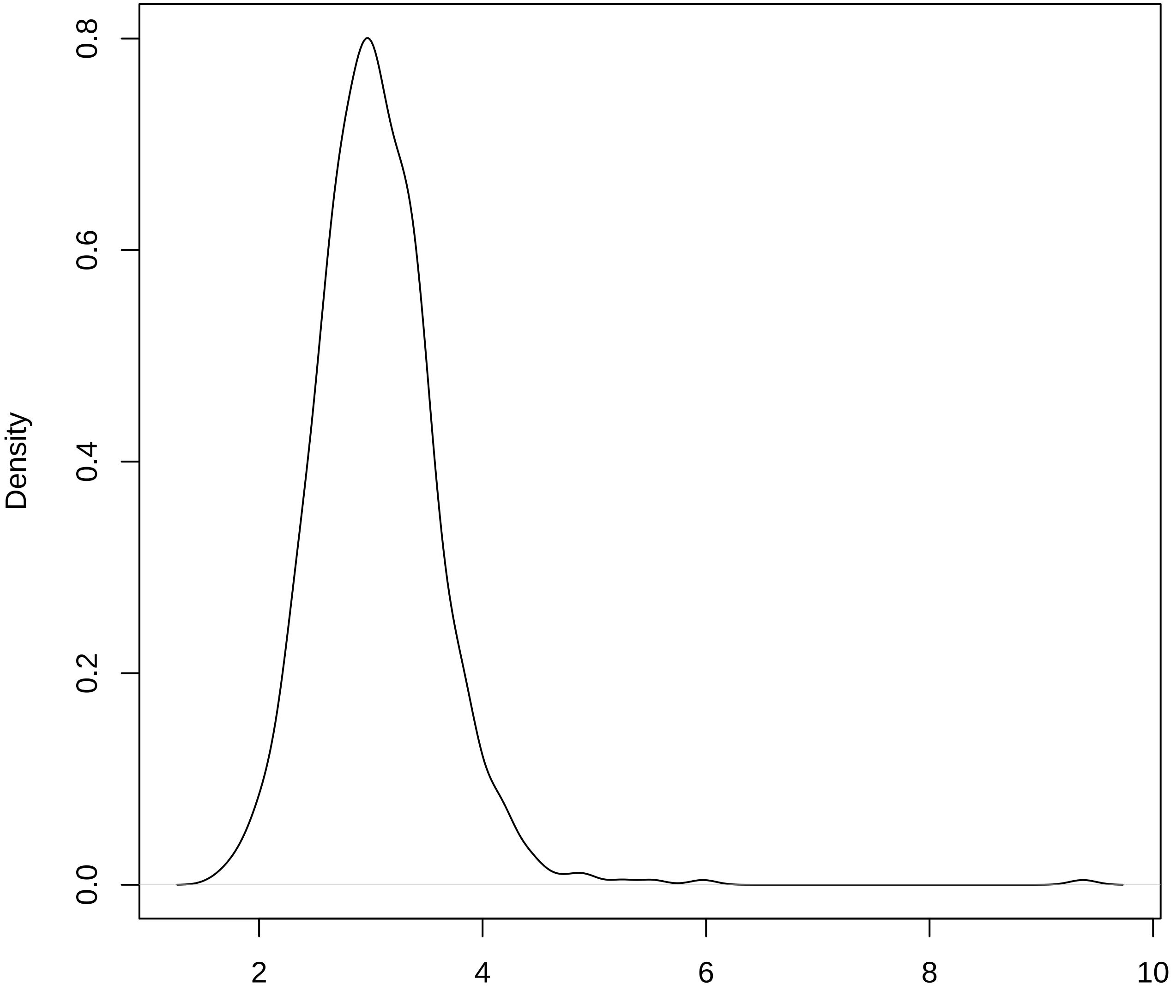

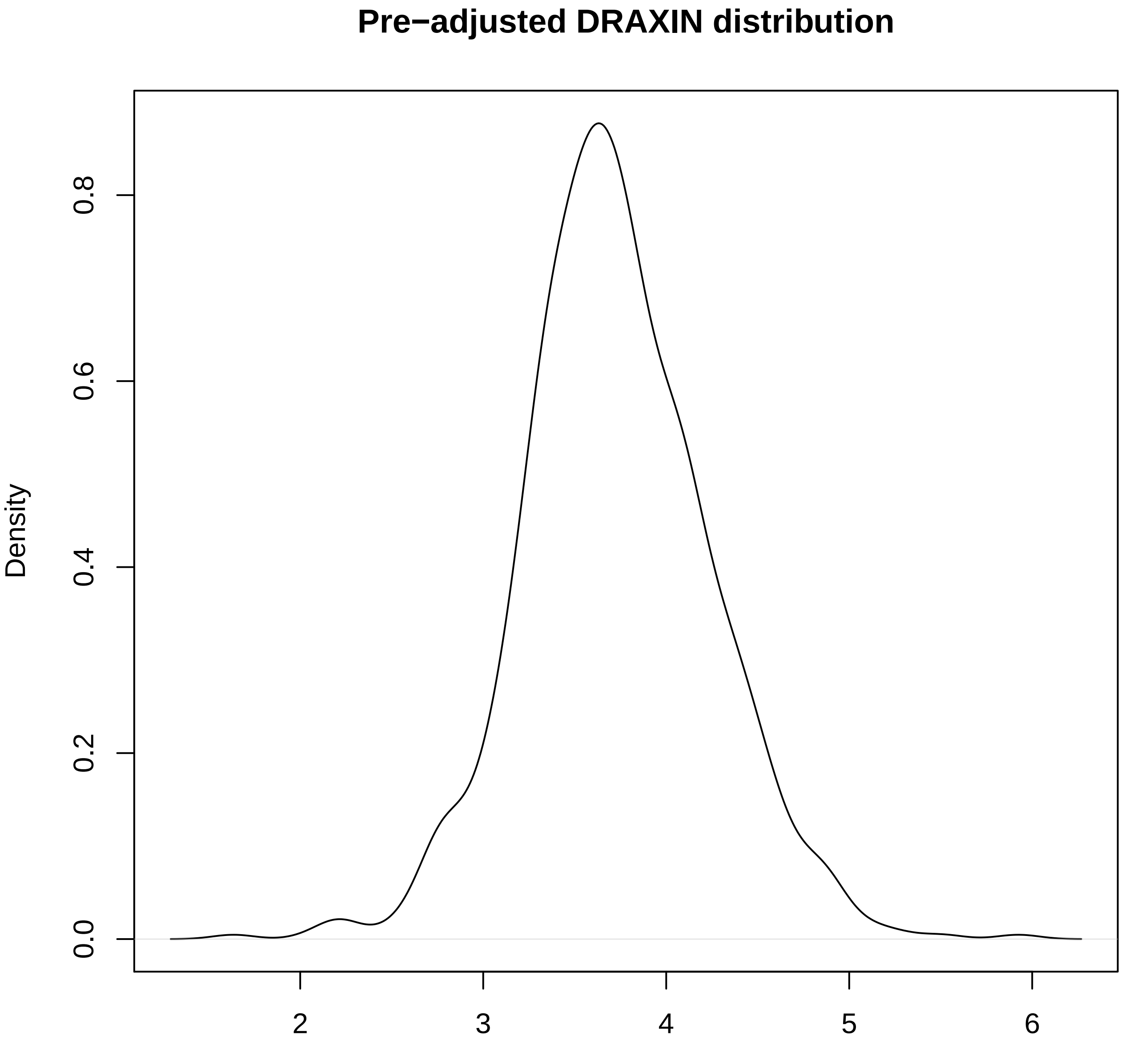

Pre−adjusted DRAXIN distribution
Density
0.0
0.2
0.4
0.6
0.8
2
3
4
5
6

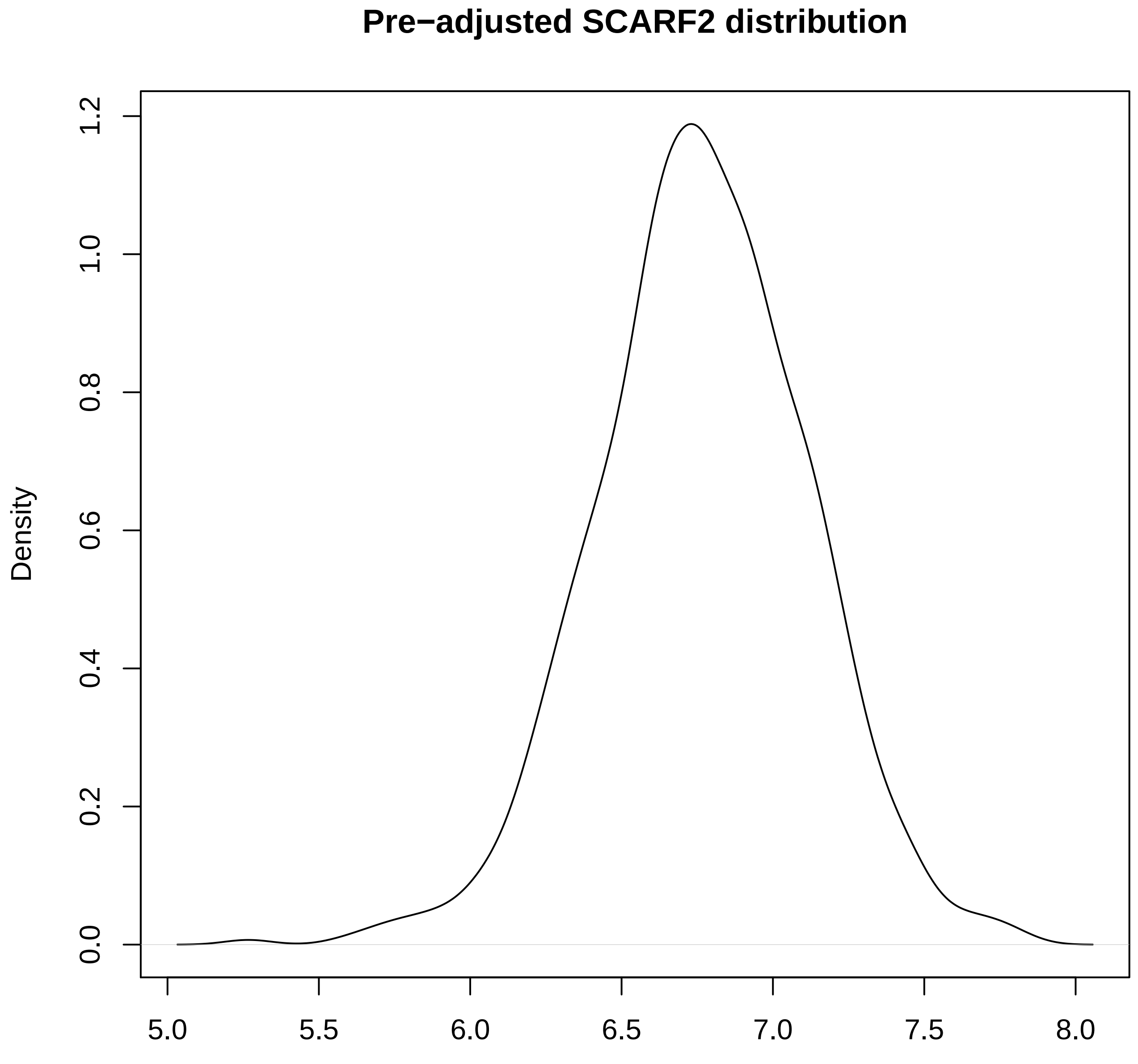
Pre−adjusted SCARF2 distribution
Density
0.0
0.2
0.4
0.6
0.8
1.0
1.2
5.0
5.5
6.0
6.5
7.0
7.5
8.0

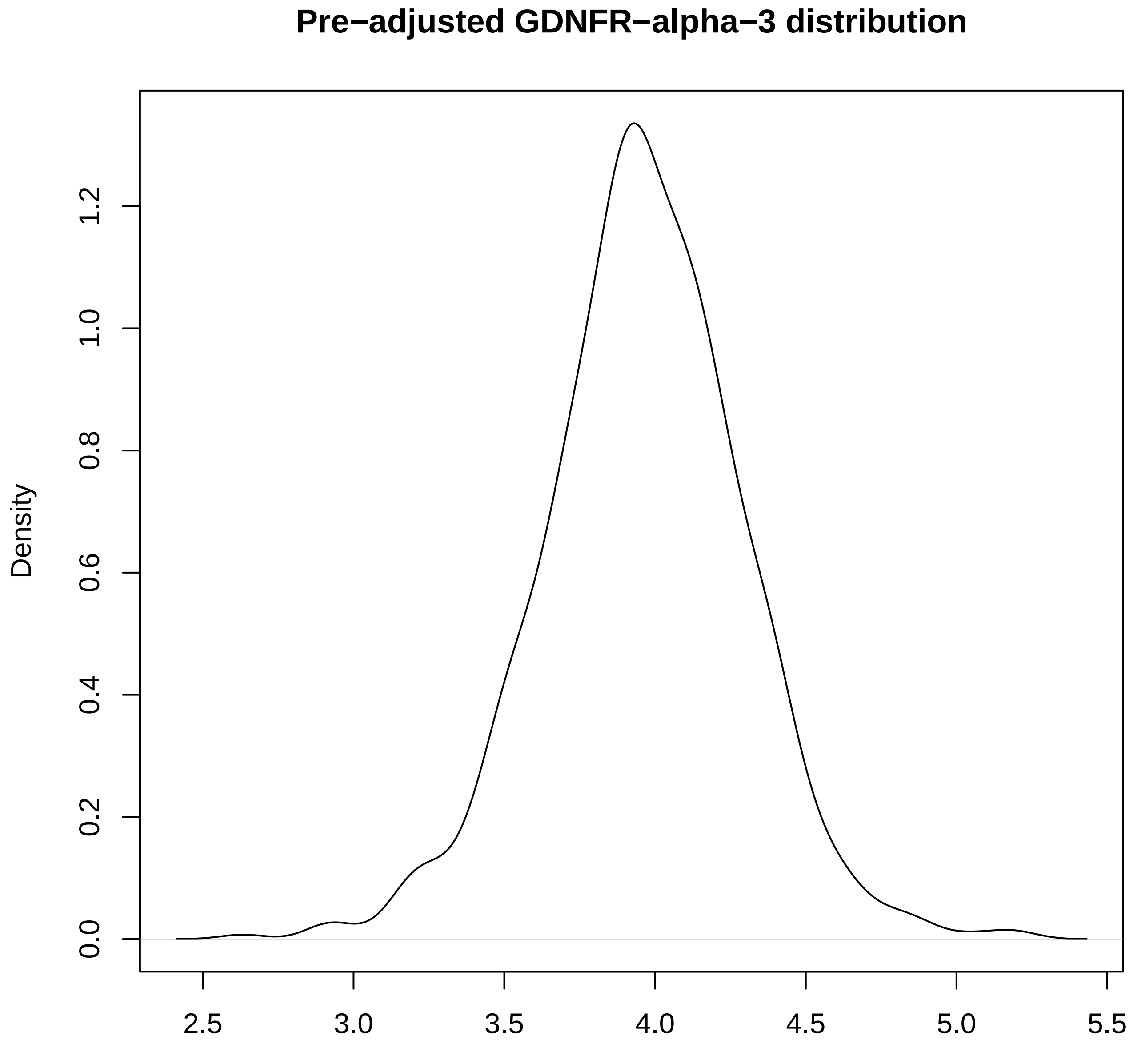

Pre−adjusted GDNFR−alpha−3 distribution
Density
0.0
0.2
0.4
0.6
0.8
1.0
1.2
2.5
3.0
3.5
4.0
4.5
5.0
5.5

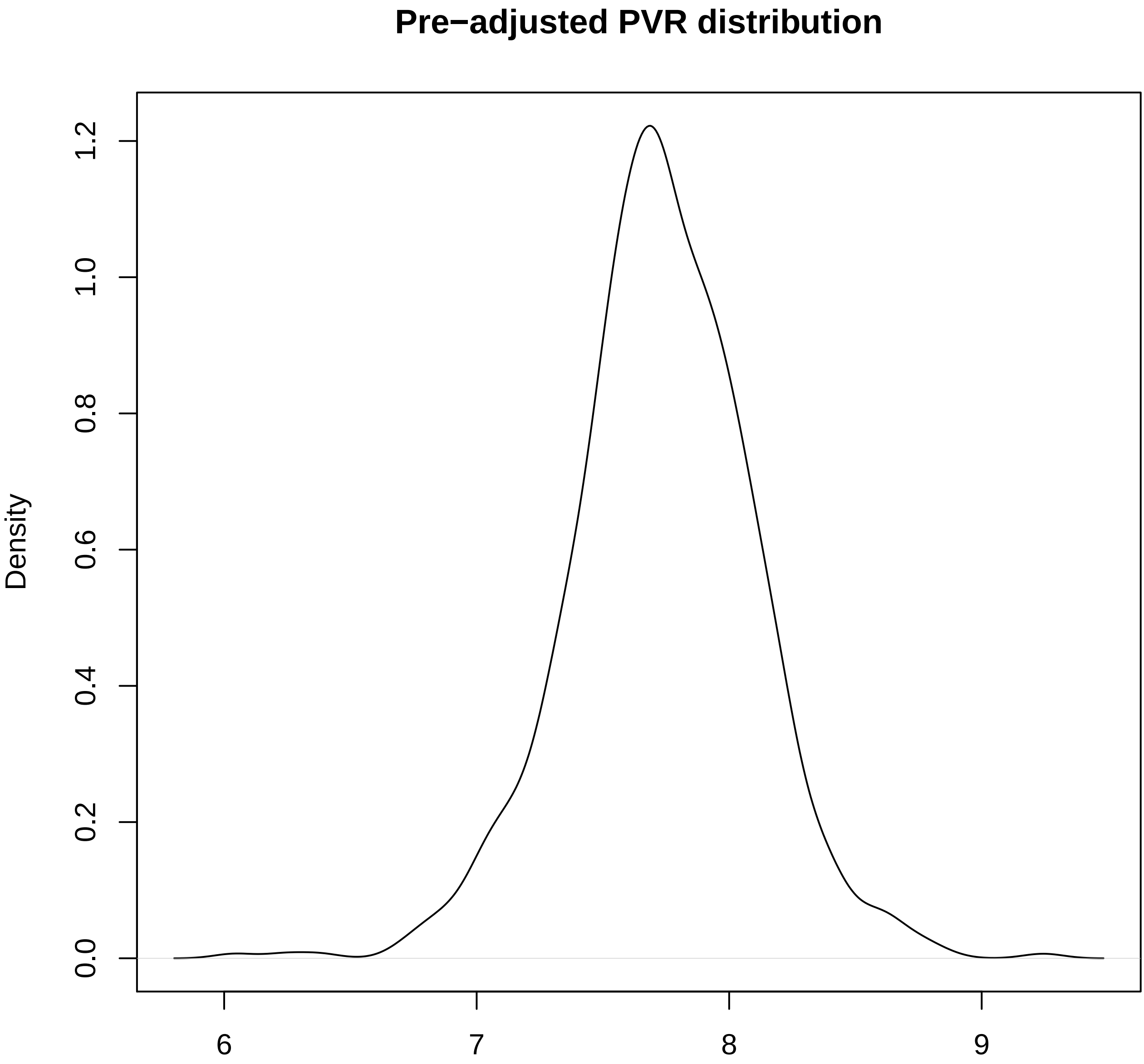
Pre−adjusted PVR distribution
Density
0.0
0.2
0.4
0.6
0.8
1.0
1.2
6
7
8
9

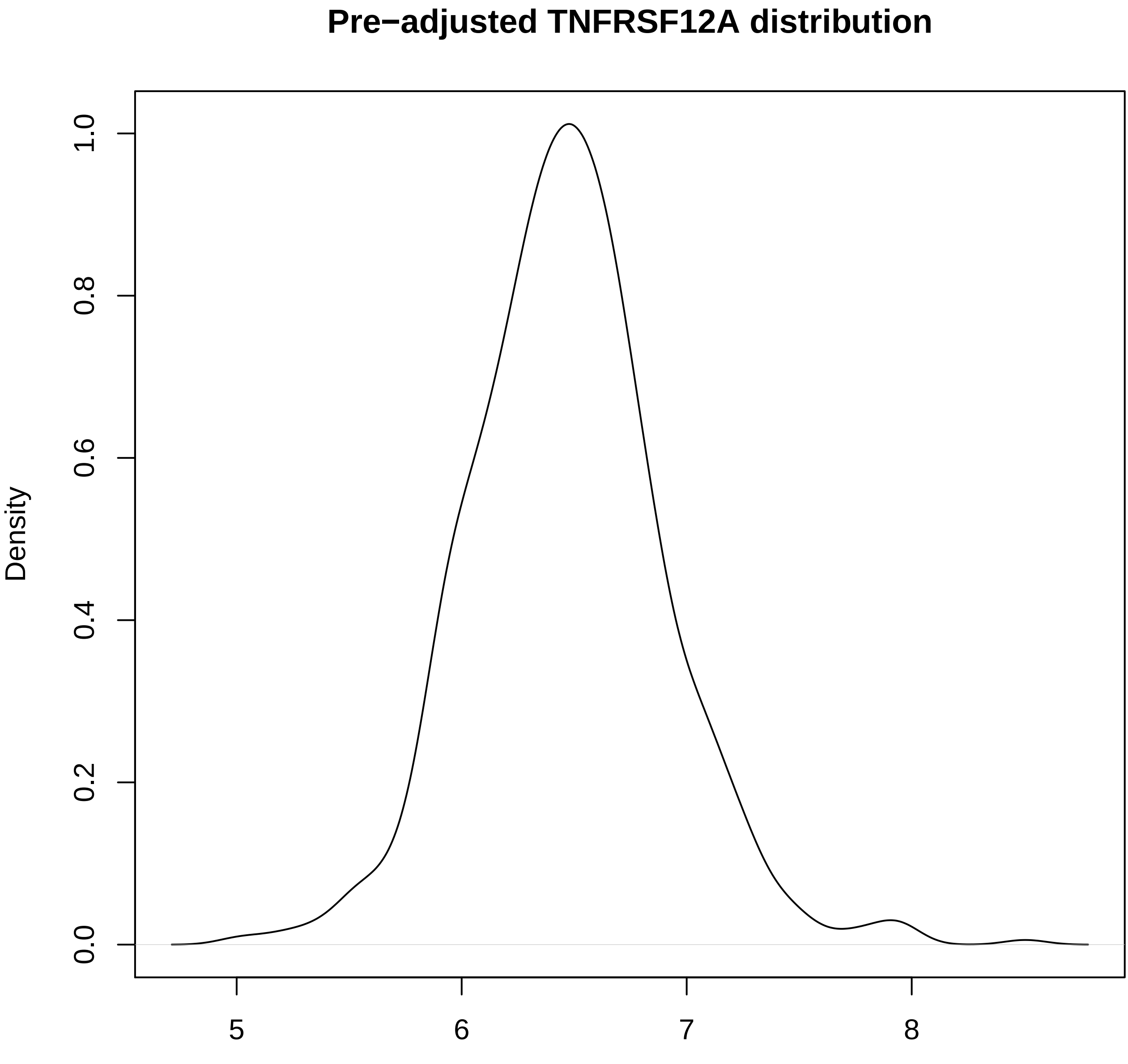
Pre−adjusted TNFRSF12A distribution
Density
0.0
0.2
0.4
0.6
0.8
1.0
5
6
7
8

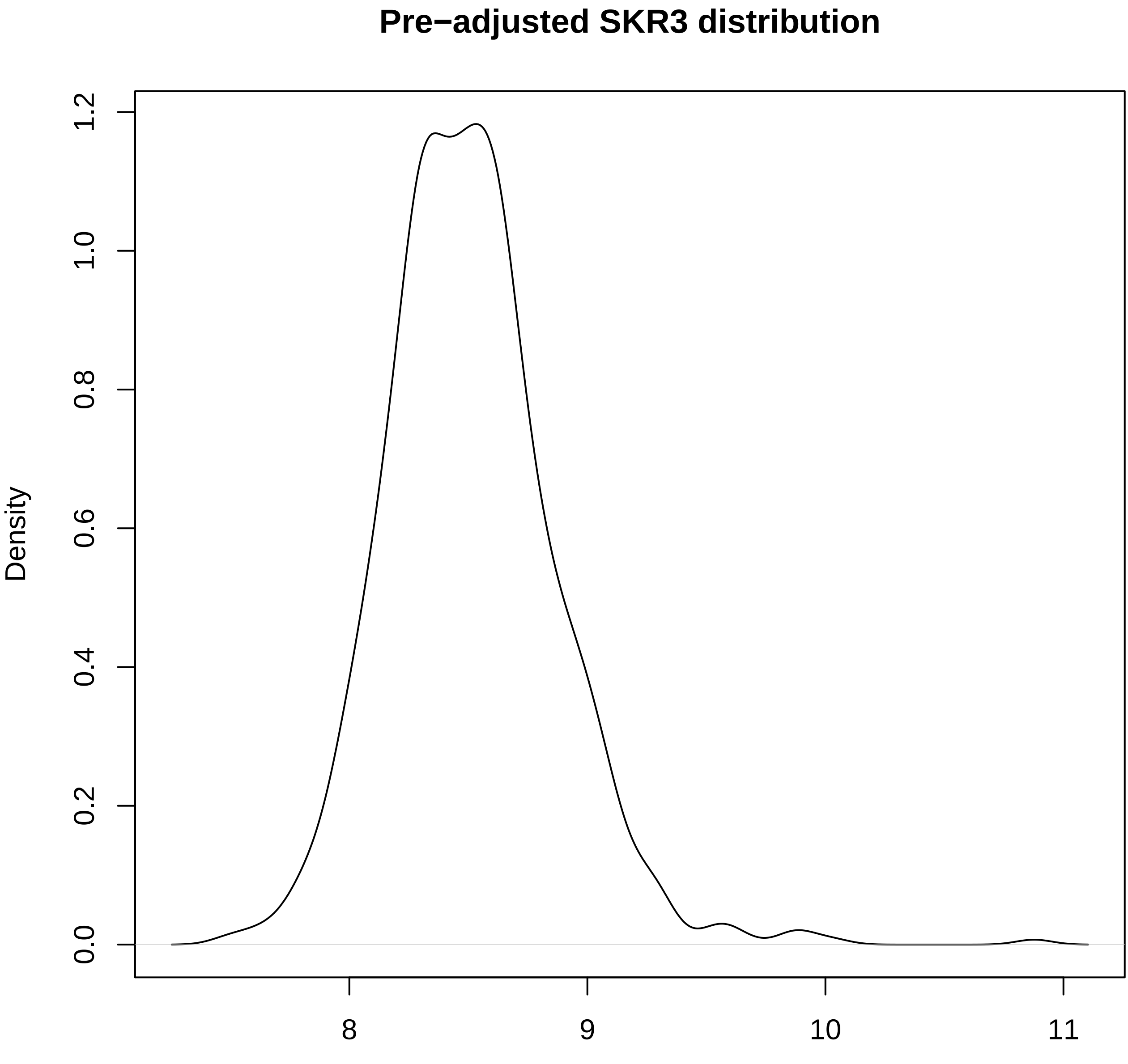
Pre−adjusted SKR3 distribution
Density
0.0
0.2
0.4
0.6
0.8
1.0
1.2
8
9
10
11

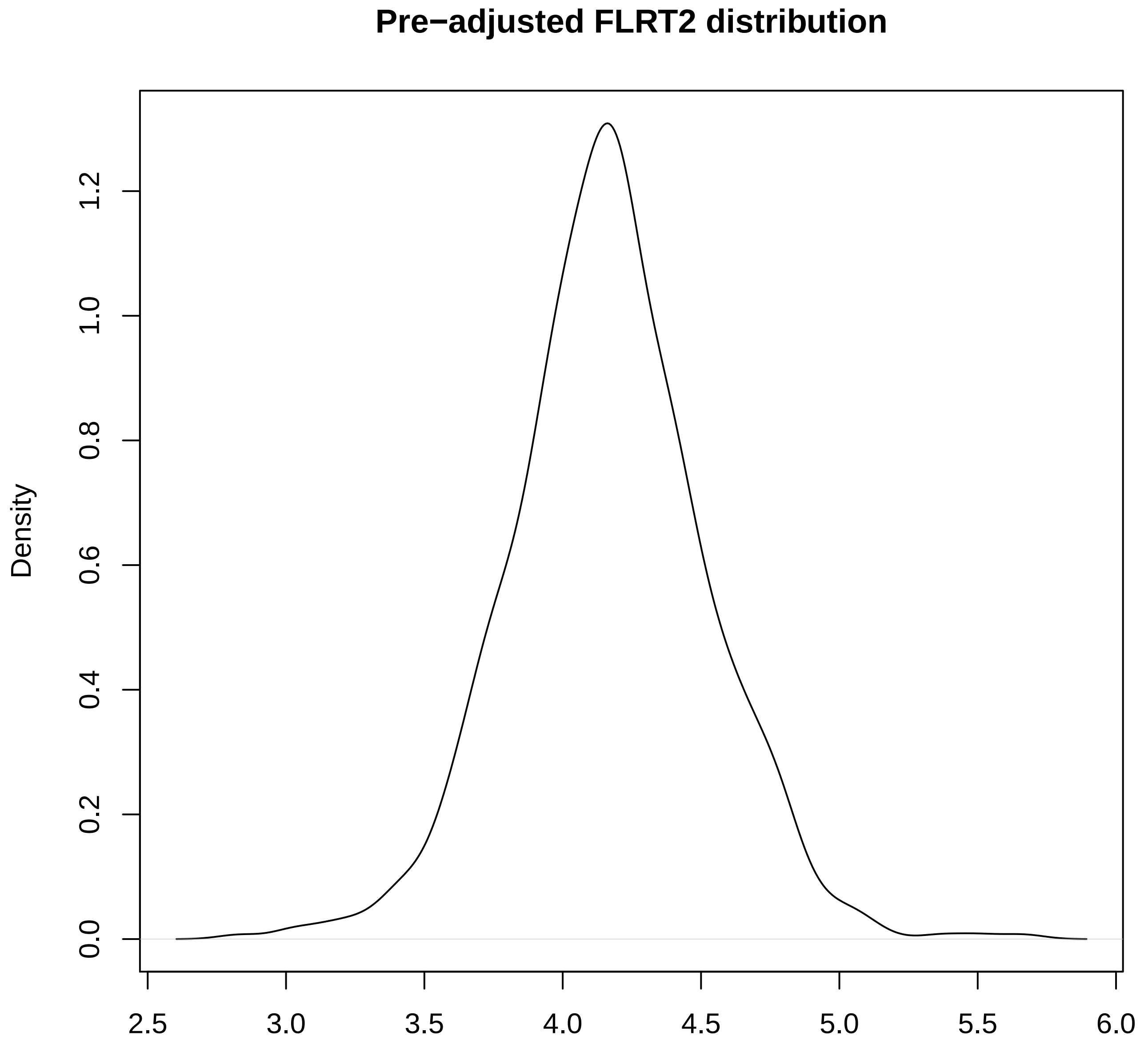
Pre−adjusted FLRT2 distribution
Density
0.0
0.2
0.4
0.6
0.8
1.0
1.2
2.5
3.0
3.5
4.0
4.5
5.0
5.5
6.0

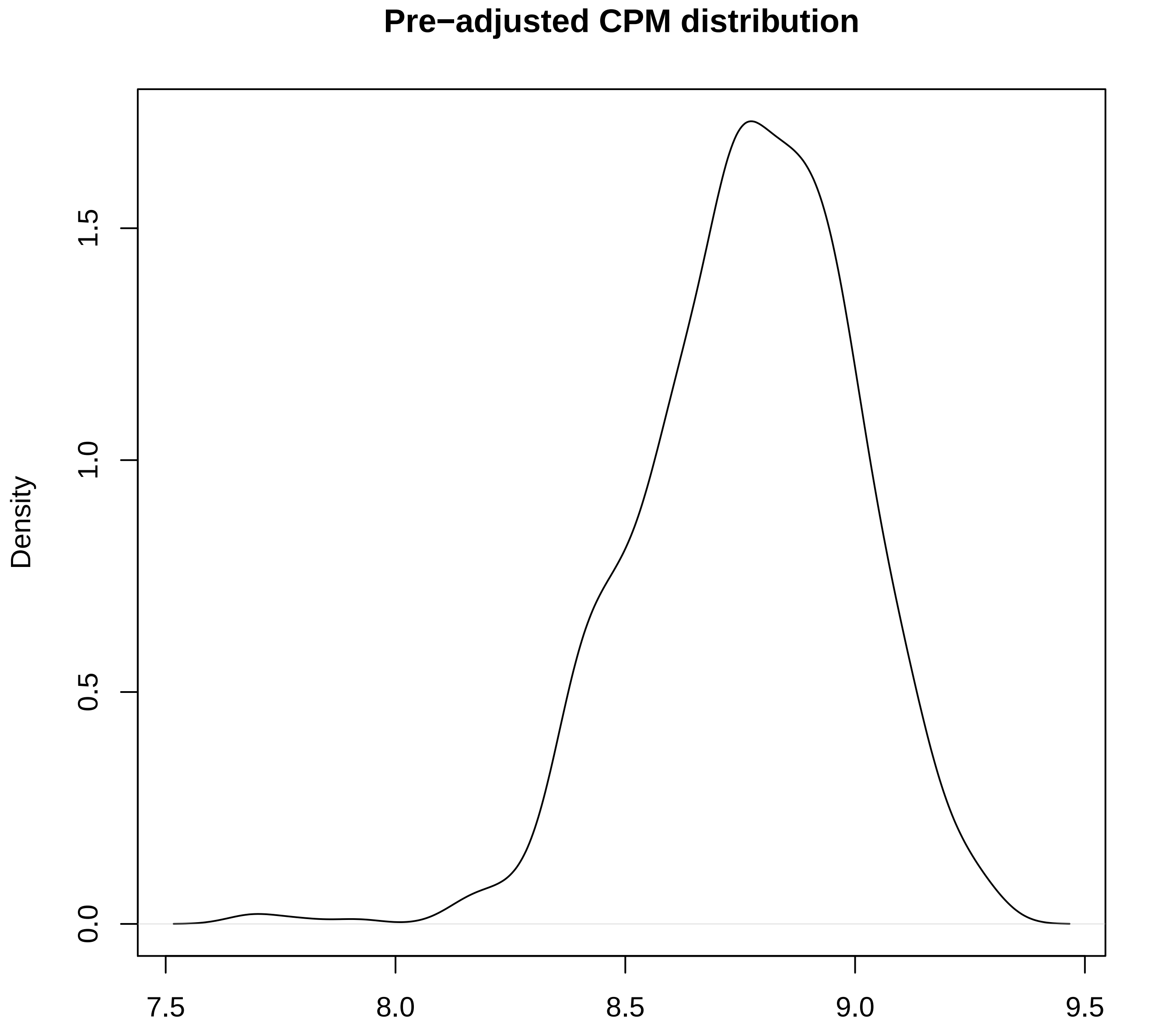

Pre−adjusted CPM distribution
Density
0.0
0.5
1.0
1.5
7.5
8.0
8.5
9.0
9.5

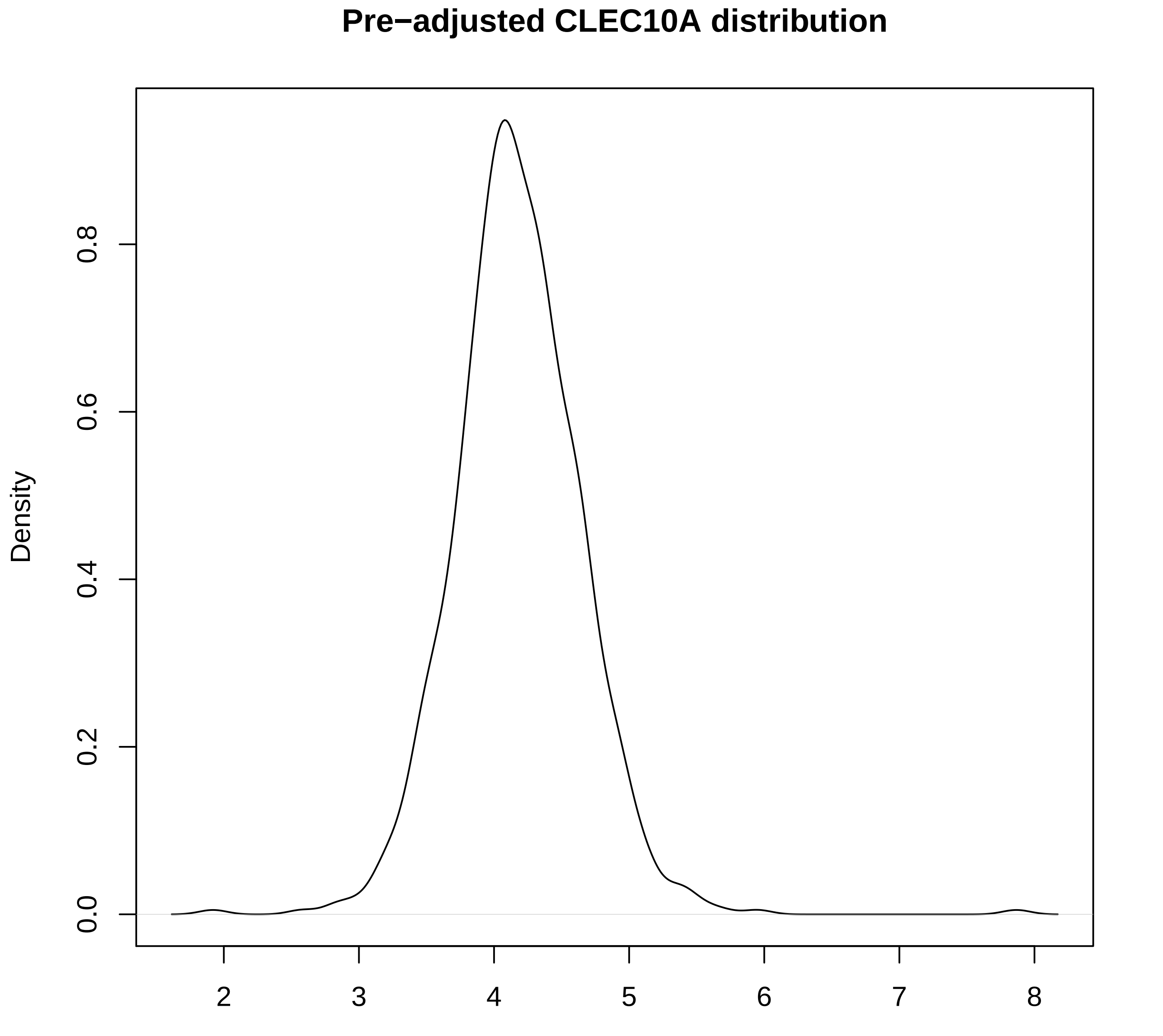
Pre−adjusted CLEC10A distribution
Density
0.0
0.2
0.4
0.6
0.8
2
3
4
5
6
7
8

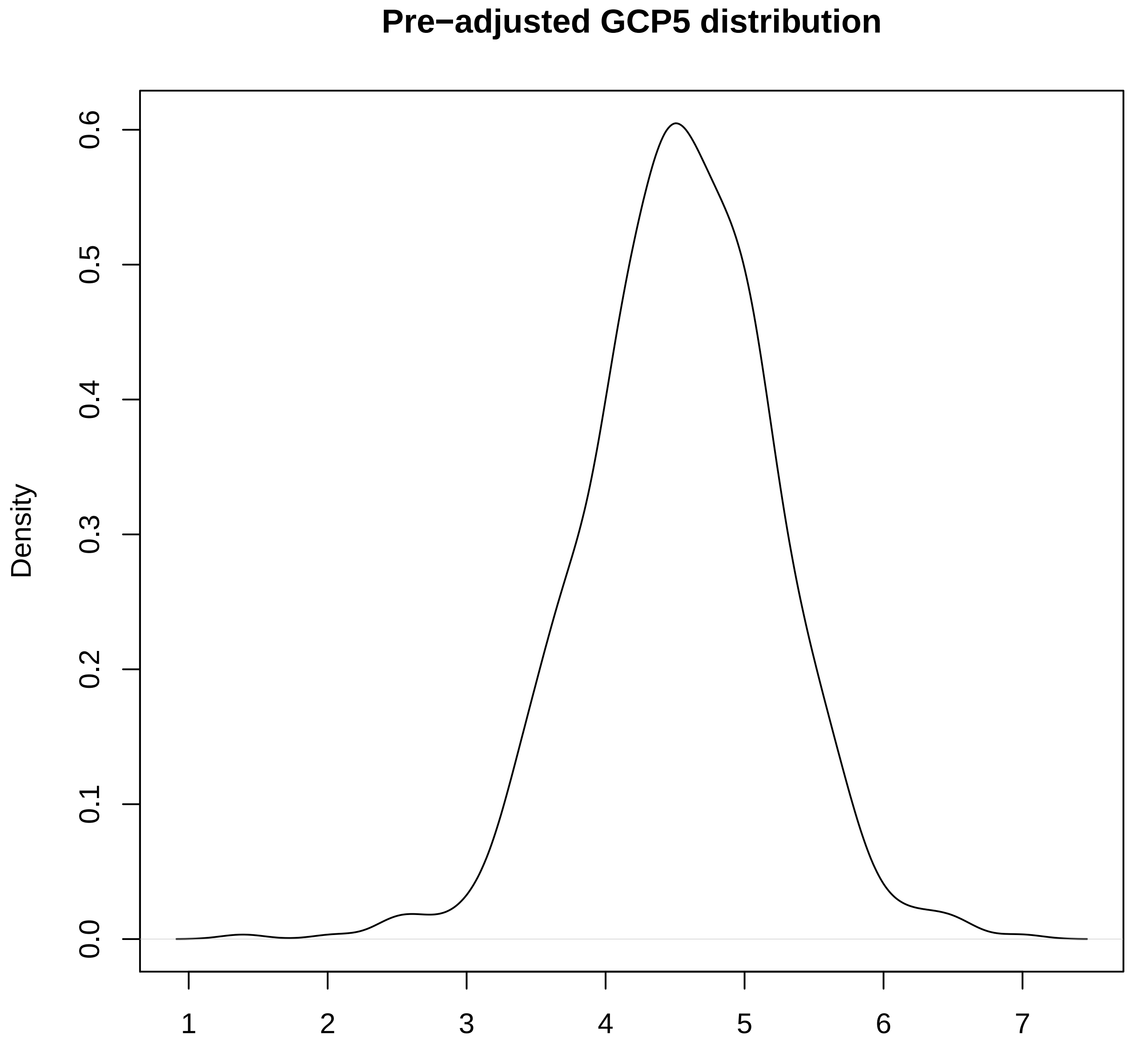
Pre−adjusted GCP5 distribution
Density
0.0
0.1
0.2
0.3
0.4
0.5
0.6
1
2
3
4
5
6
7

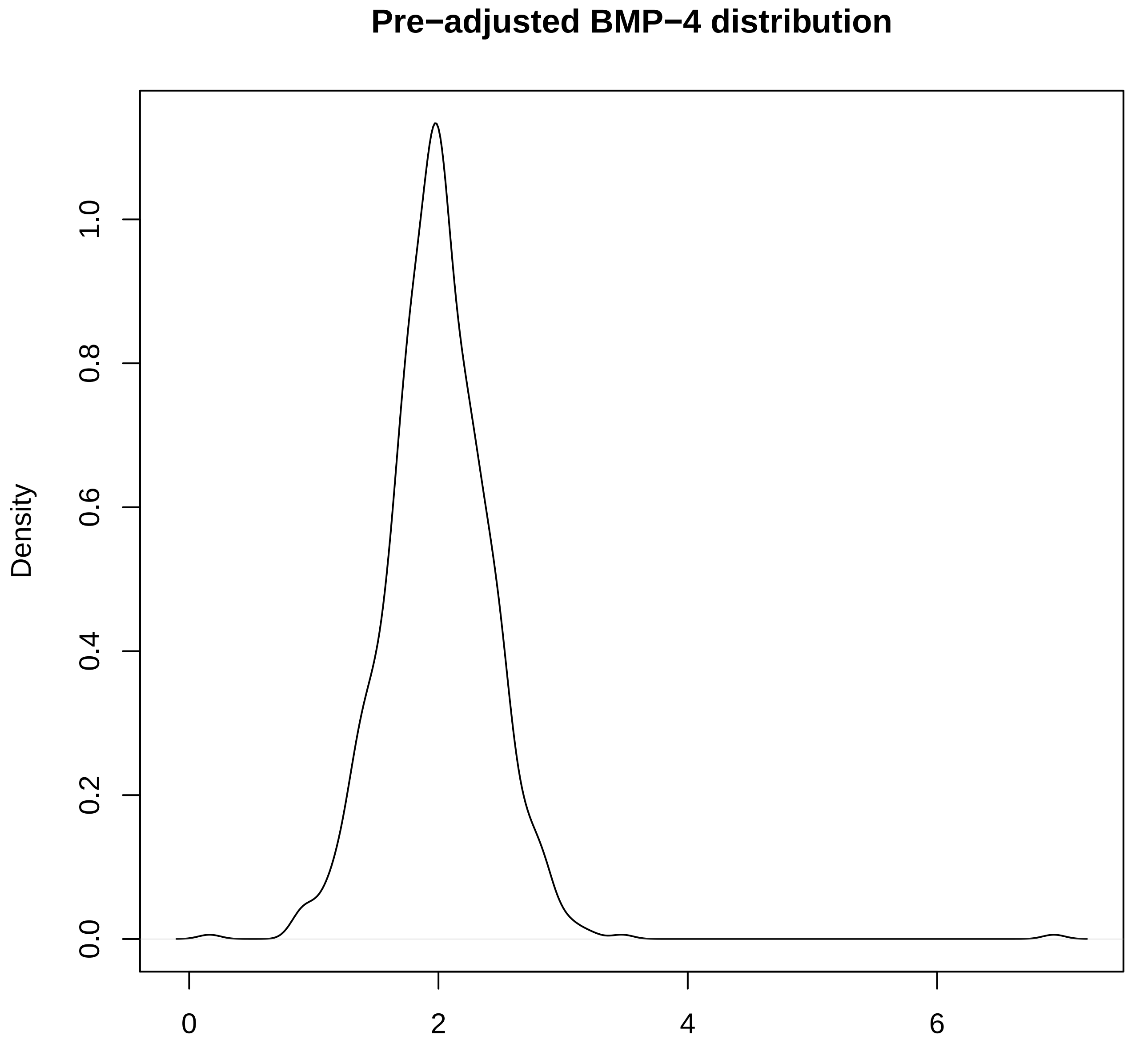
Pre−adjusted BMP−4 distribution
Density
0.0
0.2
0.4
0.6
0.8
1.0
0
2
4
6

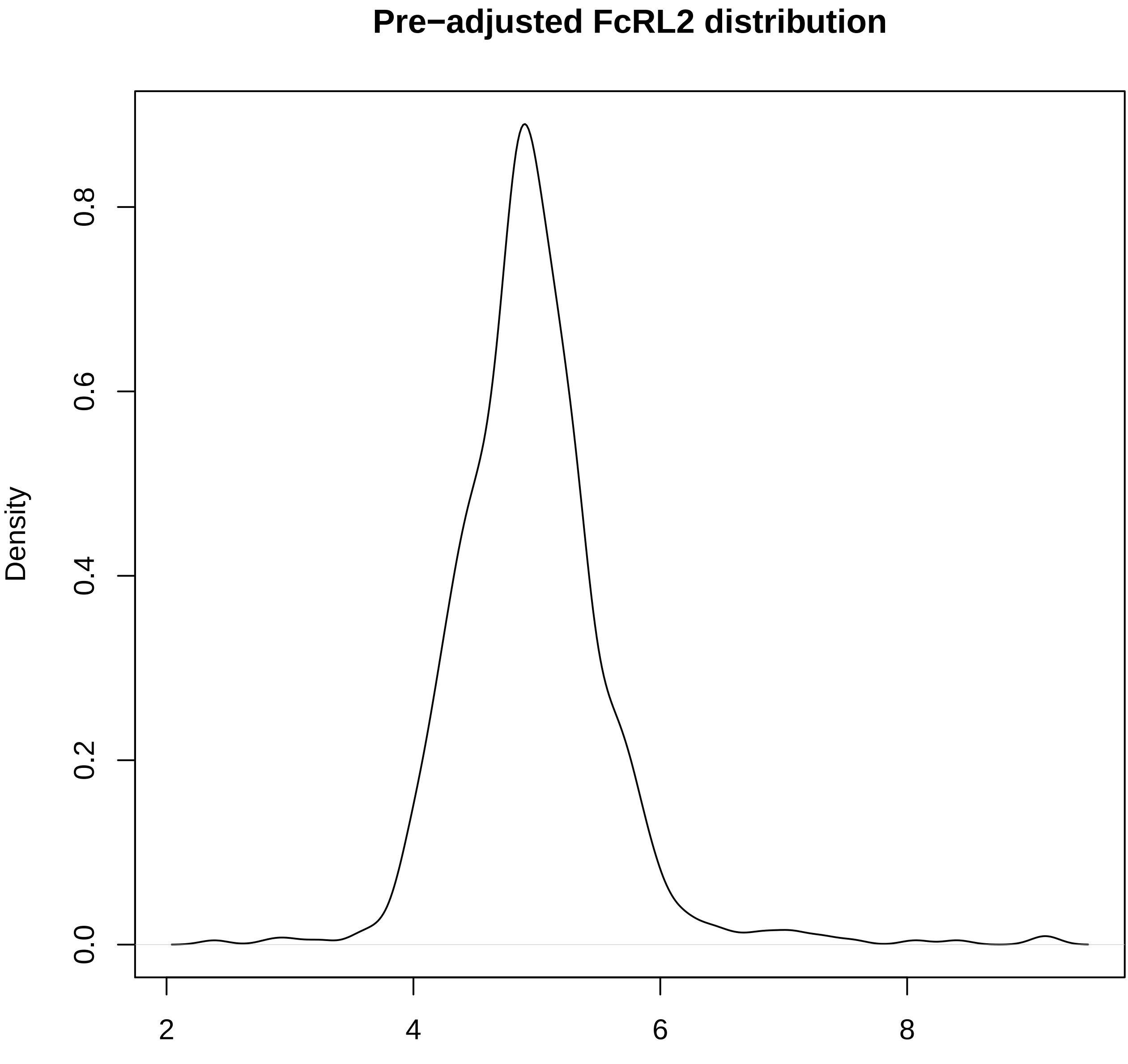
Pre−adjusted FcRL2 distribution
Density
0.0
0.2
0.4
0.6
0.8
2
4
6
8

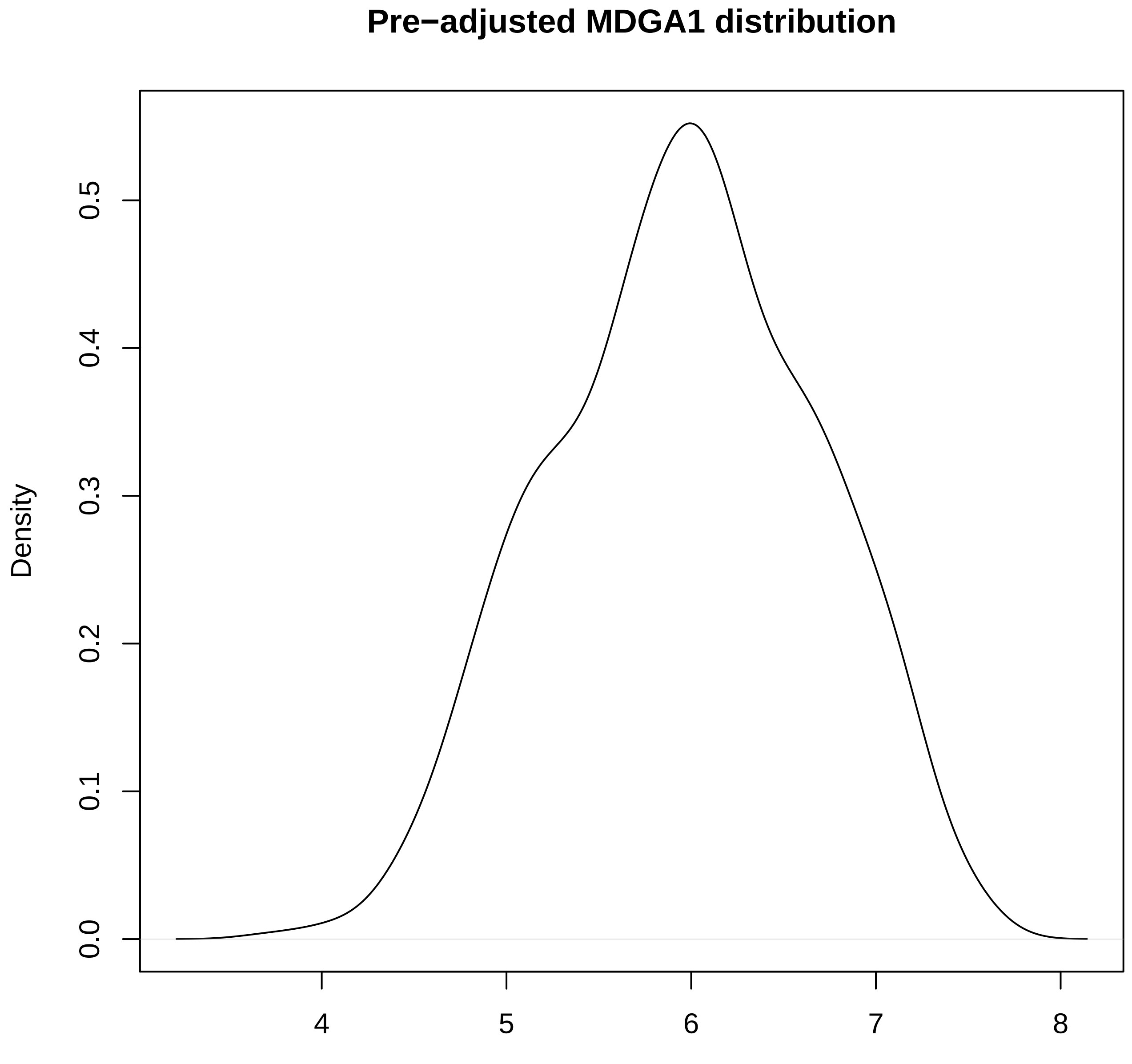
Pre−adjusted MDGA1 distribution
Density
0.0
0.1
0.2
0.3
0.4
0.5
4
5
6
7
8

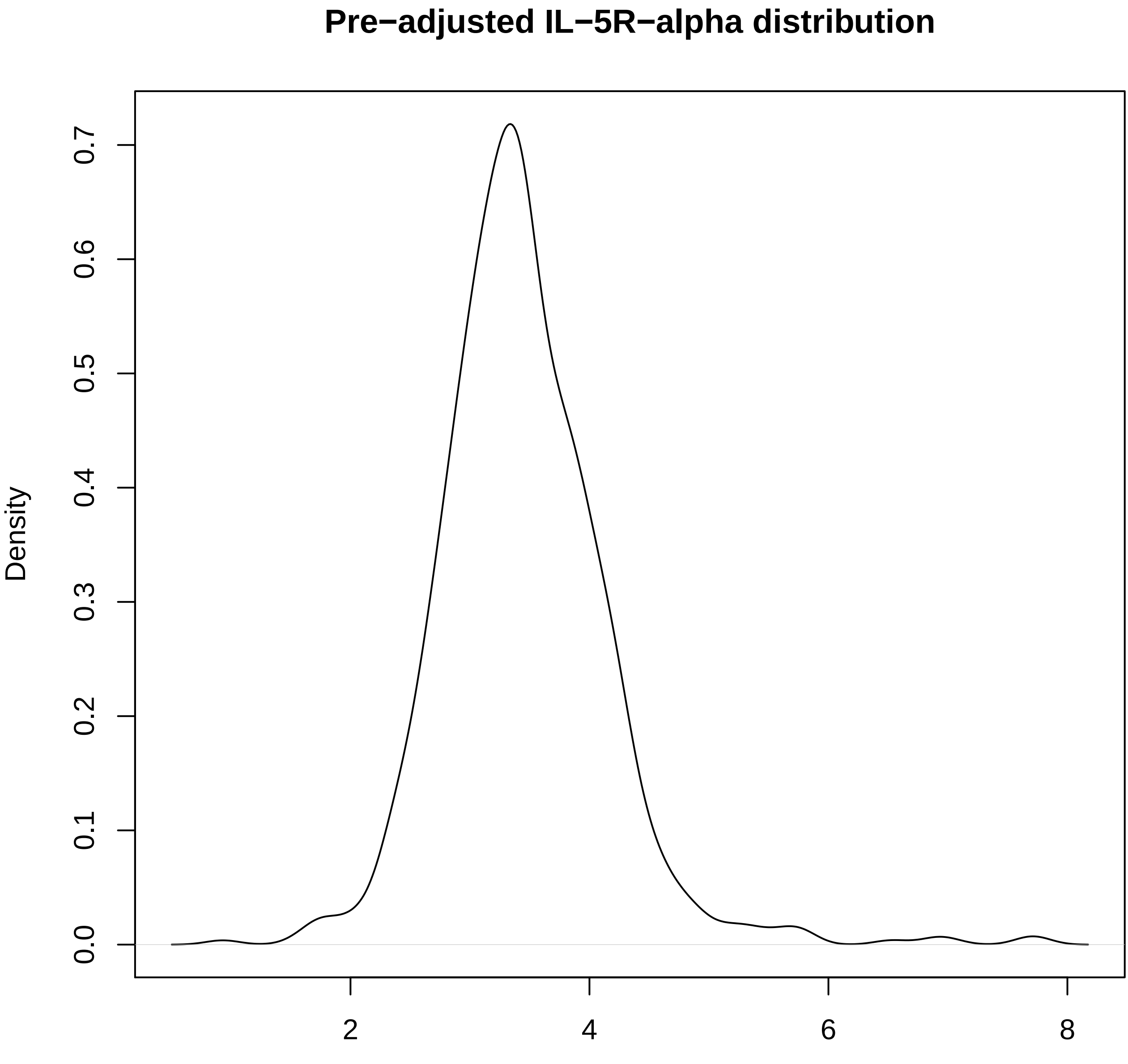
Pre−adjusted IL−5R−alpha distribution
Density
0.0
0.1
0.2
0.3
0.4
0.5
0.6
0.7
2
4
6
8

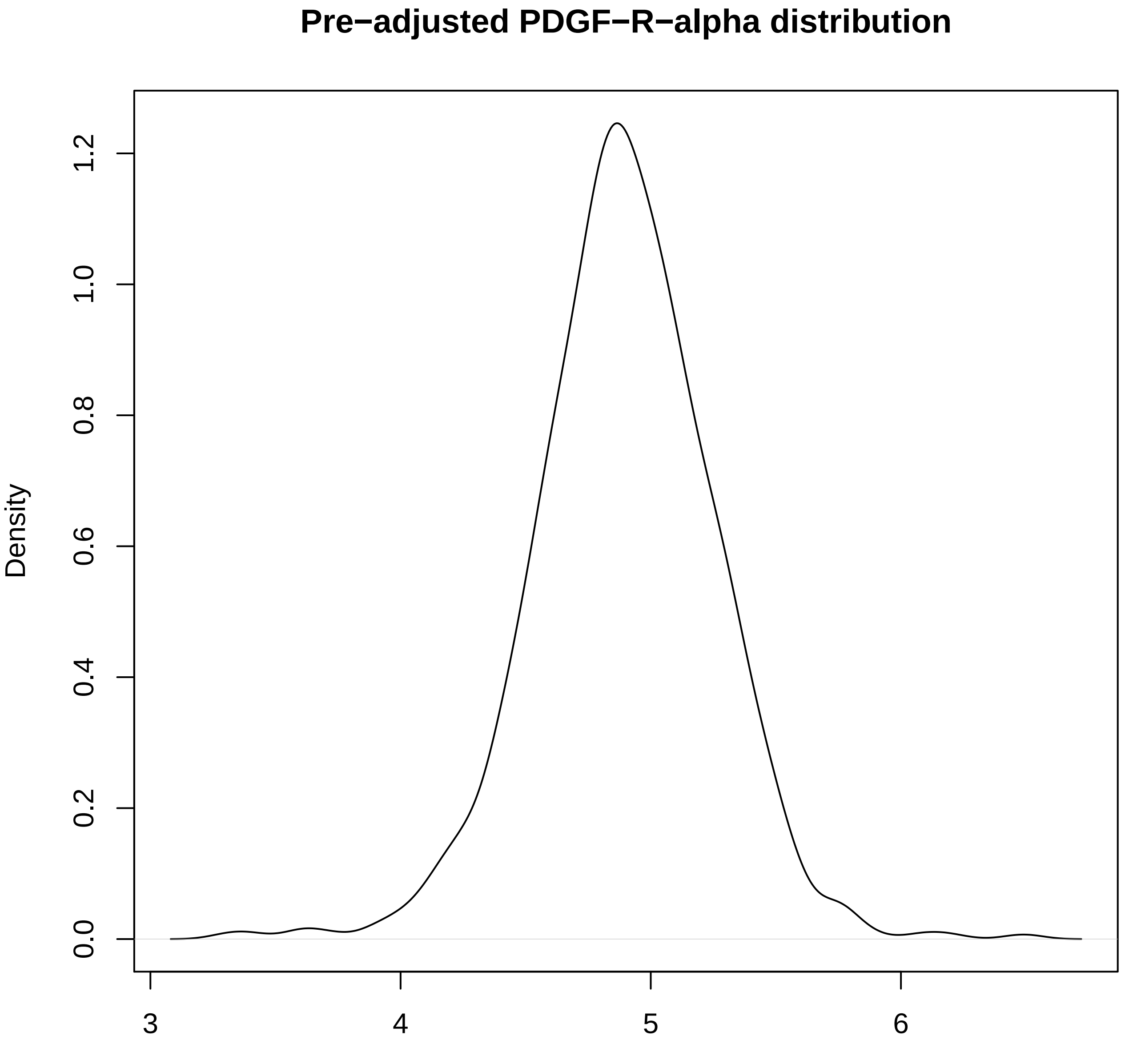
Pre−adjusted PDGF−R−alpha distribution
Density
0.0
0.2
0.4
0.6
0.8
1.0
1.2
3
4
5
6

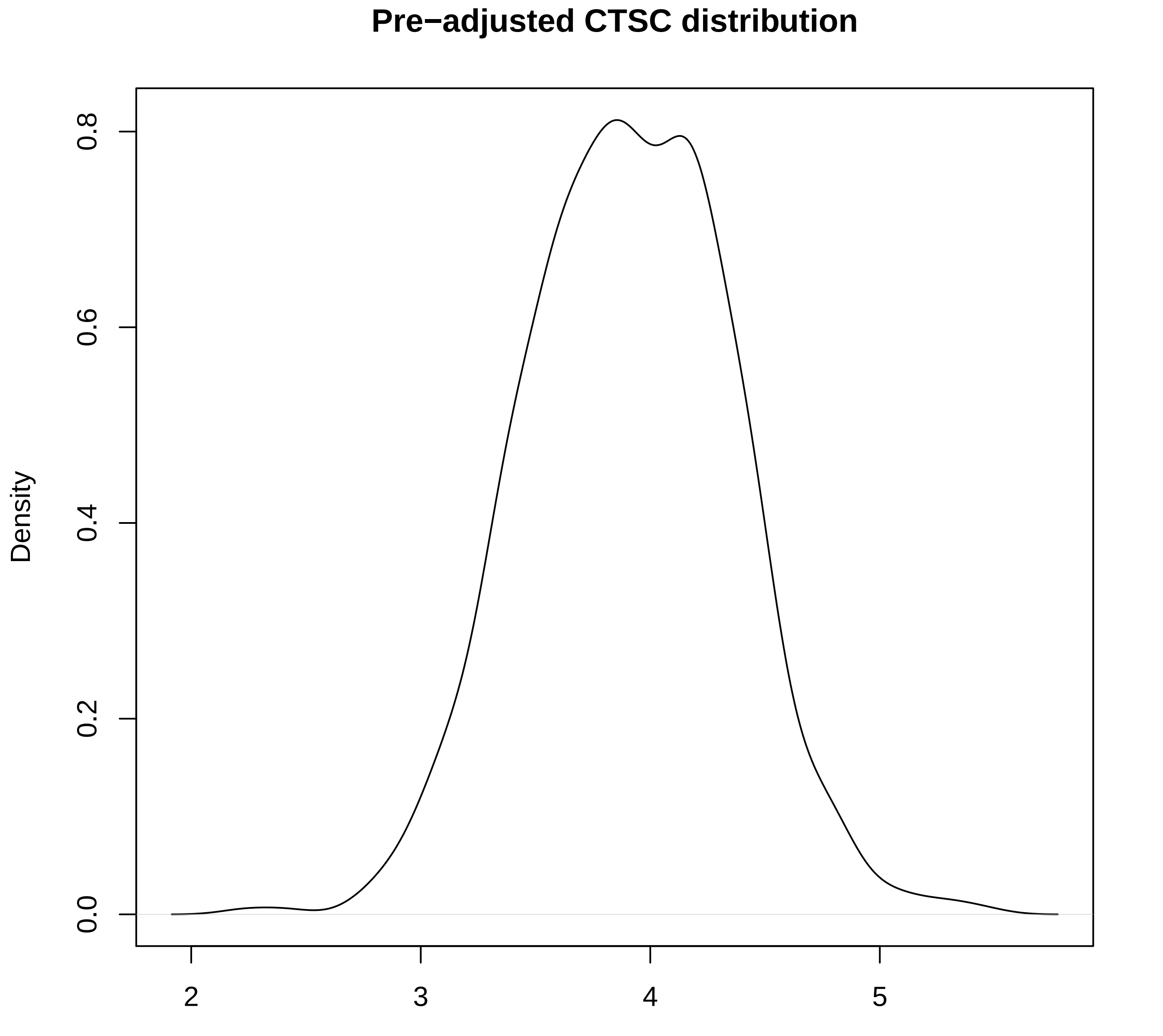
Pre−adjusted CTSC distribution
Density
0.0
0.2
0.4
0.6
0.8
2
3
4
5

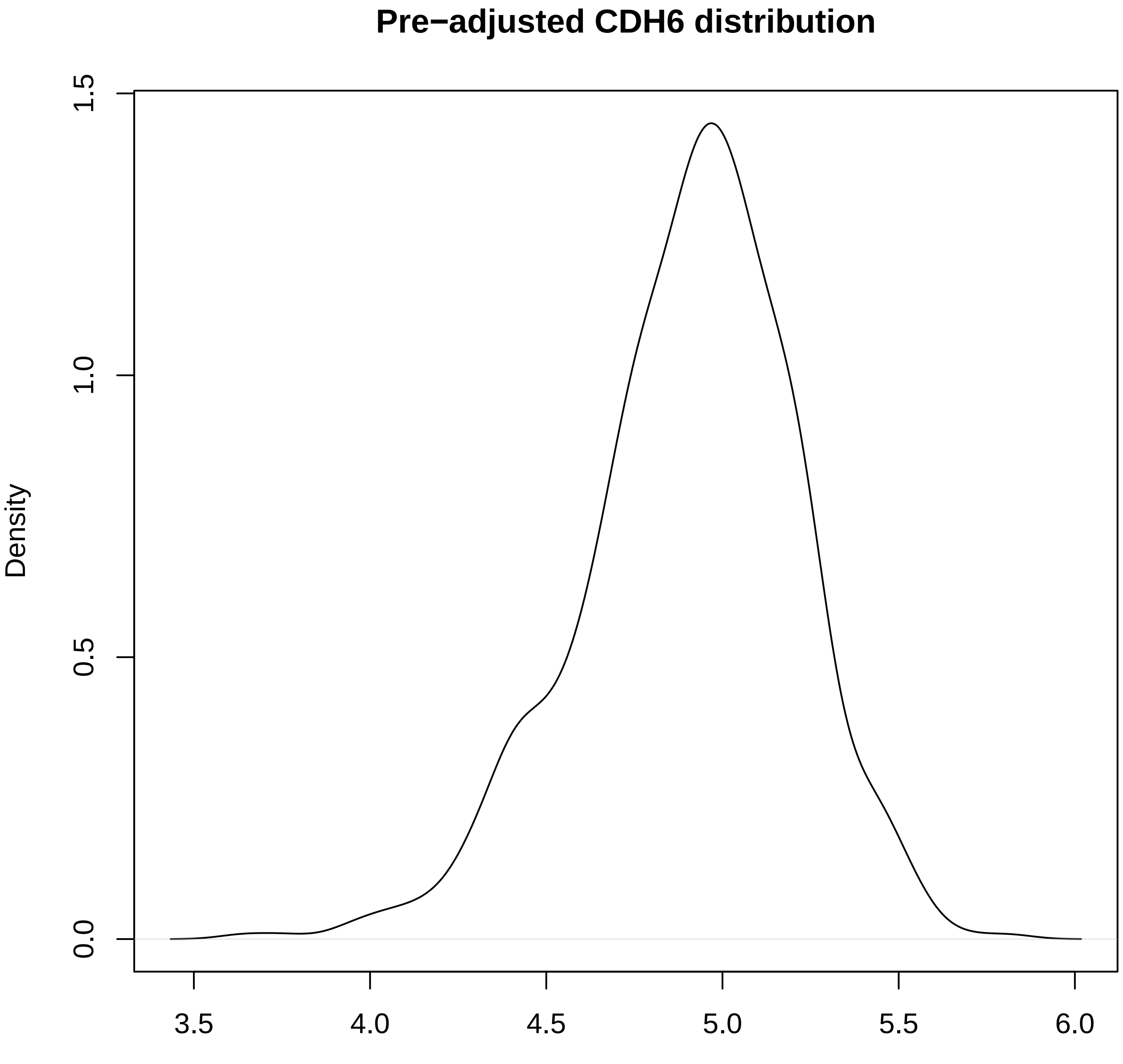
Pre−adjusted CDH6 distribution
Density
0.0
0.5
1.0
1.5
3.5
4.0
4.5
5.0
5.5
6.0

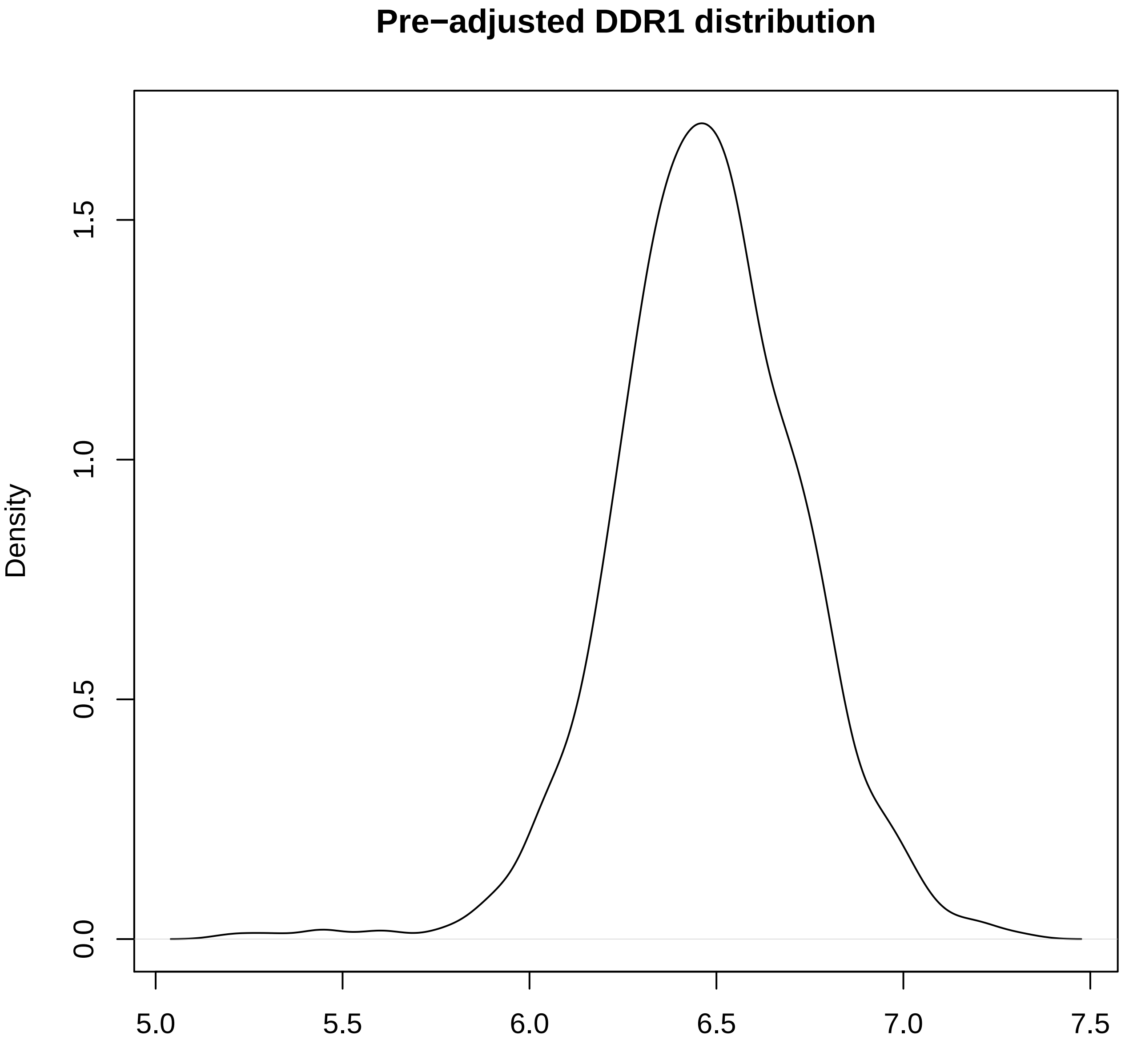

Pre−adjusted DDR1 distribution
Density
0.0
0.5
1.0
1.5
5.0
5.5
6.0
6.5
7.0
7.5

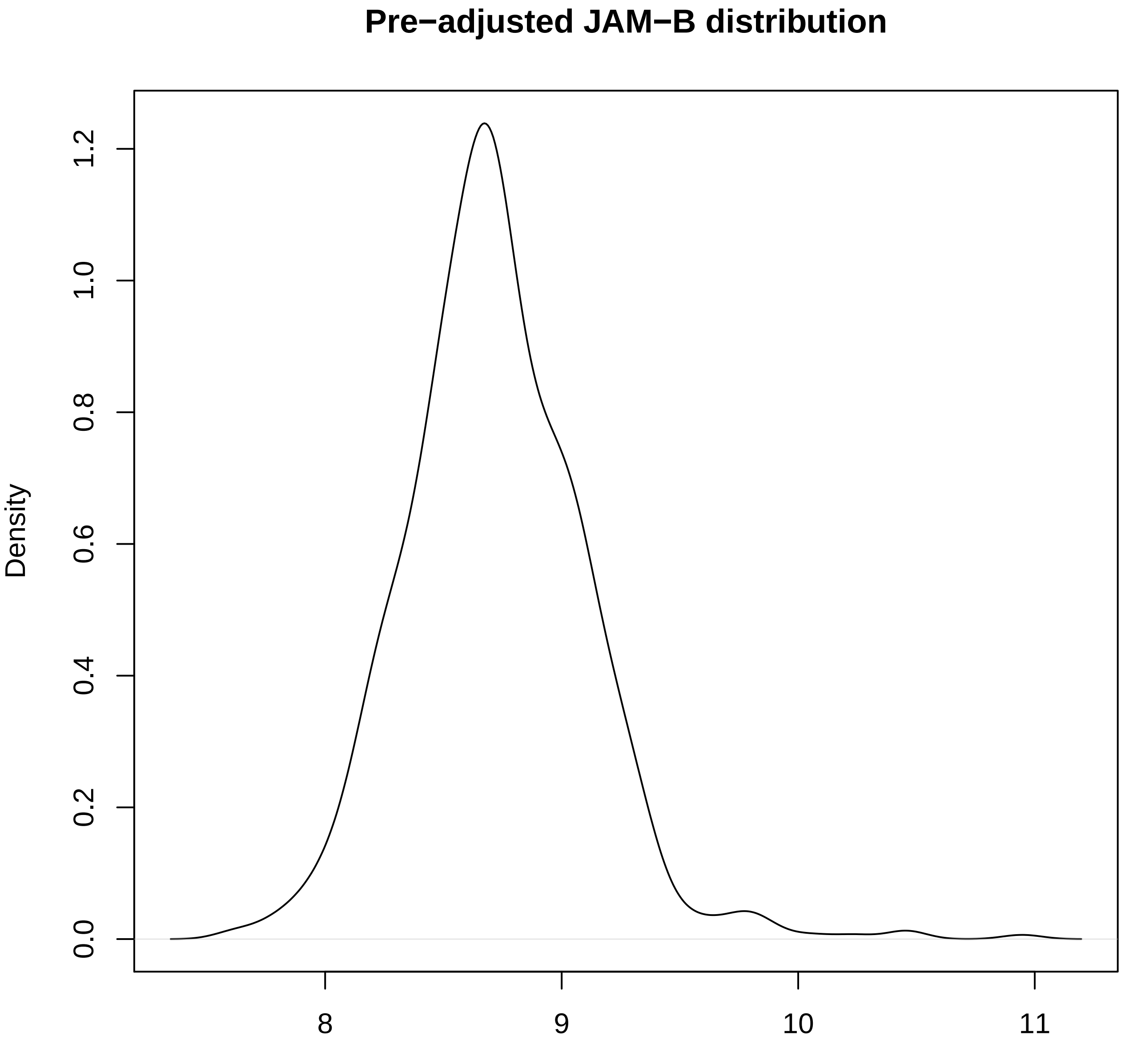
Pre−adjusted JAM−B distribution
Density
0.0
0.2
0.4
0.6
0.8
1.0
1.2
8
9
10
11

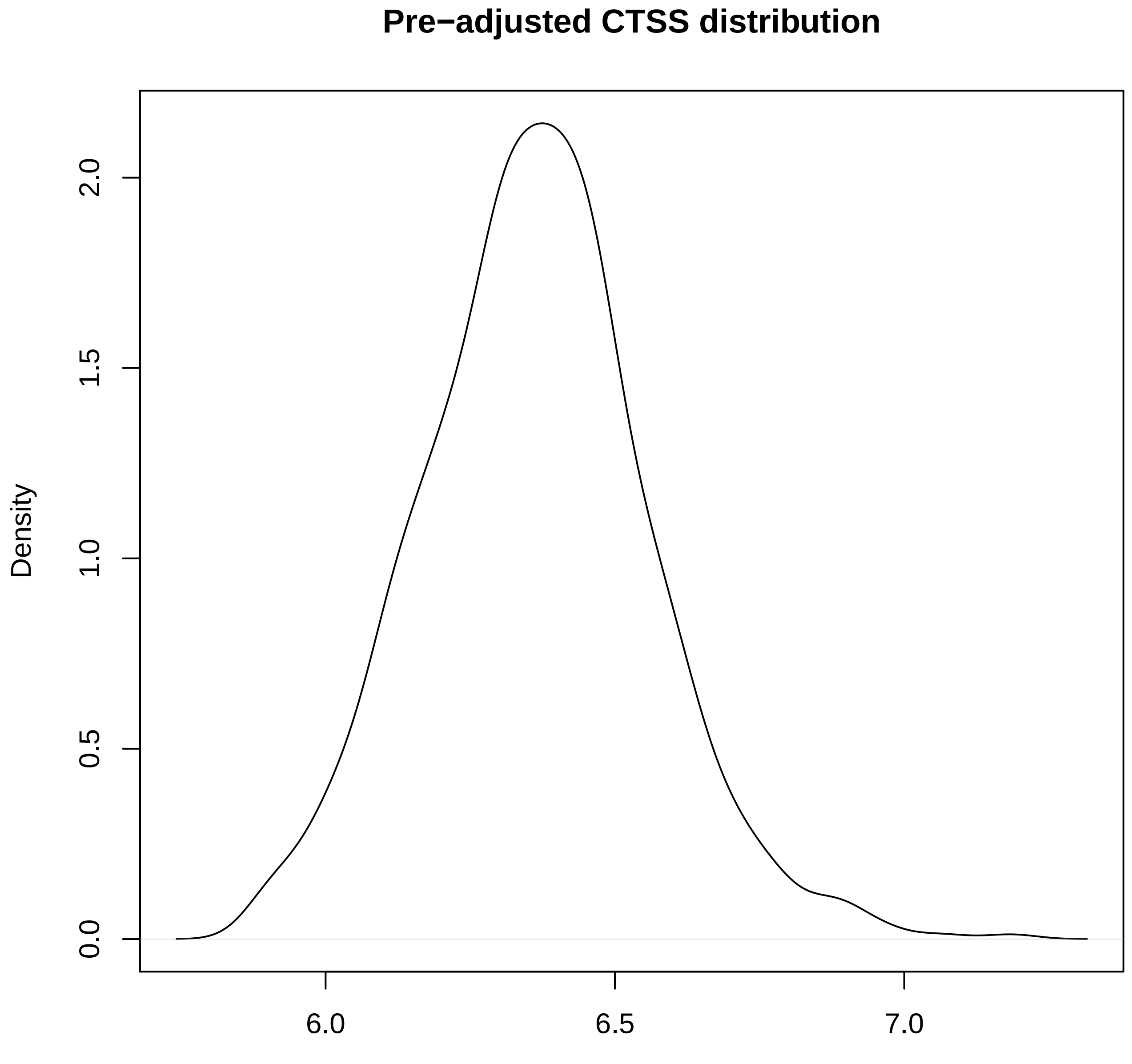
Pre−adjusted CTSS distribution
Density
0.0
0.5
1.0
1.5
2.0
6.0
6.5
7.0

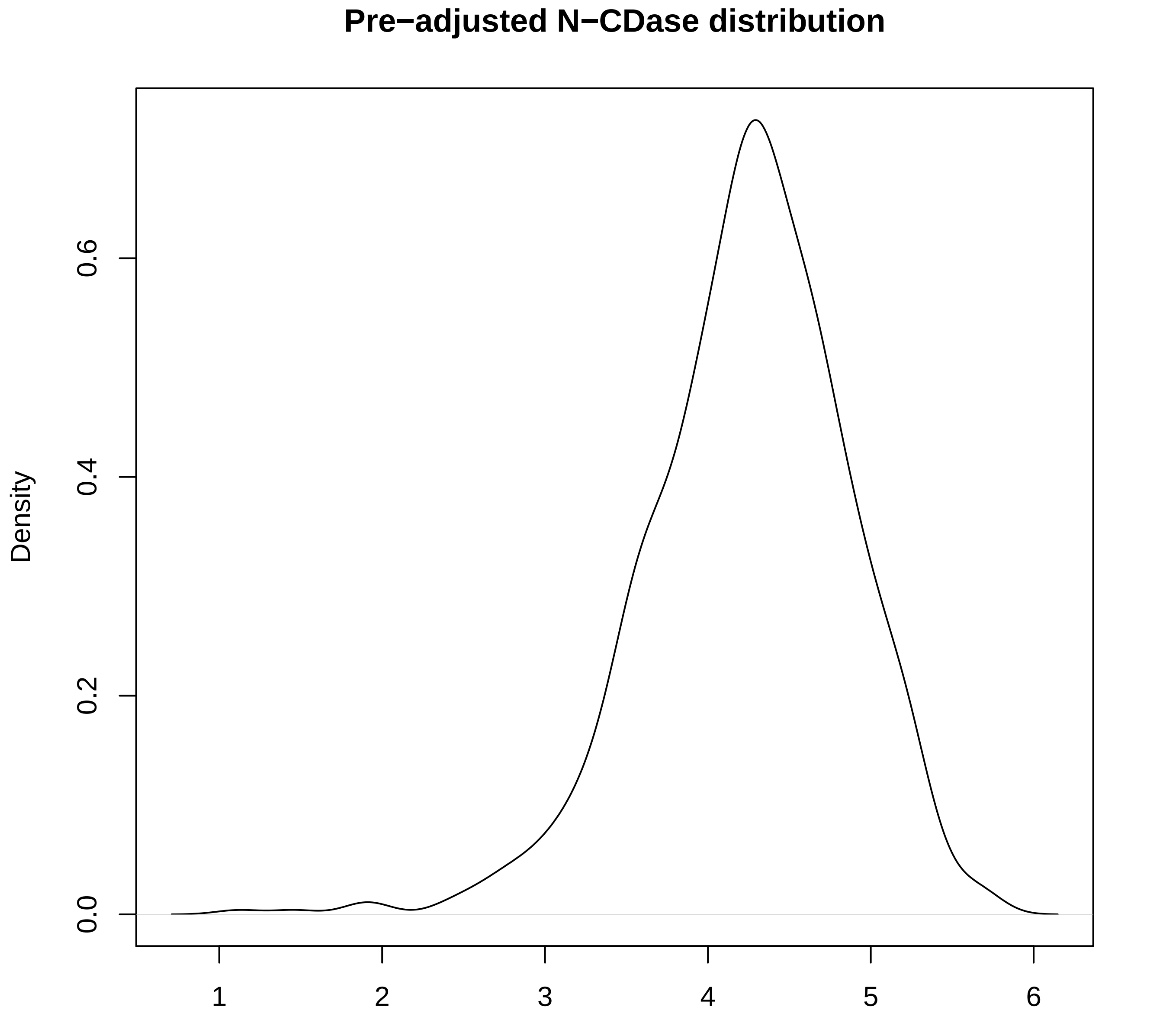
Pre−adjusted N−CDase distribution
Density
0.0
0.2
0.4
0.6
1
2
3
4
5
6

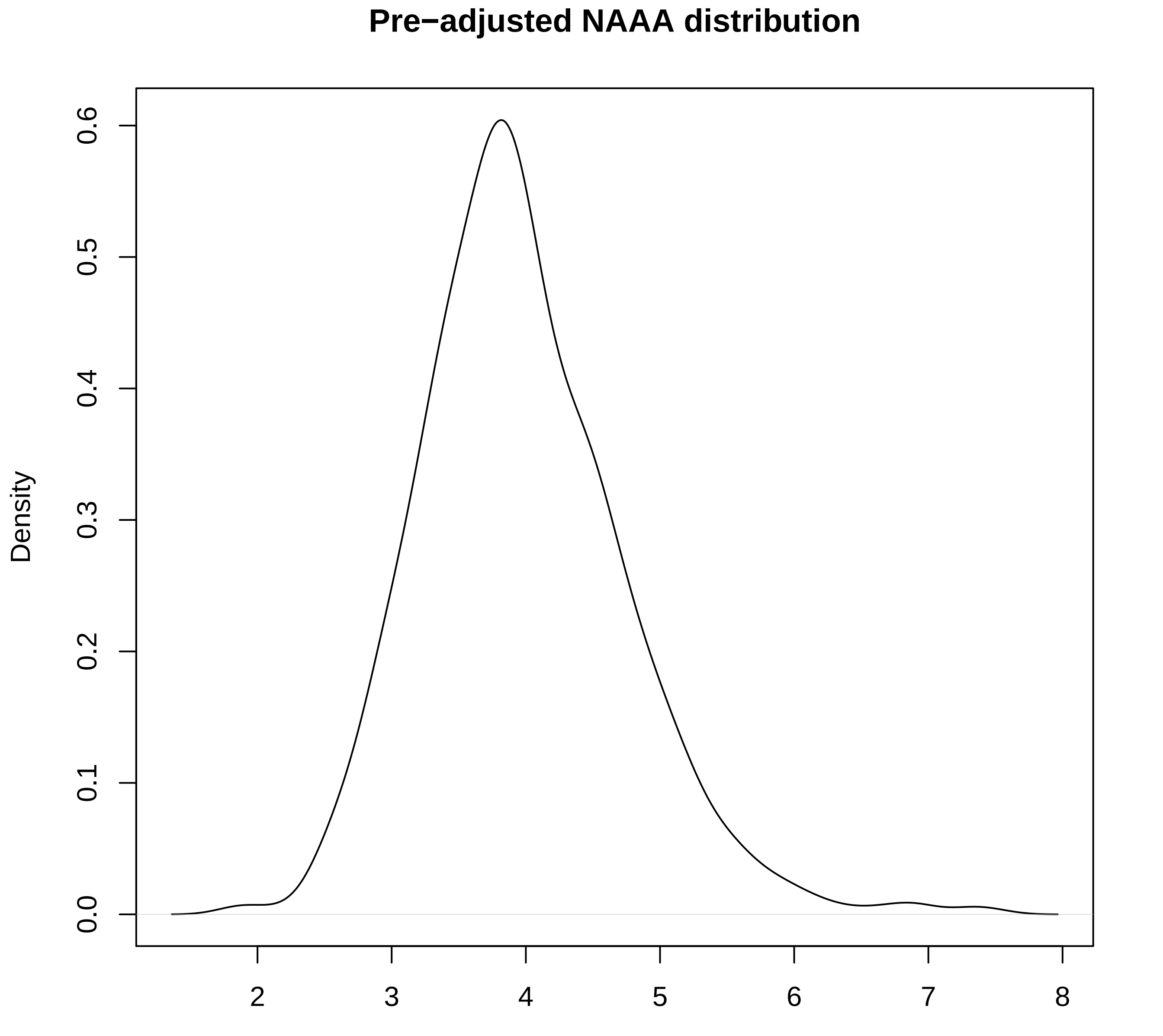
Pre−adjusted NAAA distribution
Density
0.0
0.1
0.2
0.3
0.4
0.5
0.6
2
3
4
5
6
7
8

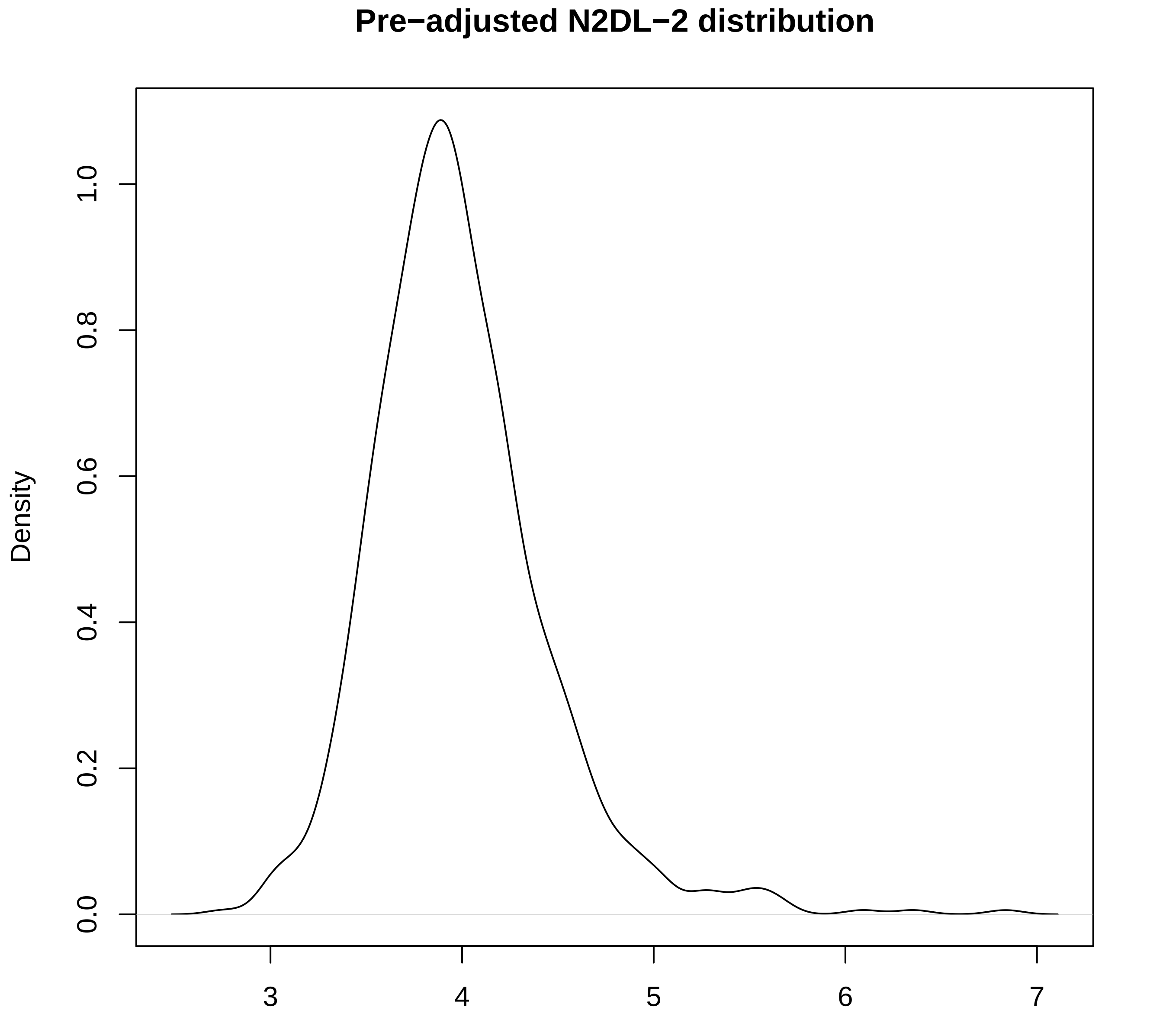
Pre−adjusted N2DL−2 distribution
Density
0.0
0.2
0.4
0.6
0.8
1.0
3
4
5
6
7

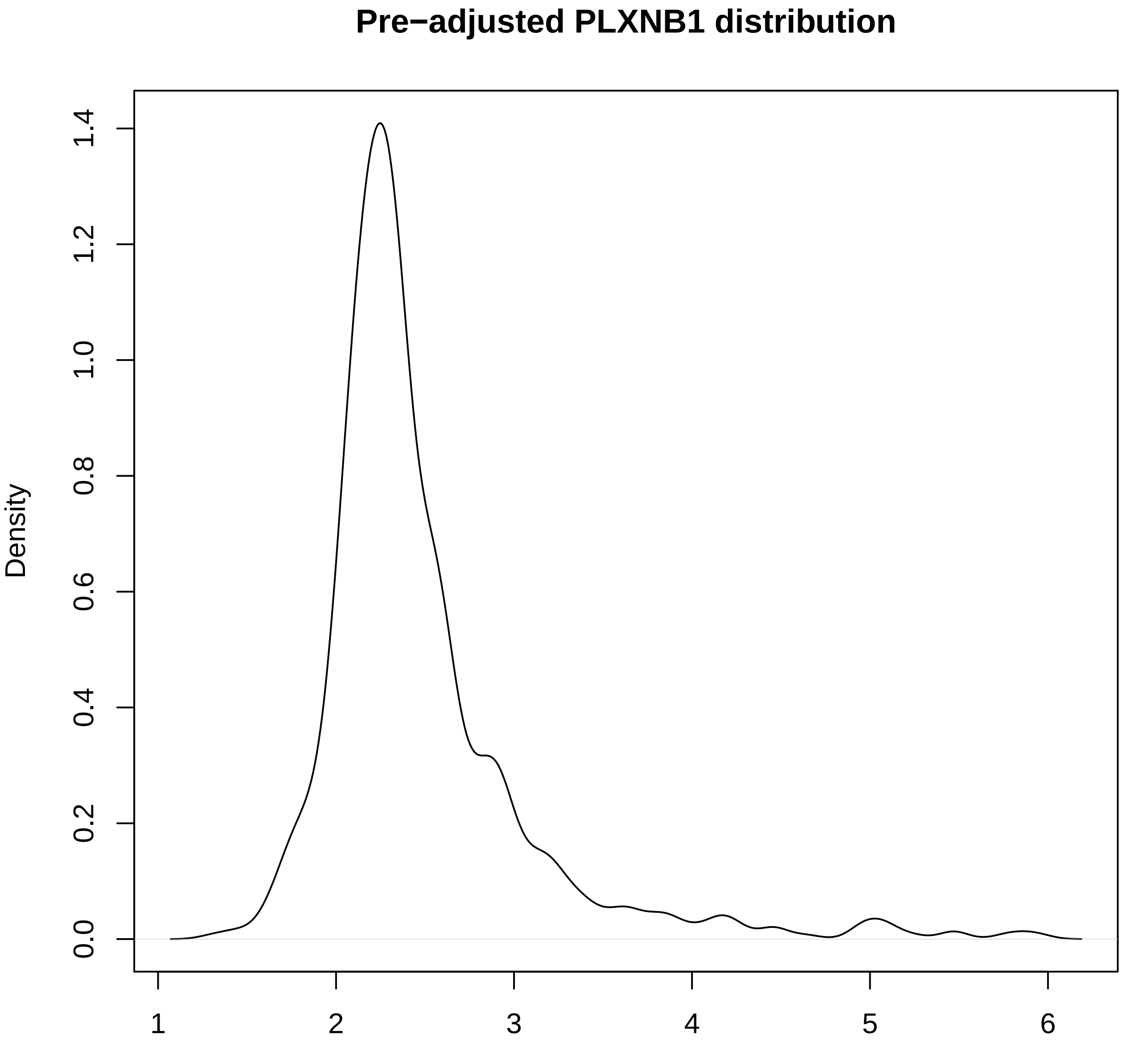
Pre−adjusted PLXNB1 distribution
Density
0.0
0.2
0.4
0.6
0.8
1.0
1.2
1.4
1
2
3
4
5
6

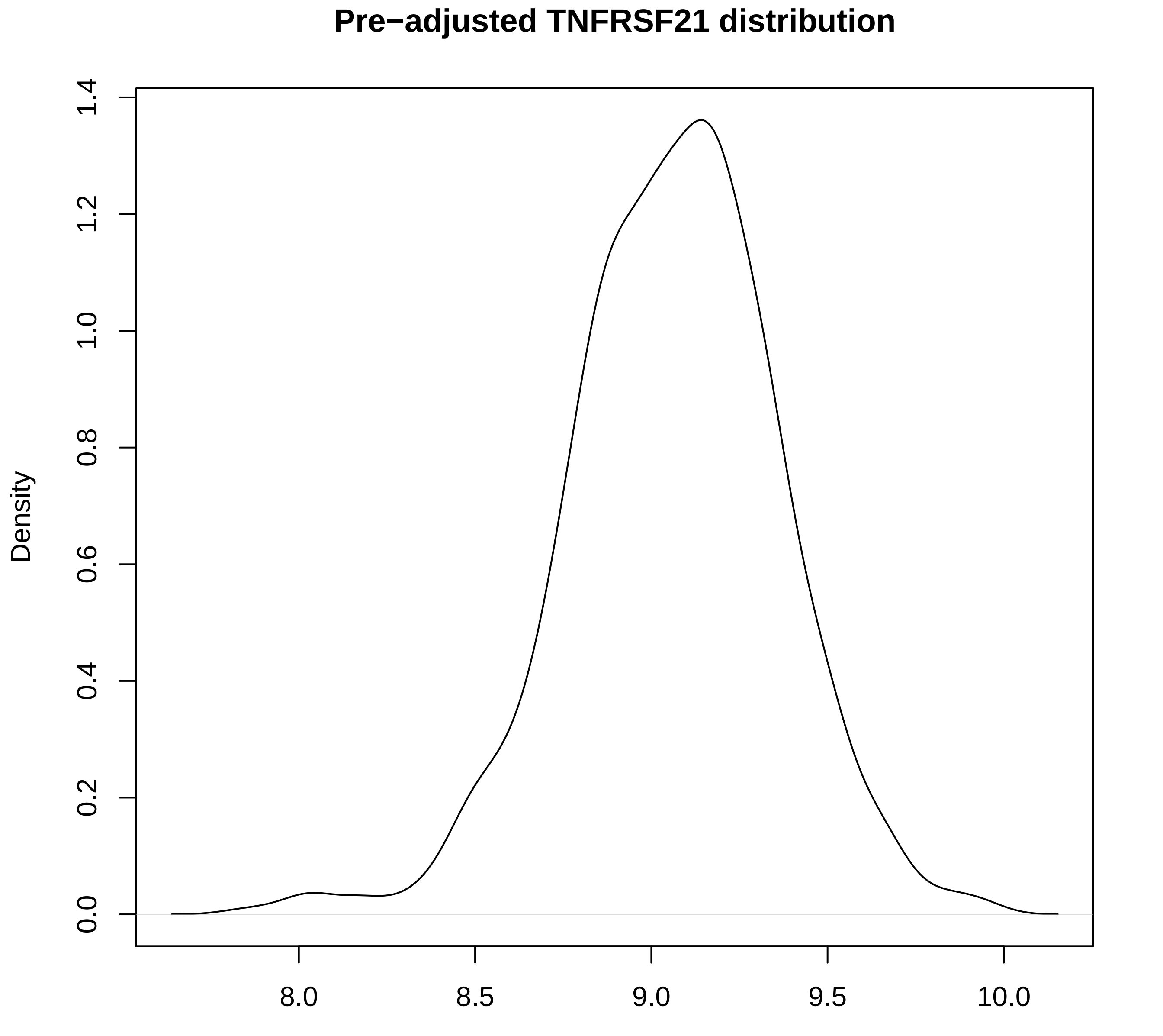

Pre−adjusted TNFRSF21 distribution
Density
0.0
0.2
0.4
0.6
0.8
1.0
1.2
1.4
8.0
8.5
9.0
9.5
10.0

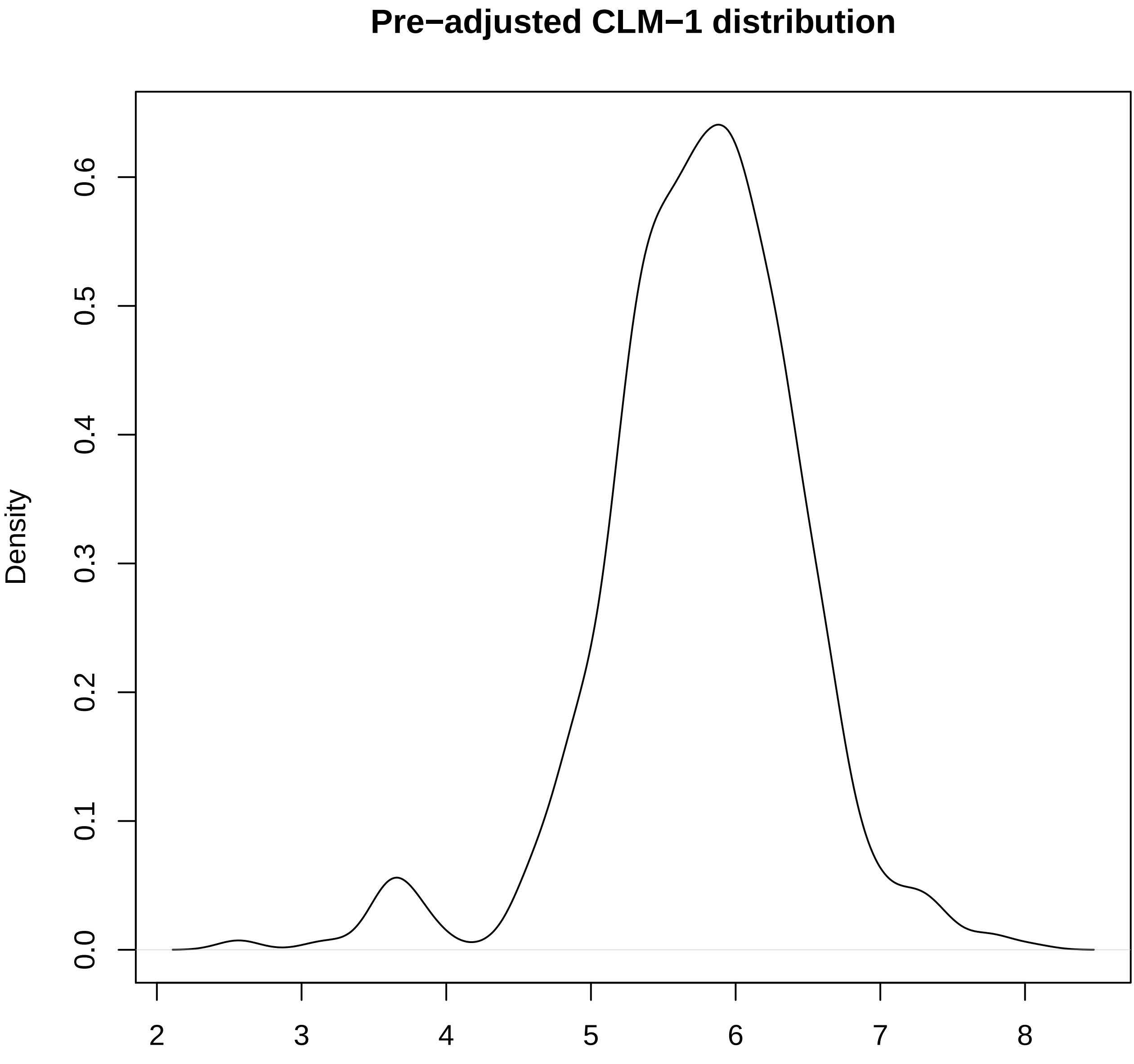
Pre−adjusted CLM−1 distribution
Density
0.0
0.1
0.2
0.3
0.4
0.5
0.6
2
3
4
5
6
7
8

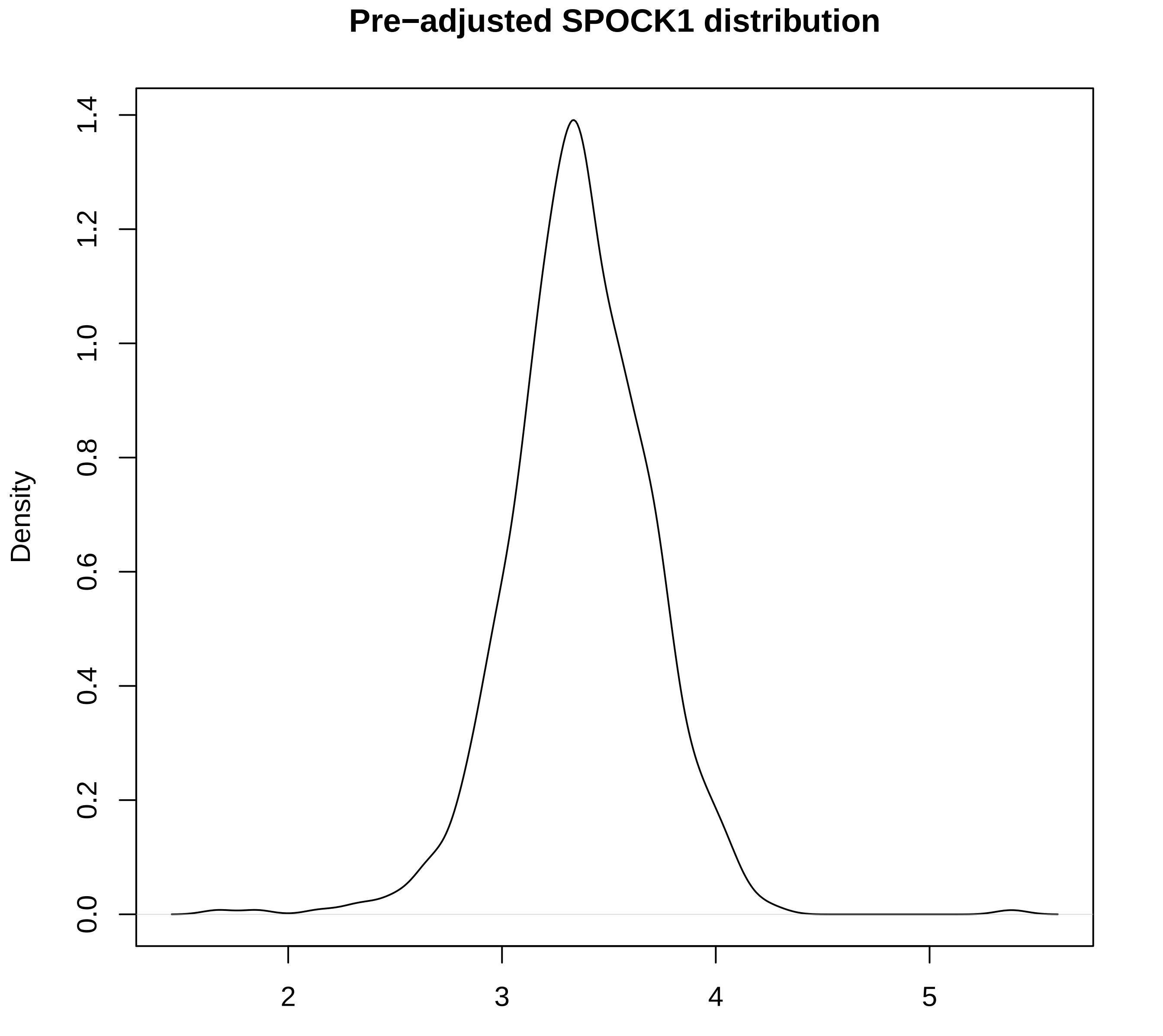
Pre−adjusted SPOCK1 distribution
Density
0.0
0.2
0.4
0.6
0.8
1.0
1.2
1.4
2
3
4
5

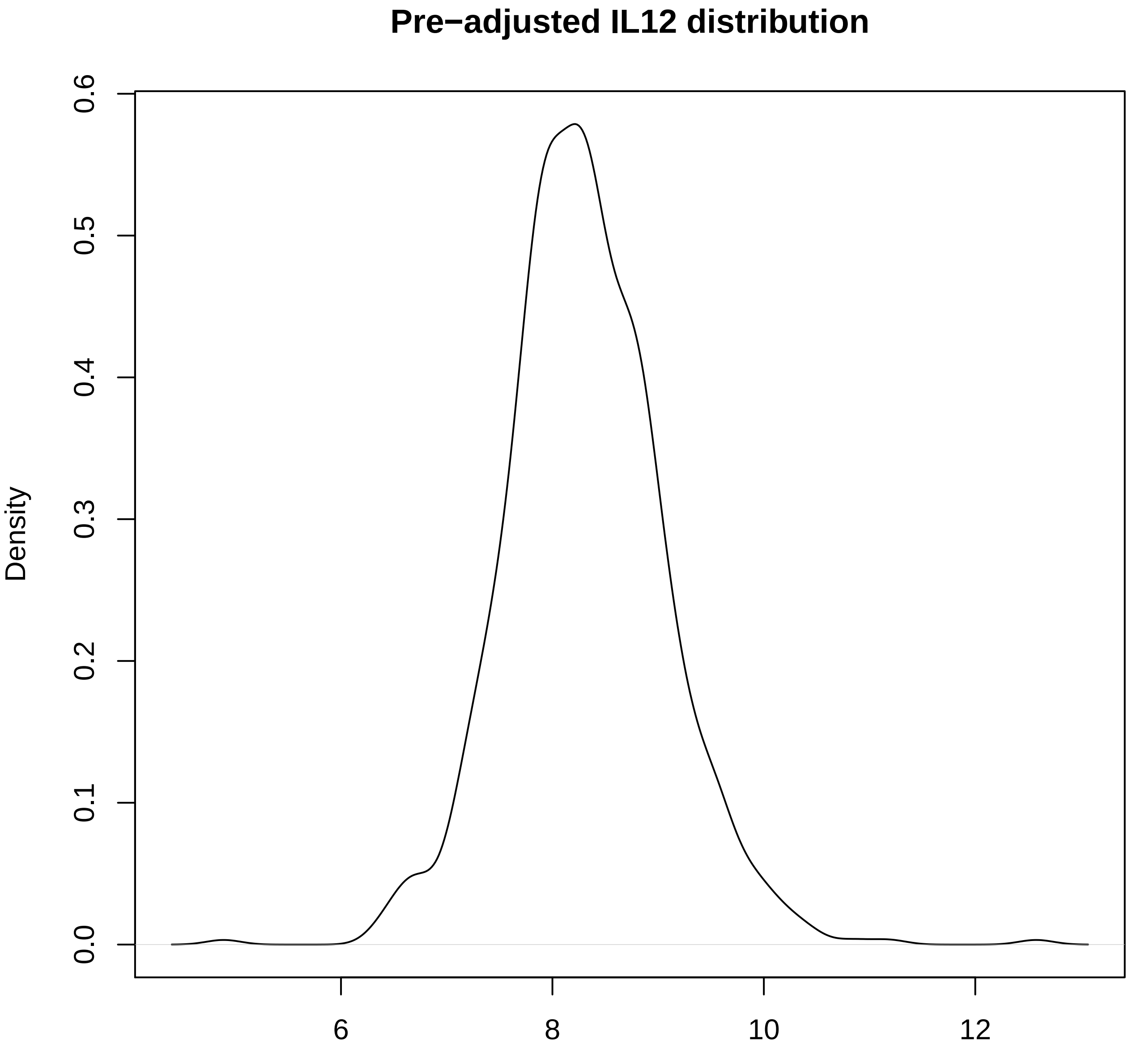

Pre−adjusted IL12 distribution
Density
0.0
0.1
0.2
0.3
0.4
0.5
0.6
6
8
10
12

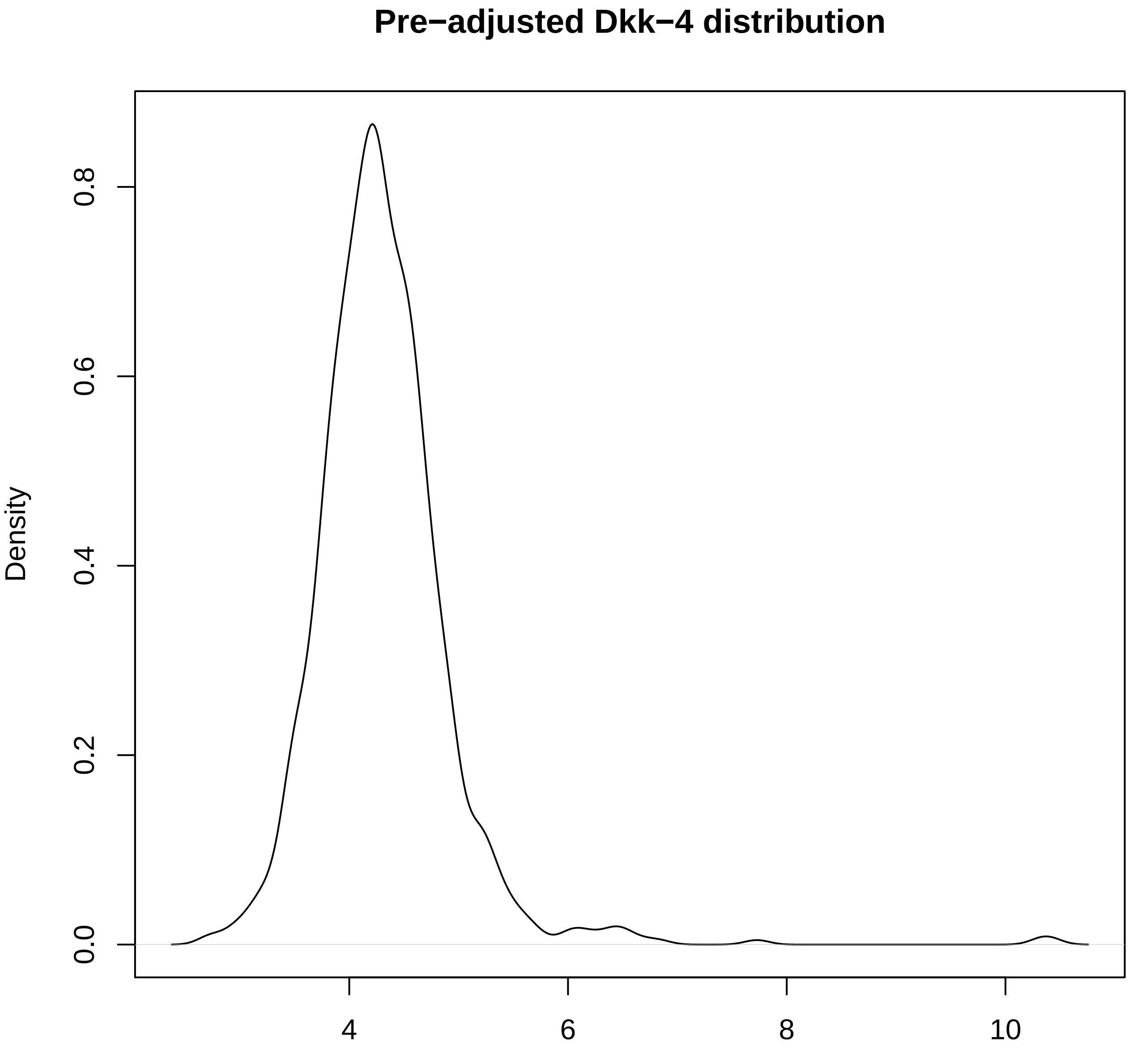
Pre−adjusted Dkk−4 distribution
Density
0.0
0.2
0.4
0.6
0.8
4
6
8
10

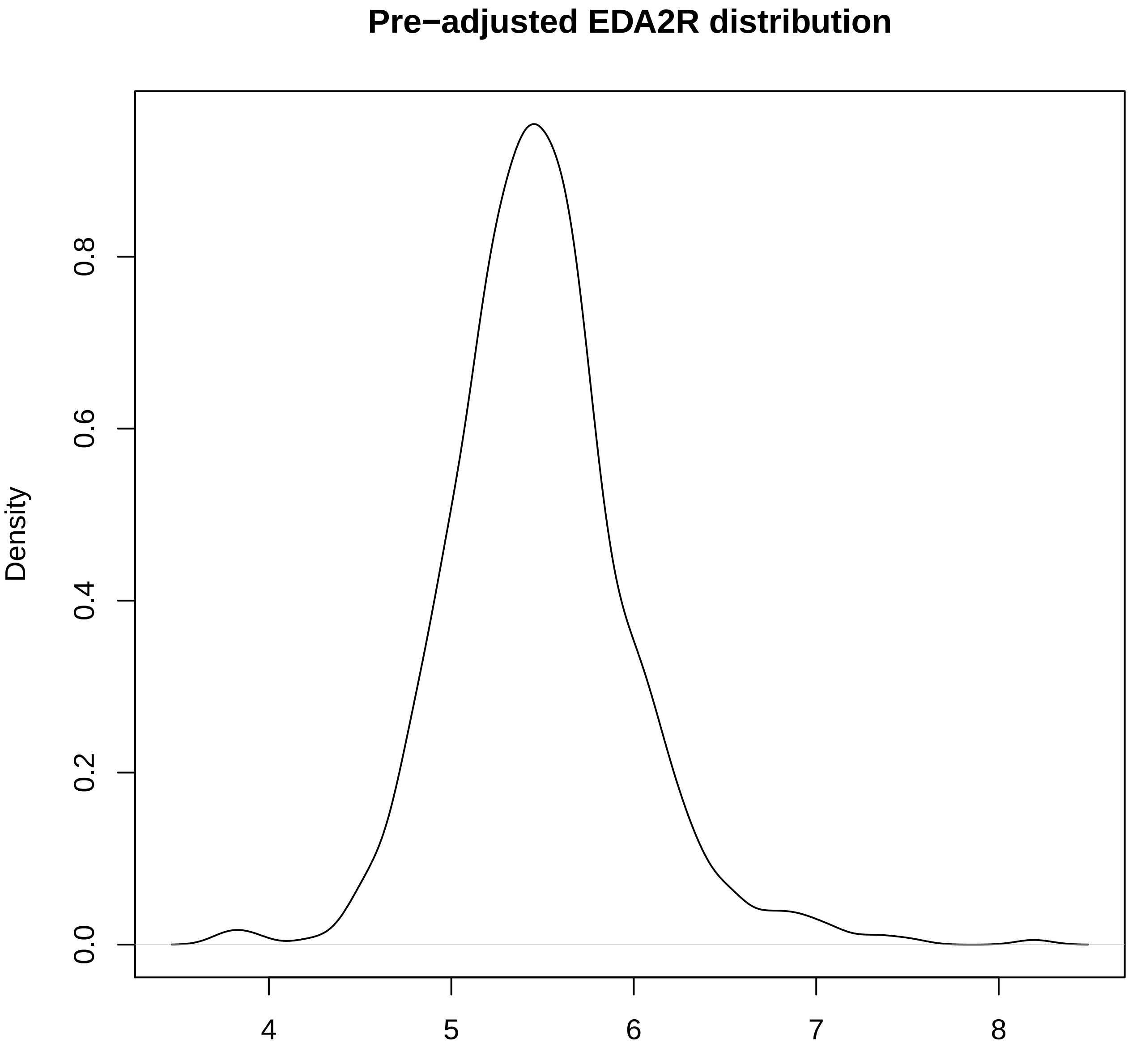
Pre−adjusted EDA2R distribution
Density
0.0
0.2
0.4
0.6
0.8
4
5
6
7
8

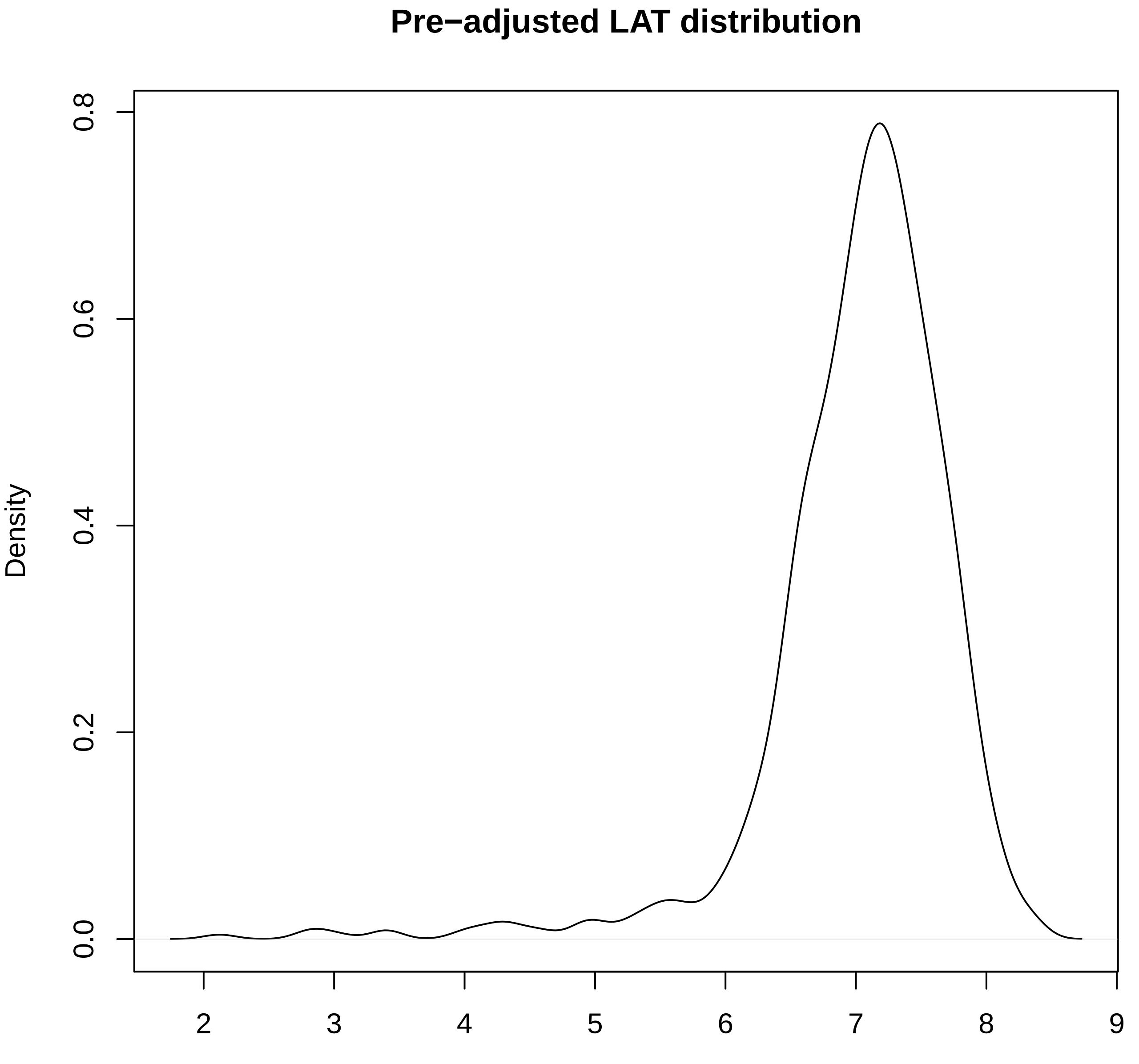
Pre−adjusted LAT distribution
Density
0.0
0.2
0.4
0.6
0.8
2
3
4
5
6
7
8
9

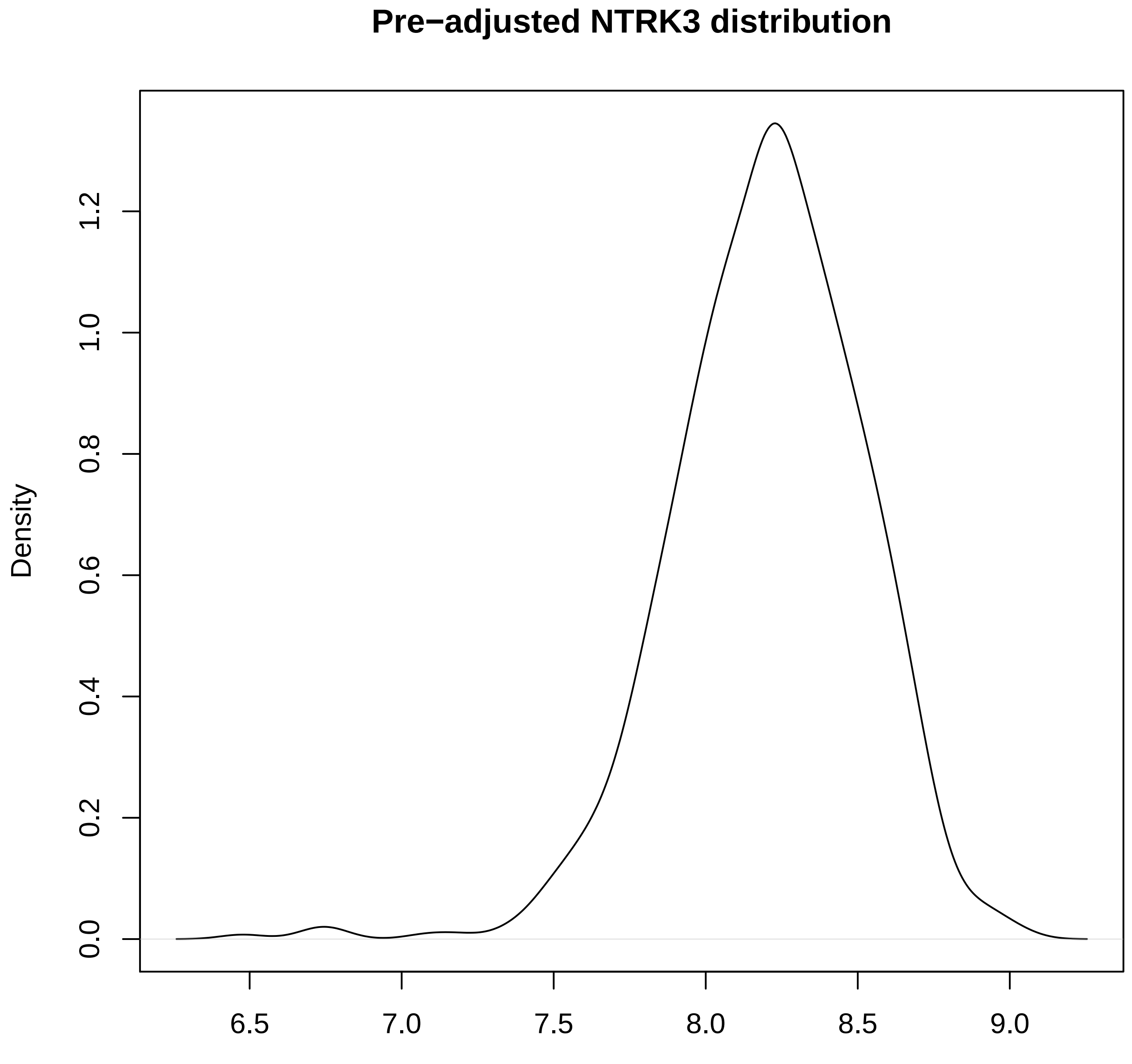
Pre−adjusted NTRK3 distribution
Density
0.0
0.2
0.4
0.6
0.8
1.0
1.2
6.5
7.0
7.5
8.0
8.5
9.0

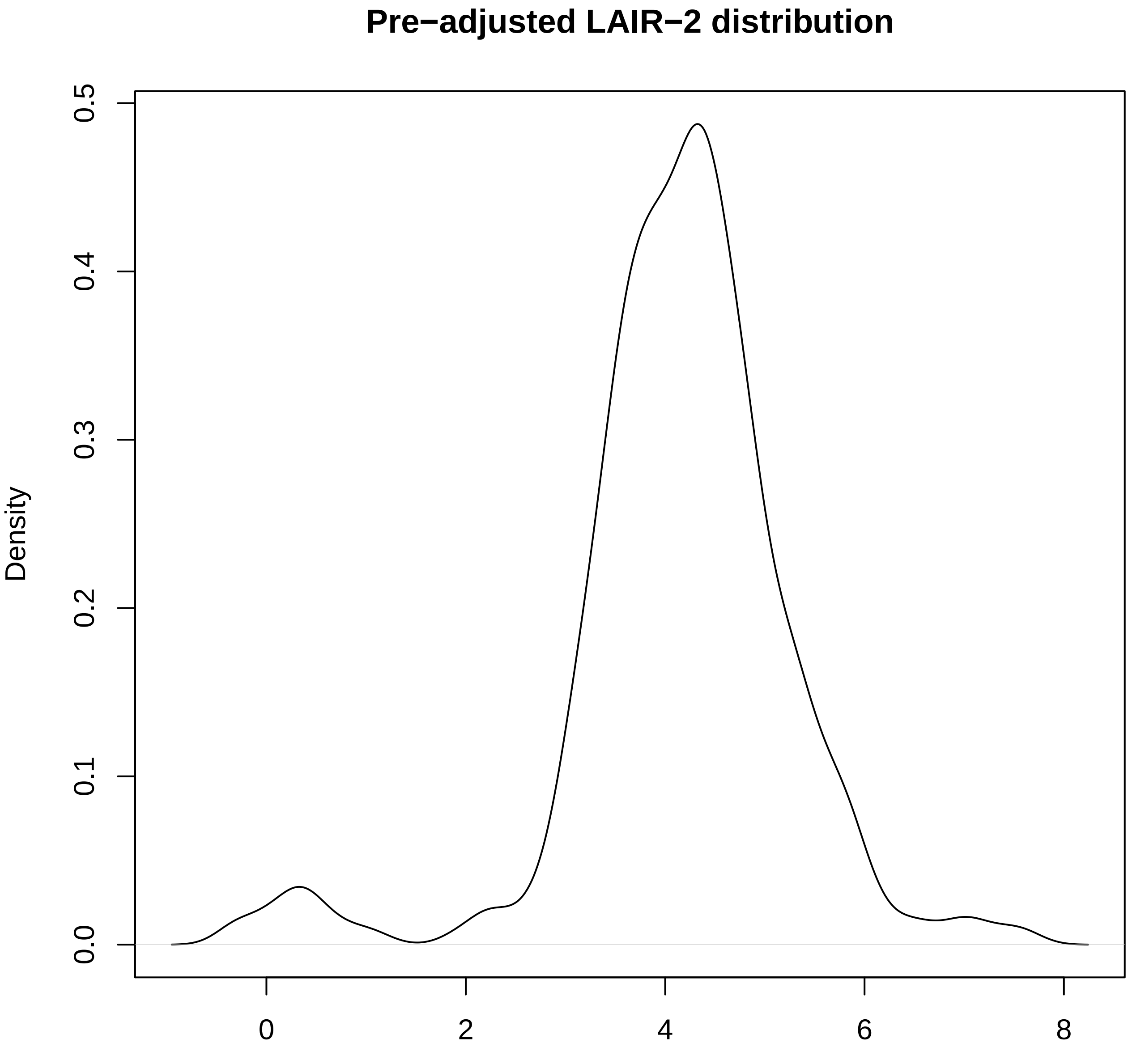

Pre−adjusted LAIR−2 distribution
Density
0.0
0.1
0.2
0.3
0.4
0.5
0
2
4
6
8

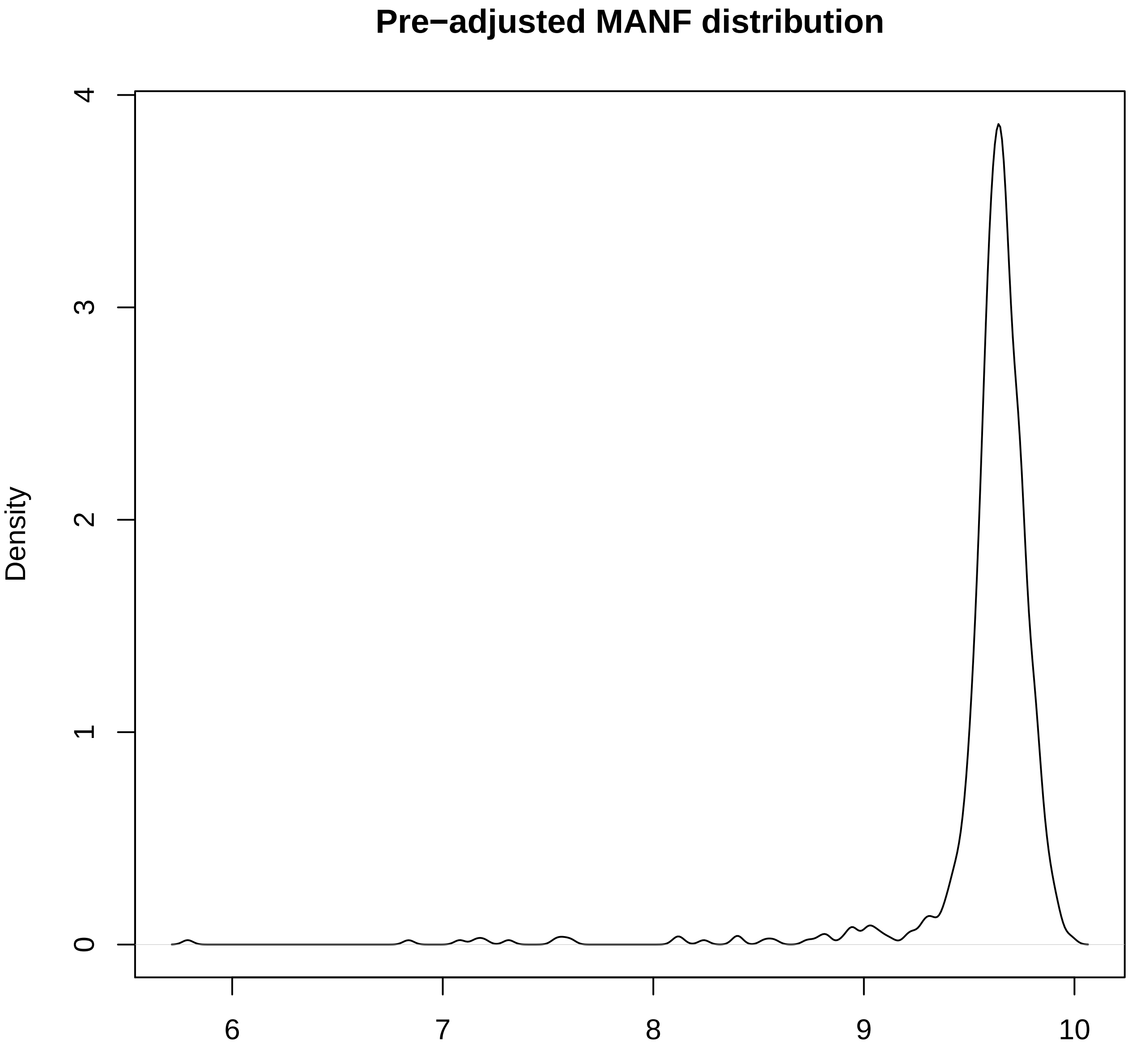
Pre−adjusted MANF distribution
Density
0
1
2
3
4
6
7
8
9
10

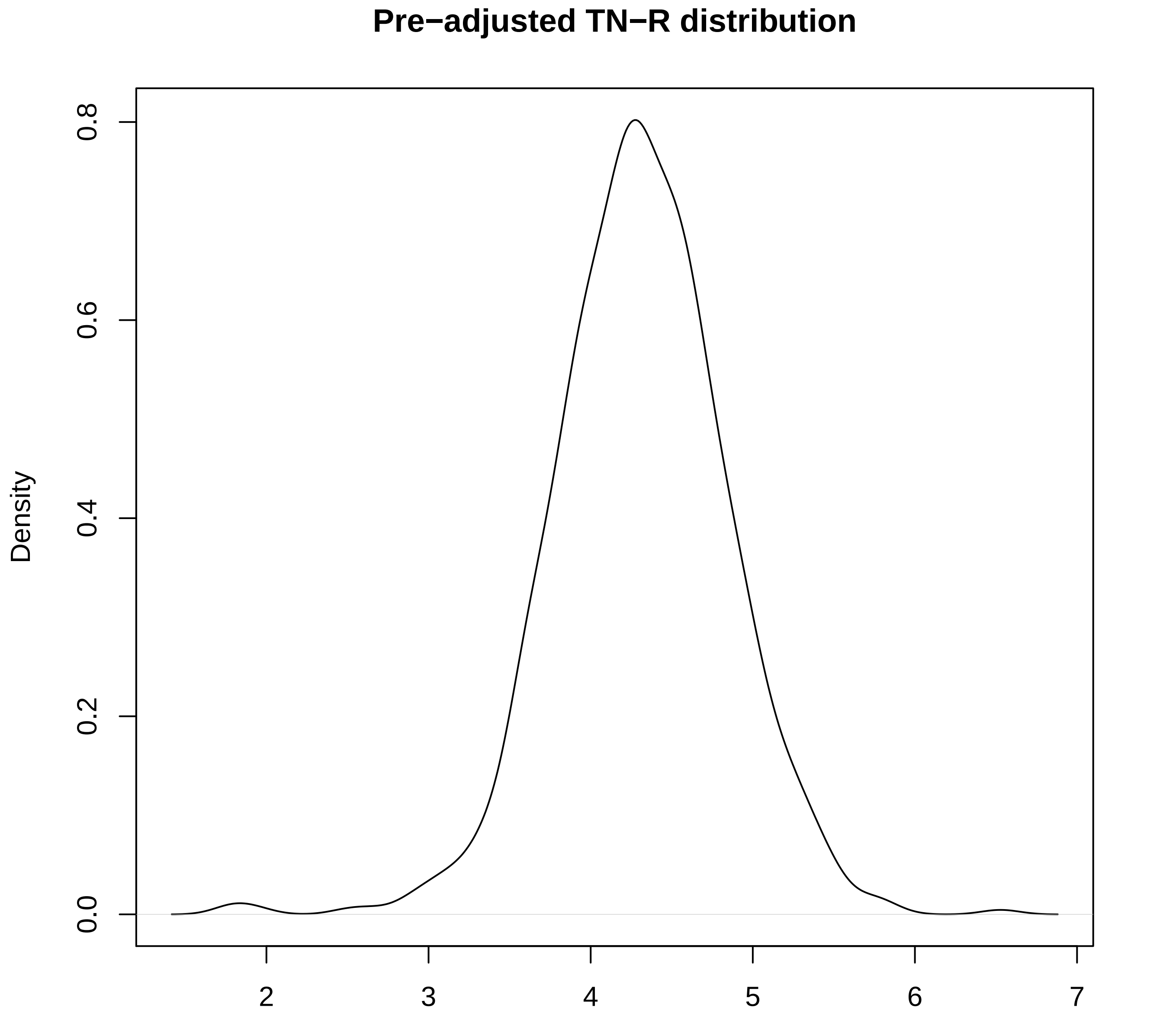
Pre−adjusted TN−R distribution
Density
0.0
0.2
0.4
0.6
0.8
2
3
4
5
6
7

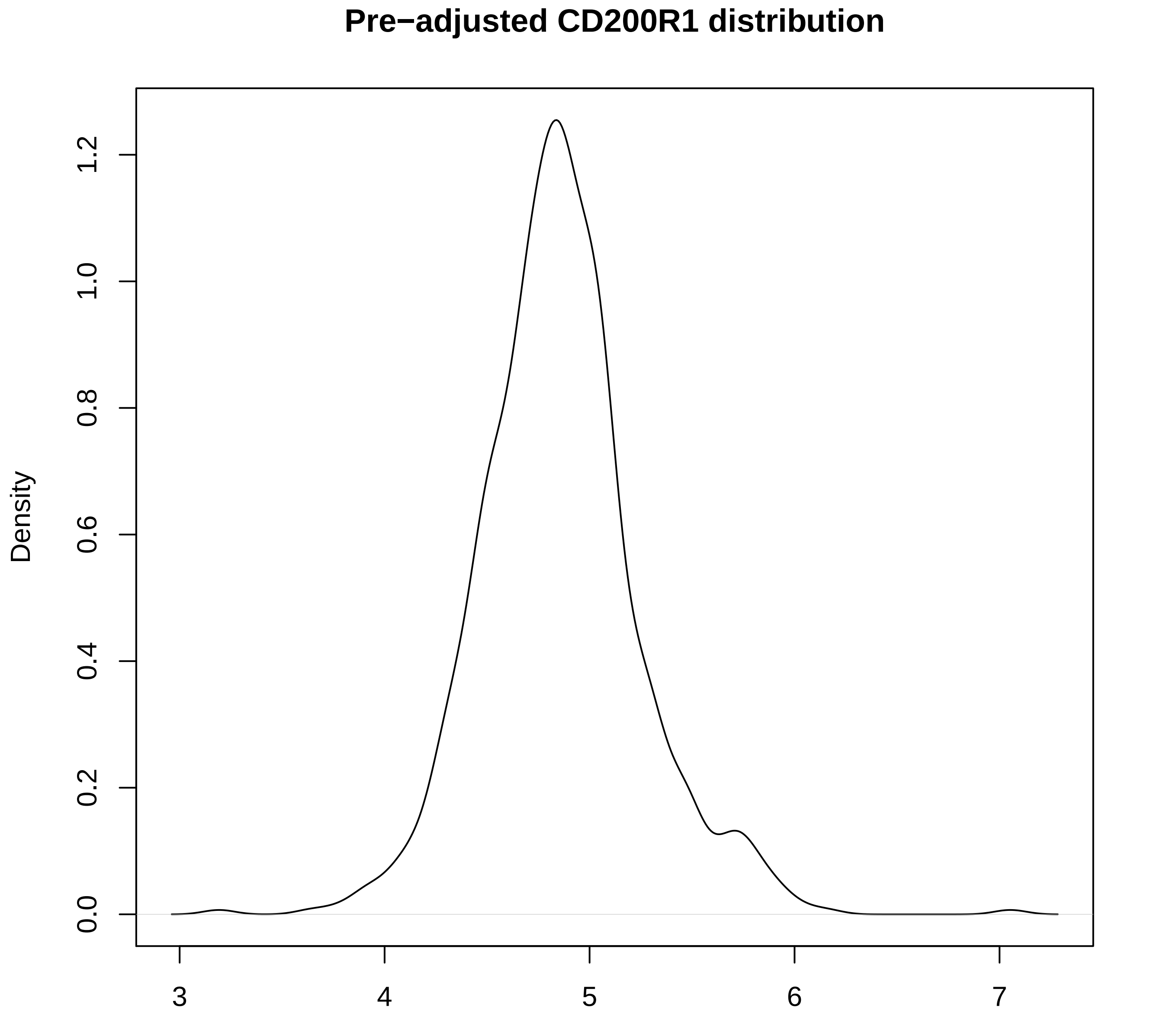
Pre−adjusted CD200R1 distribution
Density
0.0
0.2
0.4
0.6
0.8
1.0
1.2
3
4
5
6
7

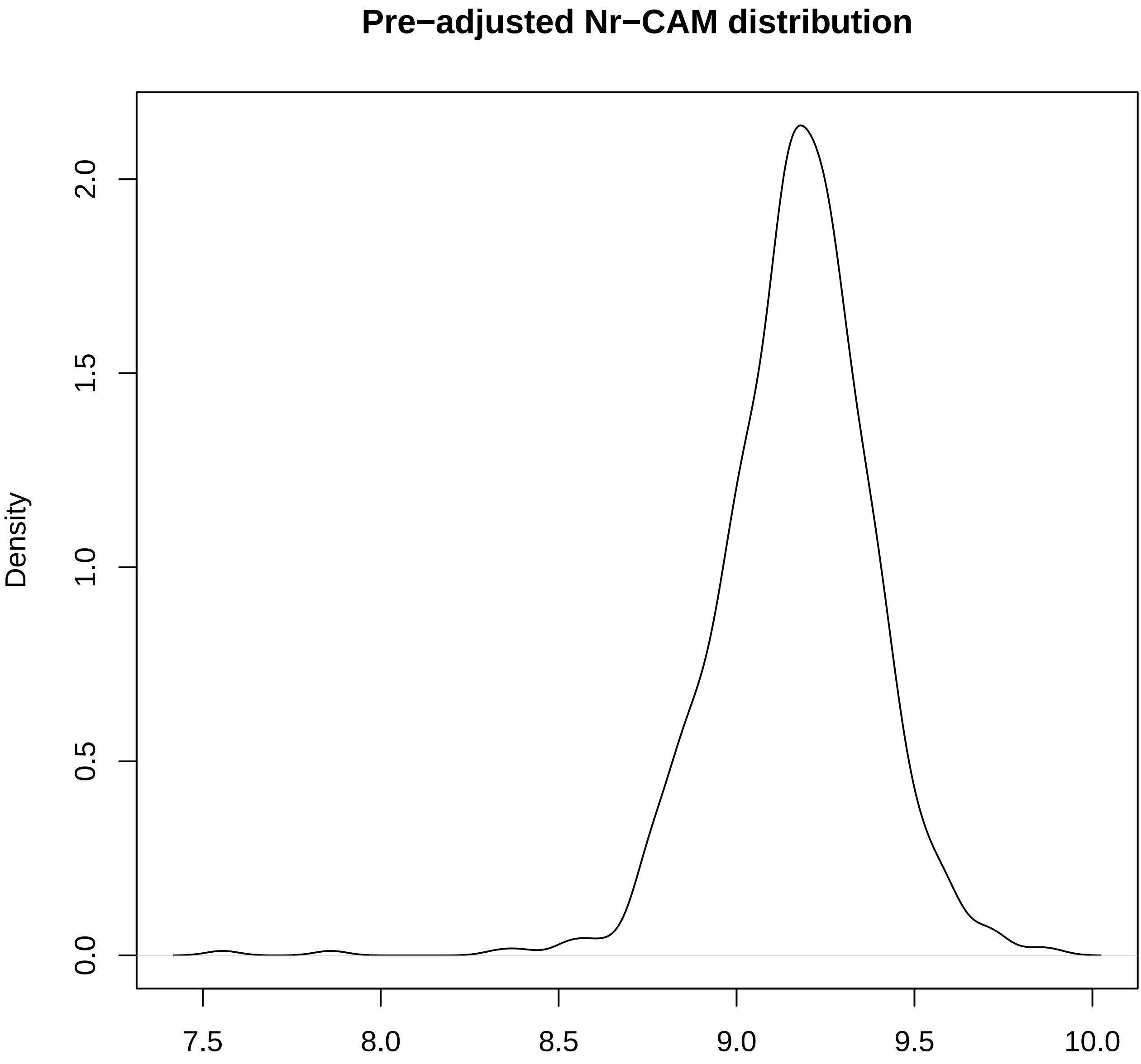
Pre−adjusted Nr−CAM distribution
Density
0.0
0.5
1.0
1.5
2.0
7.5
8.0
8.5
9.0
9.5
10.0

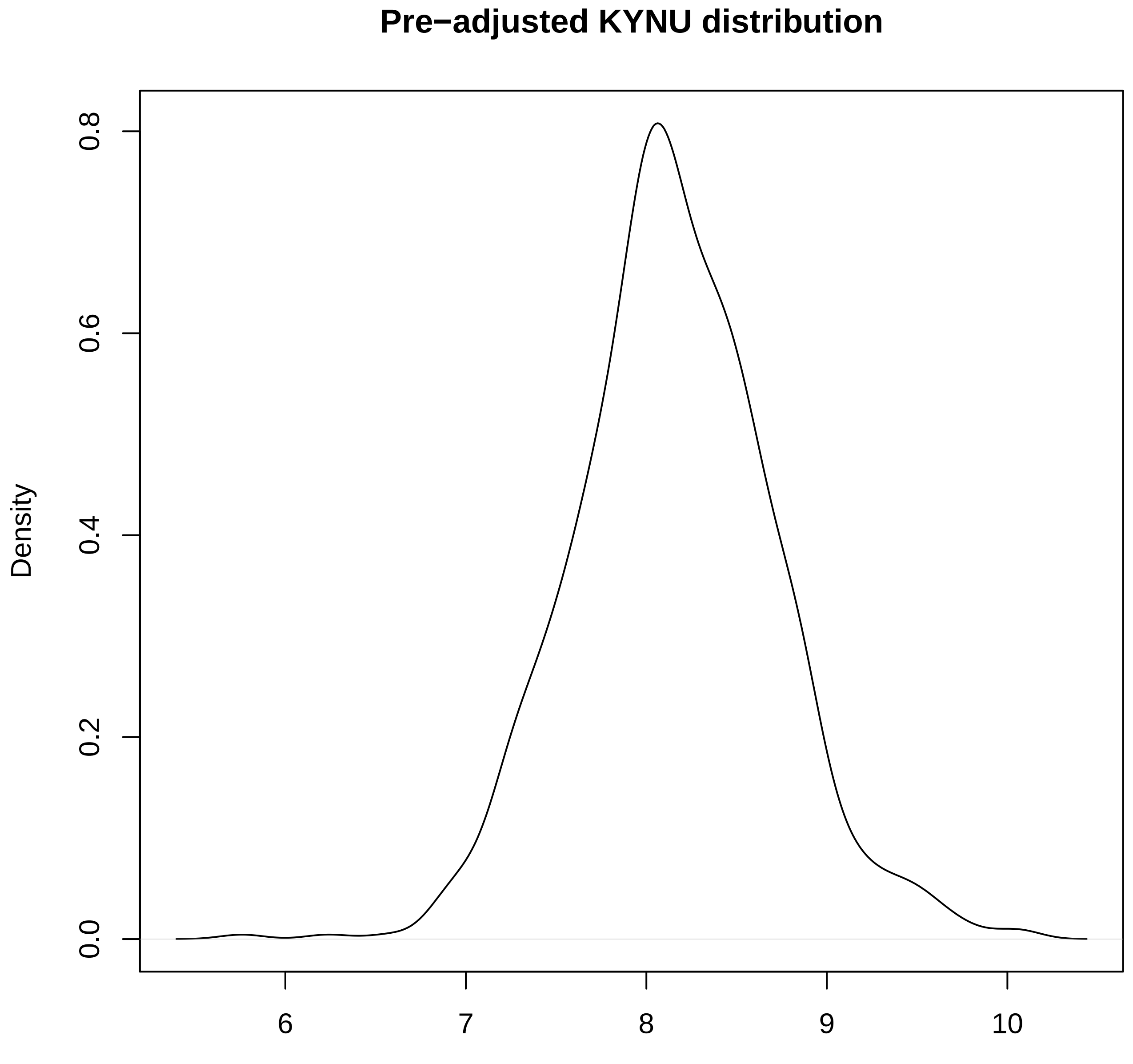
Pre−adjusted KYNU distribution
Density
0.0
0.2
0.4
0.6
0.8
6
7
8
9
10
