# Supplementary material for: Genome and epigenome wide studies of neurological protein biomarkers in the Lothian Birth Cohort 1936
Source: Nat Commun. 2019 Jul 18;10:3160. doi: 10.1038/s41467-019-11177-x (PMC6639385; doi:10.1038/s41467-019-11177-x)
Supplement: Supplementary file 12 — Supplementary Dataset 11 [file 41467_2019_11177_MOESM12_ESM.pdf]

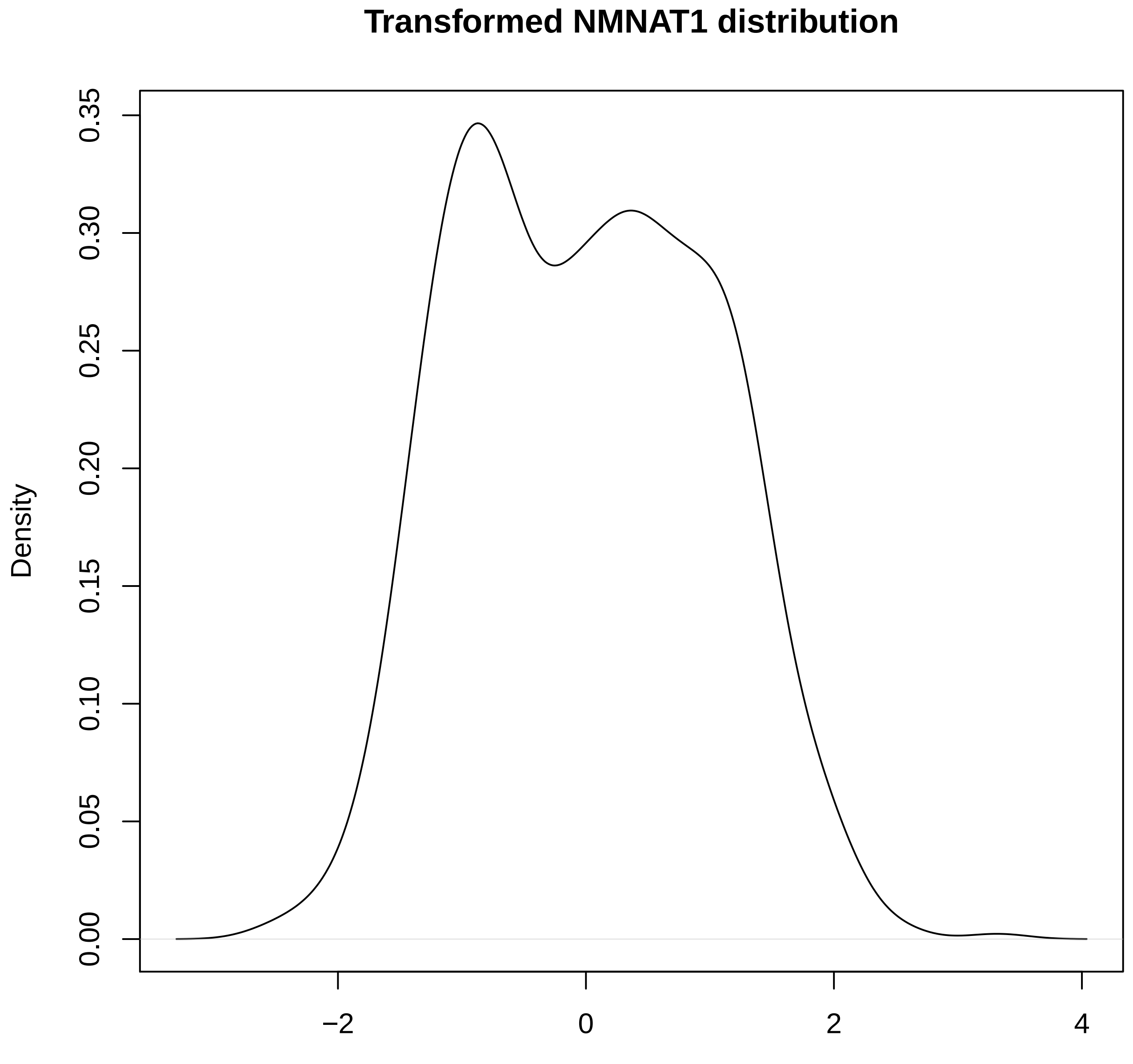
Transformed NMNAT1 distribution
Density
0.00
0.05
0.10
0.15
0.20
0.25
0.30
0.35
−2
0
2
4

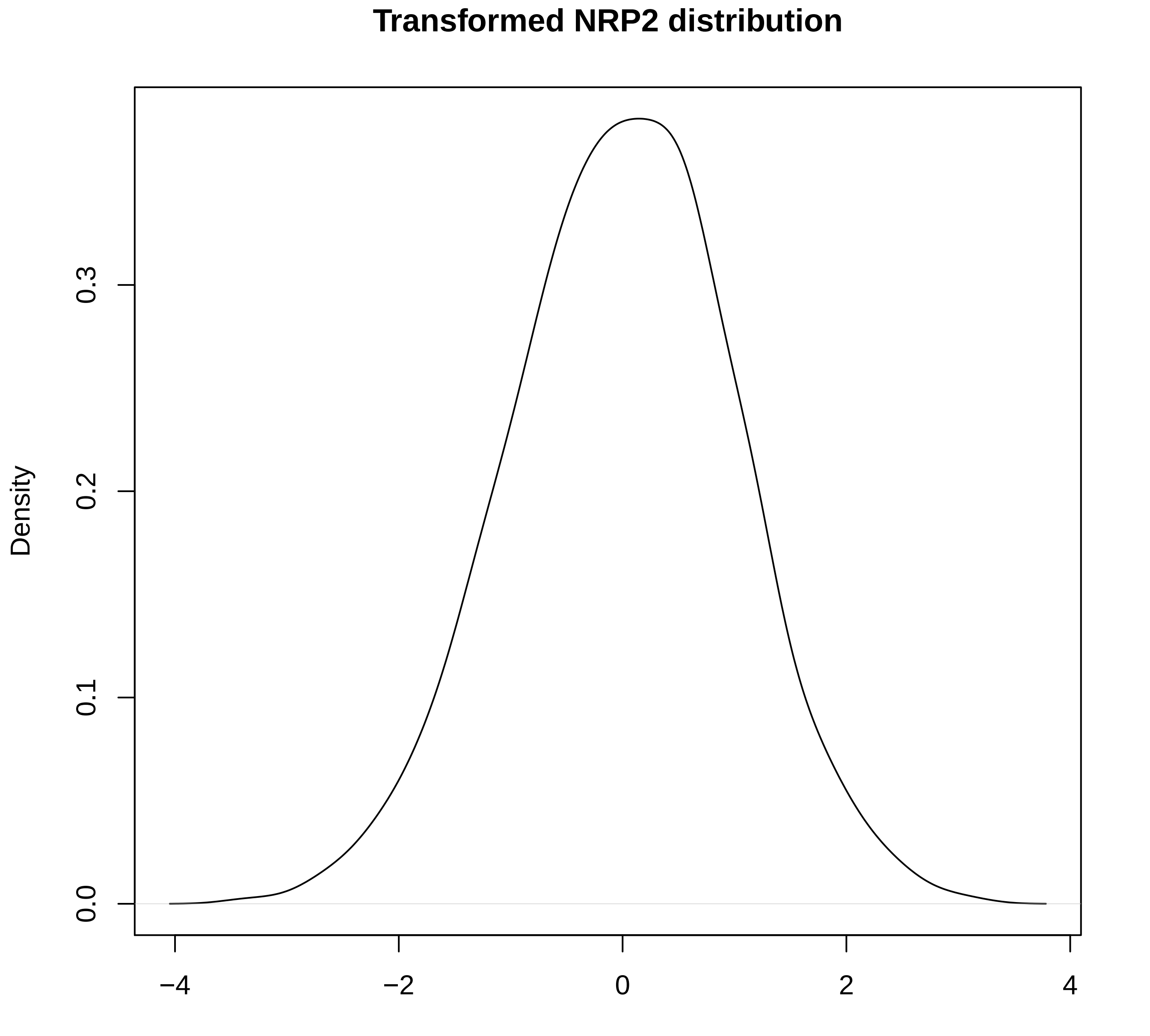

Transformed NRP2 distribution
Density
0.0
0.1
0.2
0.3
−4
−2
0
2
4

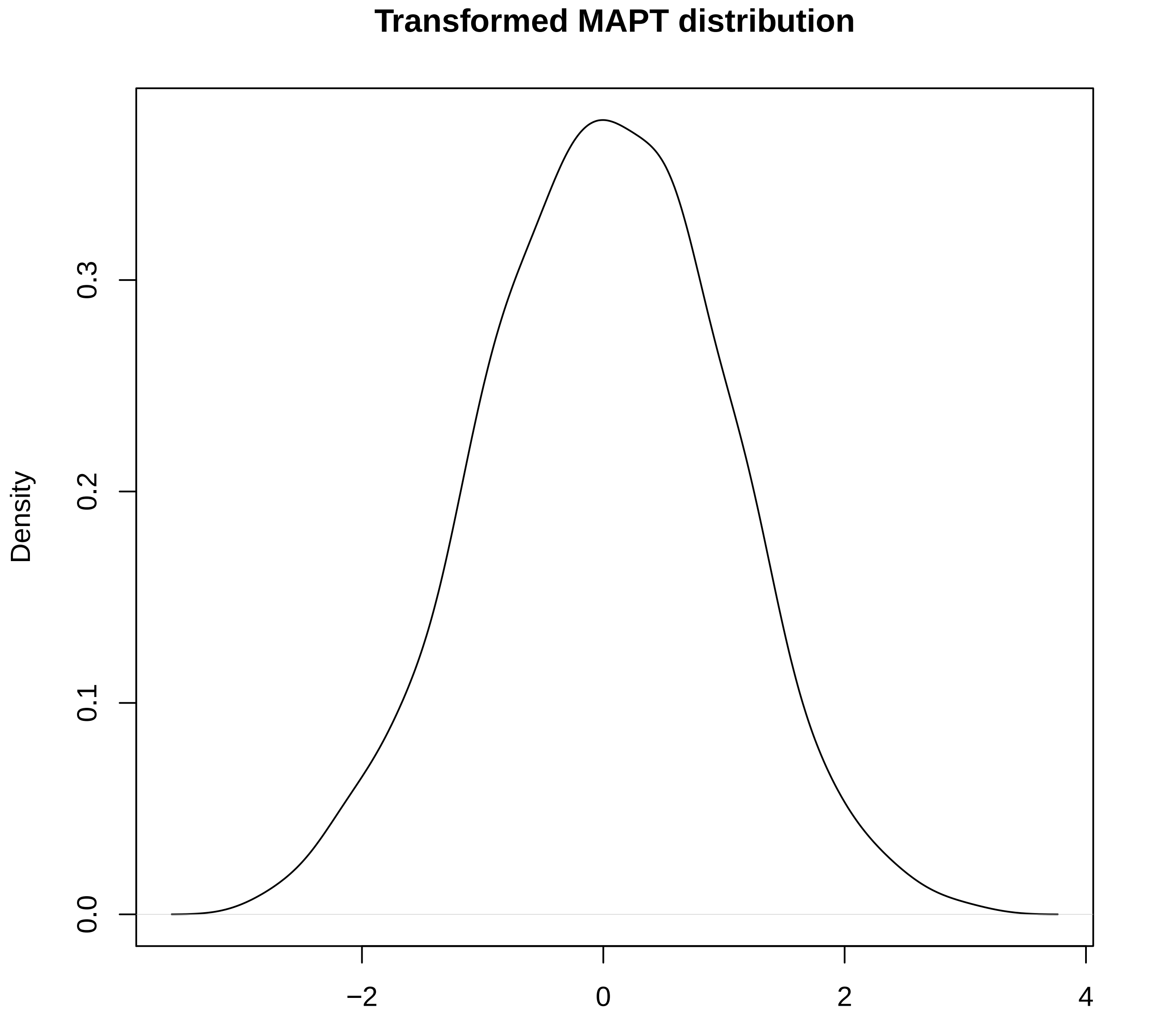
Transformed MAPT distribution
Density
0.0
0.1
0.2
0.3
−2
0
2
4

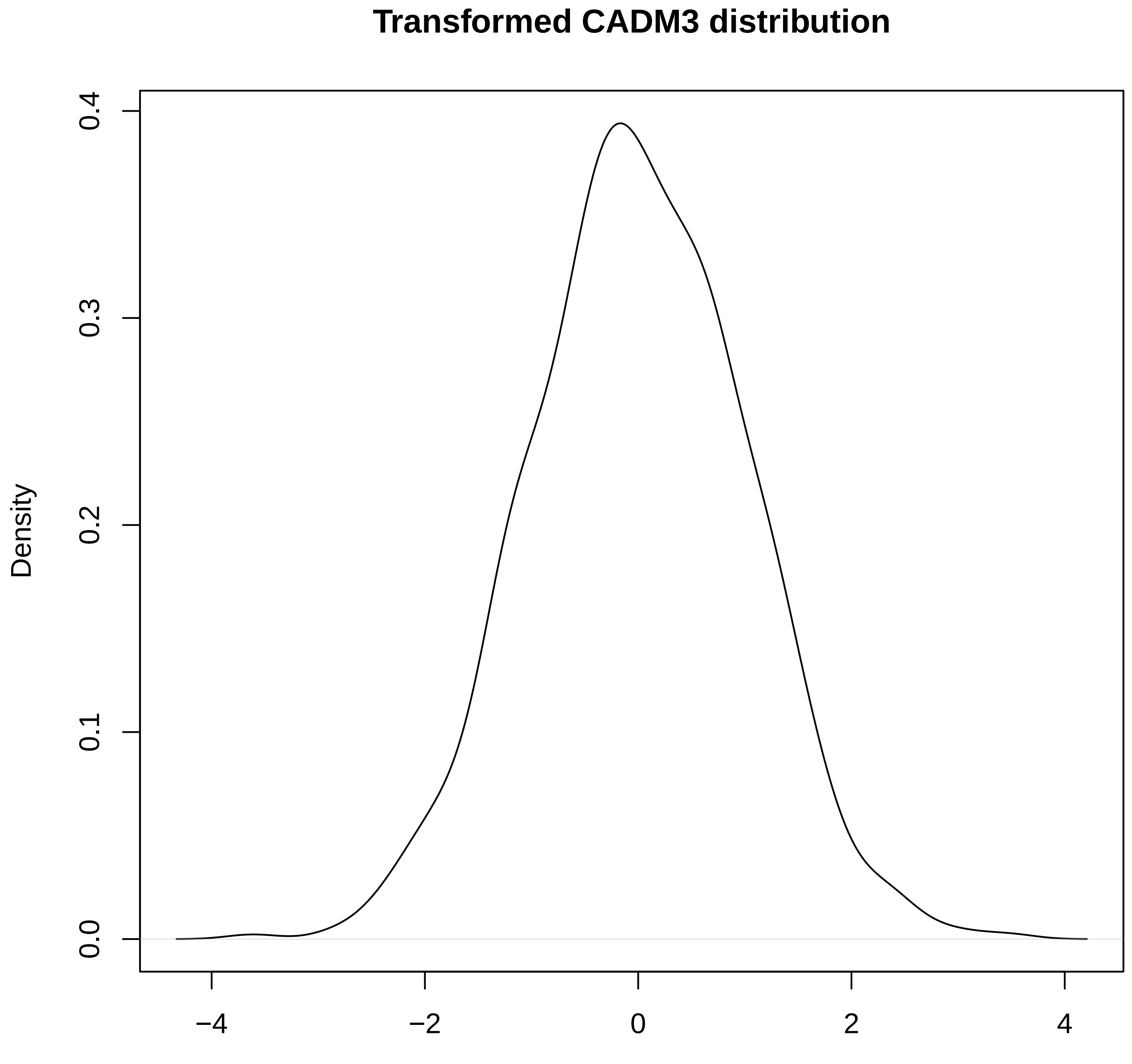
Transformed CADM3 distribution
Density
0.0
0.1
0.2
0.3
0.4
−4
−2
0
2
4

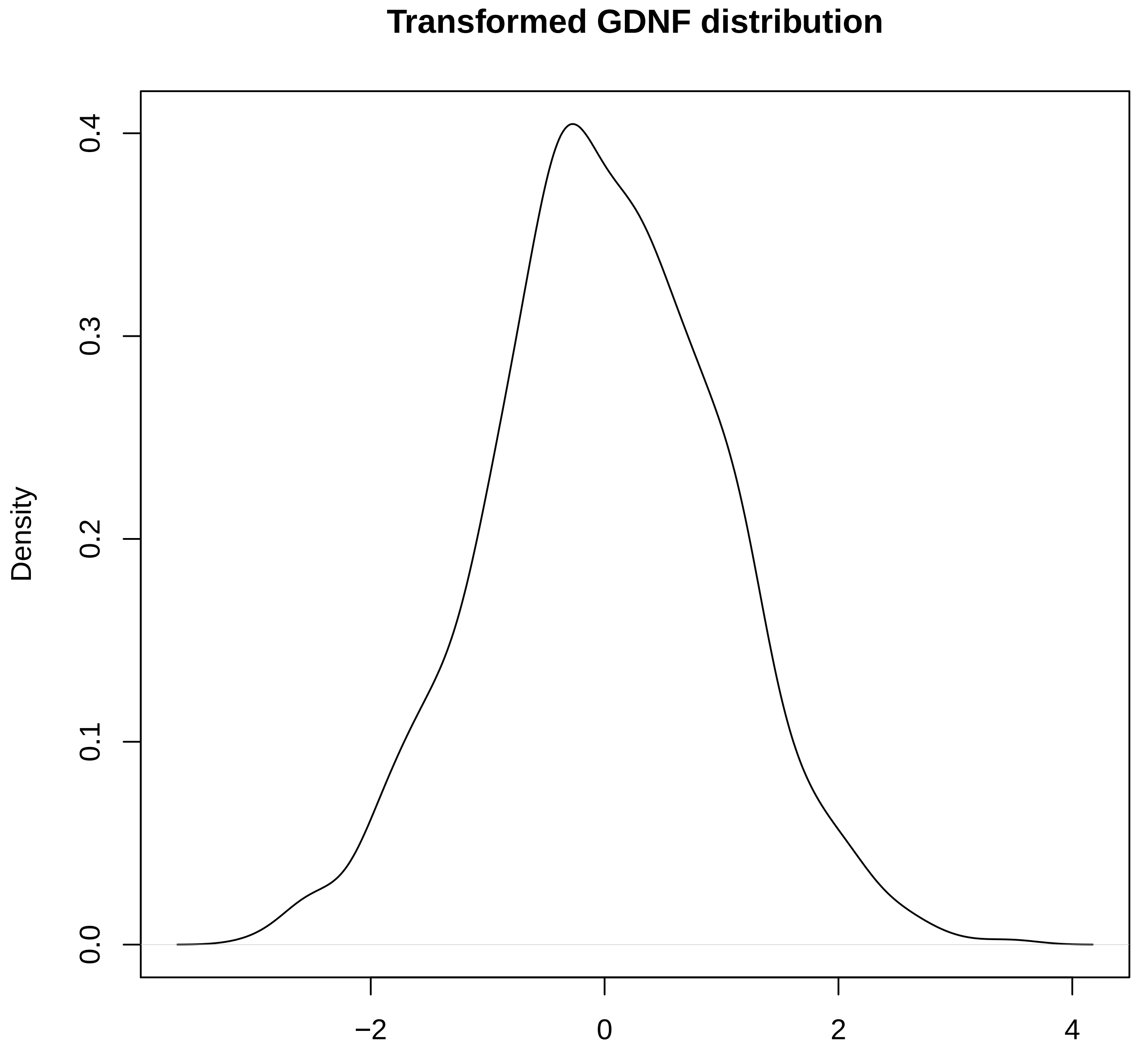
Transformed GDNF distribution
Density
0.0
0.1
0.2
0.3
0.4
−2
0
2
4

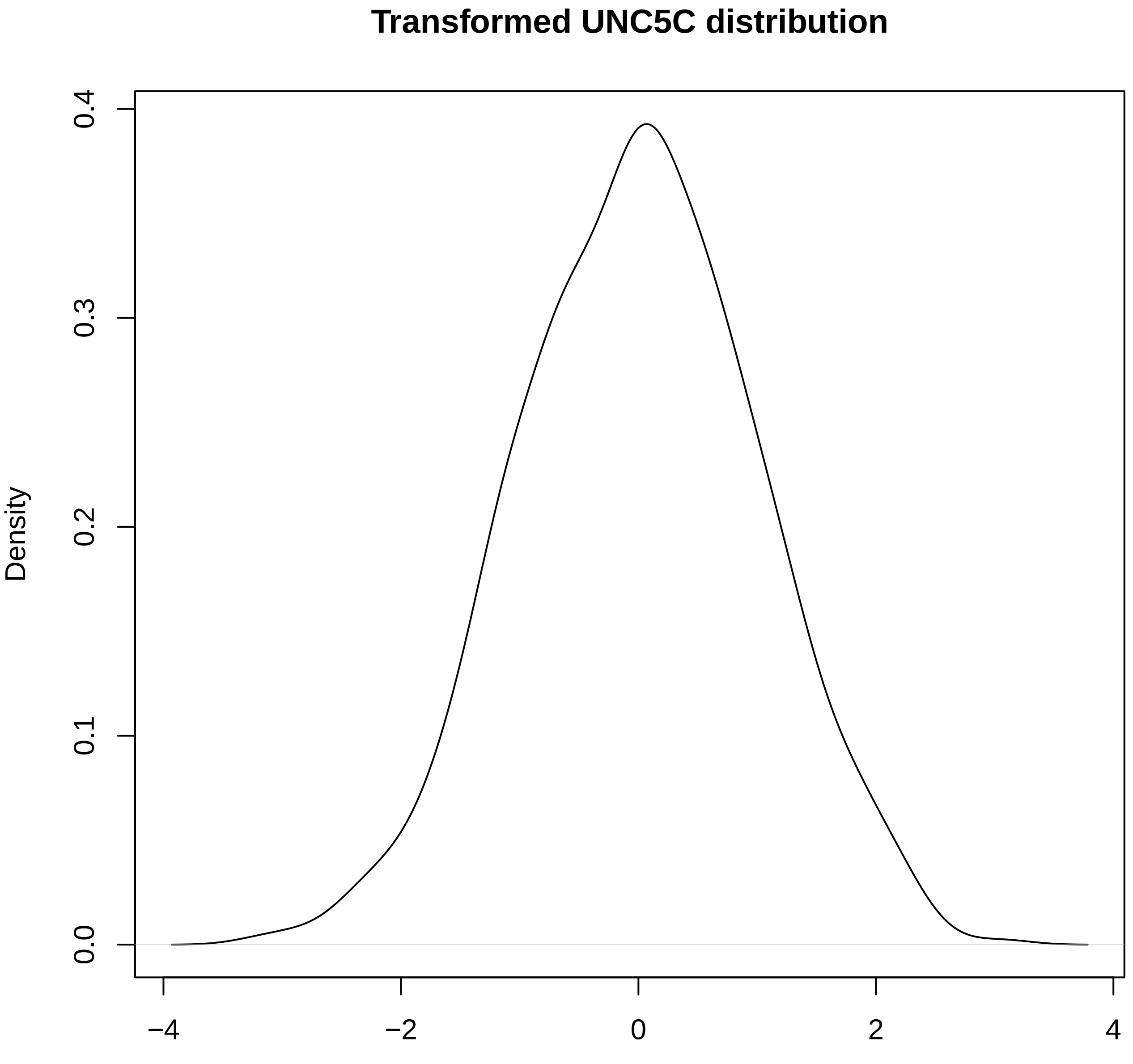

Transformed UNC5C distribution
Density
0.0
0.1
0.2
0.3
0.4
−4
−2
0
2
4

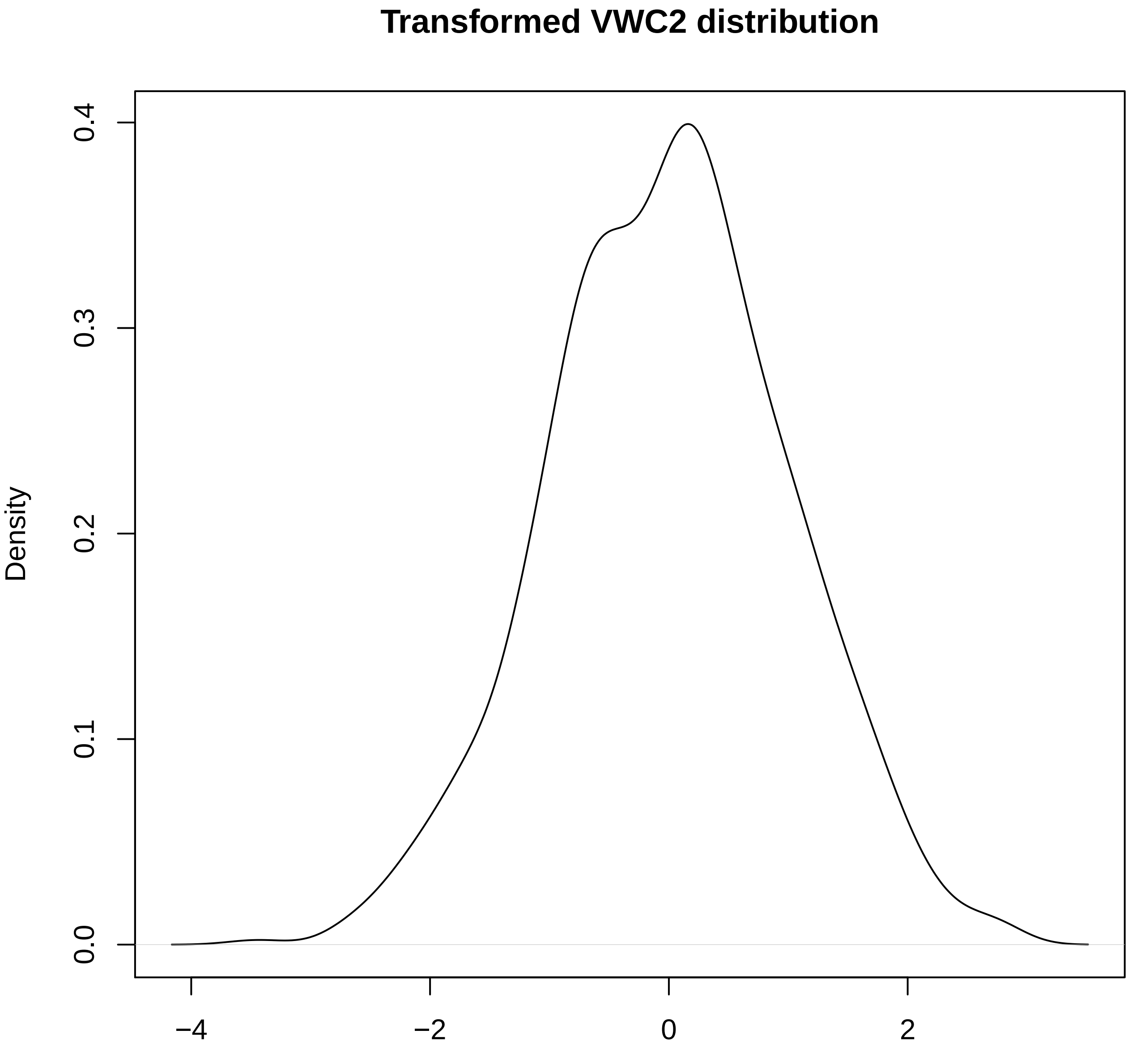
Transformed VWC2 distribution
Density
0.0
0.1
0.2
0.3
0.4
−4
−2
0
2

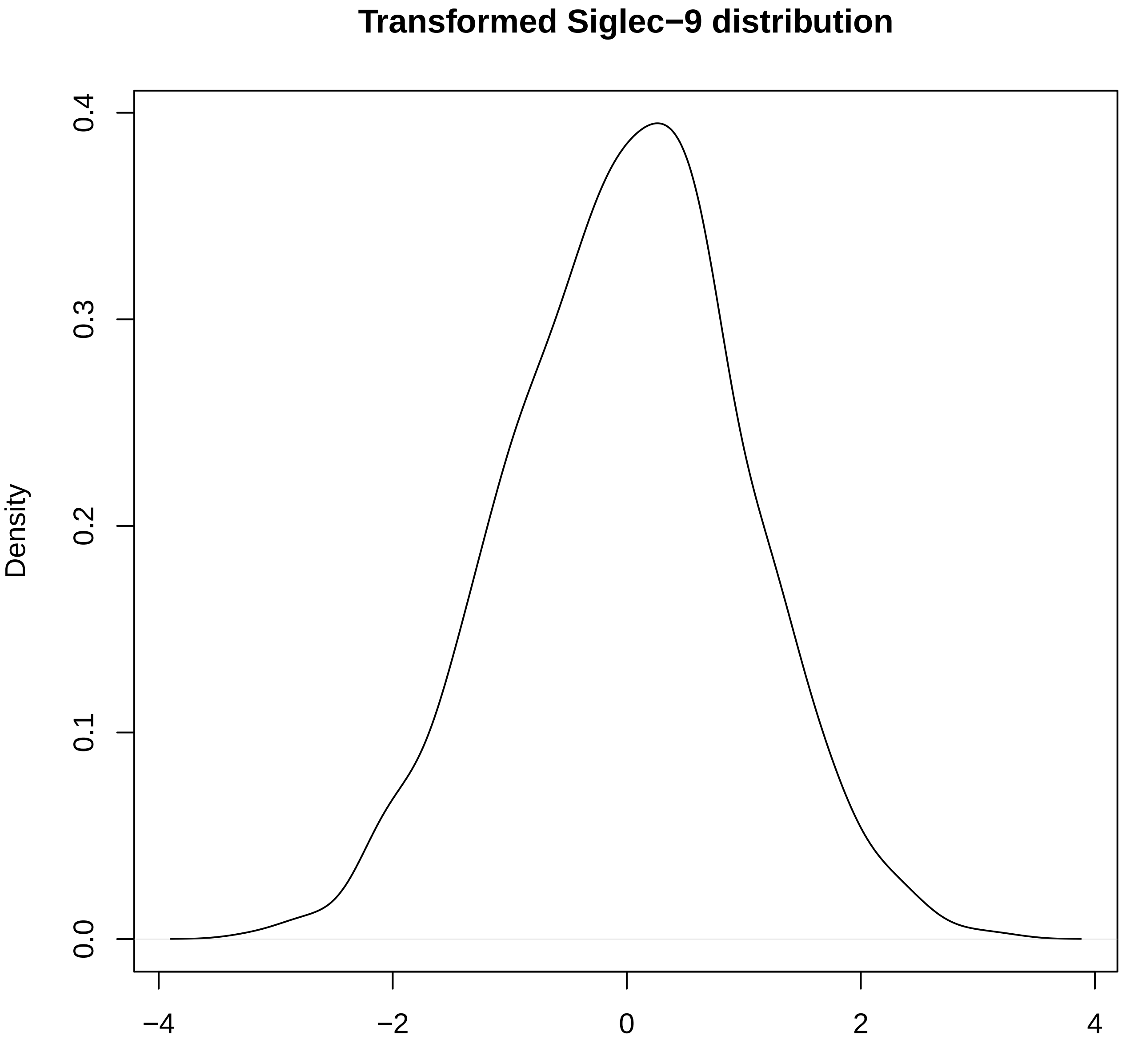
Transformed Siglec−9 distribution
Density
0.0
0.1
0.2
0.3
0.4
−4
−2
0
2
4

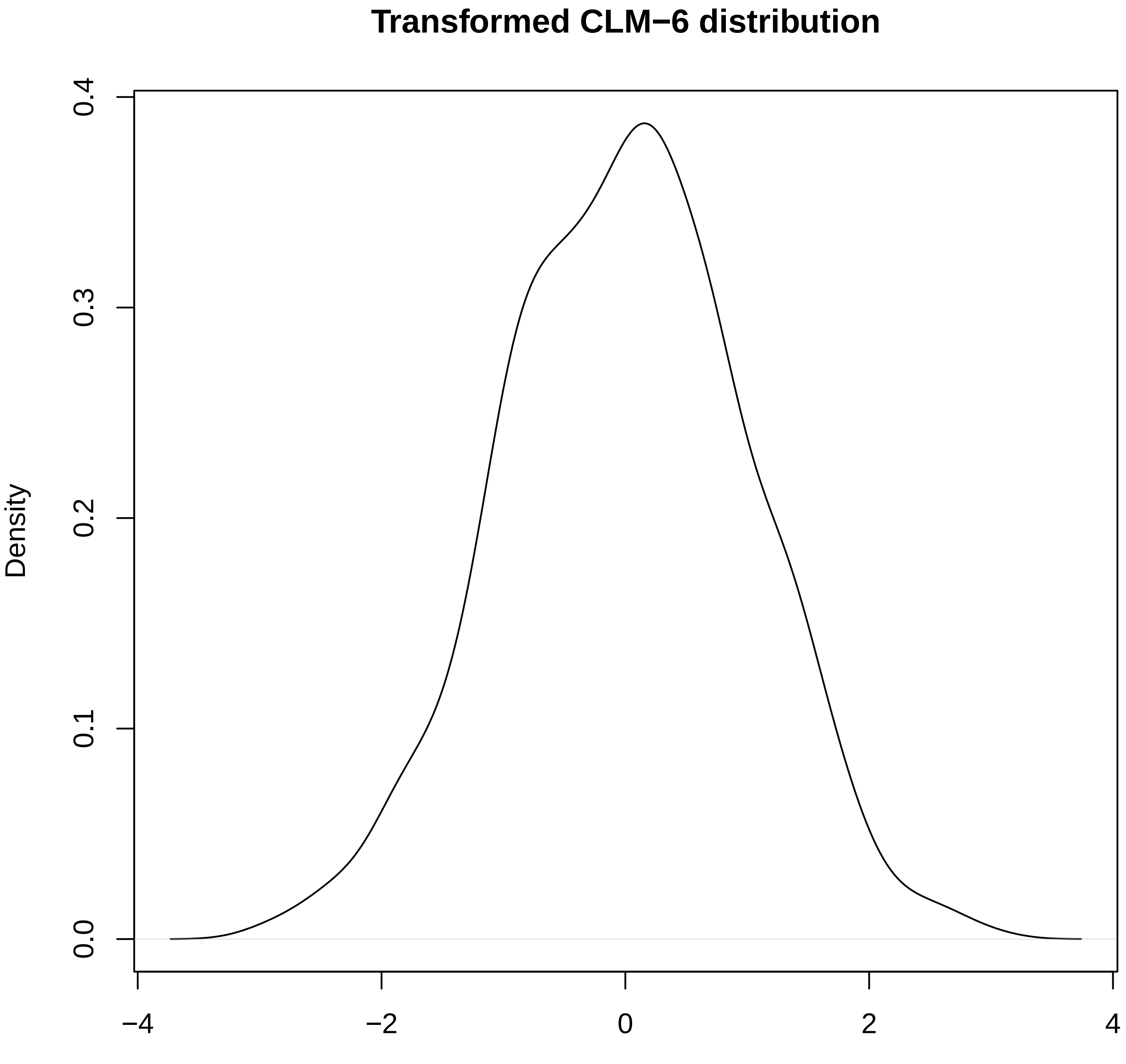

Transformed CLM−6 distribution
Density
0.0
0.1
0.2
0.3
0.4
−4
−2
0
2
4

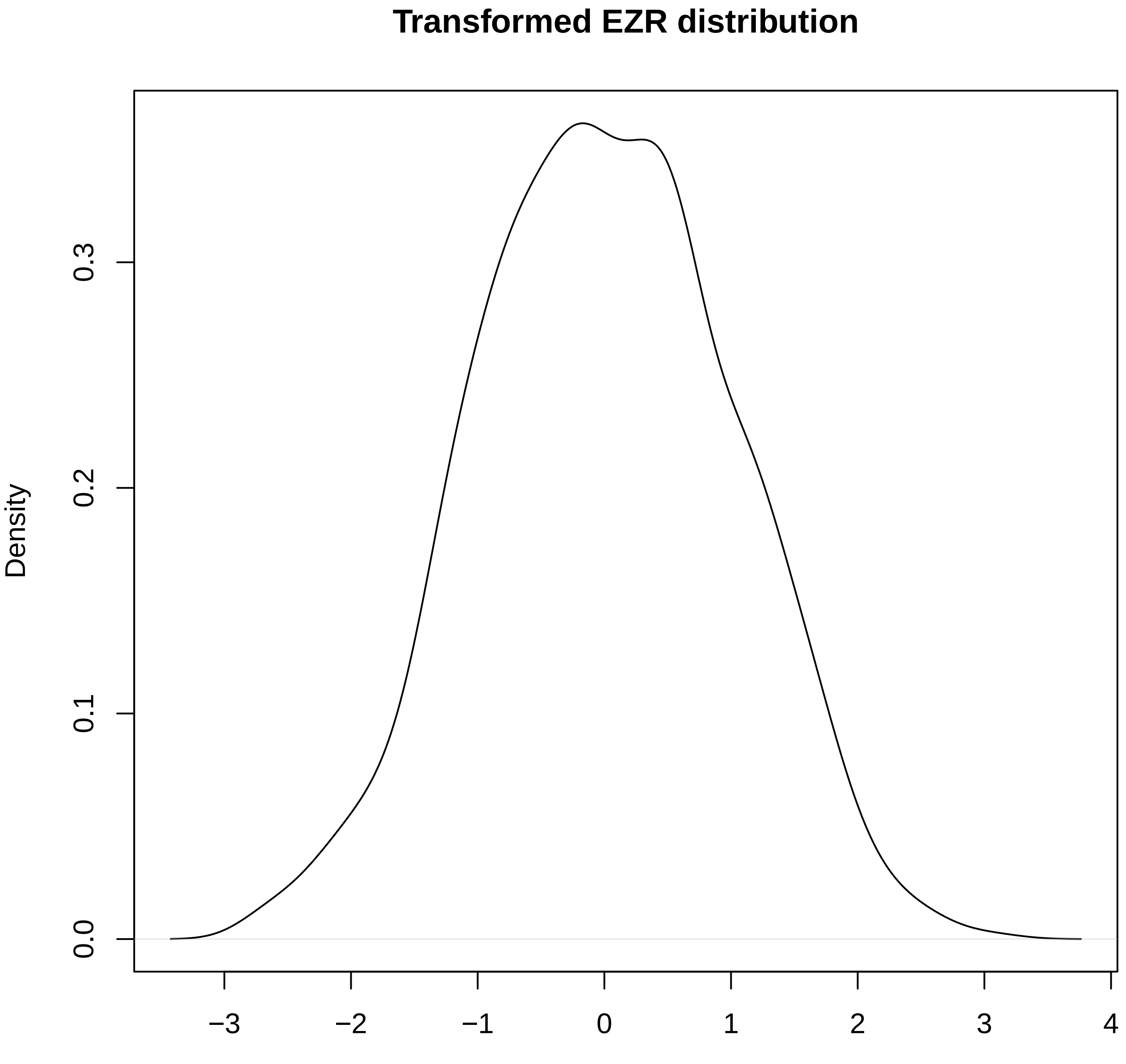
Transformed EZR distribution
Density
0.0
0.1
0.2
0.3
−3
−2
−1
0
1
2
3
4

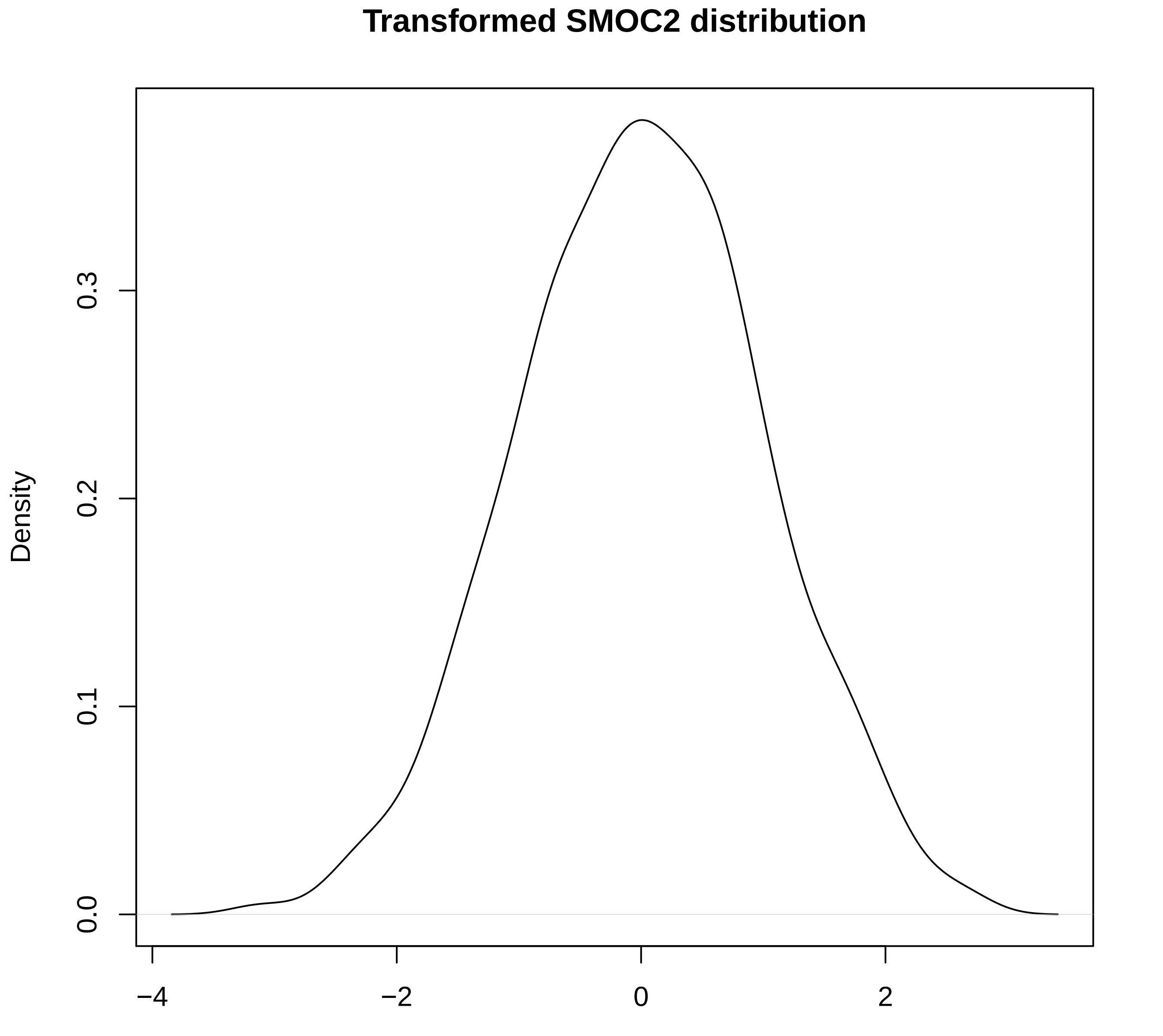

Transformed SMOC2 distribution
Density
0.0
0.1
0.2
0.3
−4
−2
0
2

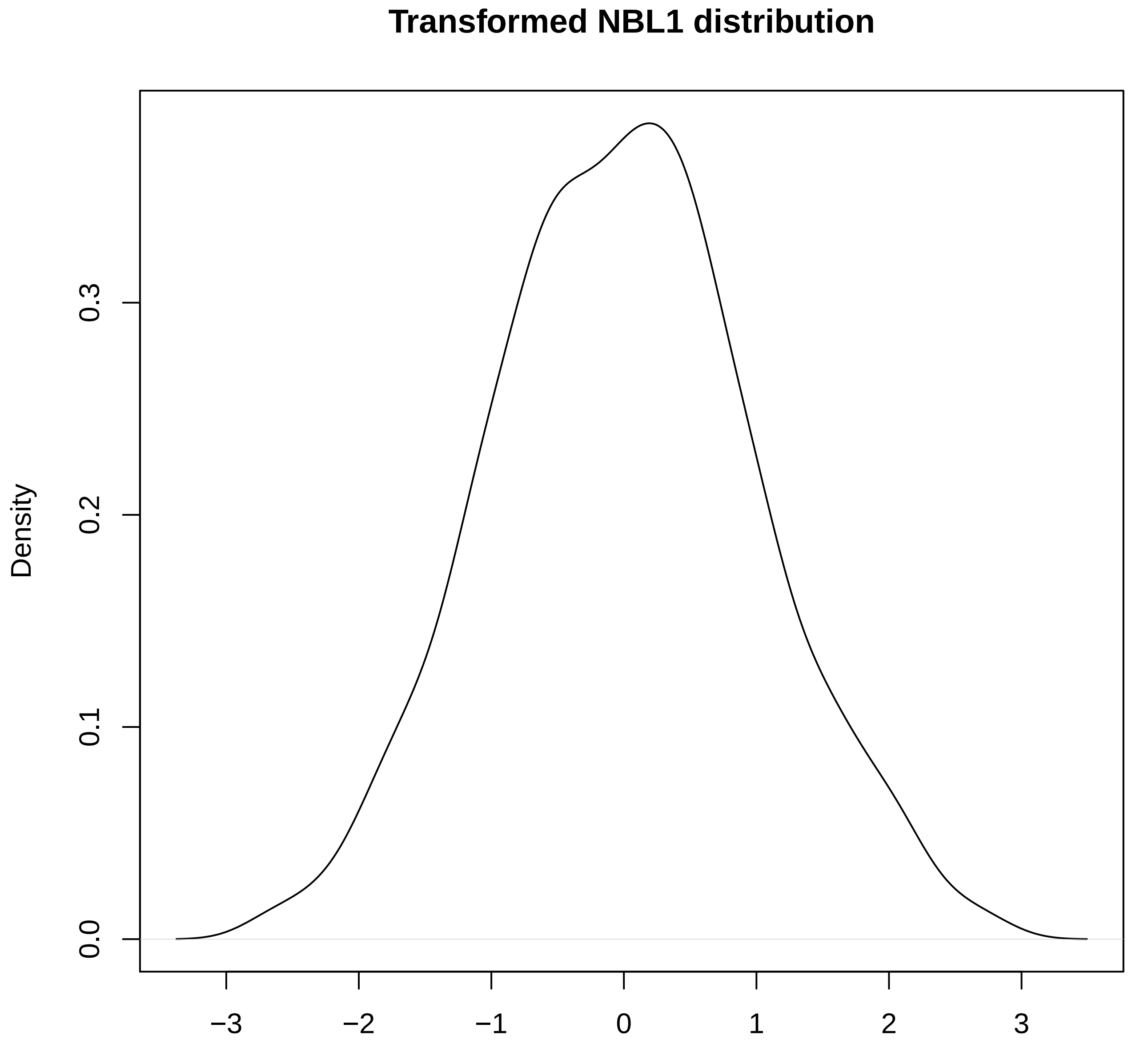

Transformed NBL1 distribution
Density
0.0
0.1
0.2
0.3
−3
−2
−1
0
1
2
3

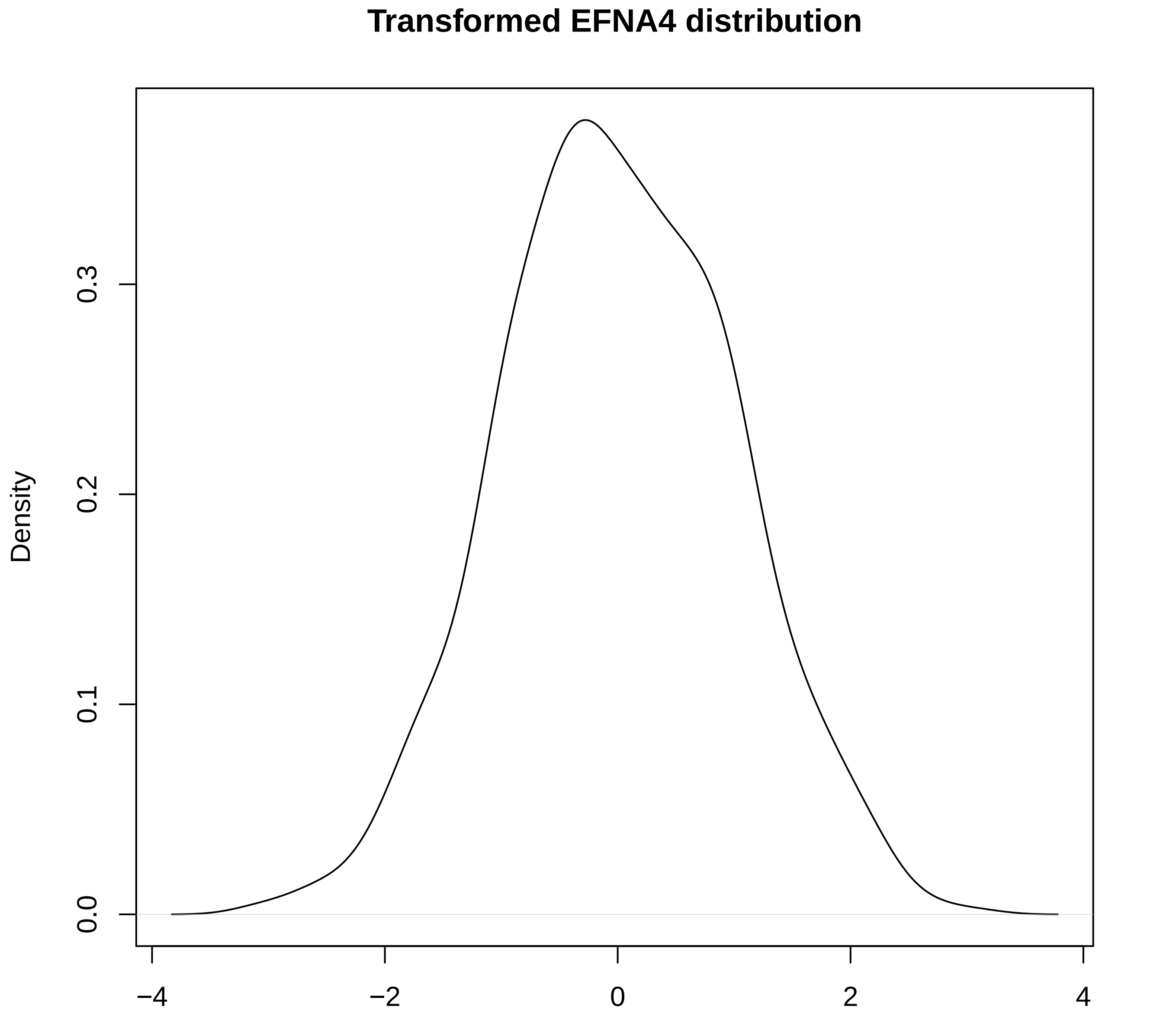

Transformed EFNA4 distribution
Density
0.0
0.1
0.2
0.3
−4
−2
0
2
4

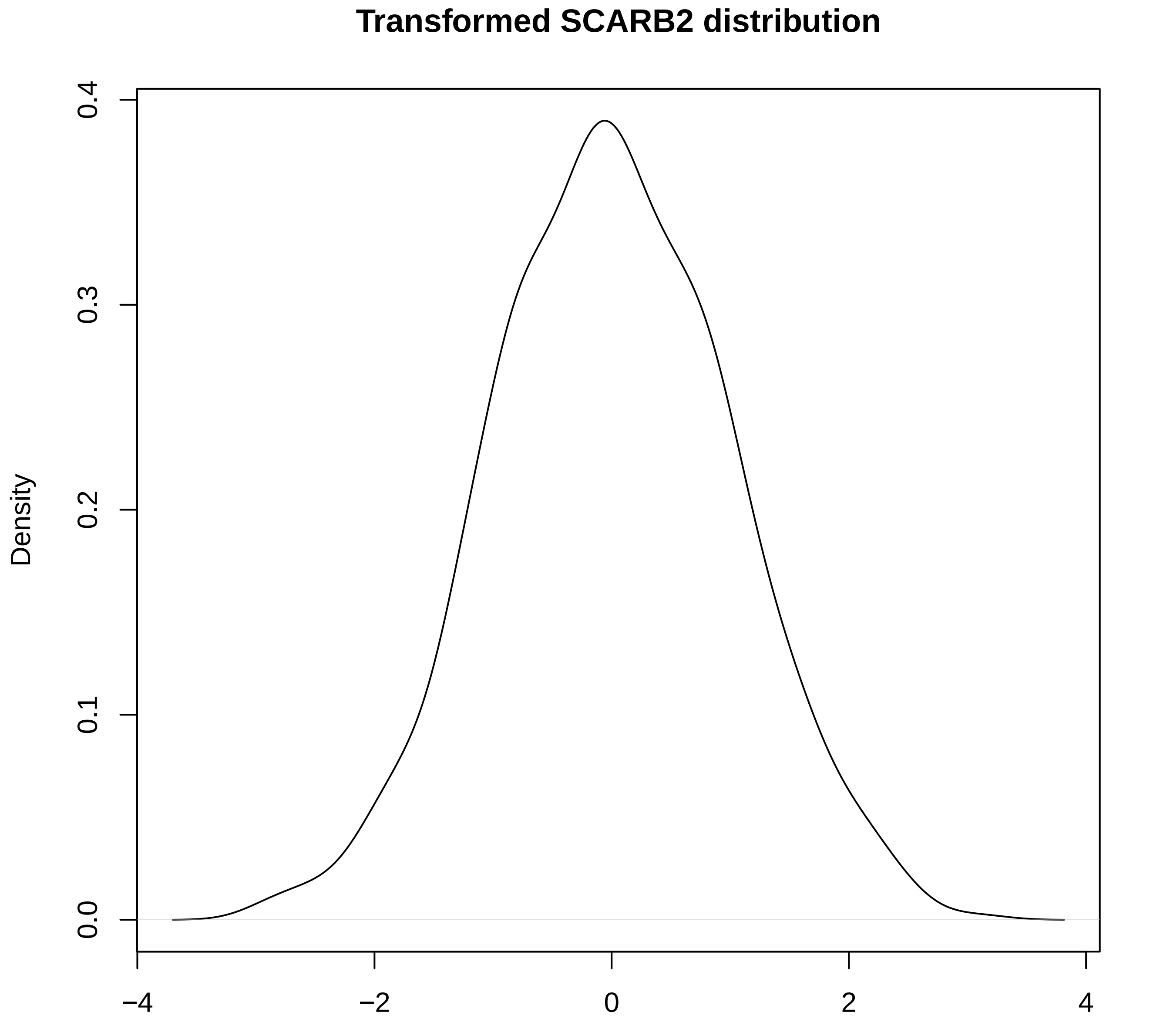
Transformed SCARB2 distribution
Density
0.0
0.1
0.2
0.3
0.4
−4
−2
0
2
4

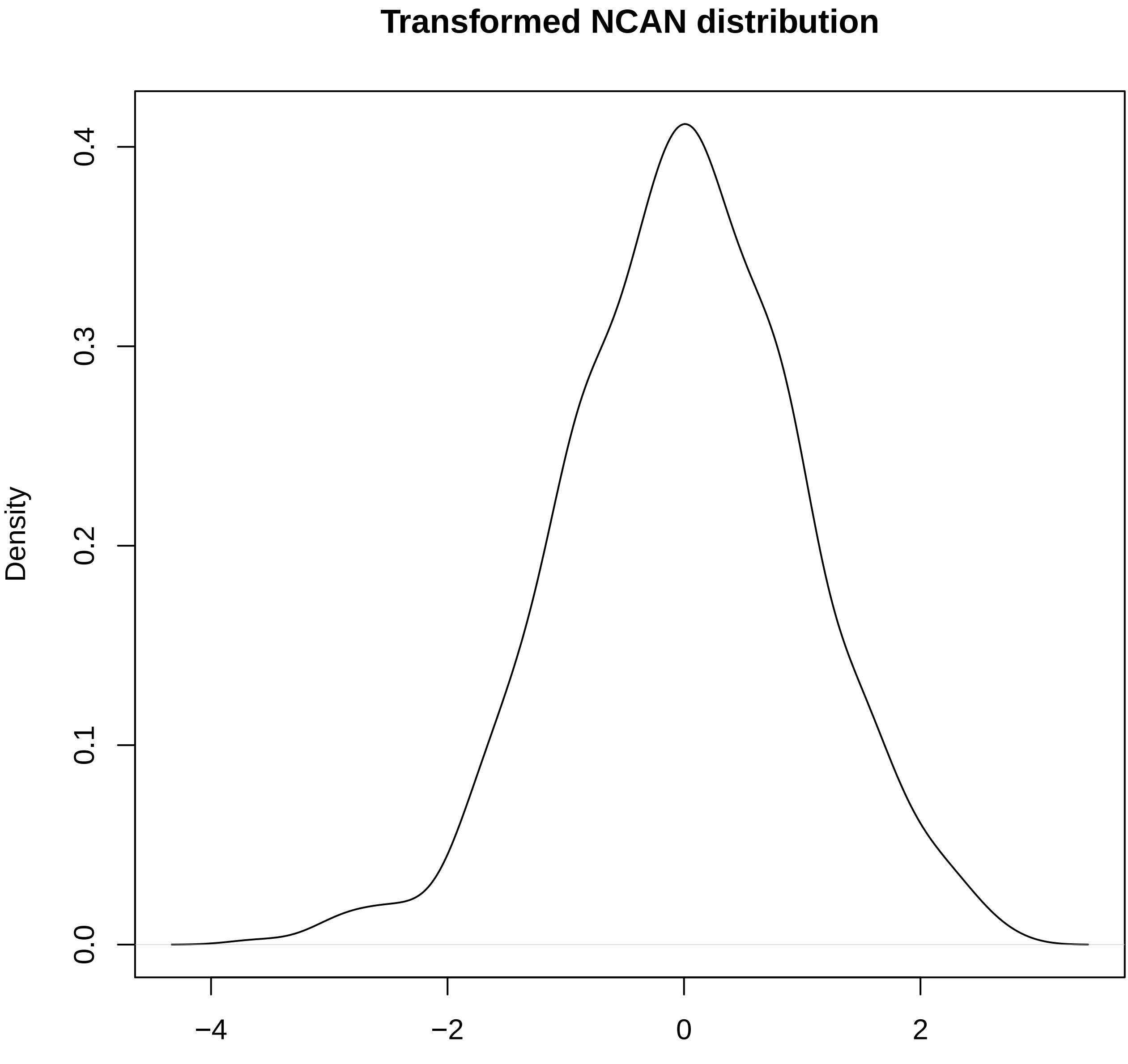

Transformed NCAN distribution
Density
0.0
0.1
0.2
0.3
0.4
−4
−2
0
2

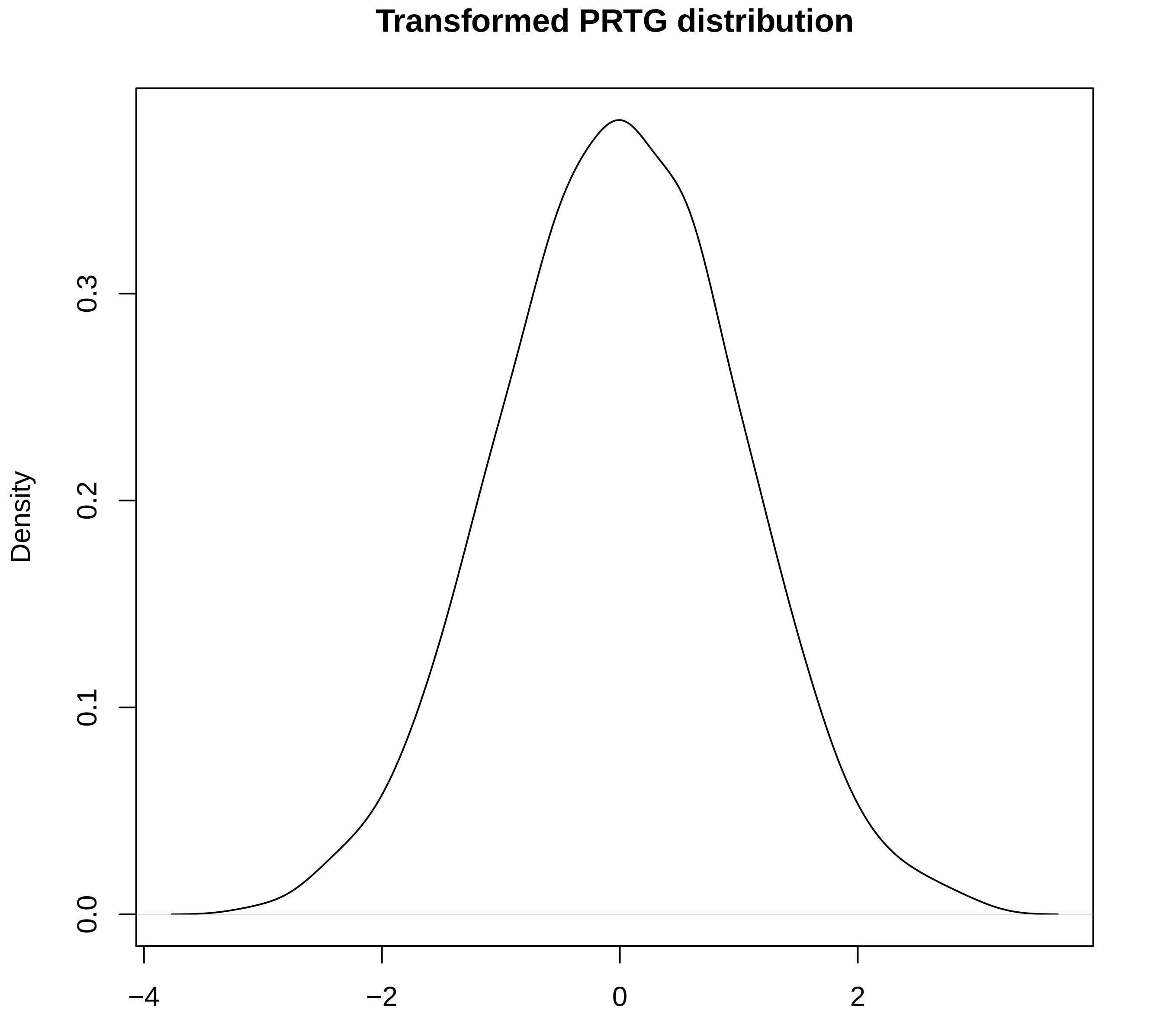
Transformed PRTG distribution
Density
0.0
0.1
0.2
0.3
−4
−2
0
2

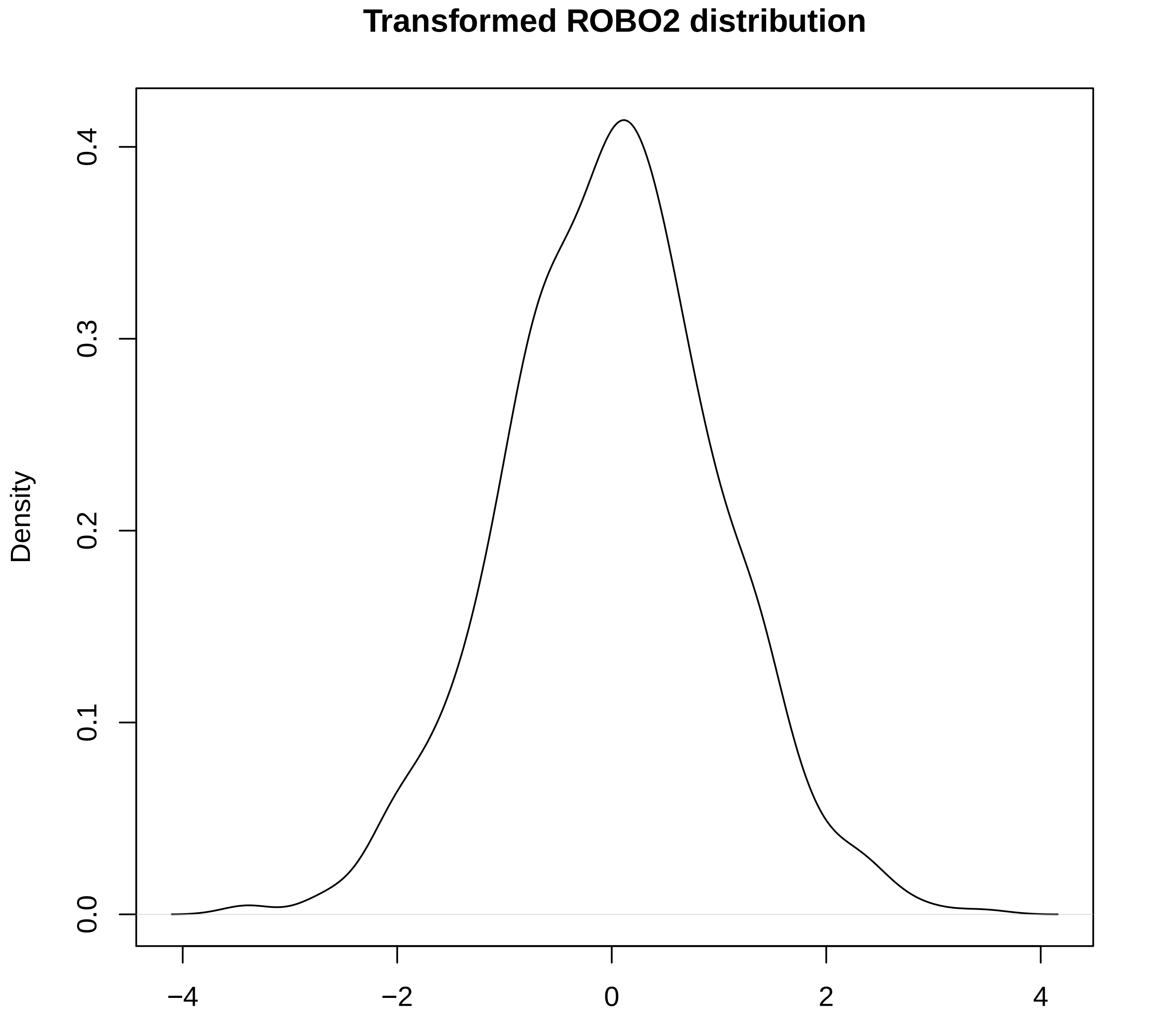

Transformed ROBO2 distribution
Density
0.0
0.1
0.2
0.3
0.4
−4
−2
0
2
4

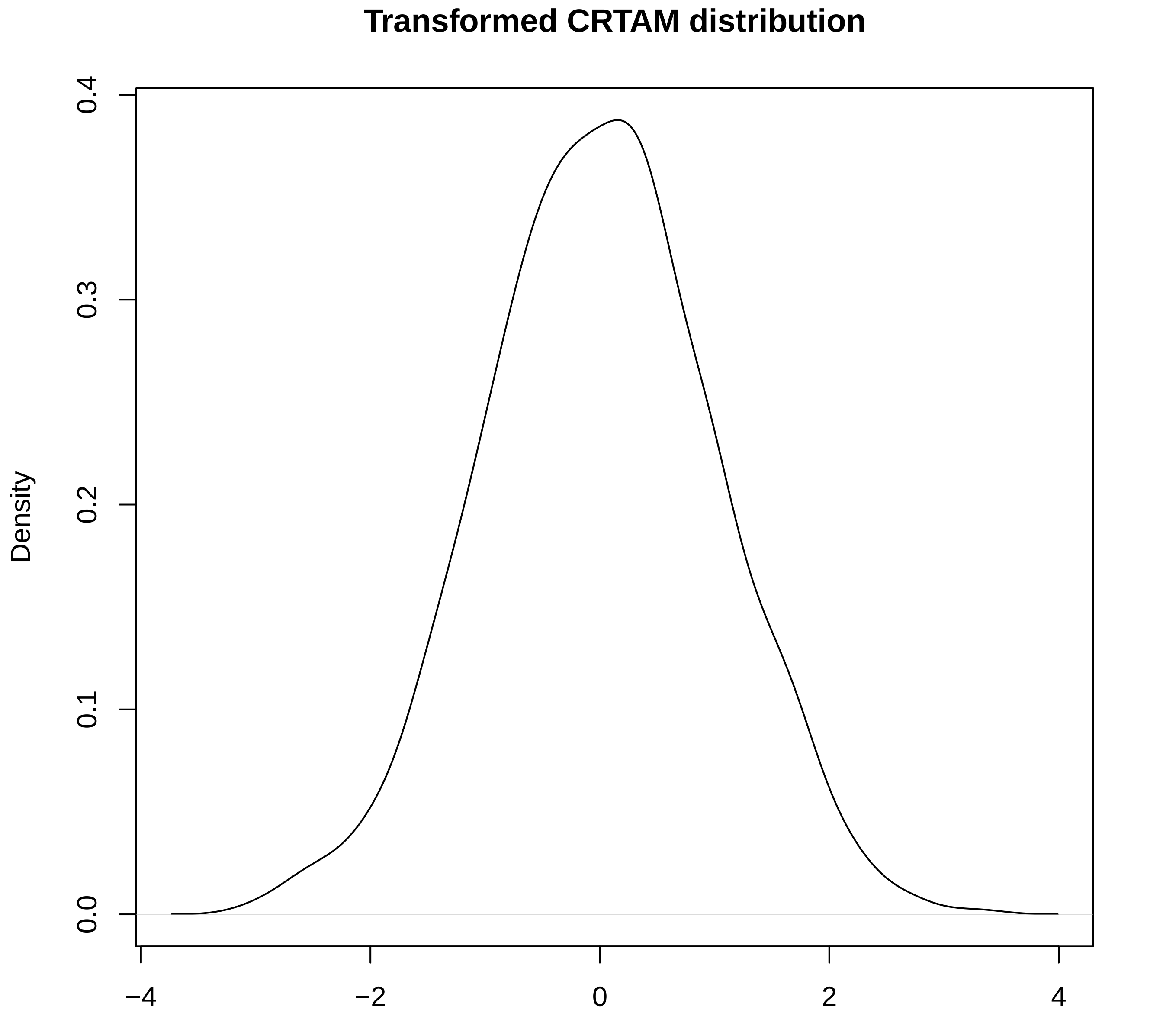
Transformed CRTAM distribution
Density
0.0
0.1
0.2
0.3
0.4
−4
−2
0
2
4

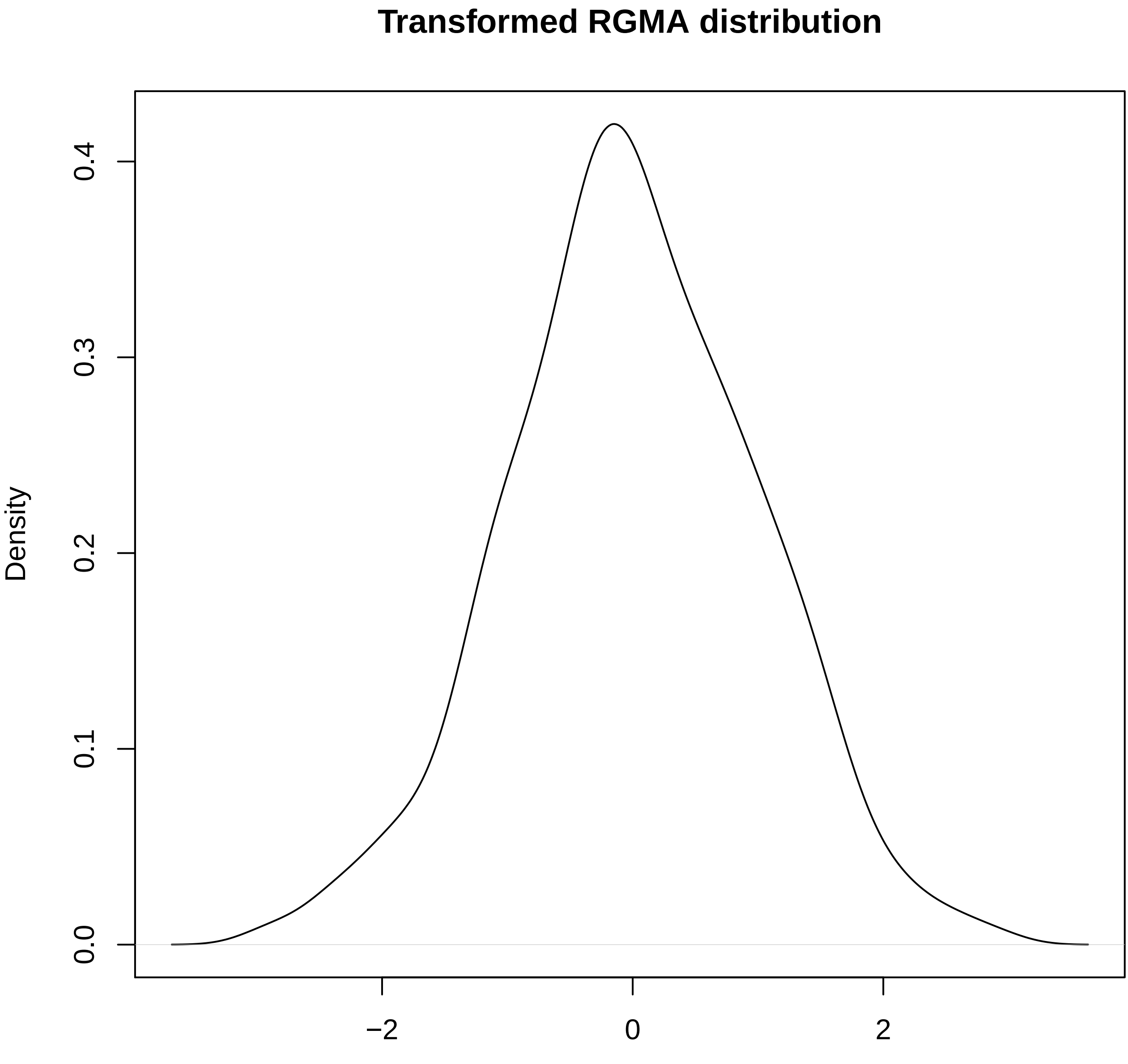

Transformed RGMA distribution
Density
0.0
0.1
0.2
0.3
0.4
−2
0
2

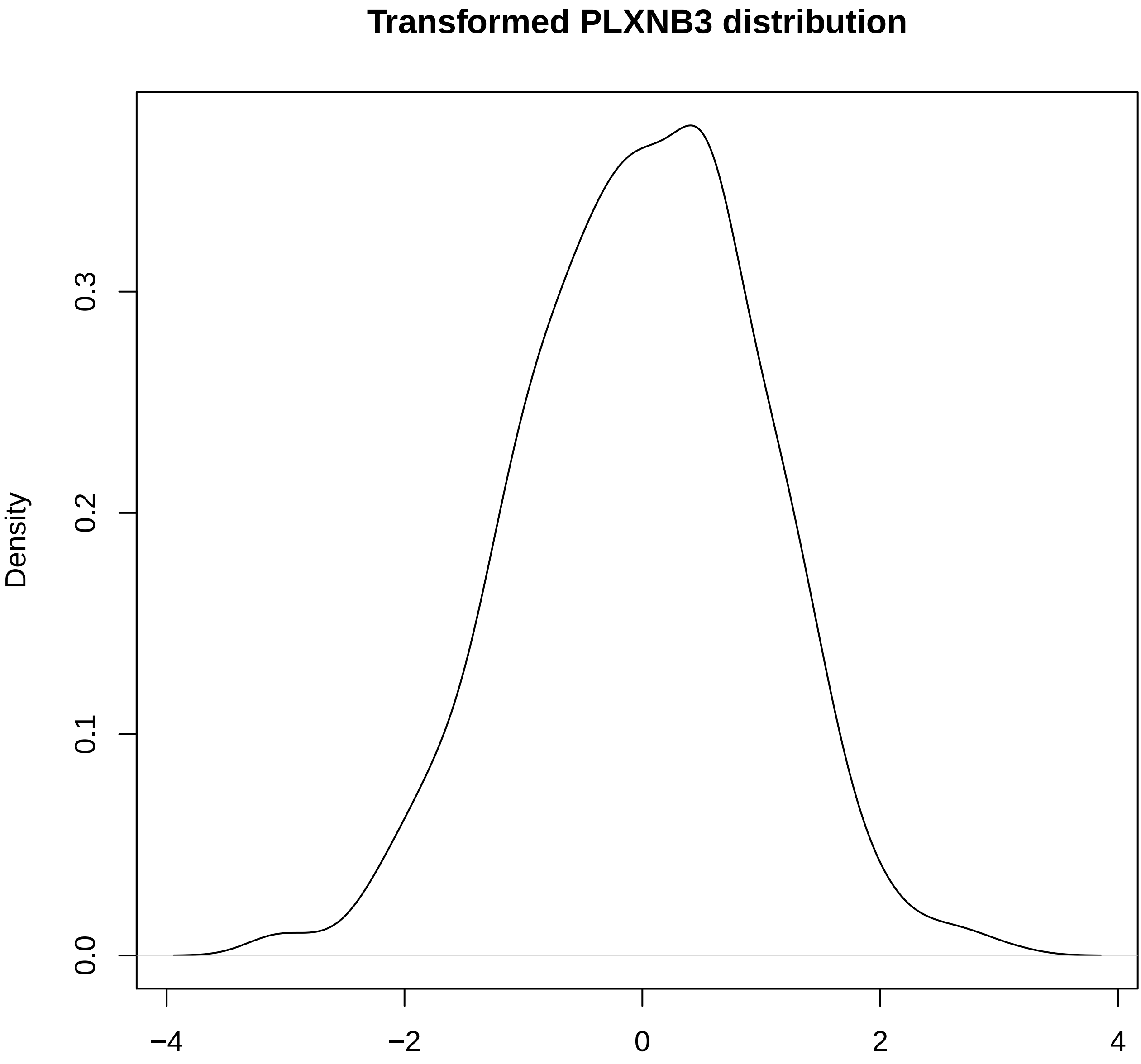

Transformed PLXNB3 distribution
Density
0.0
0.1
0.2
0.3
−4
−2
0
2
4

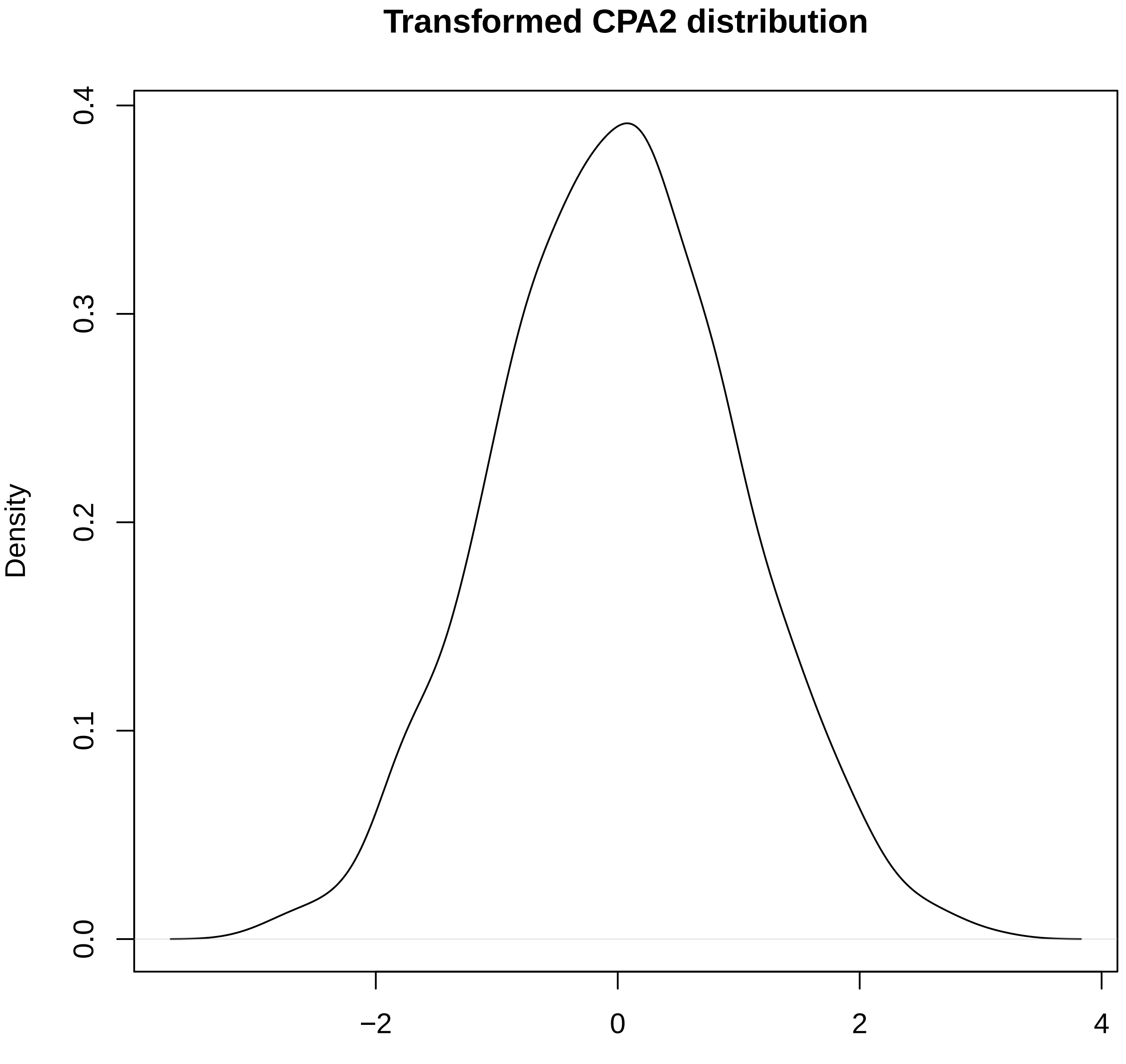

Transformed CPA2 distribution
Density
0.0
0.1
0.2
0.3
0.4
−2
0
2
4

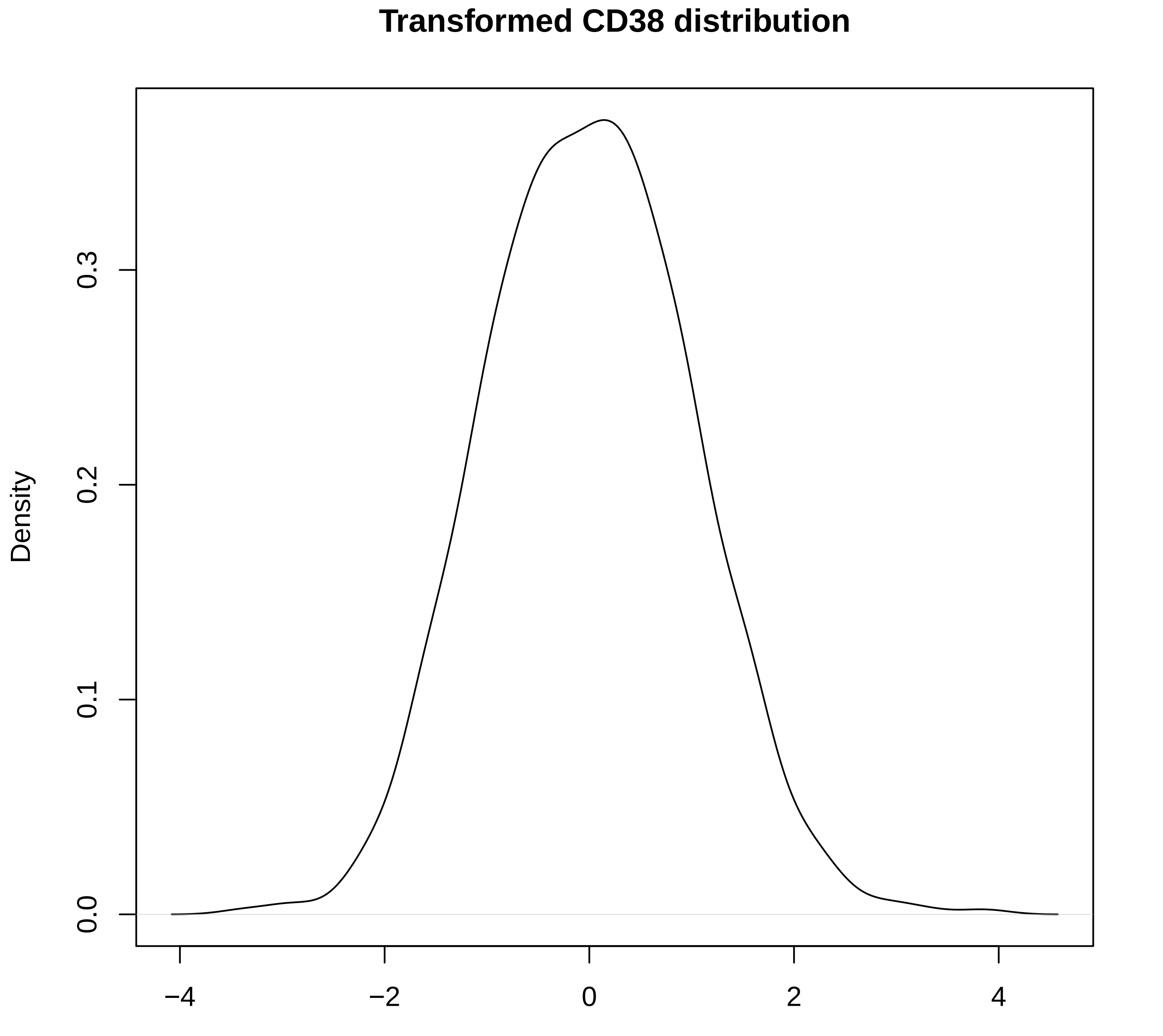

Transformed CD38 distribution
Density
0.0
0.1
0.2
0.3
−4
−2
0
2
4

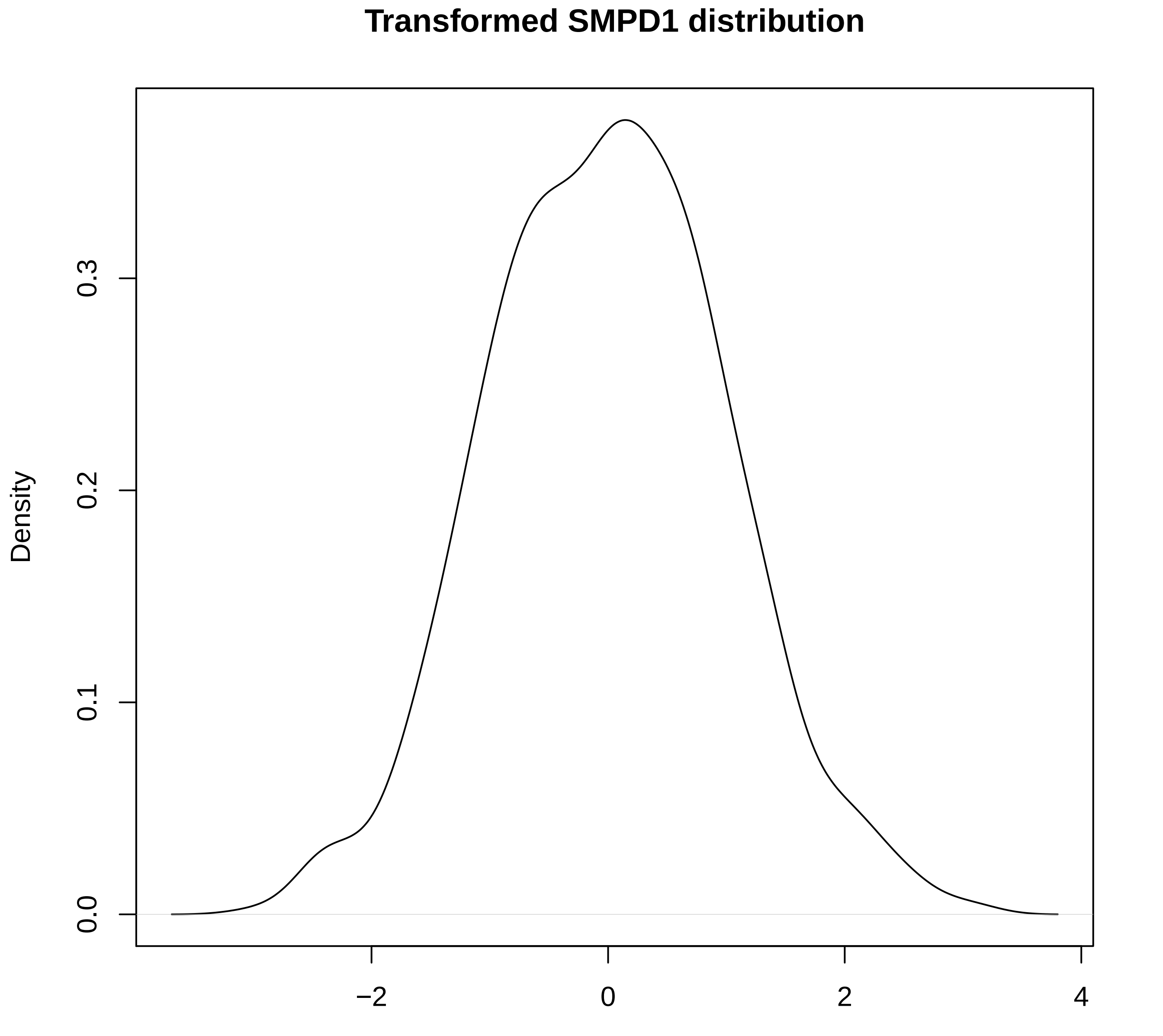
Transformed SMPD1 distribution
Density
0.0
0.1
0.2
0.3
−2
0
2
4

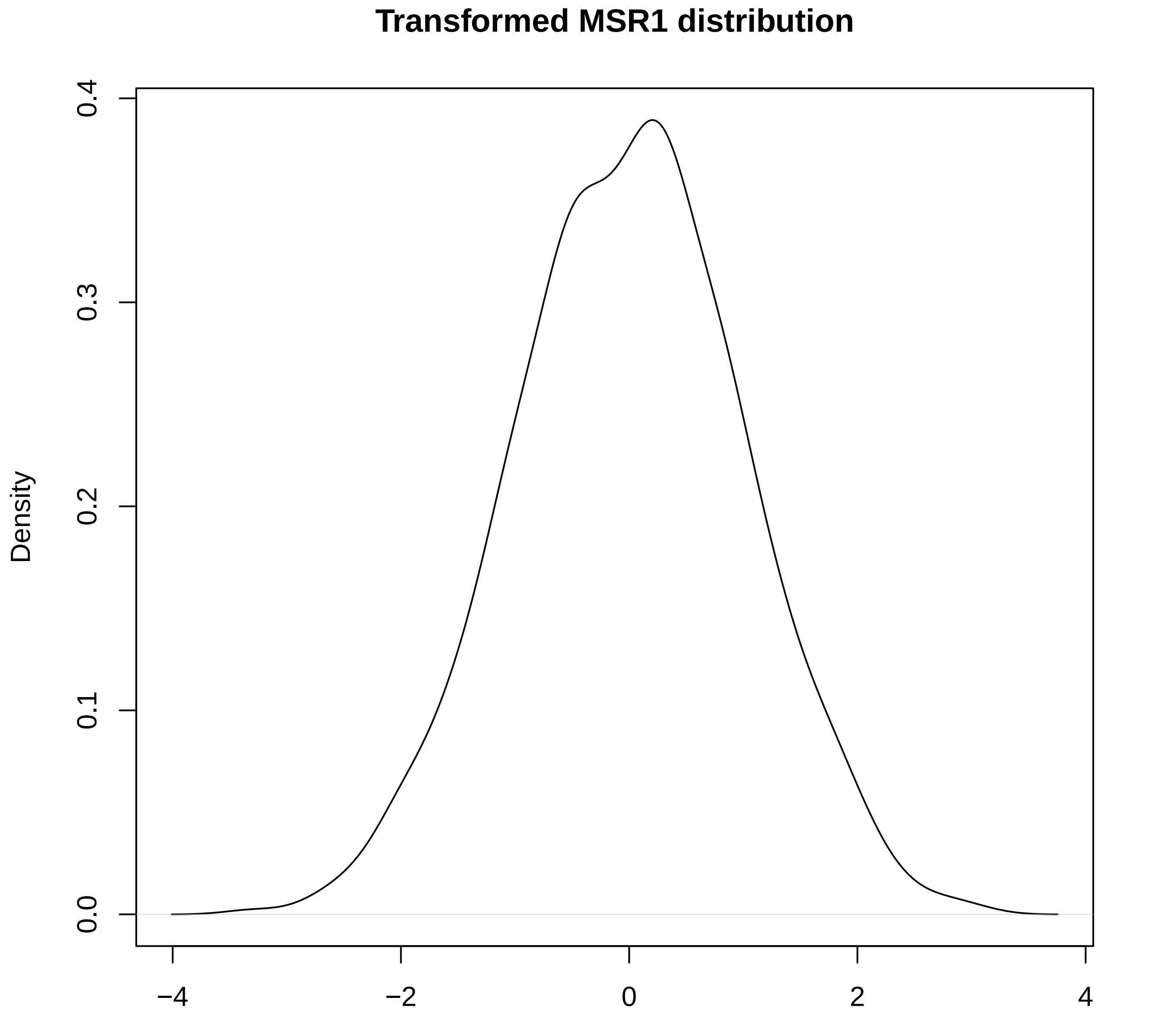
Transformed MSR1 distribution
Density
0.0
0.1
0.2
0.3
0.4
−4
−2
0
2
4

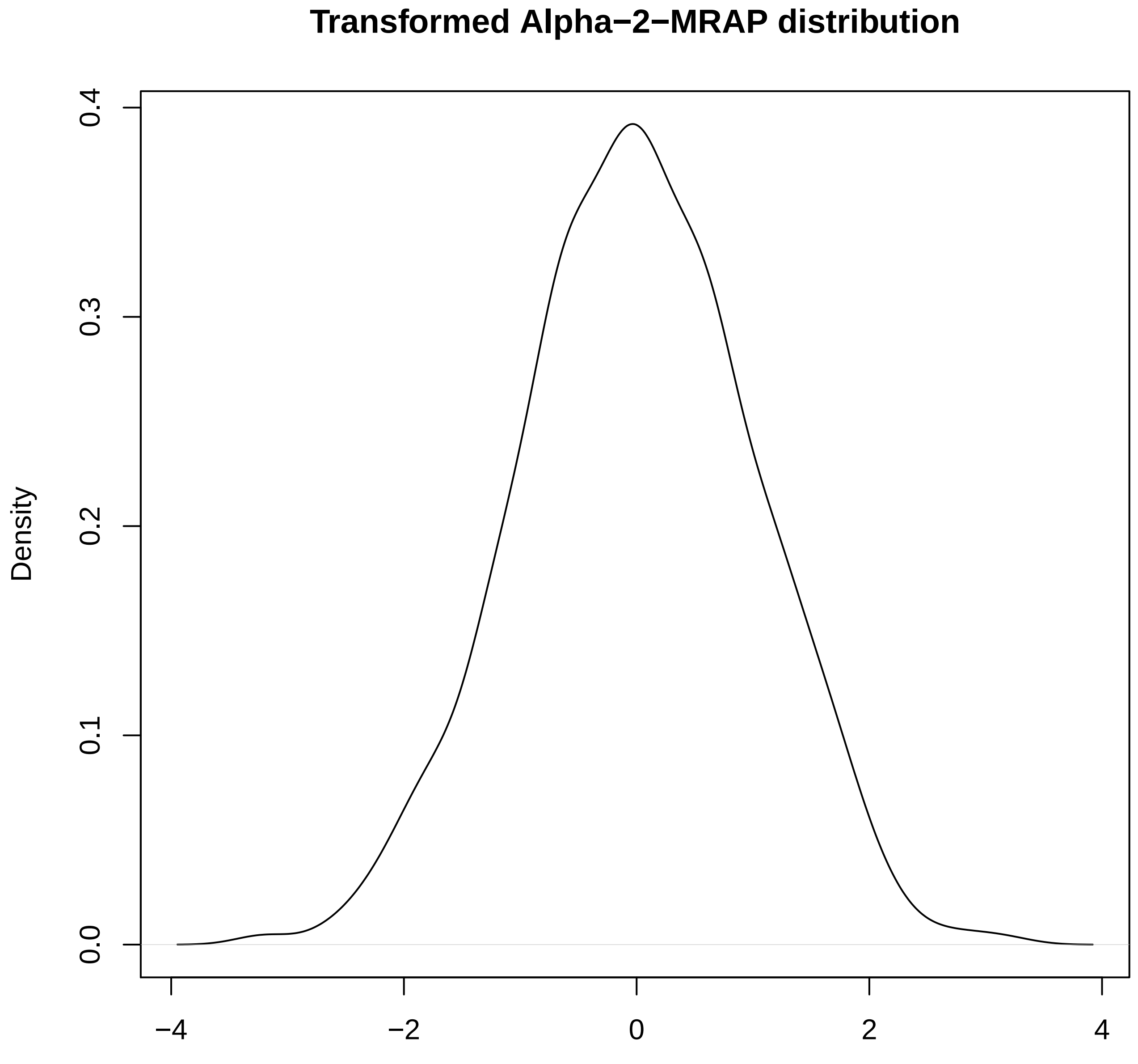

Transformed Alpha−2−MRAP distribution
Density
0.0
0.1
0.2
0.3
0.4
−4
−2
0
2
4

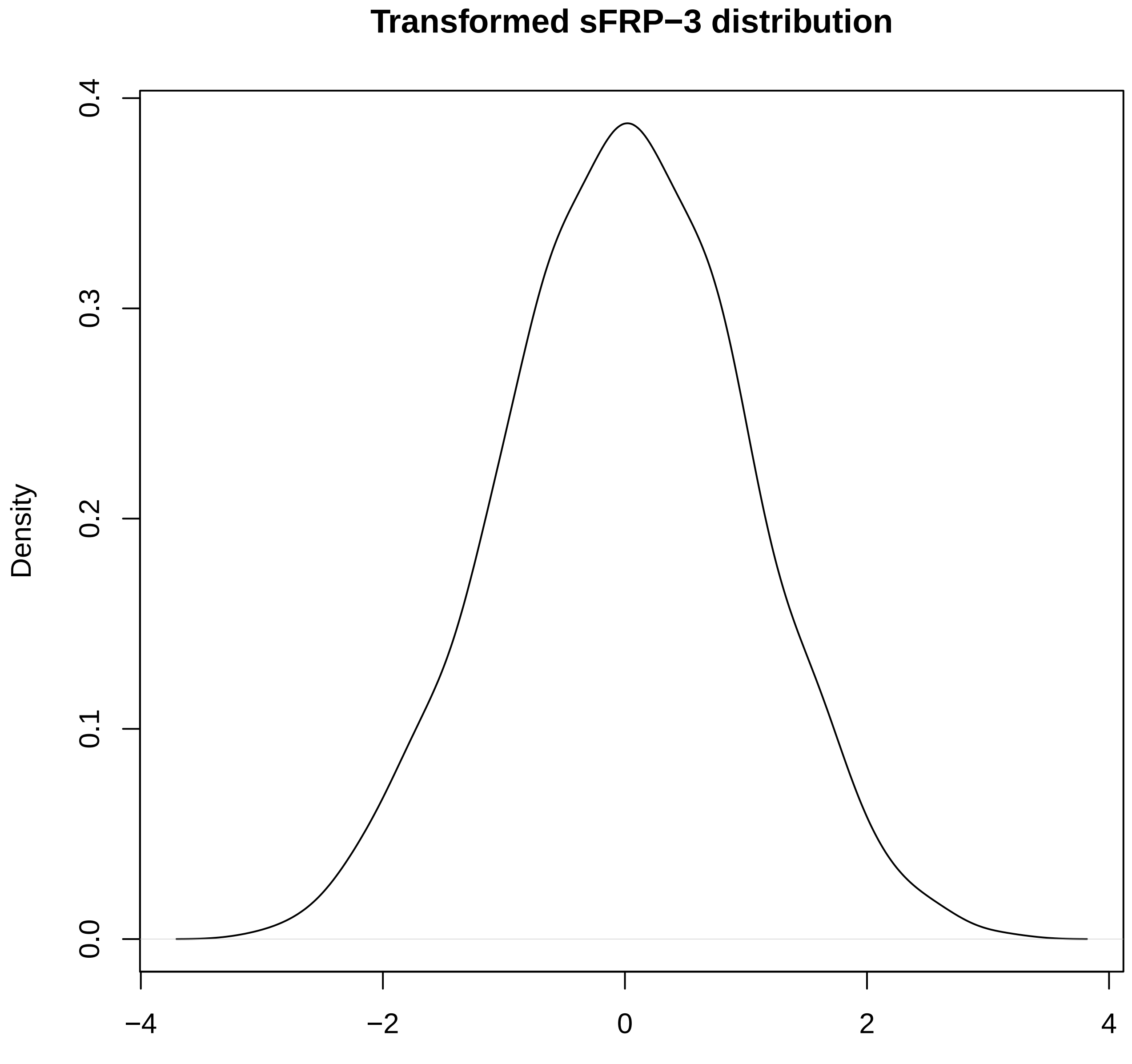

Transformed sFRP−3 distribution
Density
0.0
0.1
0.2
0.3
0.4
−4
−2
0
2
4

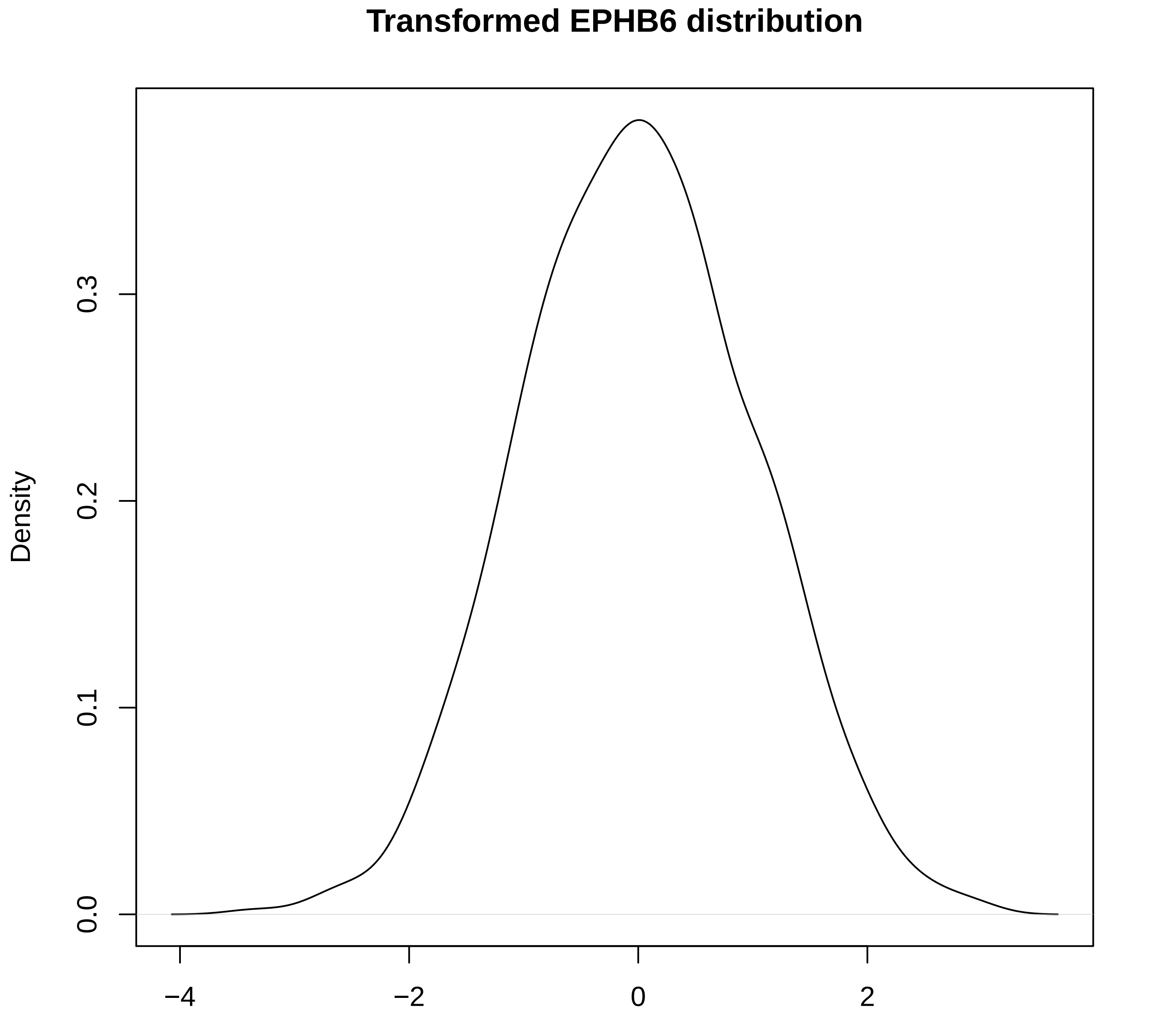

Transformed EPHB6 distribution
Density
0.0
0.1
0.2
0.3
−4
−2
0
2

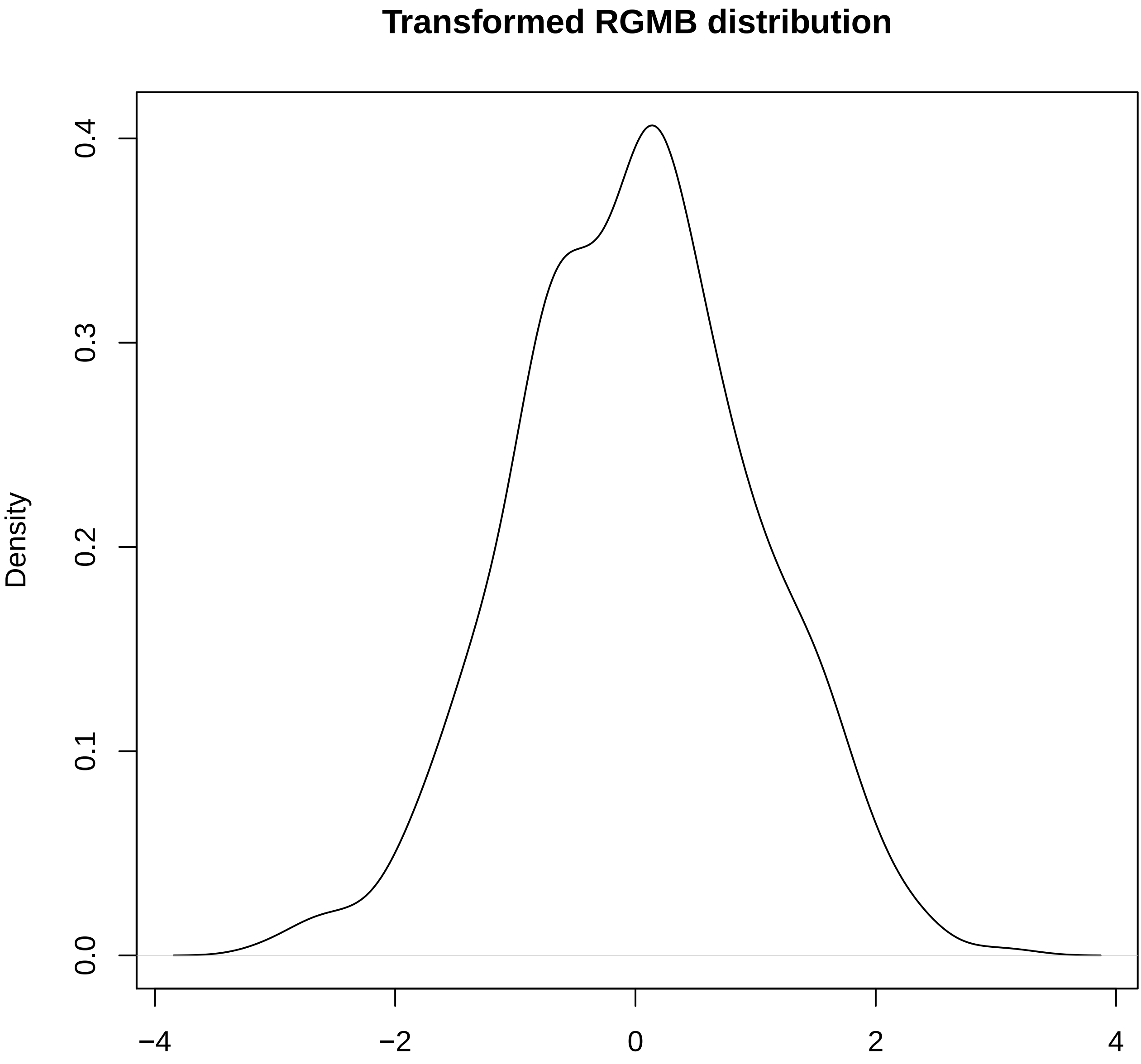

Transformed RGMB distribution
Density
0.4
0.3
0.2
0.1
0.0
−4
−2
0
2
4

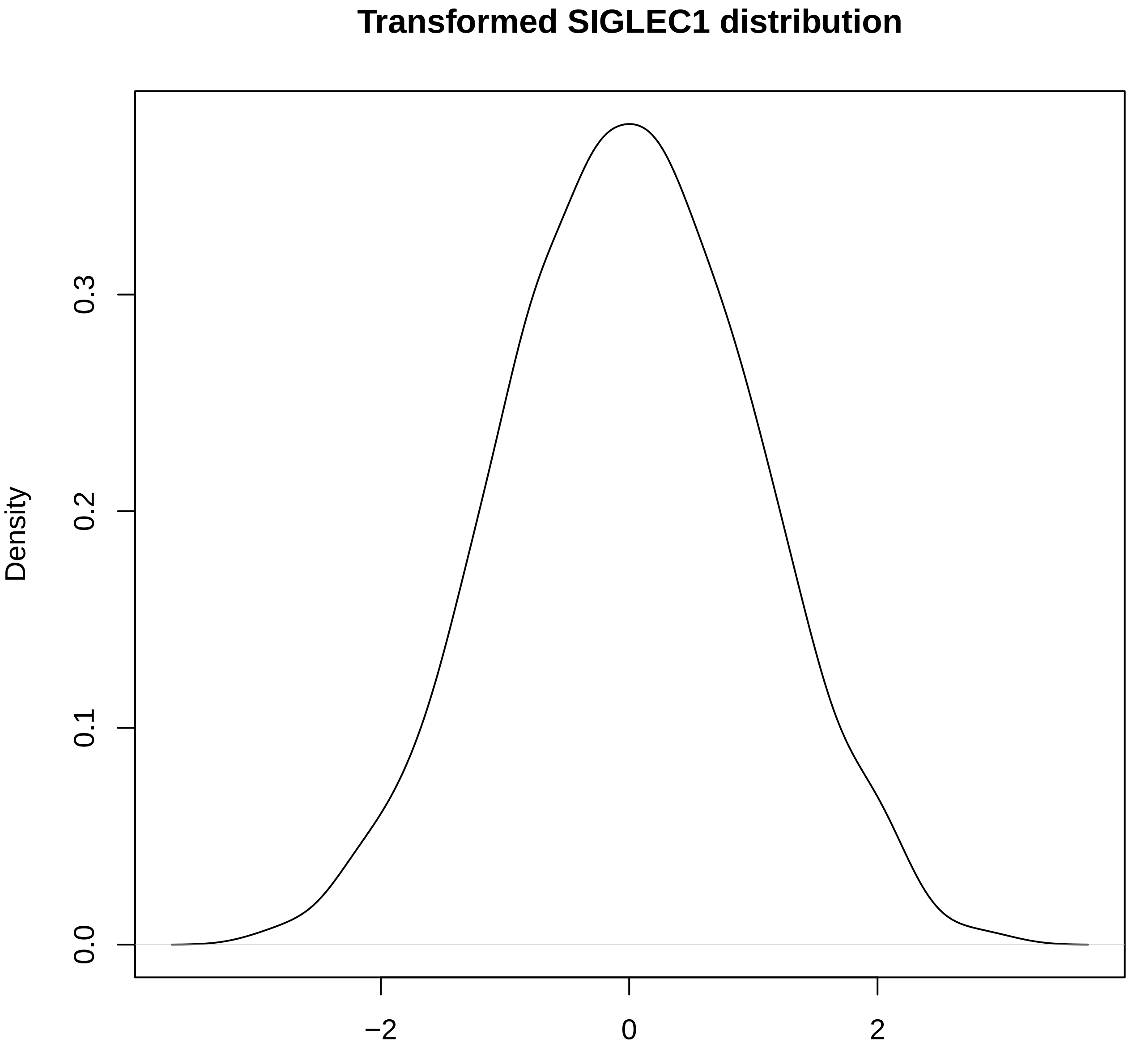
Transformed SIGLEC1 distribution
Density
0.0
0.1
0.2
0.3
−2
0
2

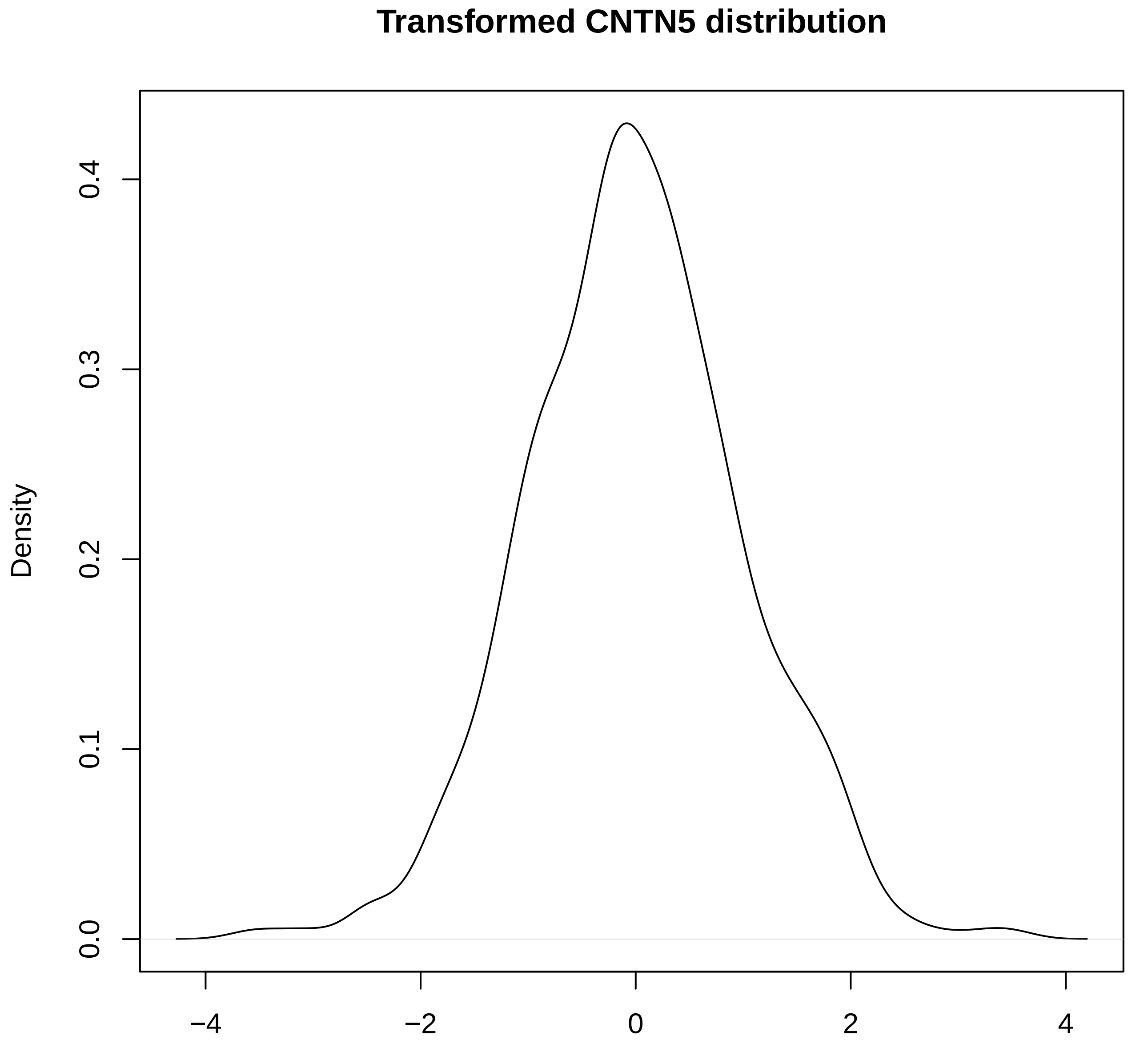

Transformed CNTN5 distribution
Density
0.0
0.1
0.2
0.3
0.4
−4
−2
0
2
4

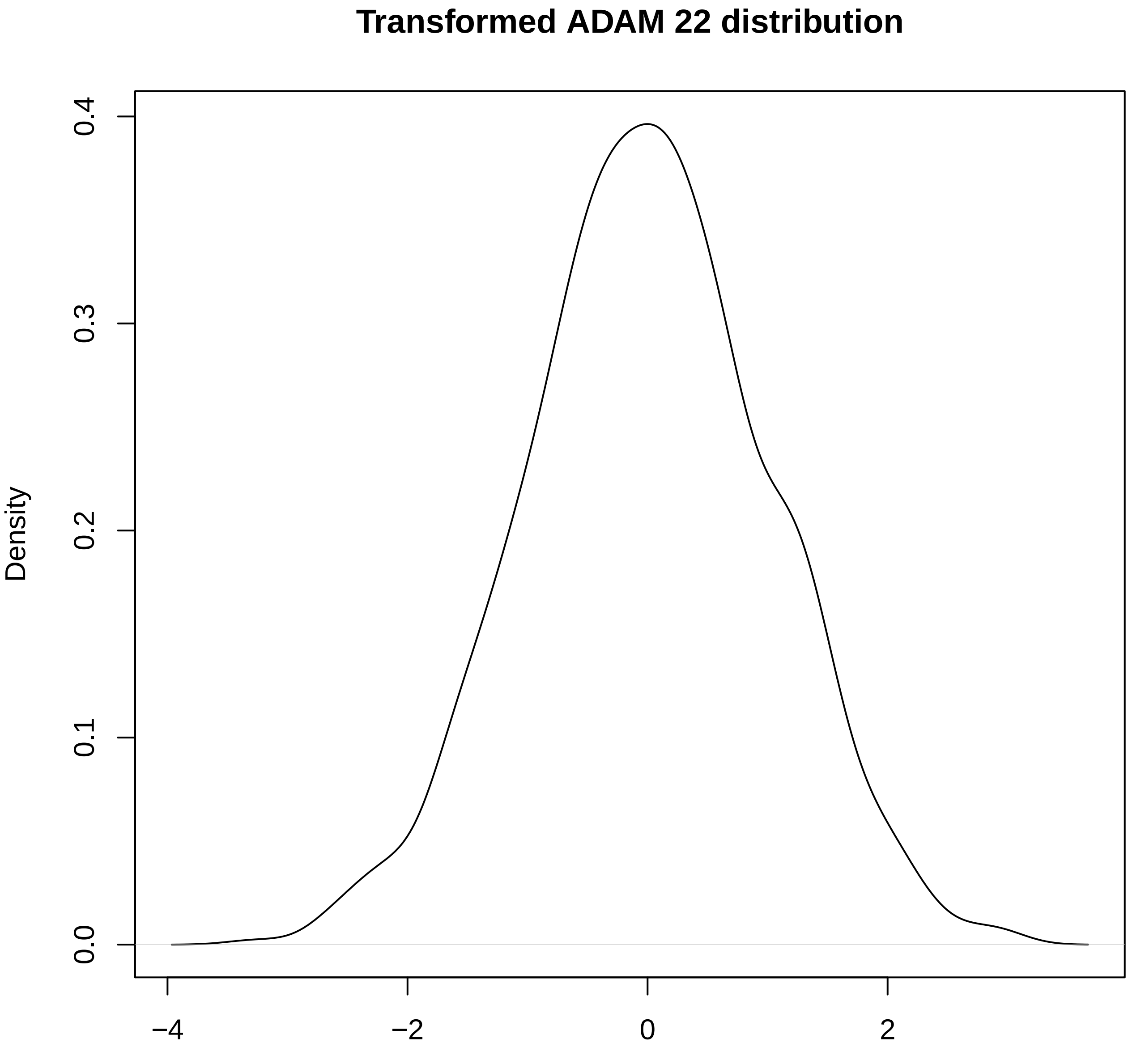
Transformed ADAM 22 distribution
Density
0.0
0.1
0.2
0.3
0.4
−4
−2
0
2

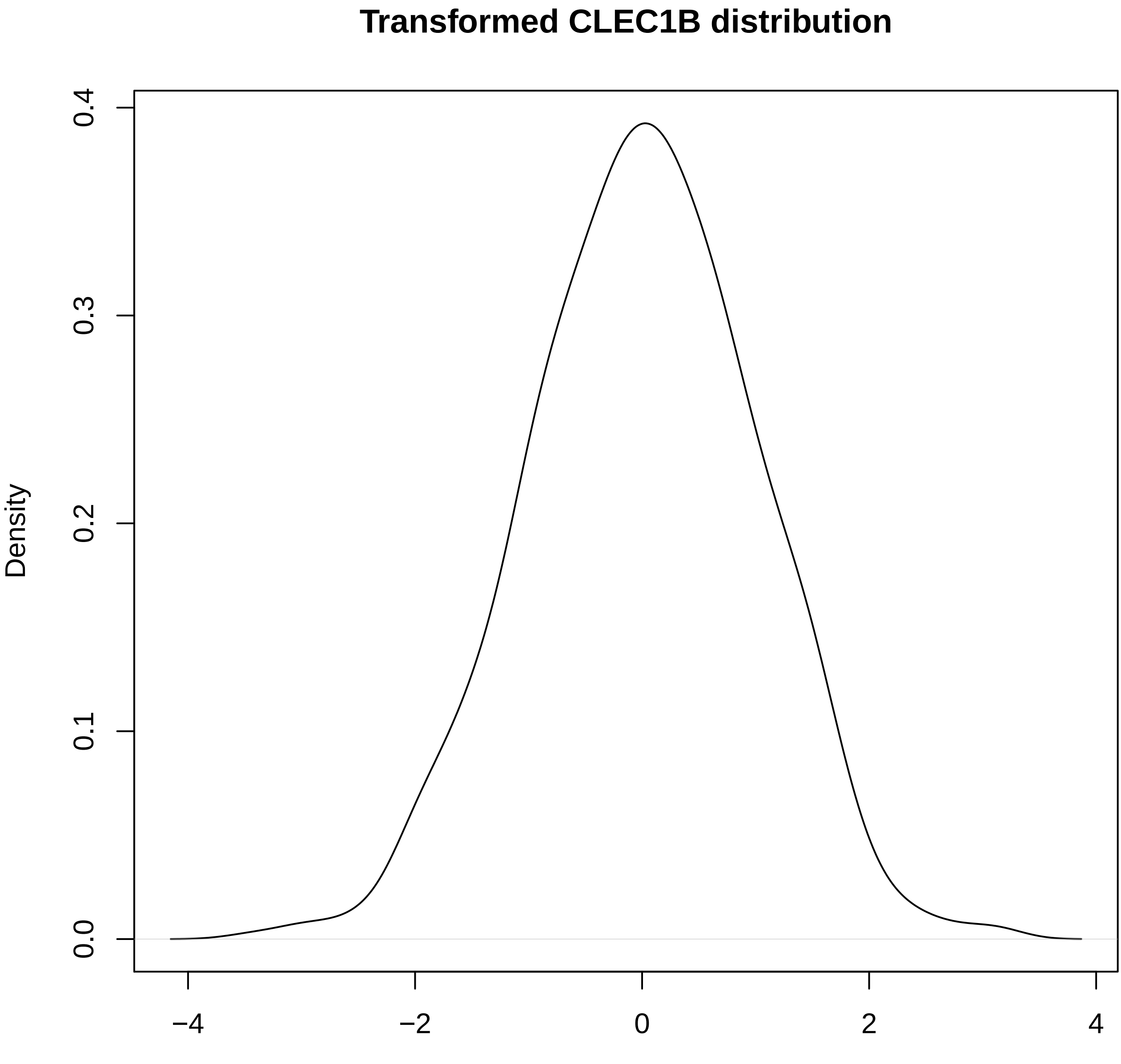
Transformed CLEC1B distribution
Density
0.0
0.1
0.2
0.3
0.4
−4
−2
0
2
4

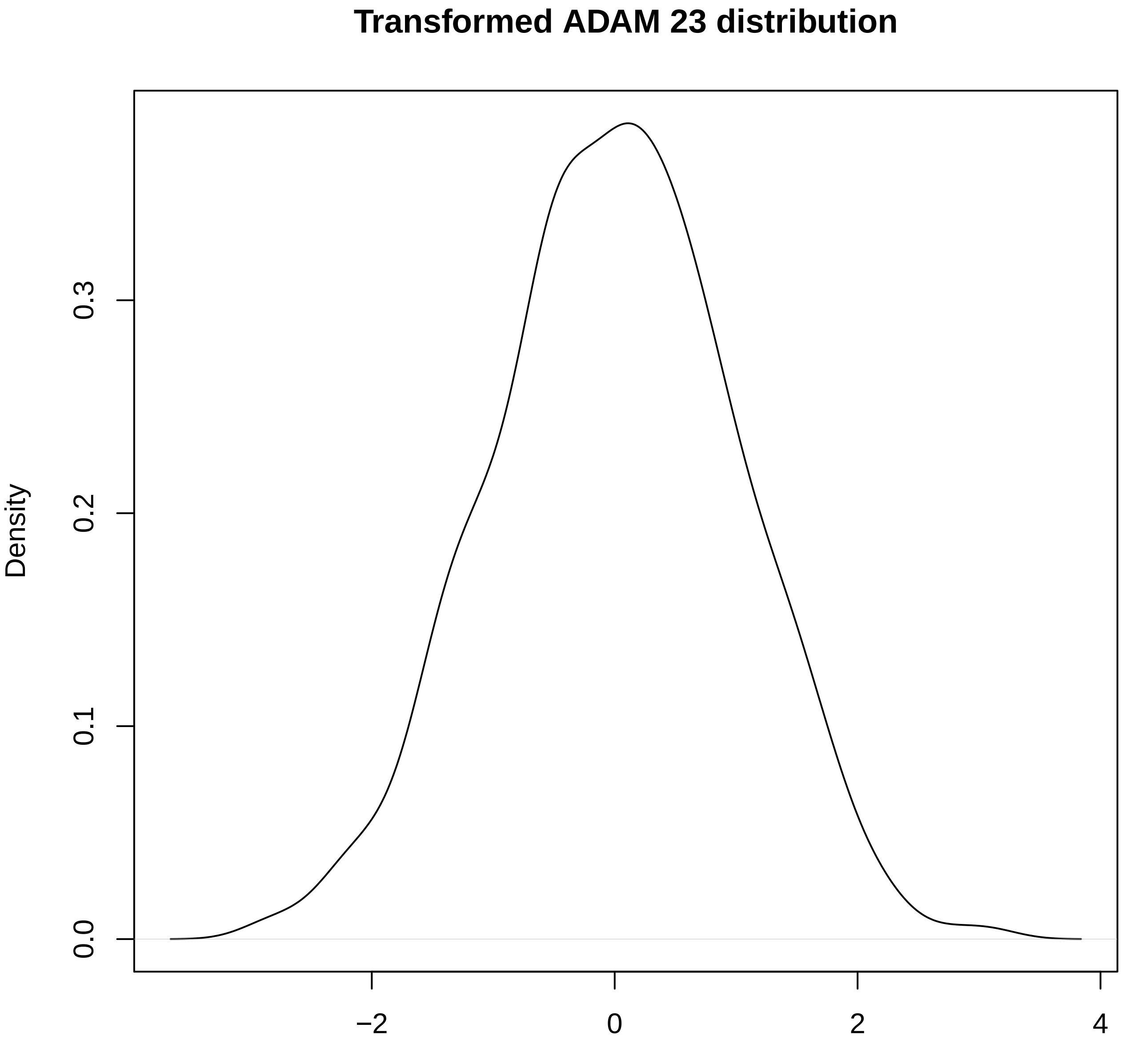

Transformed ADAM 23 distribution
Density
0.0
0.1
0.2
0.3
−2
0
2
4

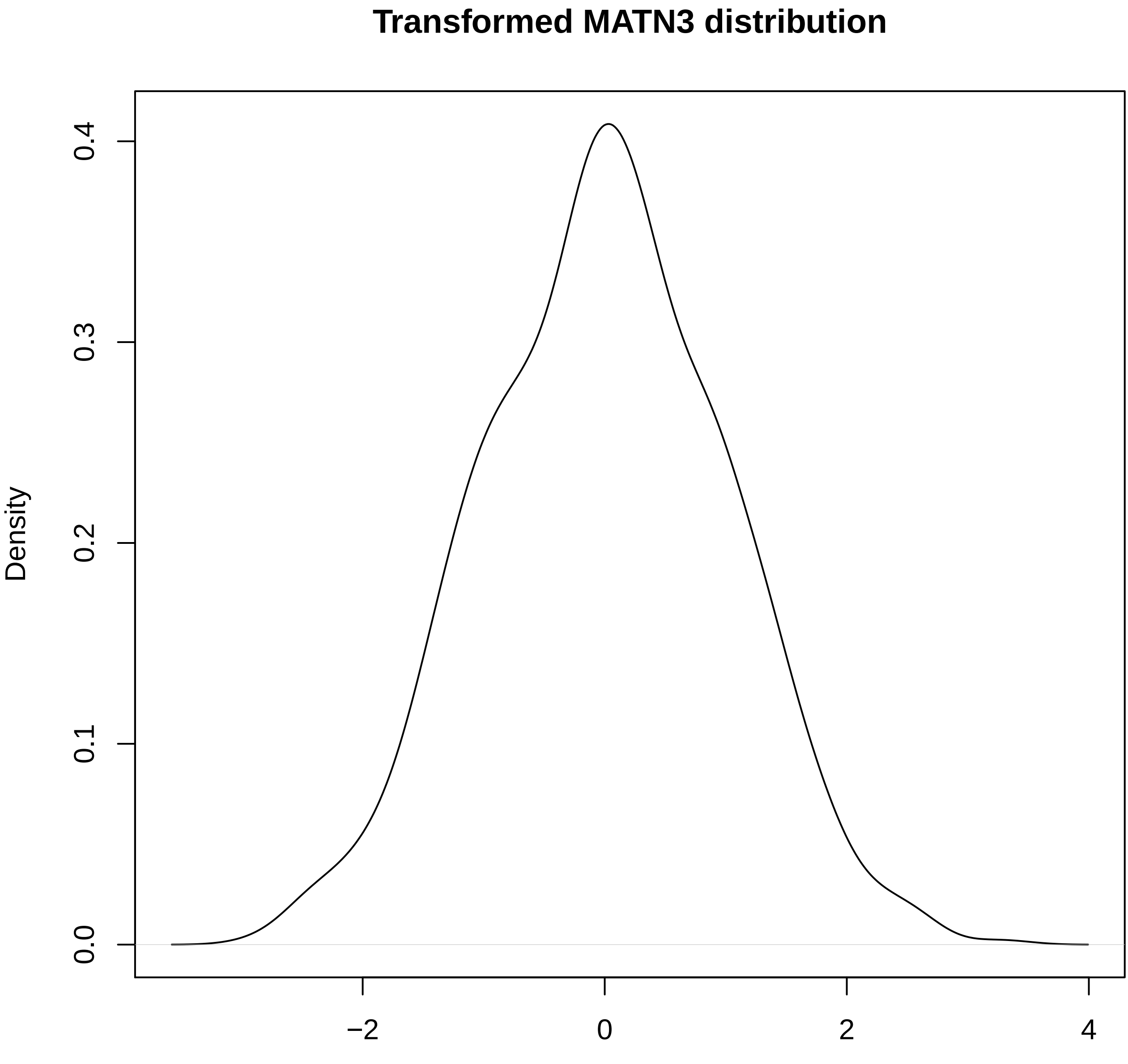

Transformed MATN3 distribution
Density
0.0
0.1
0.2
0.3
0.4
−2
0
2
4

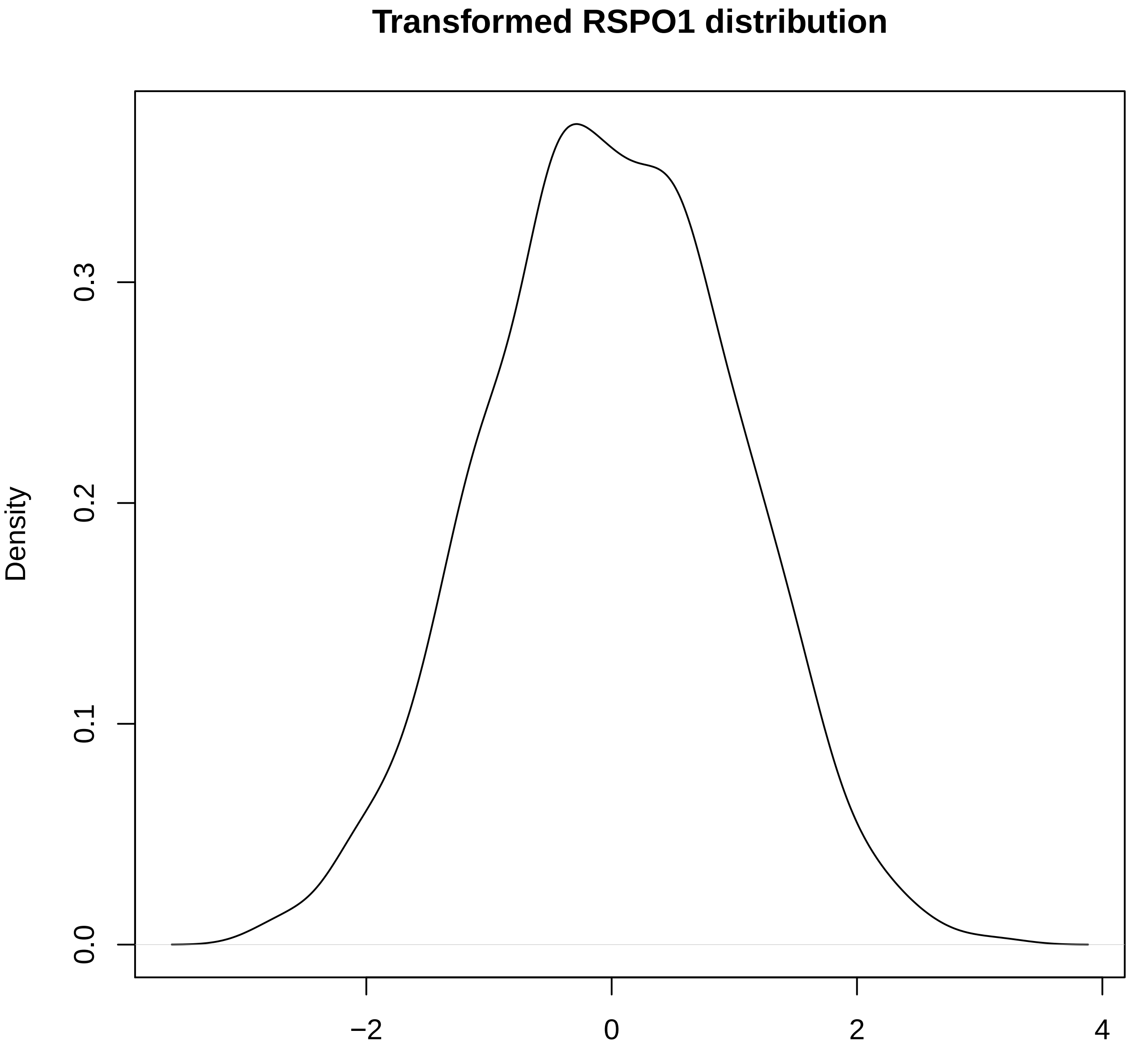

Transformed RSPO1 distribution
Density
0.0
0.1
0.2
0.3
−2
0
2
4

Transformed HAGH distribution

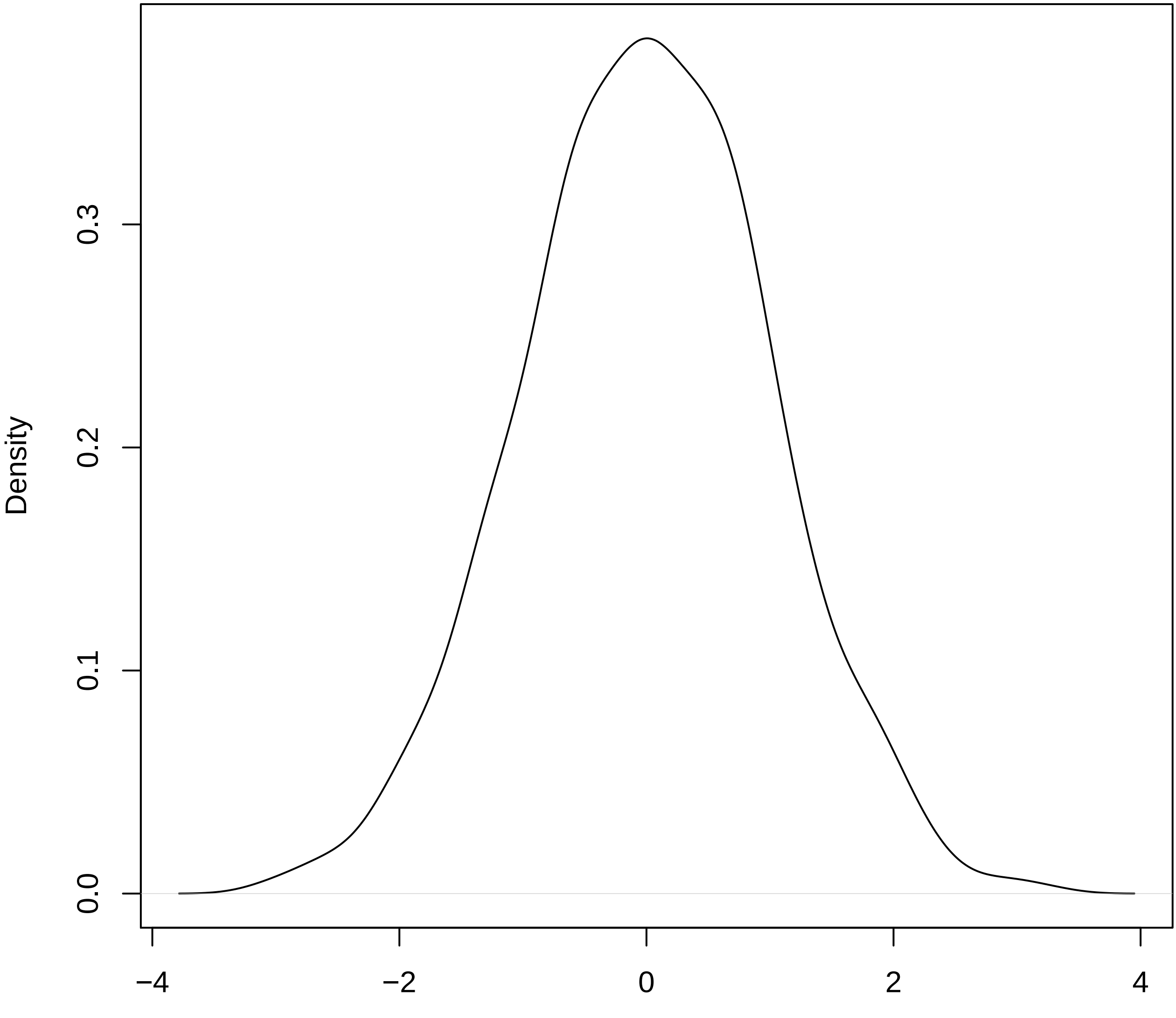

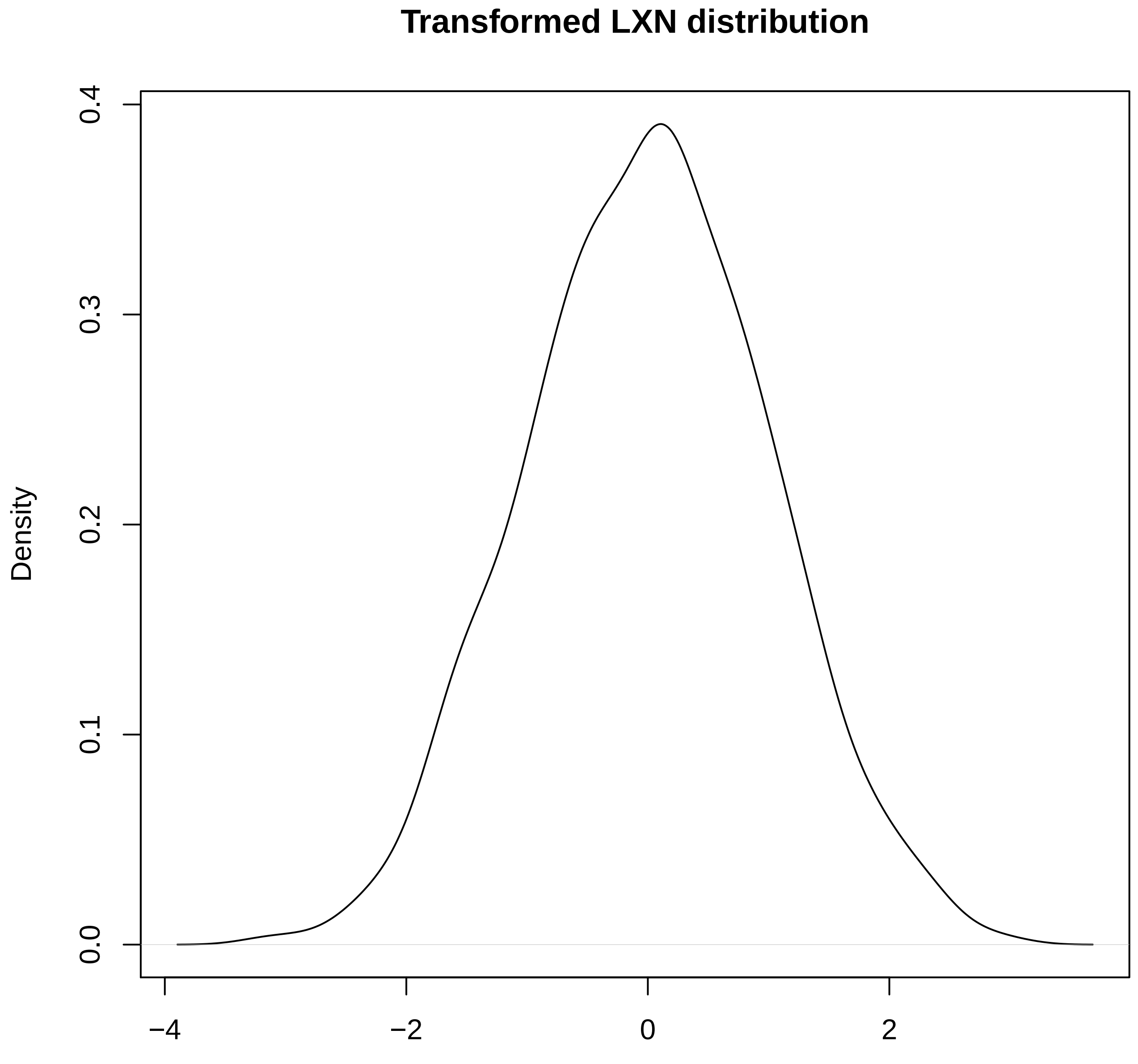
Transformed LXN distribution
Density
0.0
0.1
0.2
0.3
0.4
−4
−2
0
2

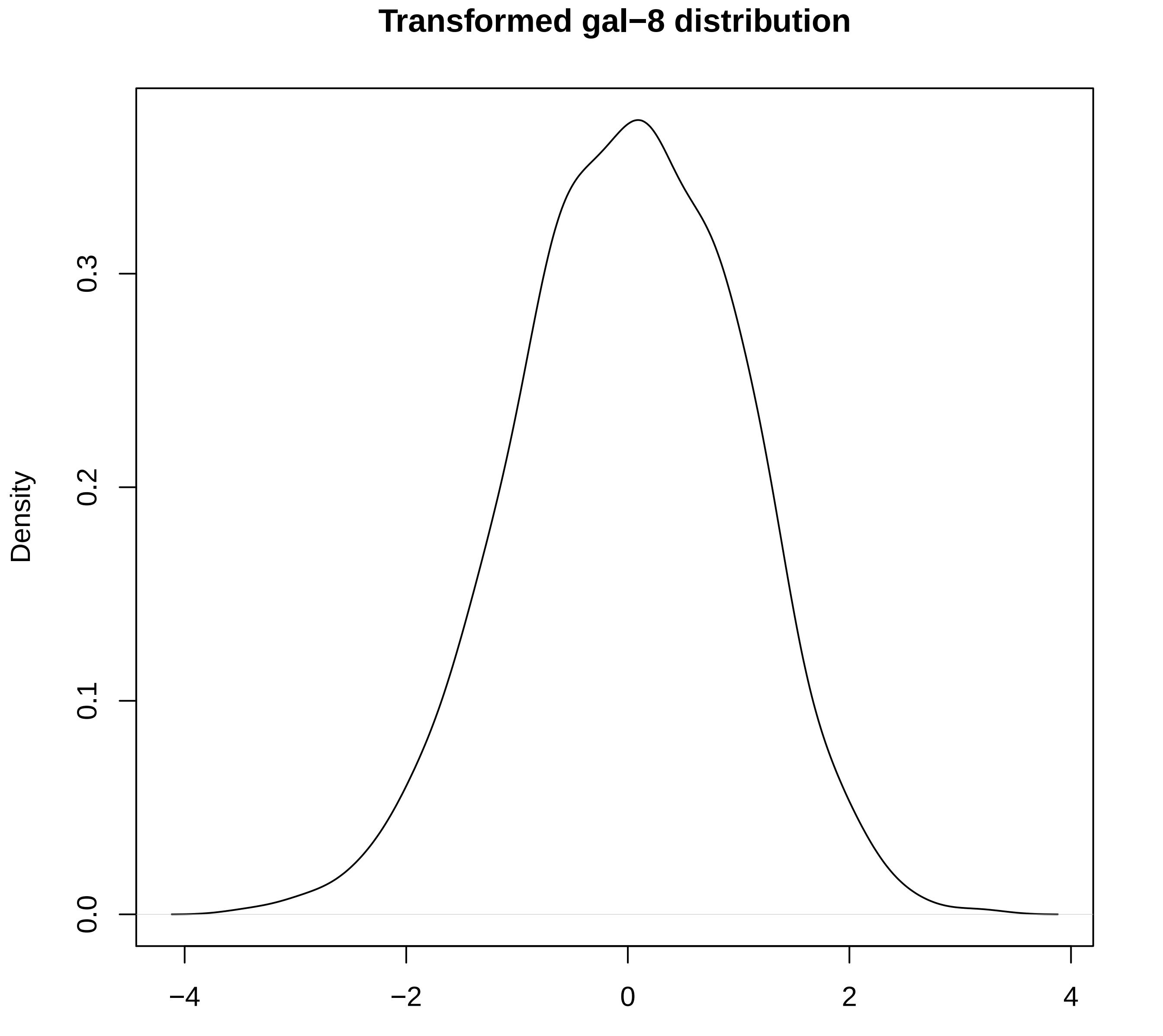
Transformed gal−8 distribution
Density
0.0
0.1
0.2
0.3
−4
−2
0
2
4

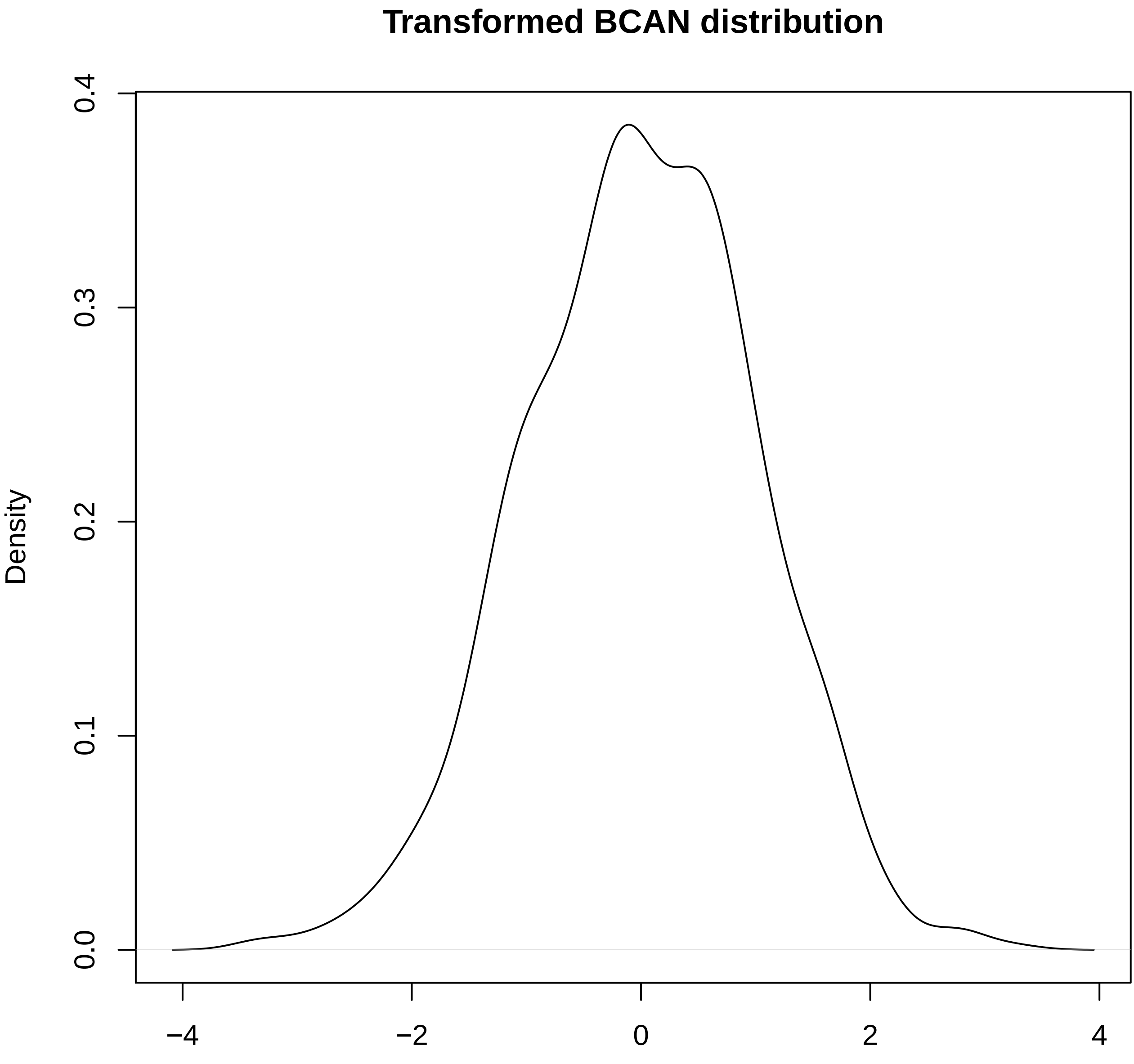

Transformed BCAN distribution
Density
0.0
0.1
0.2
0.3
0.4
−4
−2
0
2
4

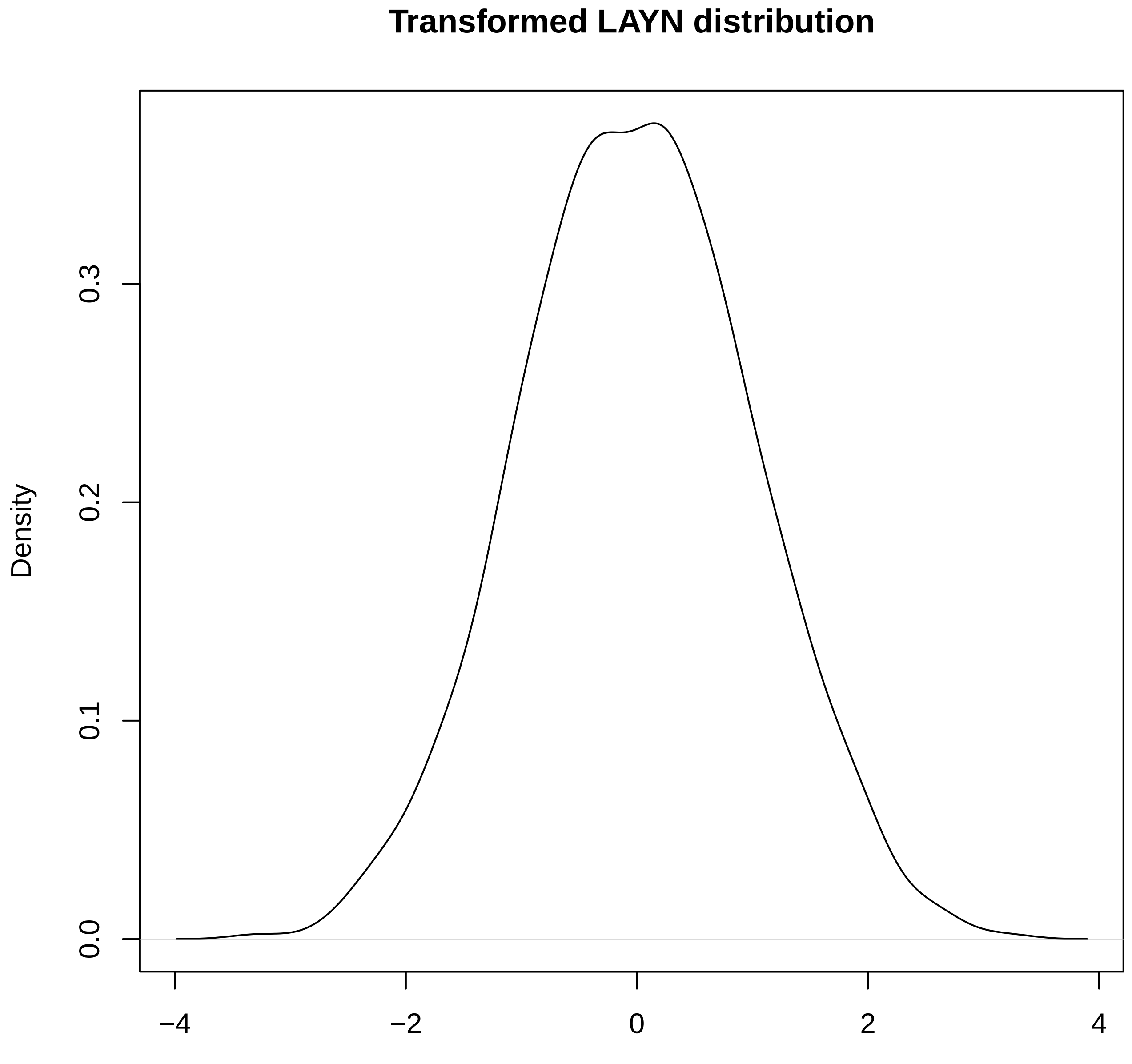

Transformed LAYN distribution
Density
0.0
0.1
0.2
0.3
−4
−2
0
2
4

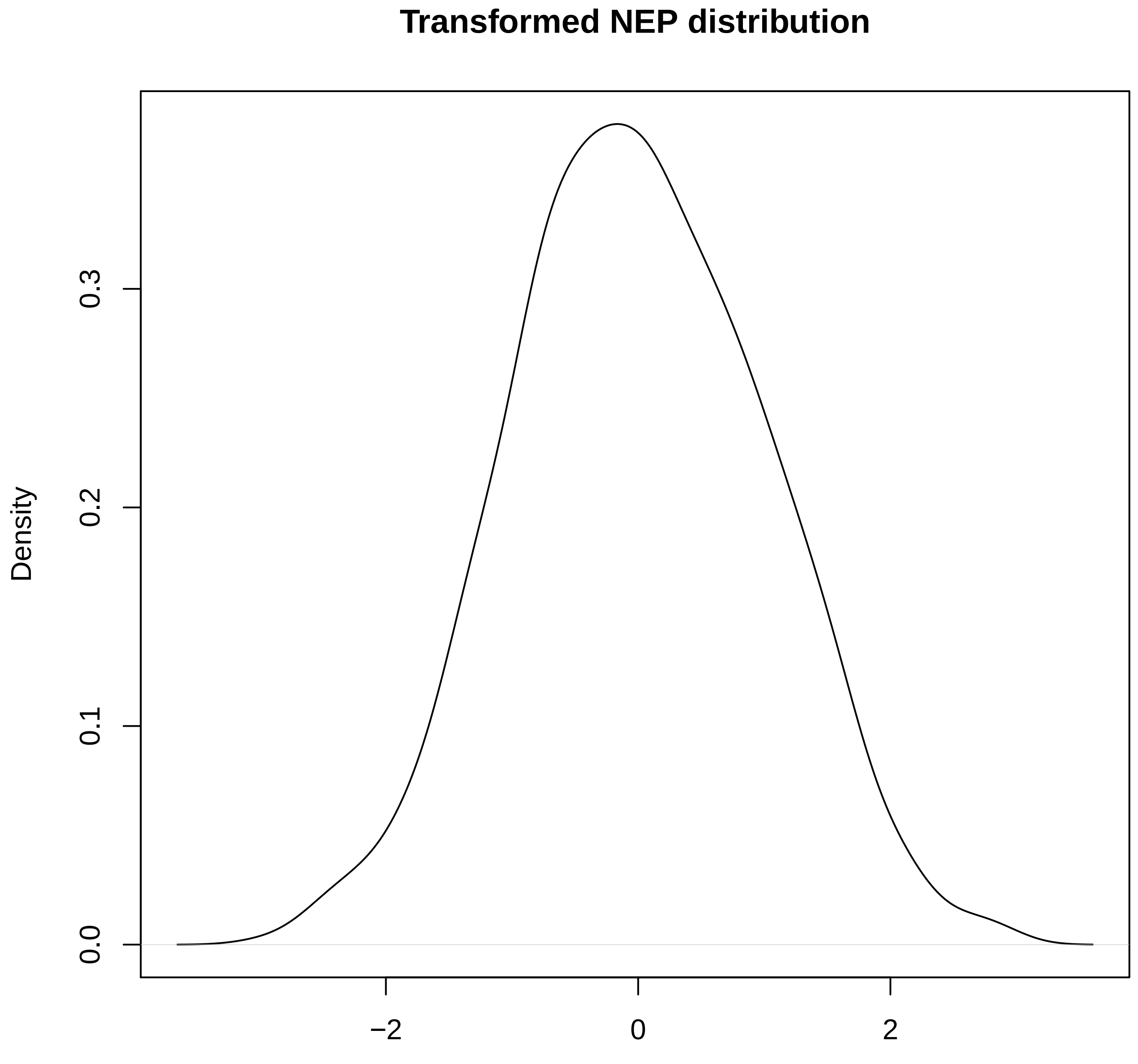
Transformed NEP distribution
Density
0.0
0.1
0.2
0.3
−2
0
2

Transformed GDF−8 distribution

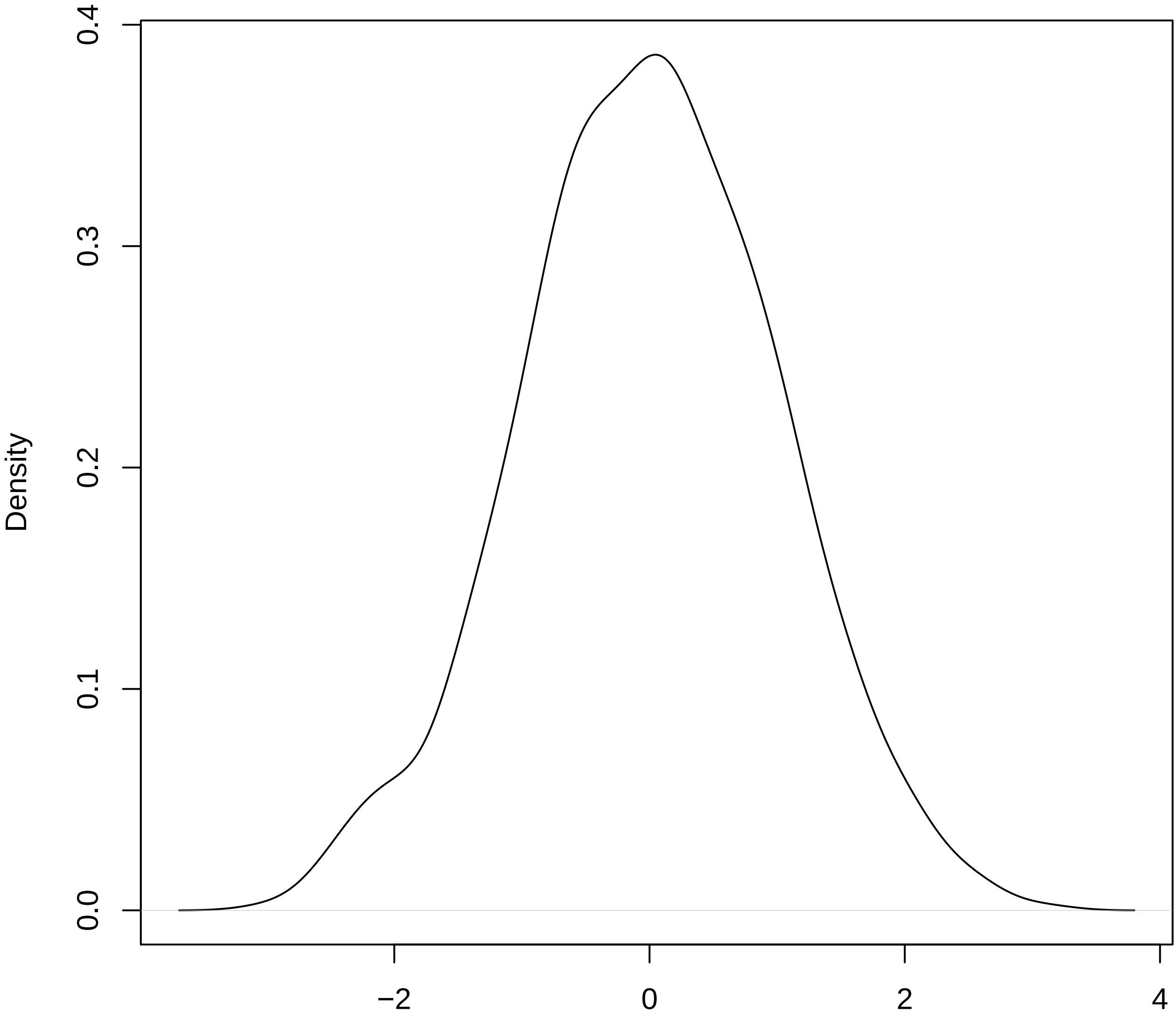

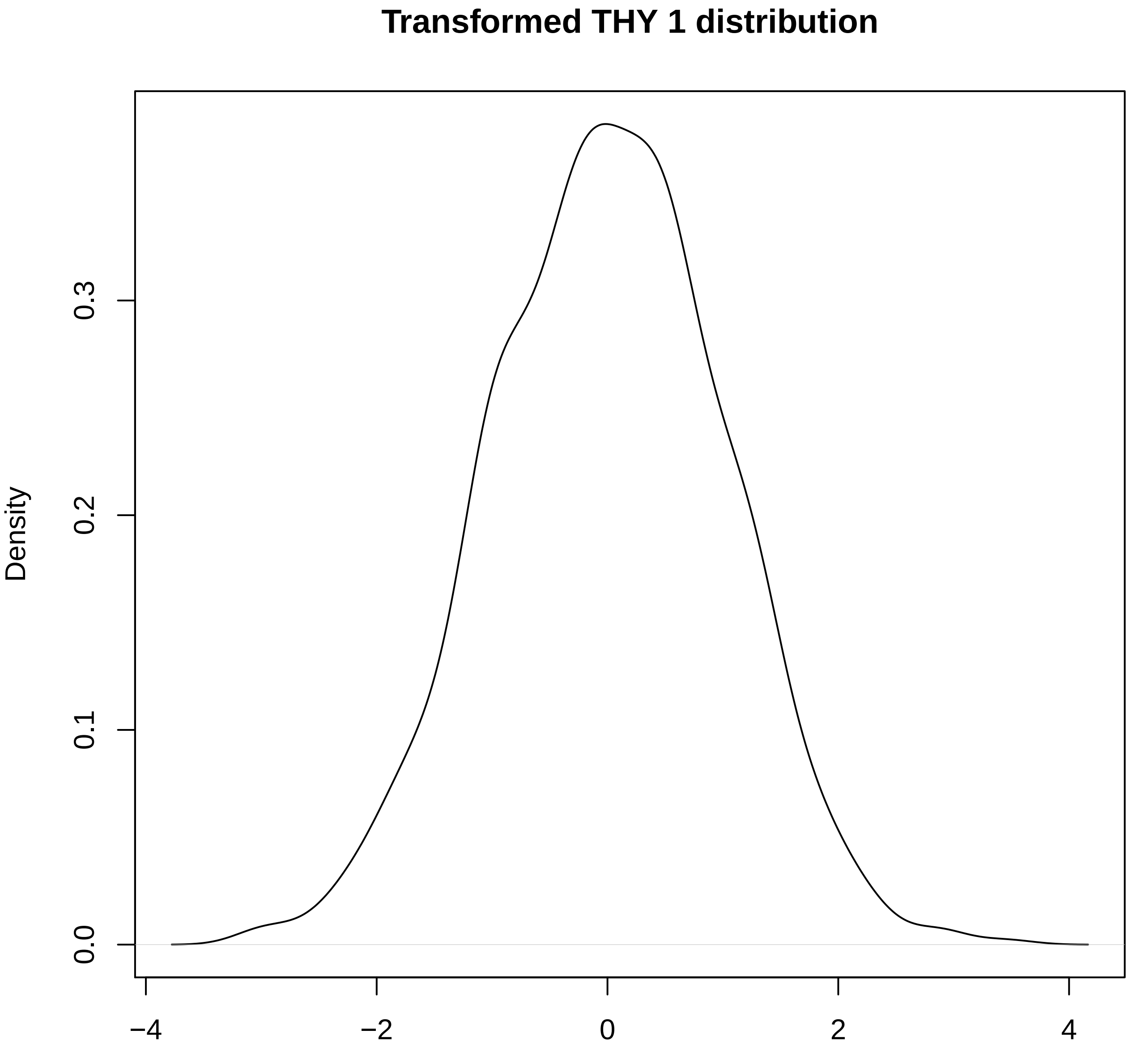

Transformed THY 1 distribution
Density
0.0
0.1
0.2
0.3
−4
−2
0
2
4

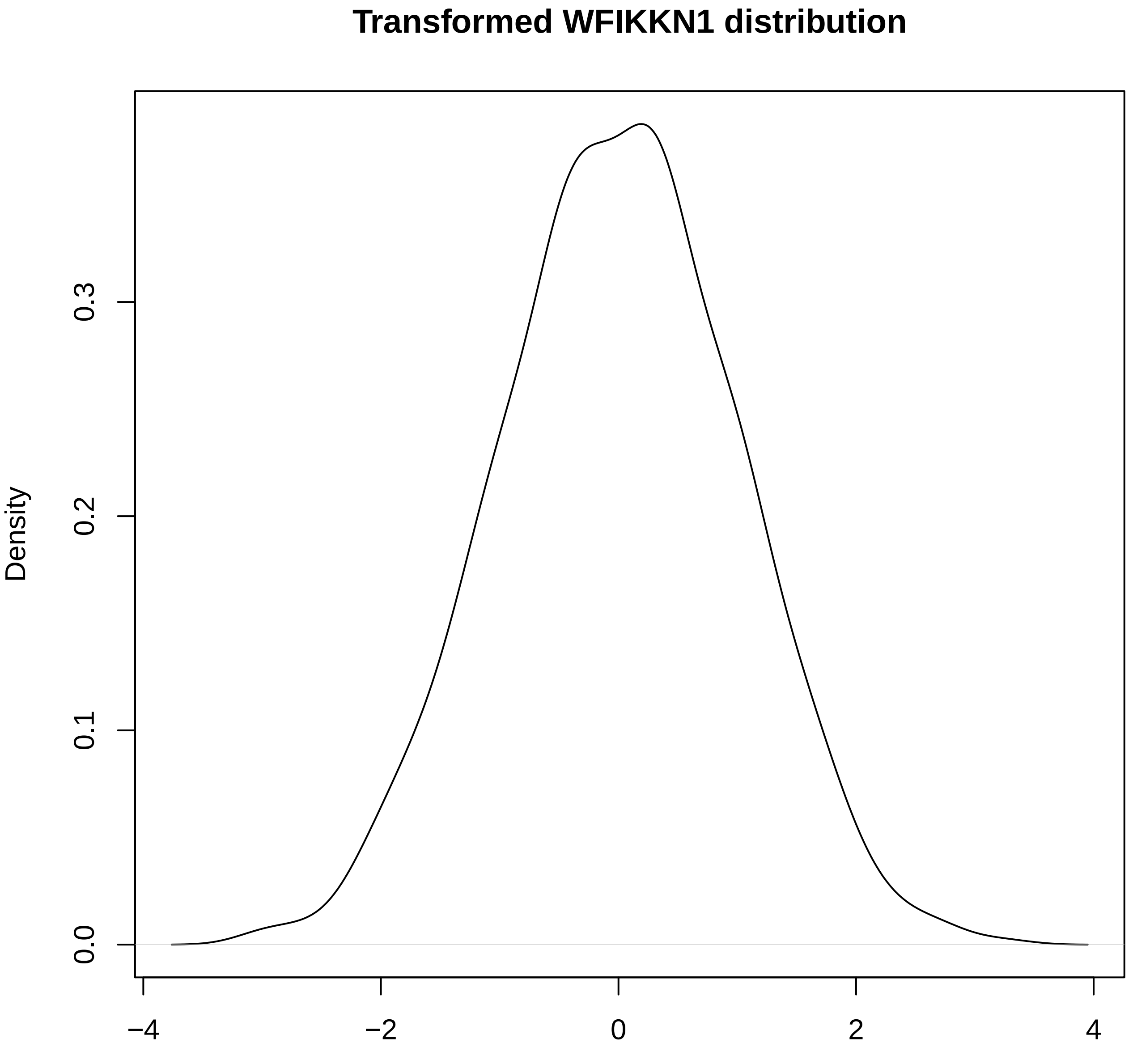
Transformed WFIKKN1 distribution
Density
0.0
0.1
0.2
0.3
−4
−2
0
2
4

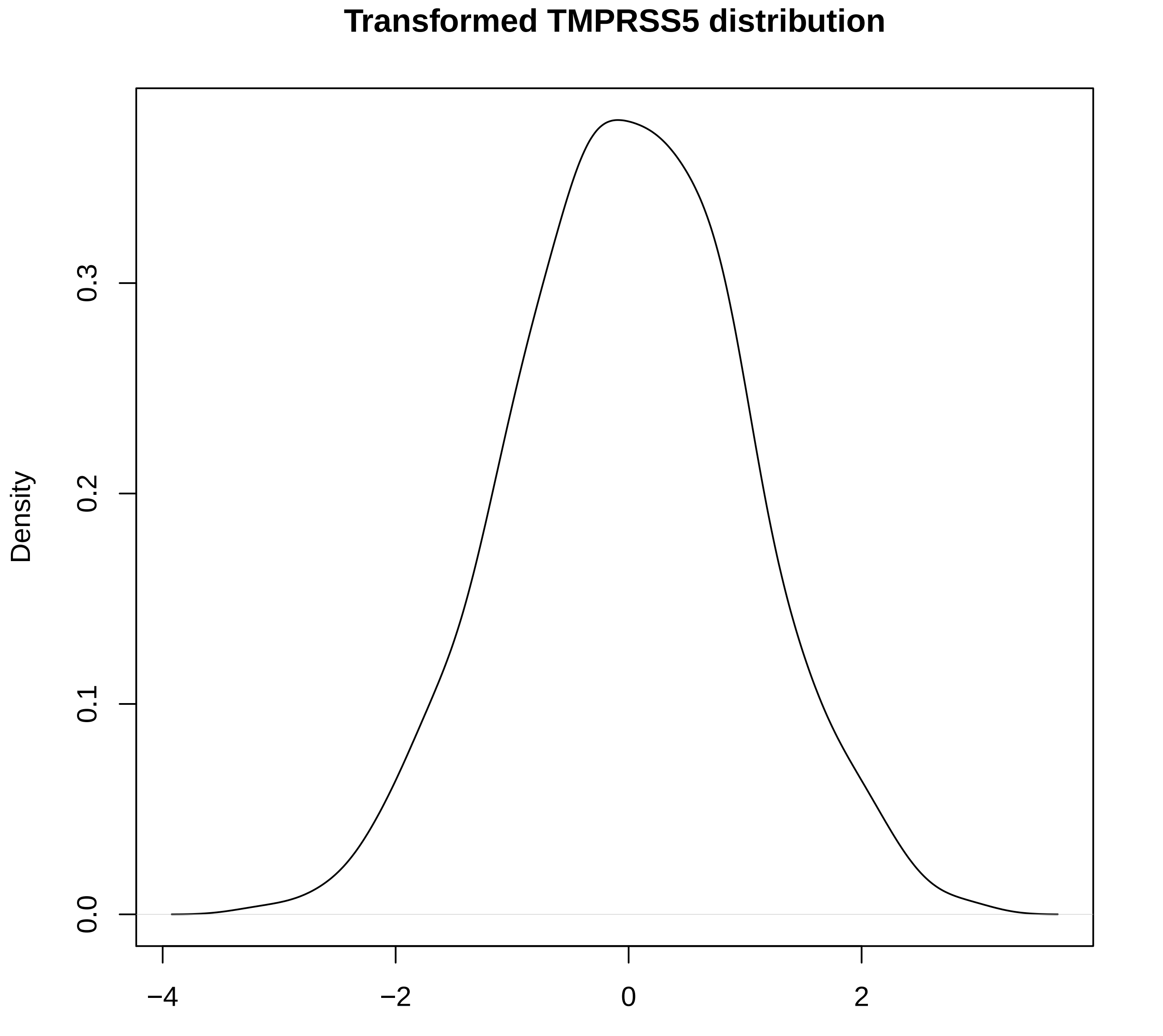
Transformed TMPRSS5 distribution
Density
0.0
0.1
0.2
0.3
−4
−2
0
2

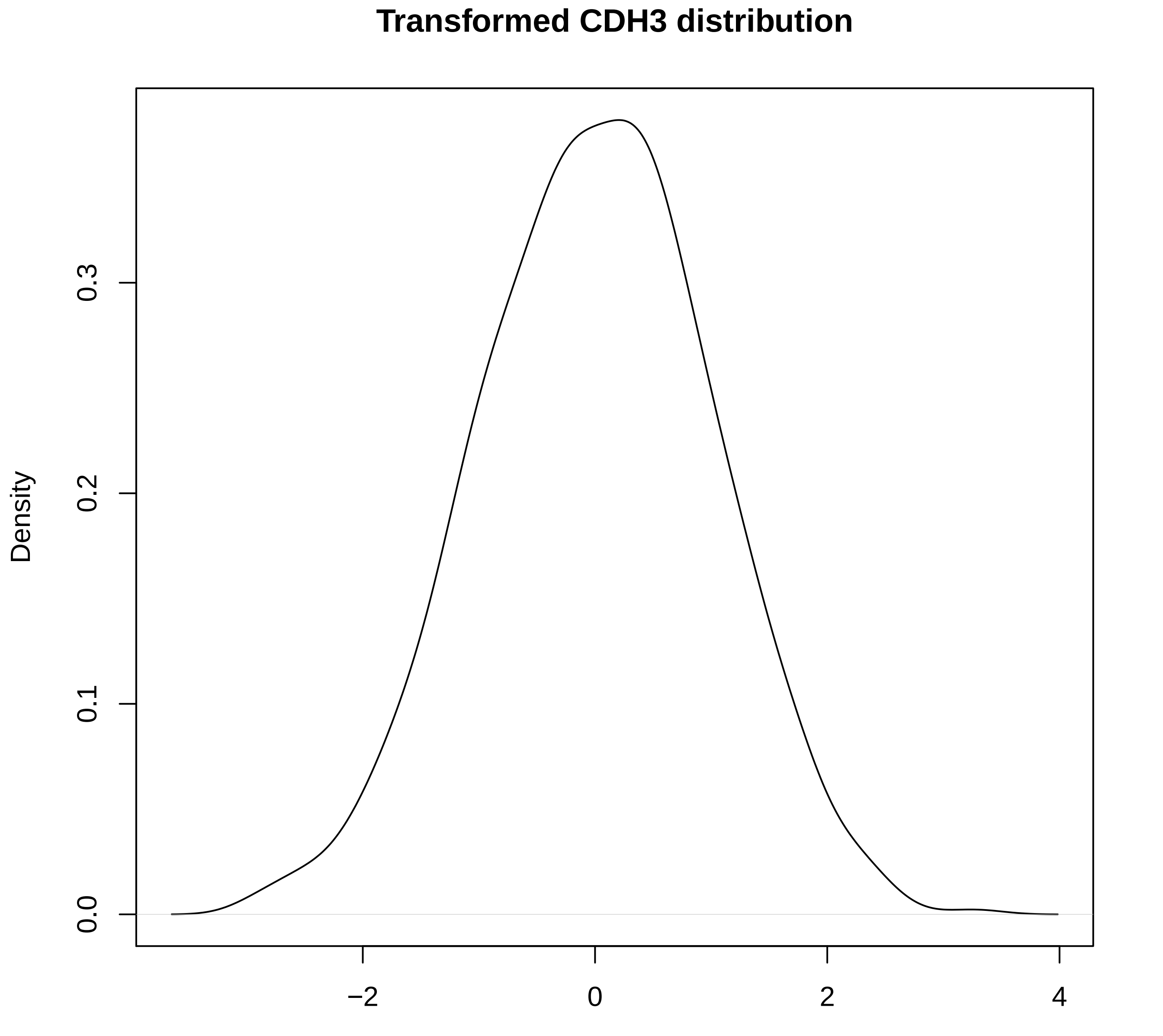
Transformed CDH3 distribution
Density
0.0
0.1
0.2
0.3
−2
0
2
4

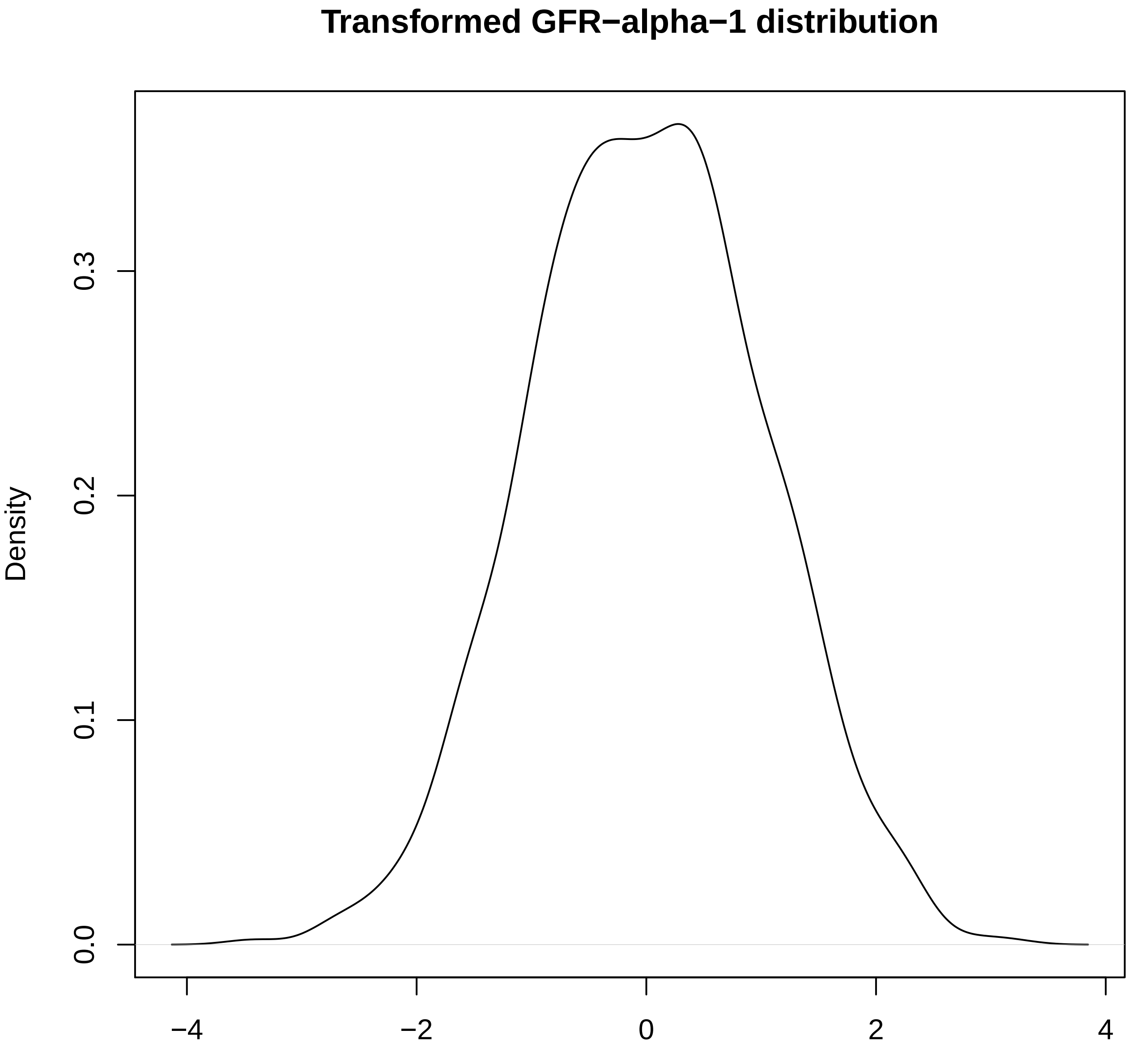
Transformed GFR−alpha−1 distribution
Density
0.0
0.1
0.2
0.3
−4
−2
0
2
4

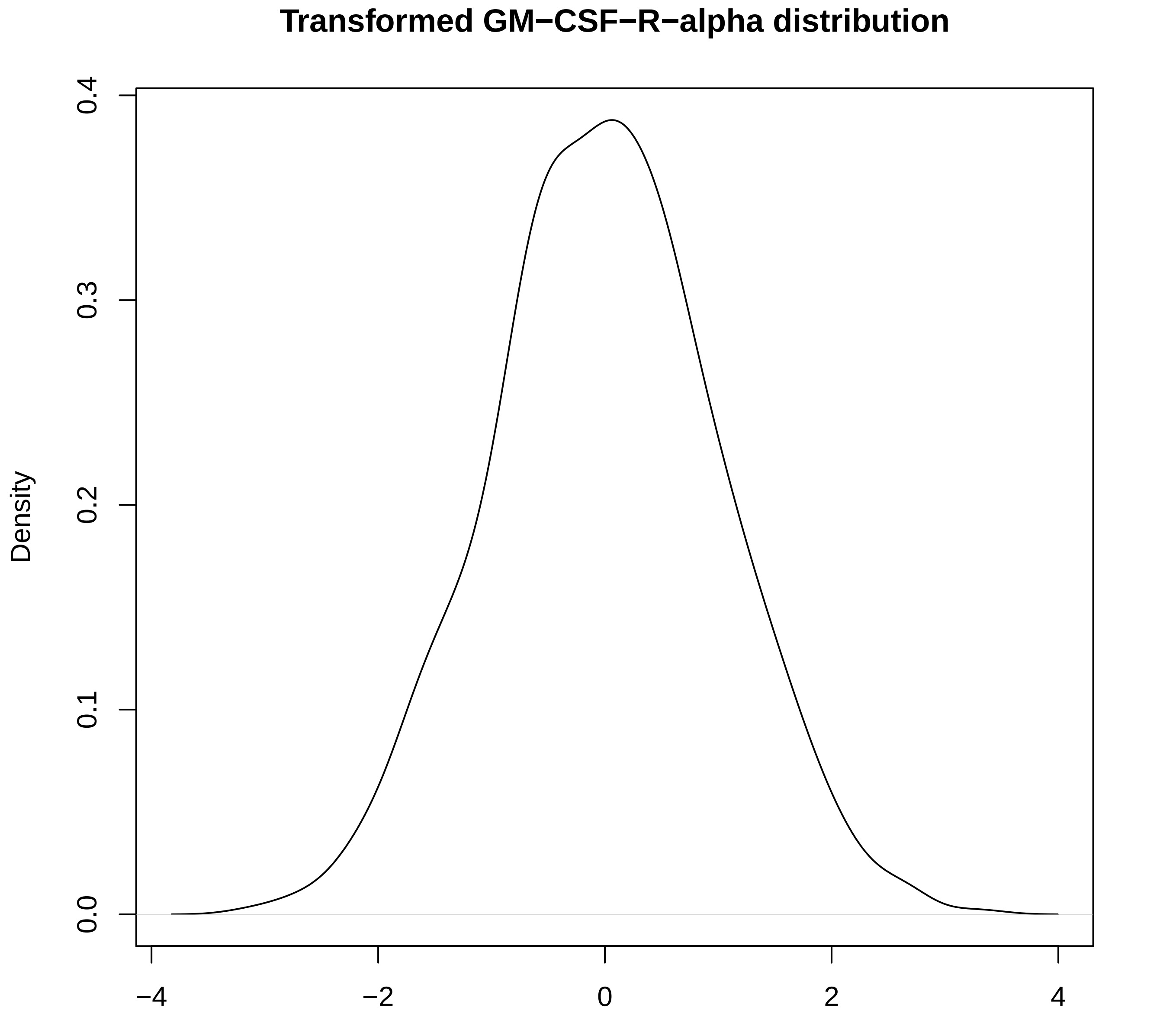
Transformed GM−CSF−R−alpha distribution
Density
0.0
0.1
0.2
0.3
0.4
−4
−2
0
2
4

Transformed Beta−NGF distribution

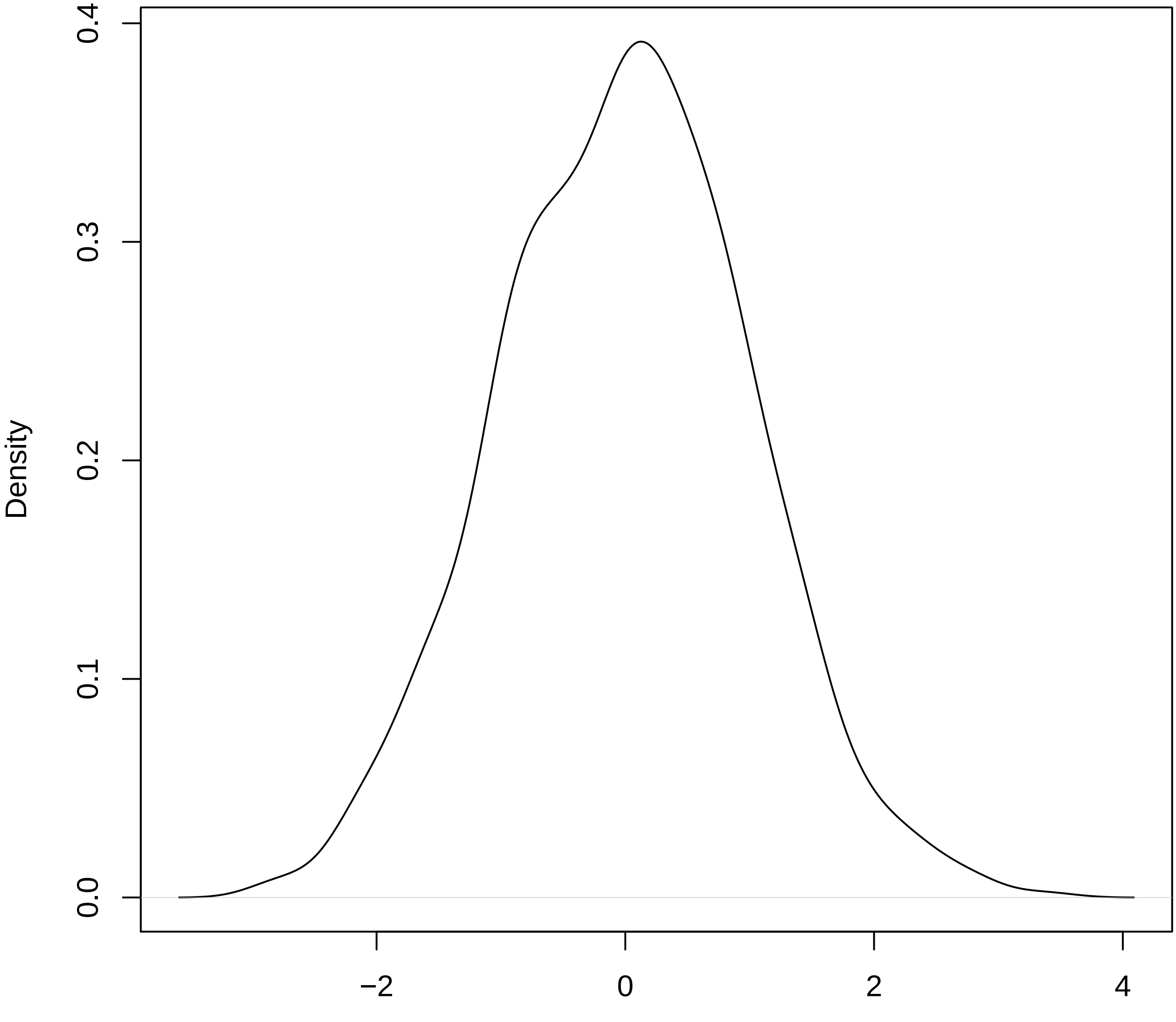

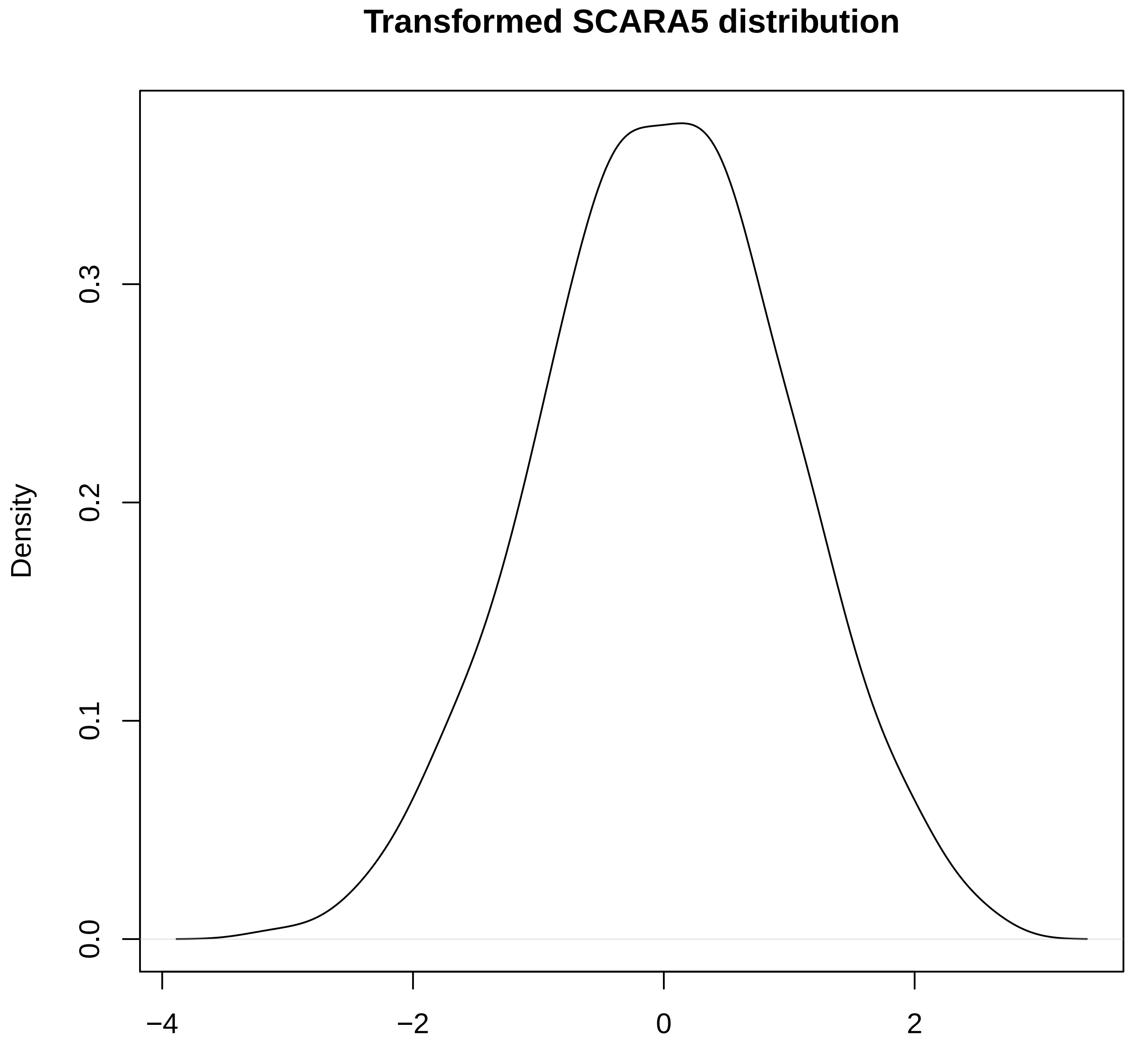

Transformed SCARA5 distribution
Density
0.0
0.1
0.2
0.3
−4
−2
0
2

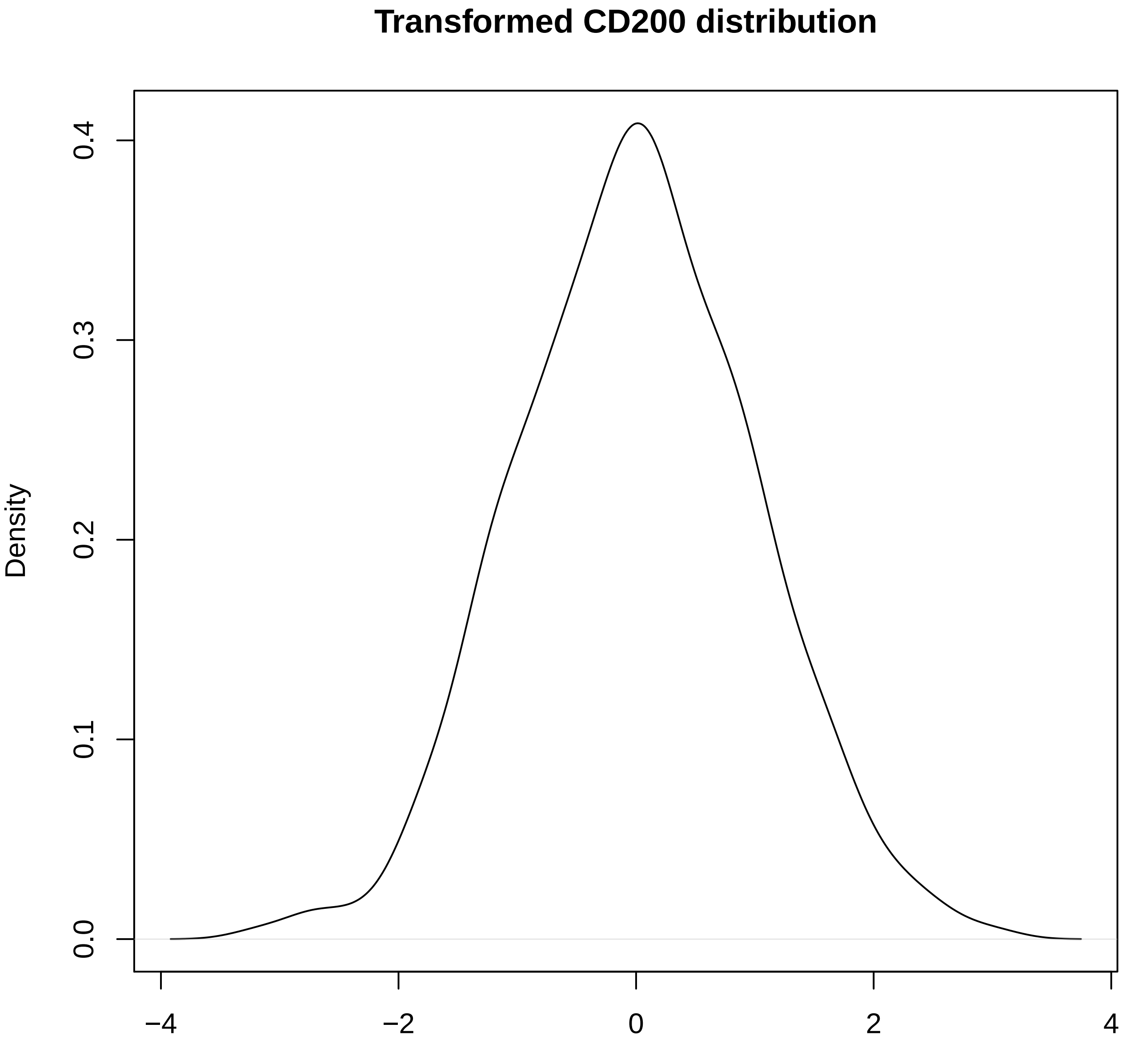
Transformed CD200 distribution
Density
0.0
0.1
0.2
0.3
0.4
−4
−2
0
2
4

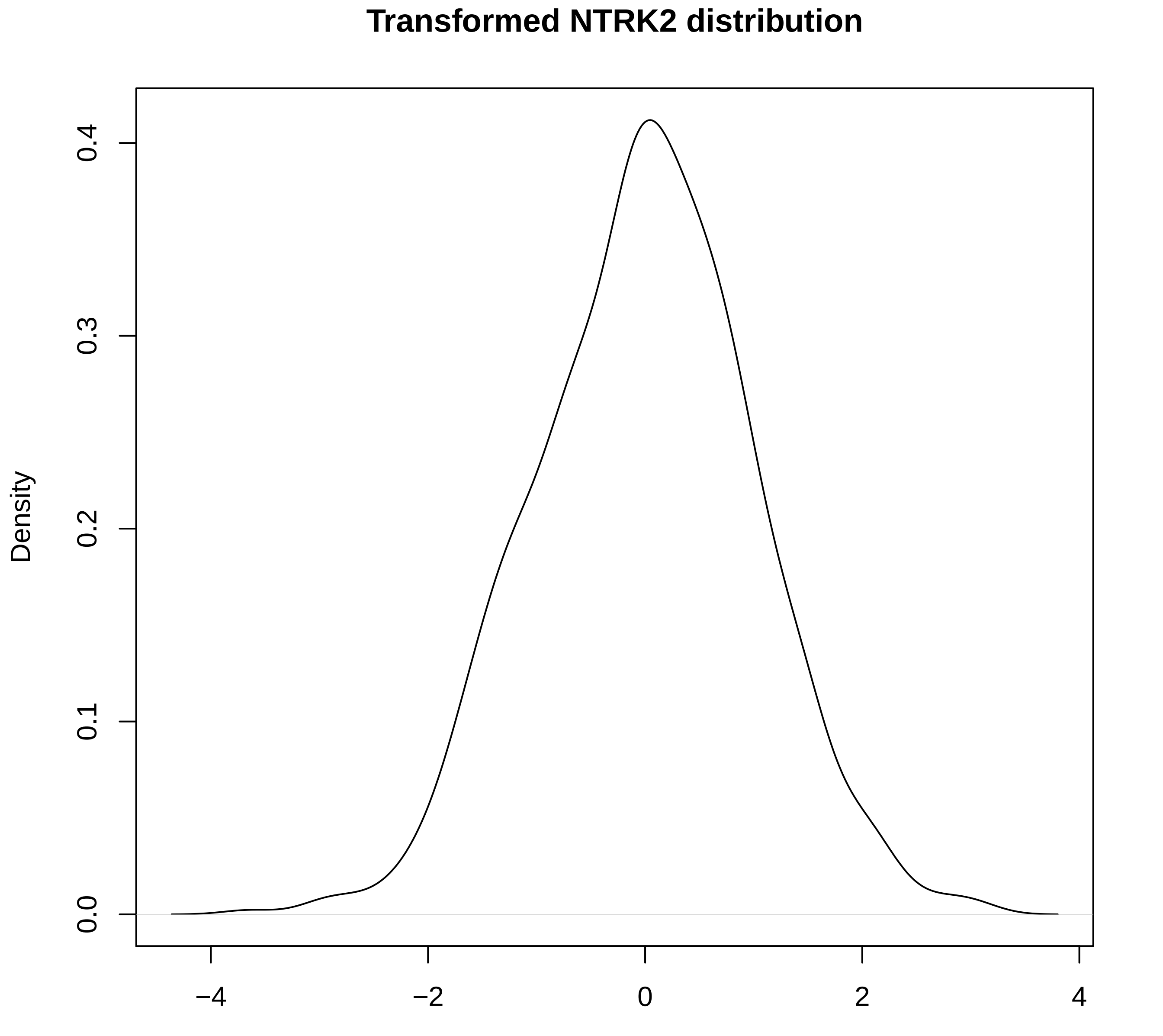
Transformed NTRK2 distribution
Density
0.0
0.1
0.2
0.3
0.4
−4
−2
0
2
4

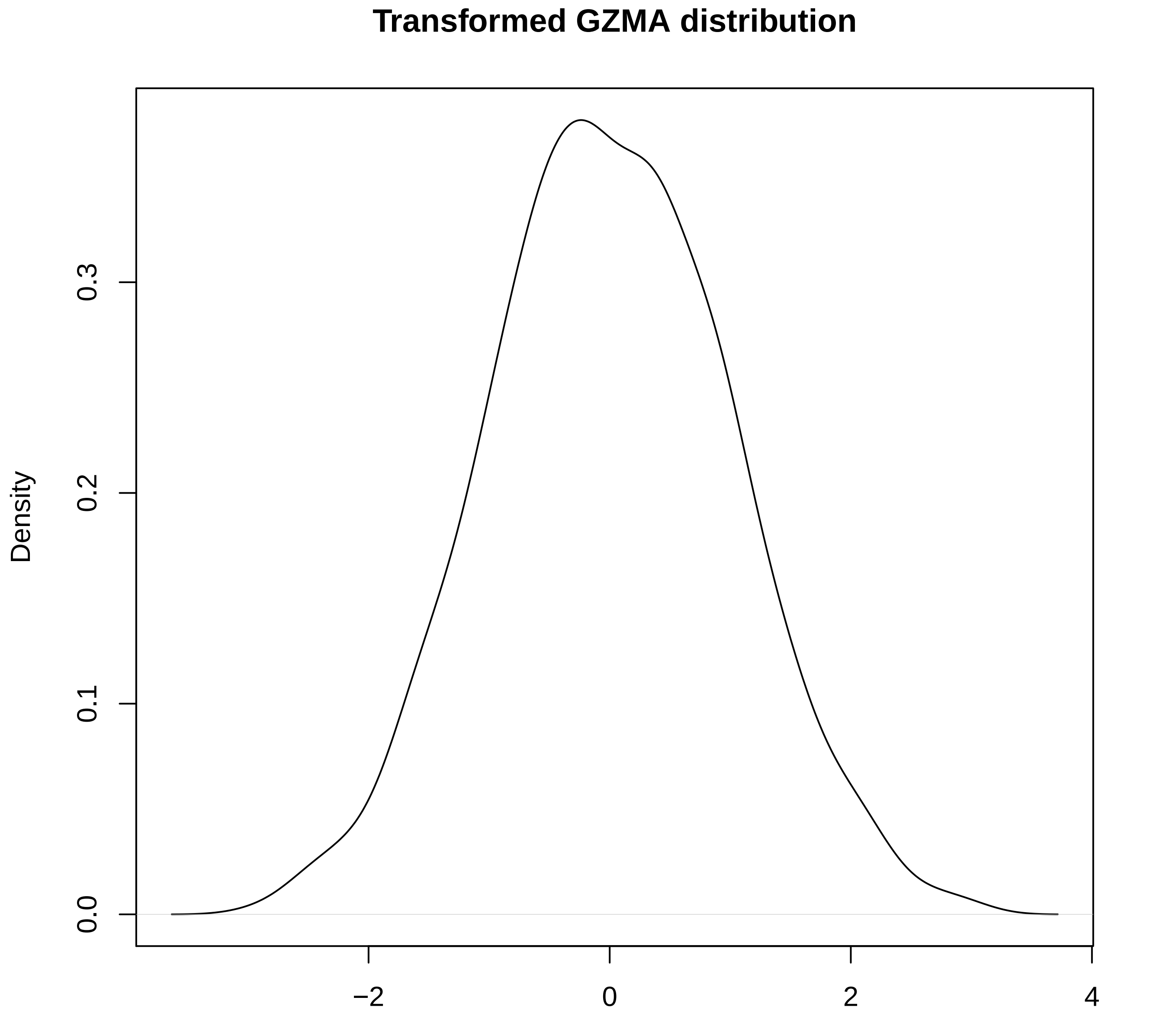
Transformed GZMA distribution
Density
0.0
0.1
0.2
0.3
−2
0
2
4

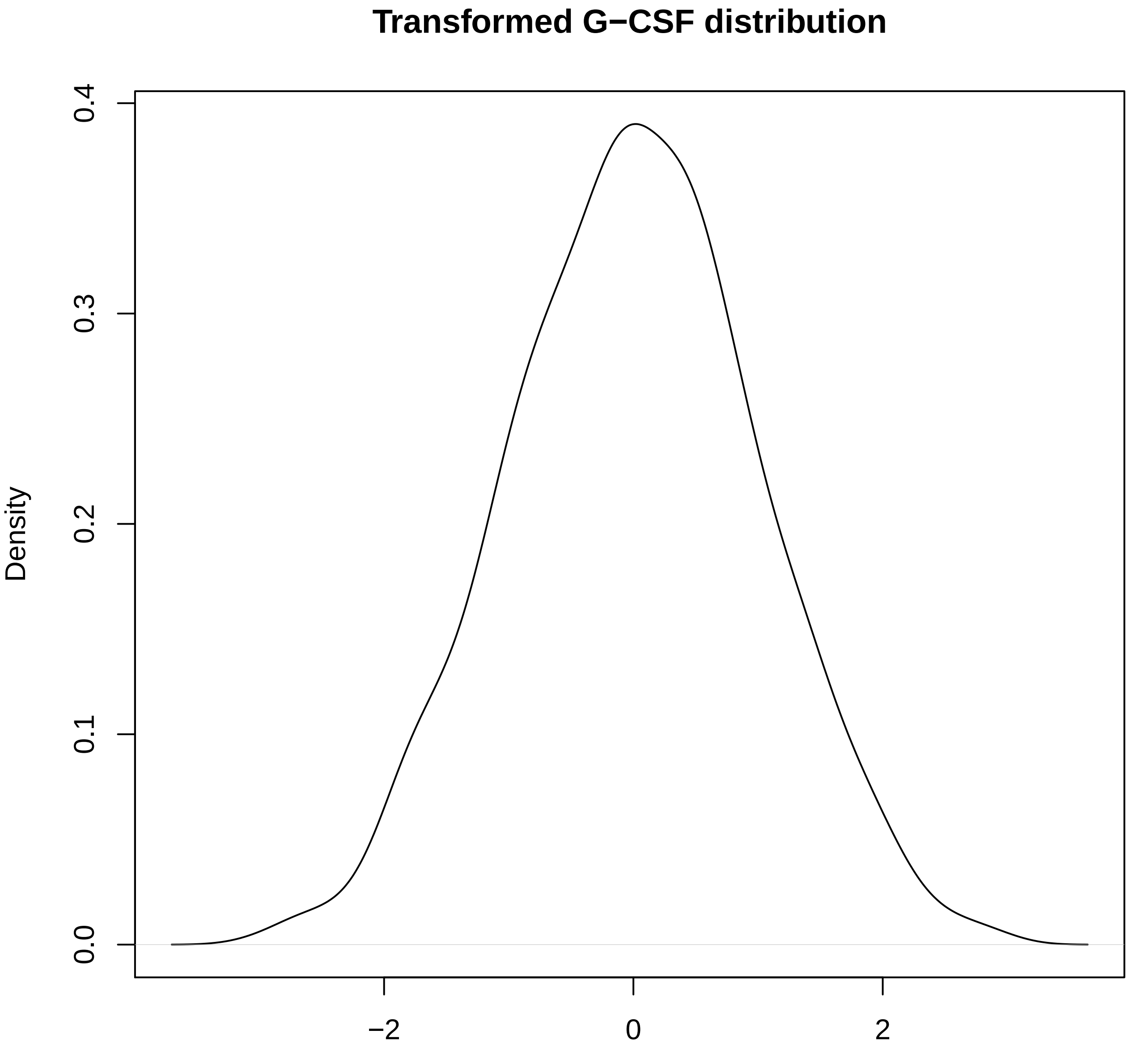
Transformed G−CSF distribution
Density
0.0
0.1
0.2
0.3
0.4
−2
0
2

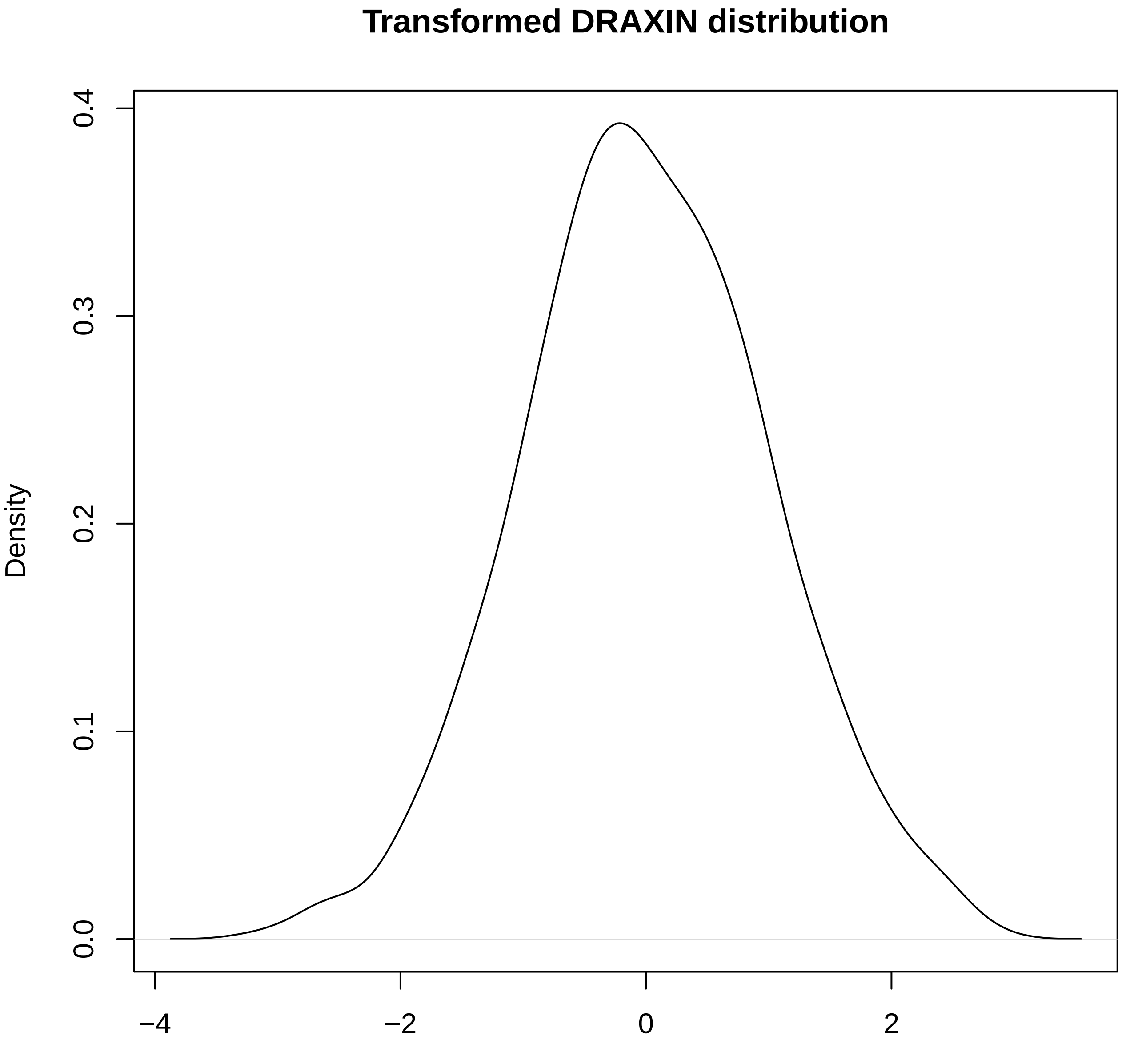

Transformed DRAXIN distribution
Density
0.0
0.1
0.2
0.3
0.4
−4
−2
0
2

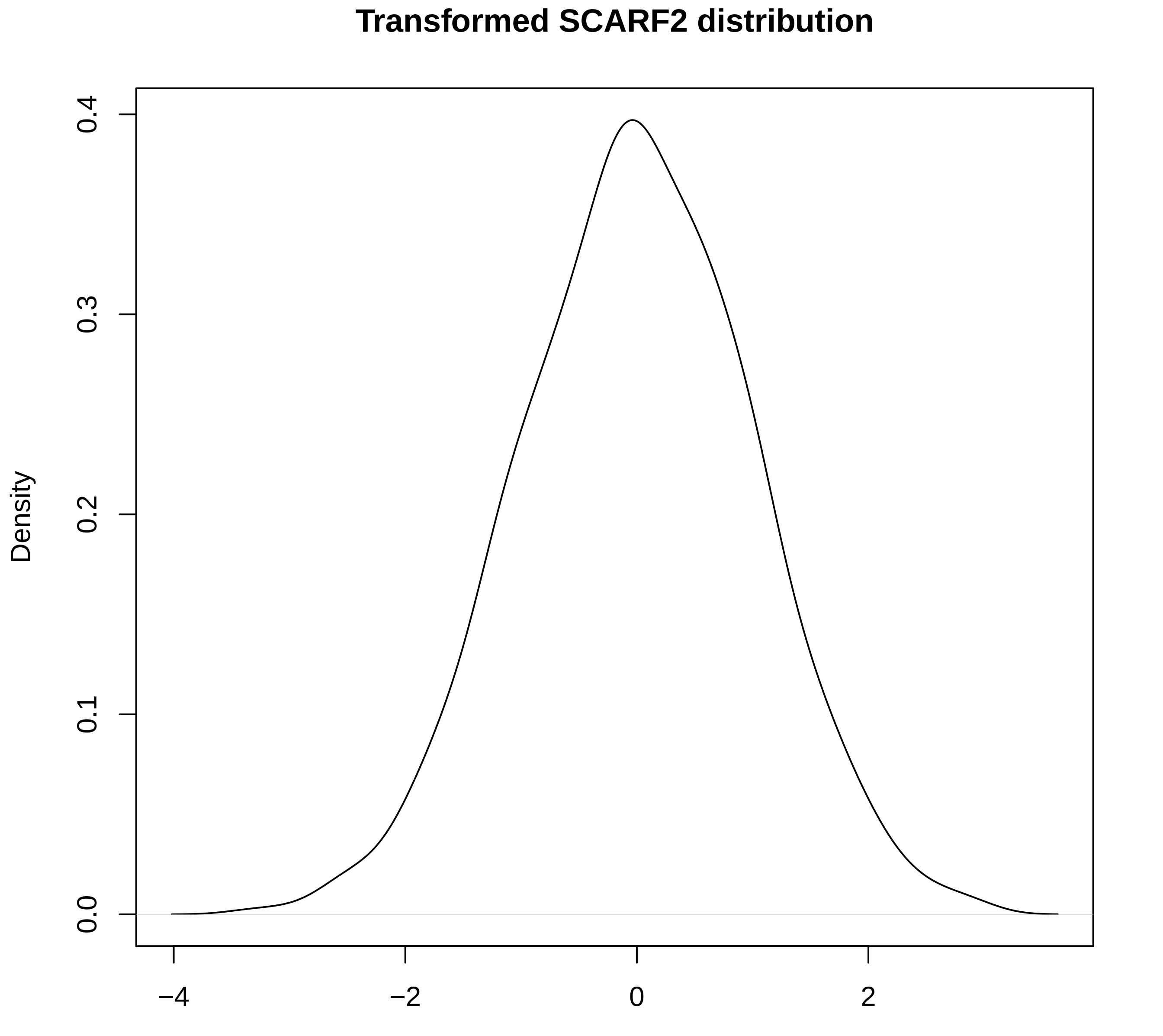

Transformed SCARF2 distribution
Density
0.0
0.1
0.2
0.3
0.4
−4
−2
0
2

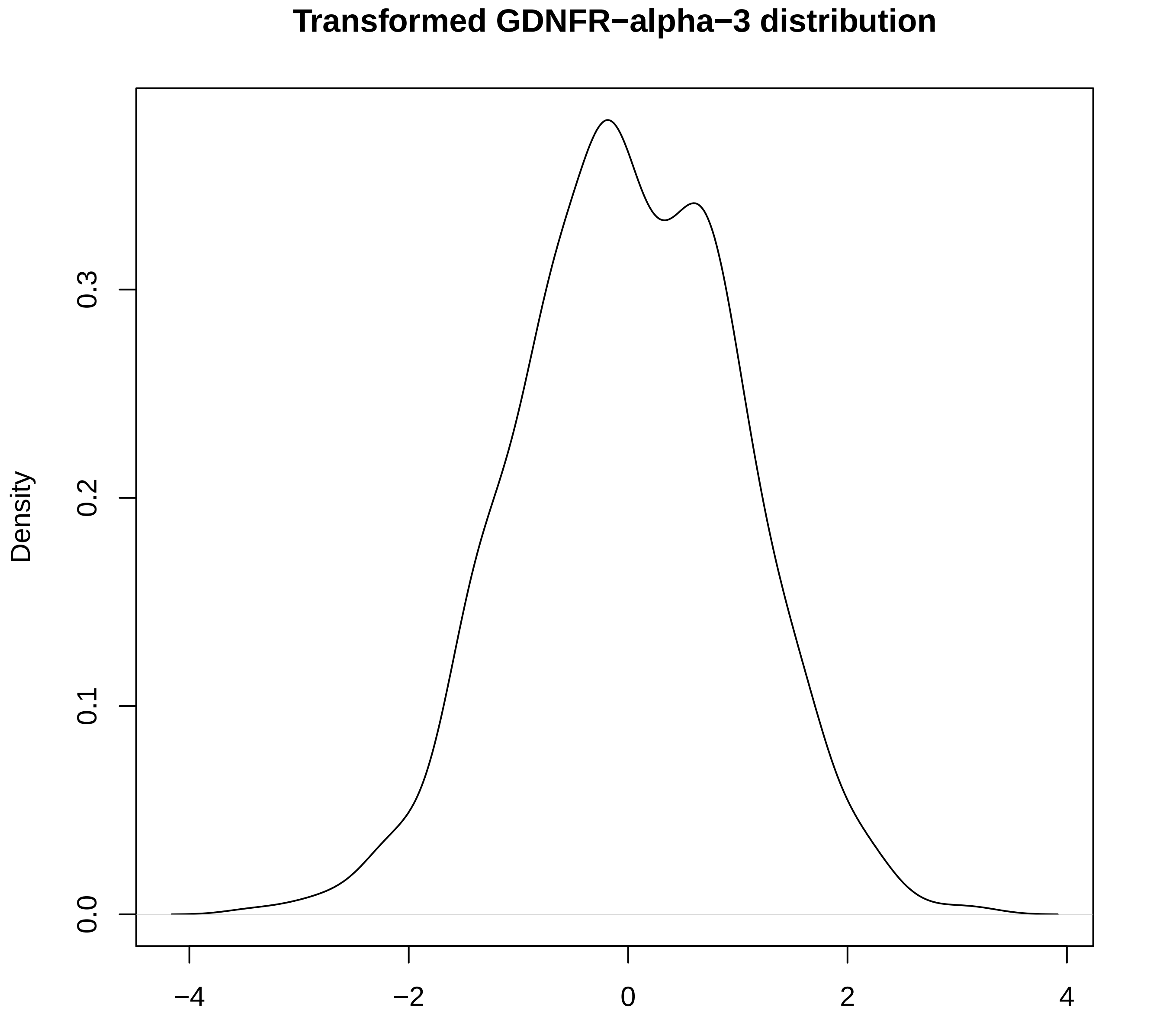

Transformed GDNFR−alpha−3 distribution
Density
0.0
0.1
0.2
0.3
−4
−2
0
2
4

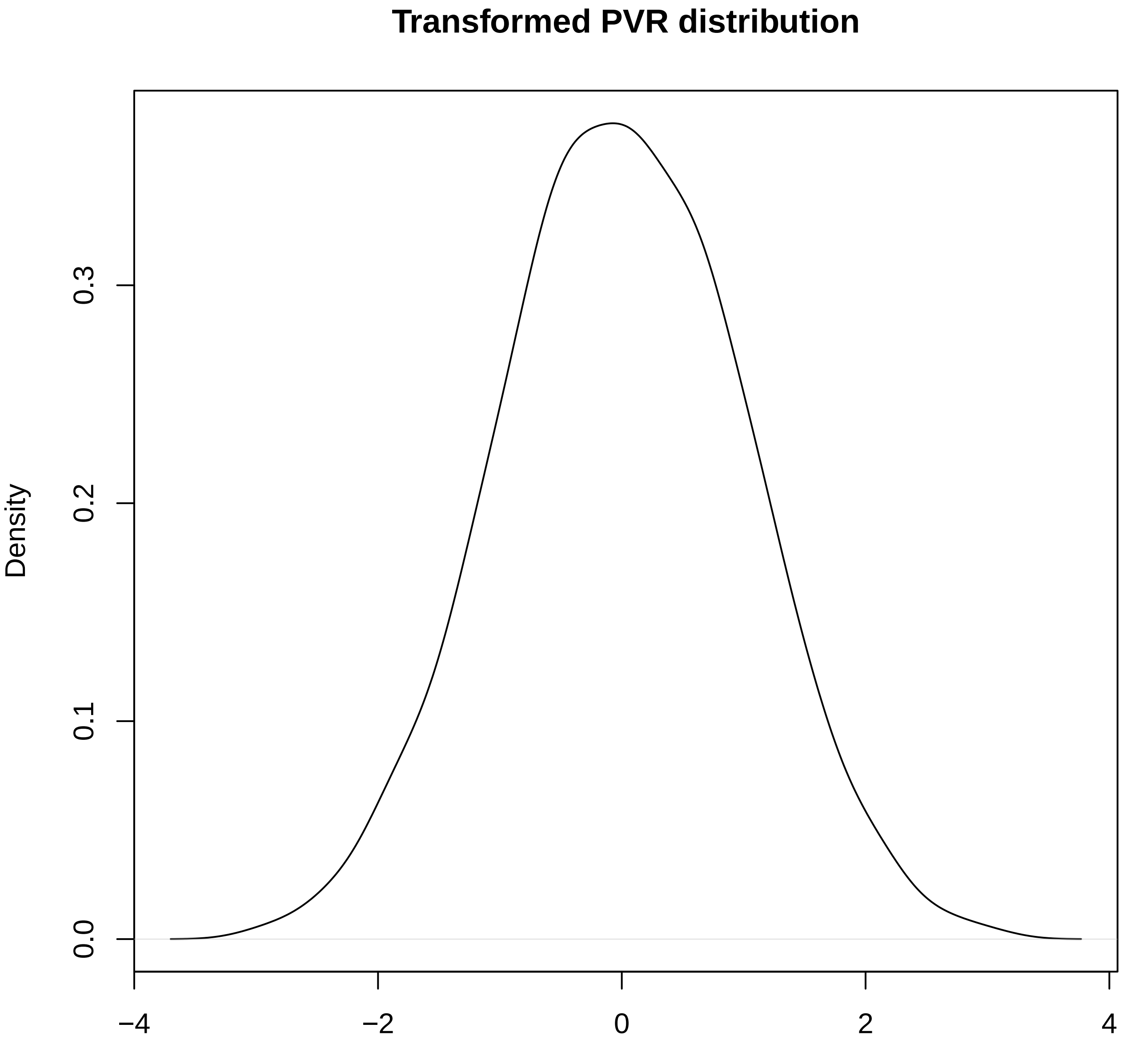
Transformed PVR distribution
Density
0.0
0.1
0.2
0.3
−4
−2
0
2
4

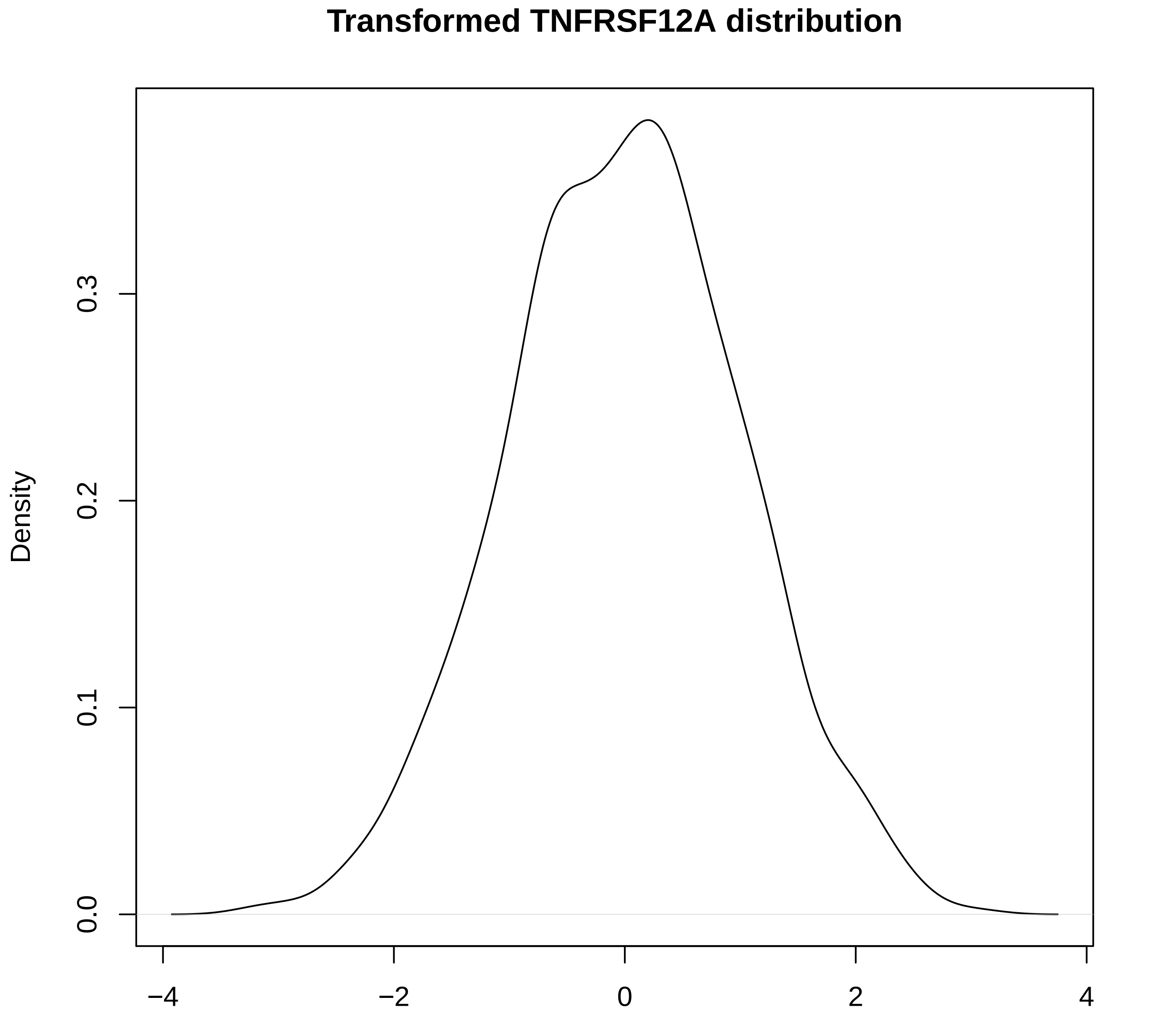

Transformed TNFRSF12A distribution
Density
0.0
0.1
0.2
0.3
−4
−2
0
2
4

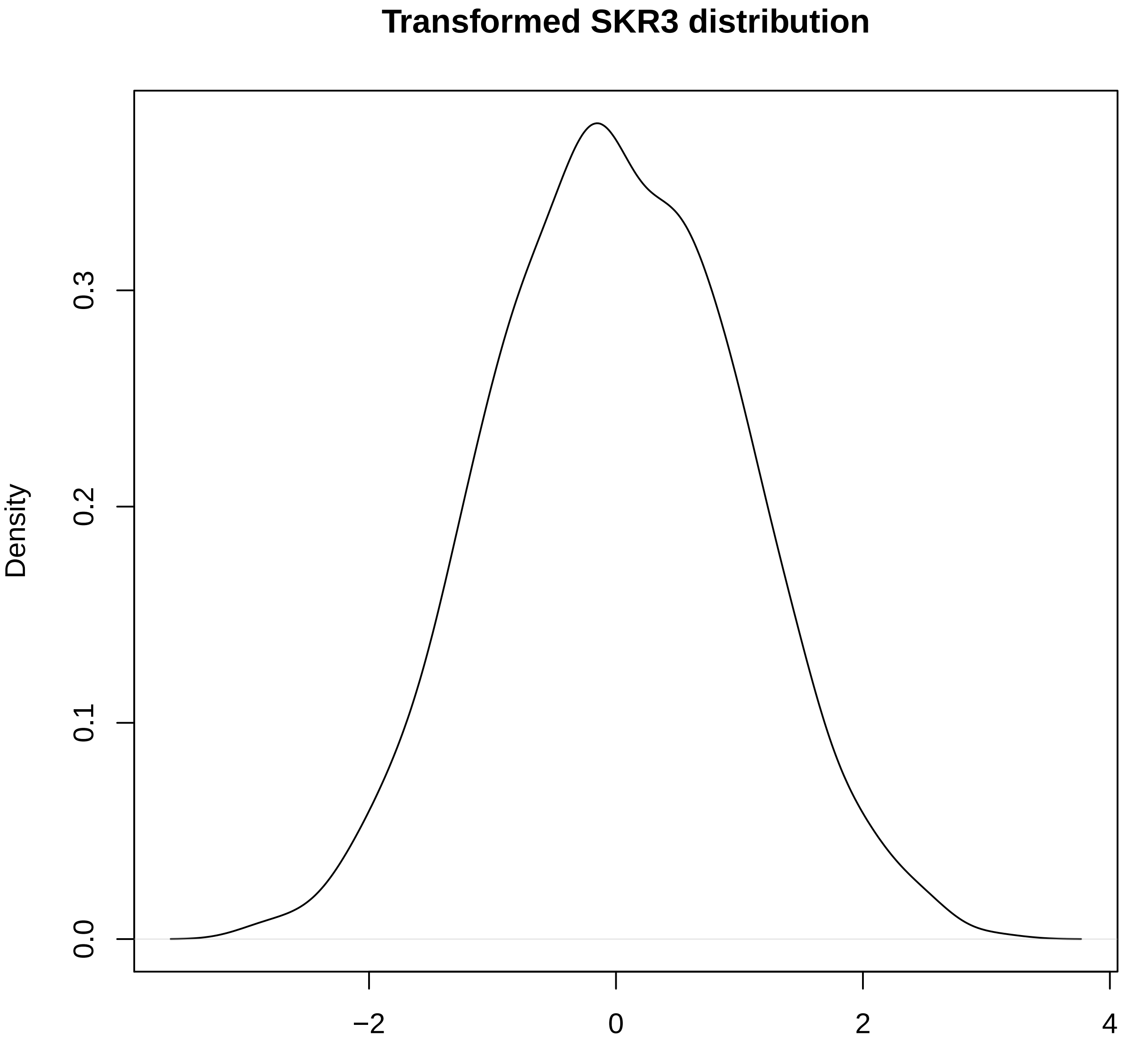
Transformed SKR3 distribution
Density
0.0
0.1
0.2
0.3
−2
0
2
4

Transformed FLRT2 distribution

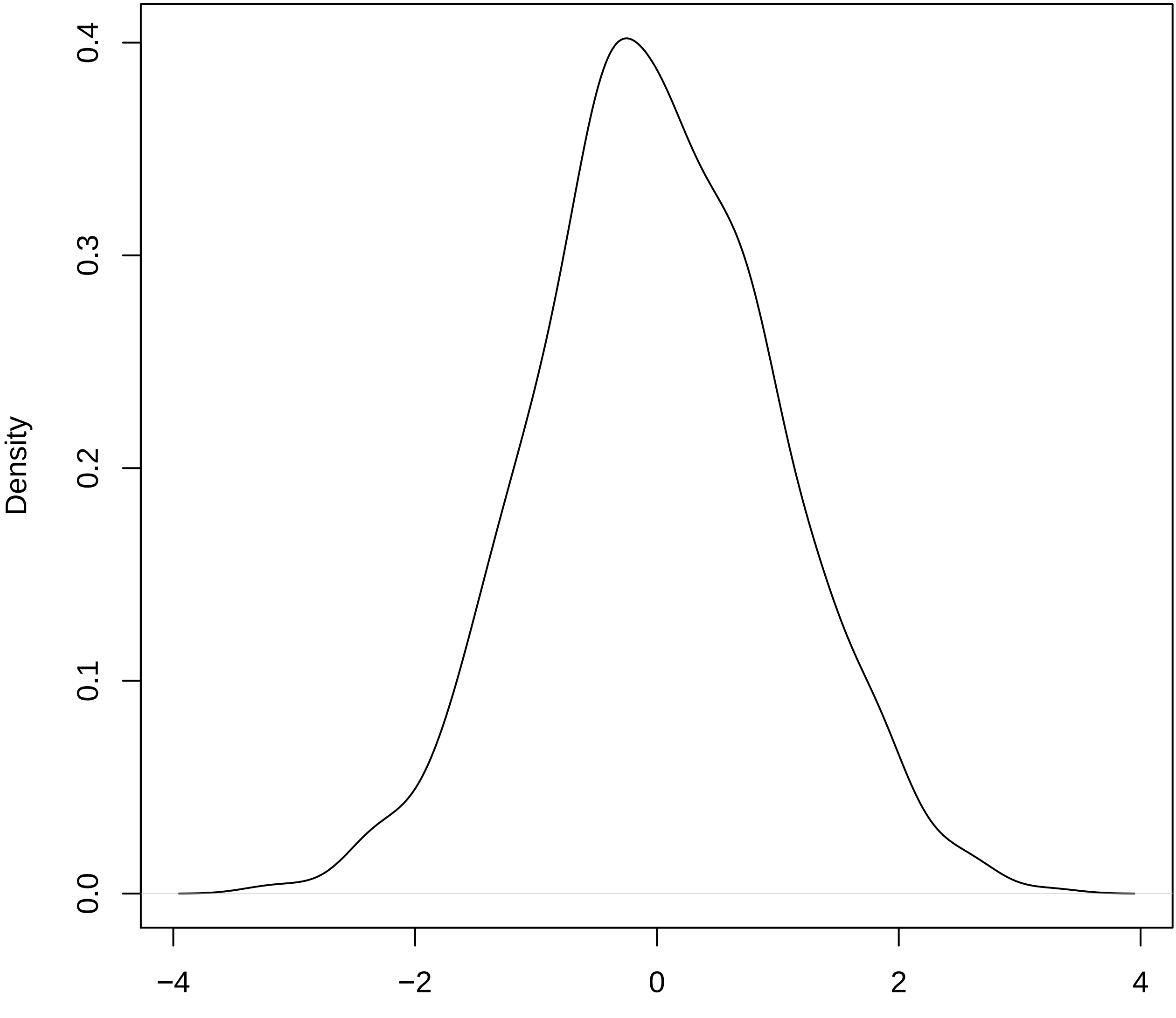

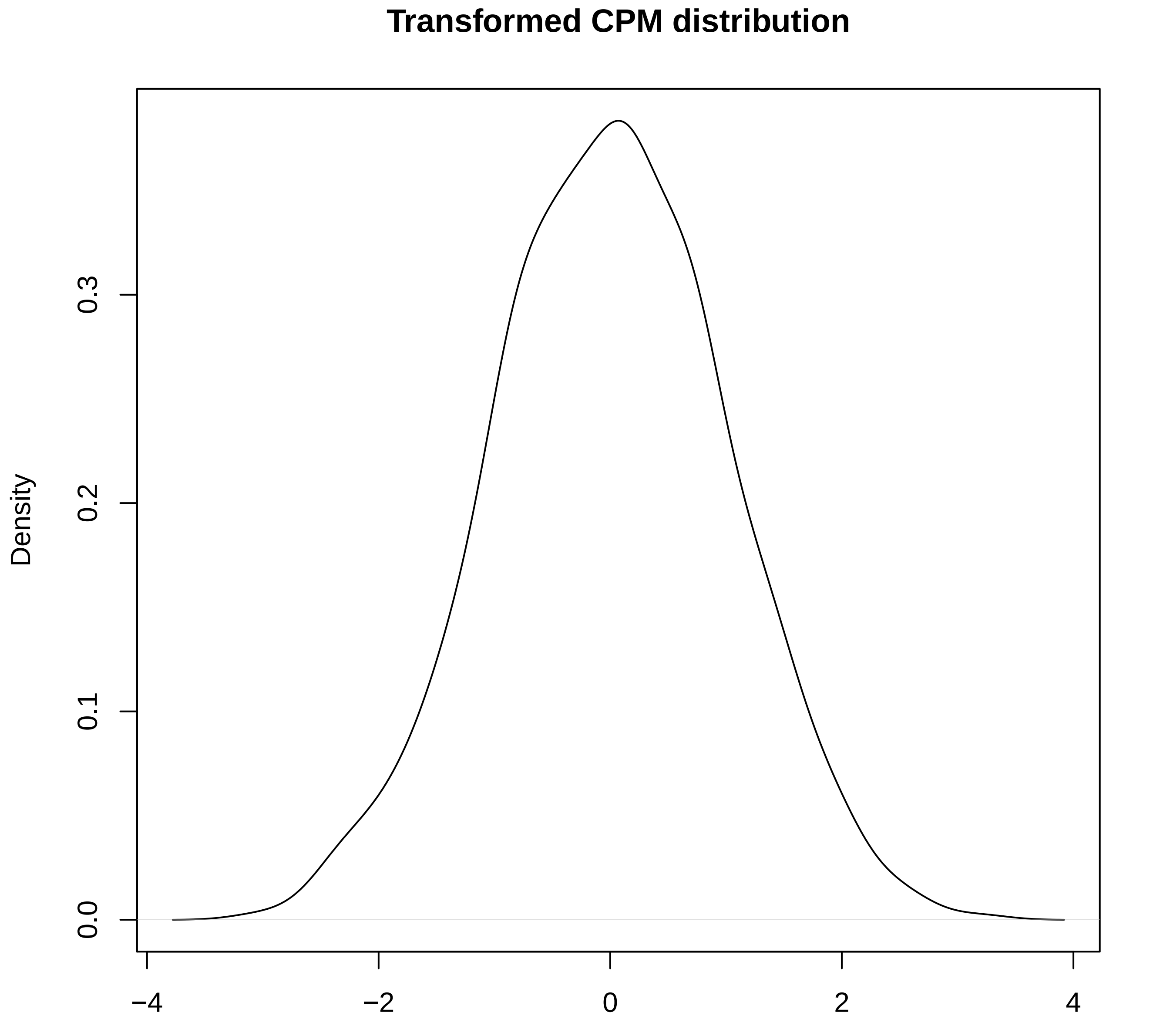
Transformed CPM distribution
Density
0.0
0.1
0.2
0.3
−4
−2
0
2
4

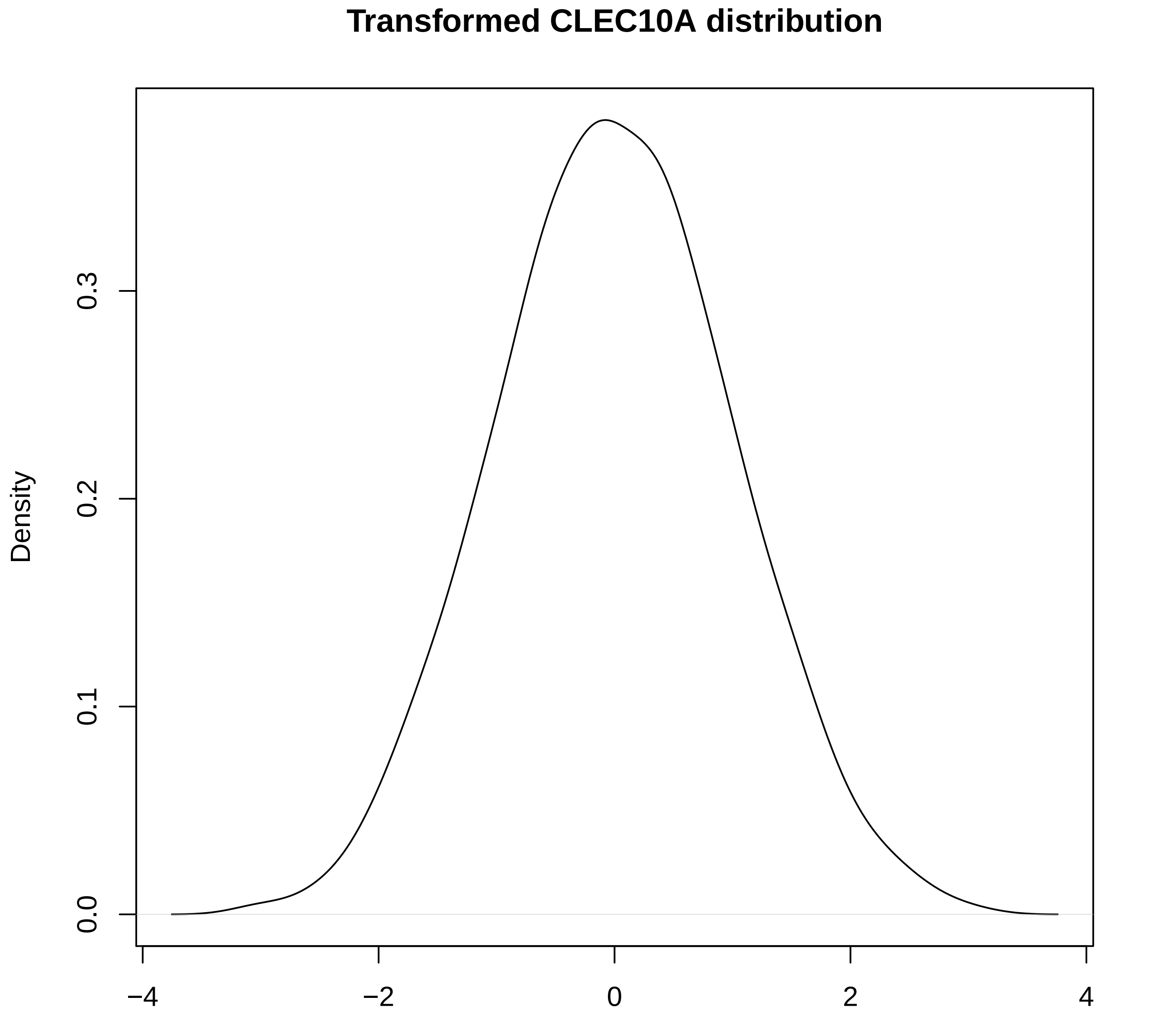

Transformed CLEC10A distribution
Density
0.0
0.1
0.2
0.3
−4
−2
0
2
4

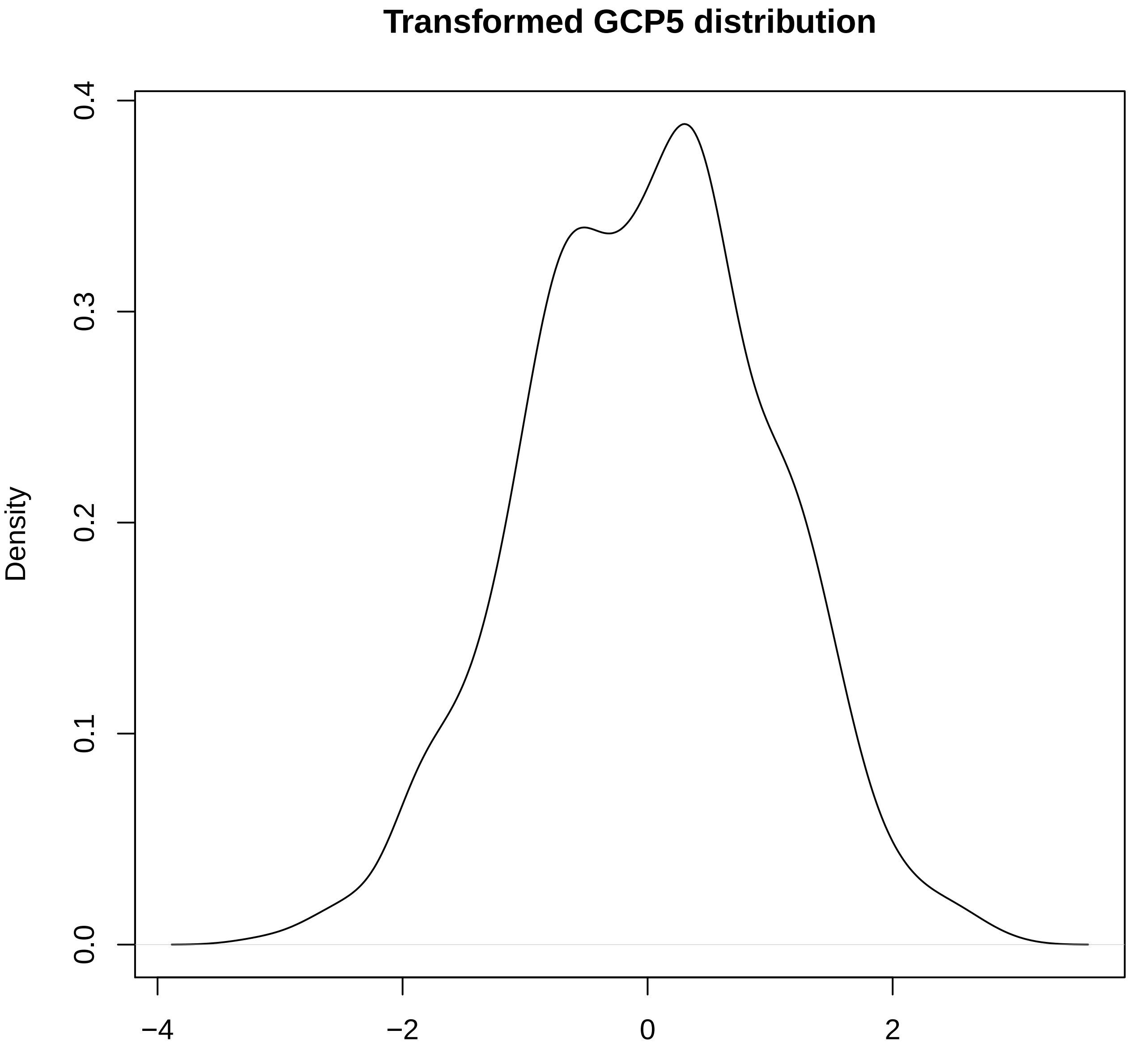

Transformed GCP5 distribution
Density
0.0
0.1
0.2
0.3
0.4
−4
−2
0
2

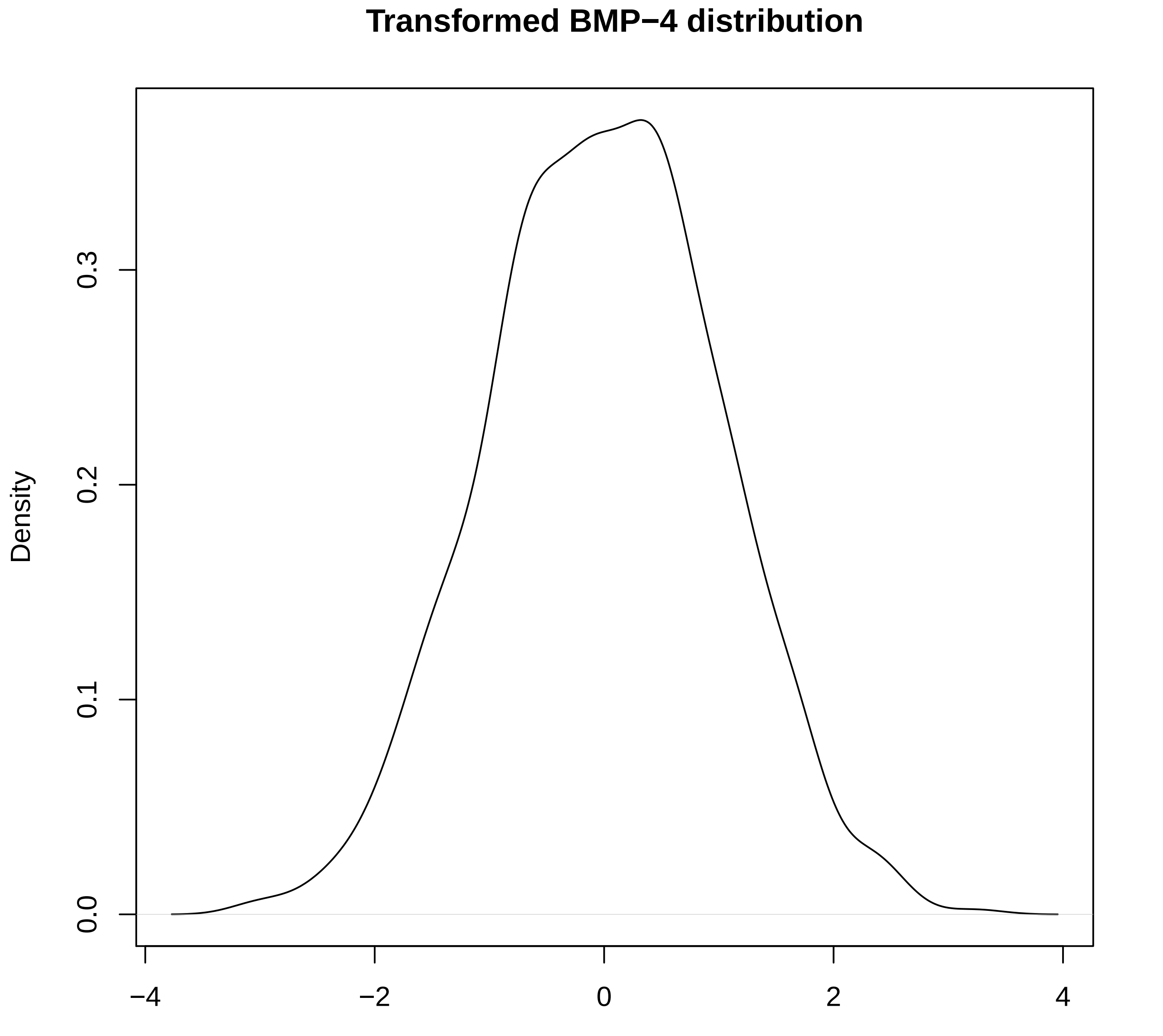
Transformed BMP−4 distribution
Density
0.0
0.1
0.2
0.3
−4
−2
0
2
4

Transformed FcRL2 distribution

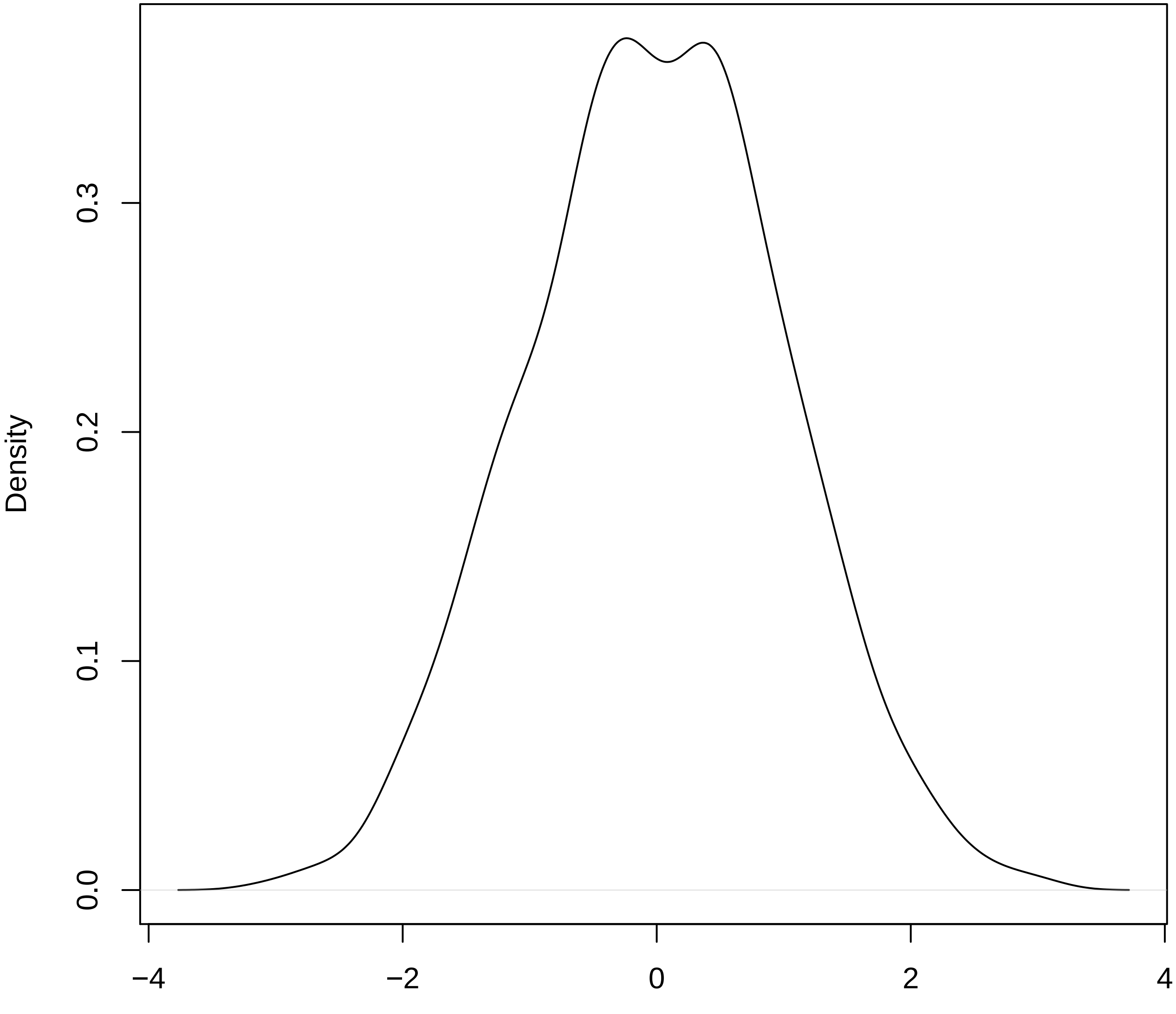

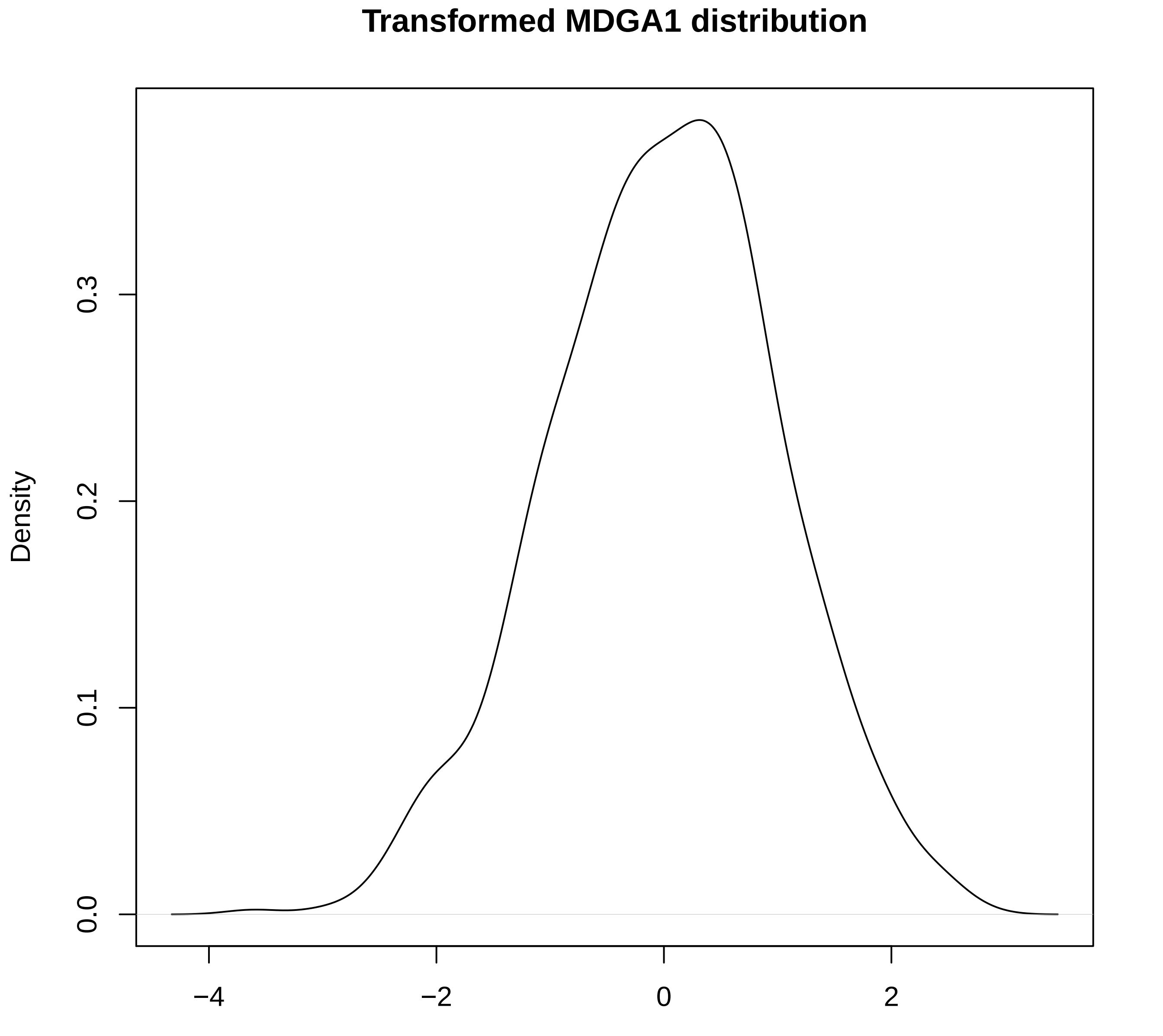

Transformed MDGA1 distribution
Density
0.0
0.1
0.2
0.3
−4
−2
0
2

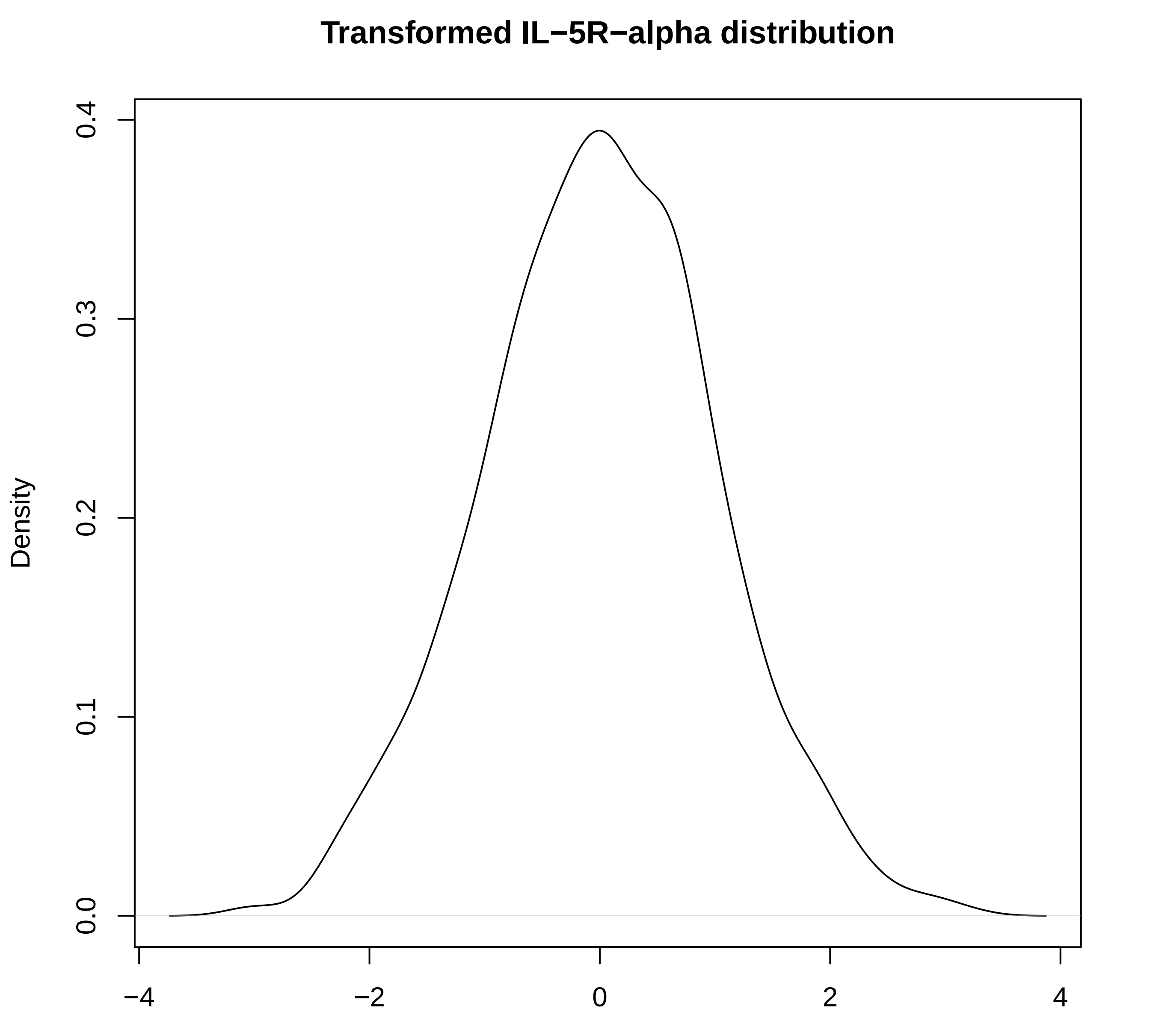

Transformed IL−5R−alpha distribution
Density
0.0
0.1
0.2
0.3
0.4
−4
−2
0
2
4

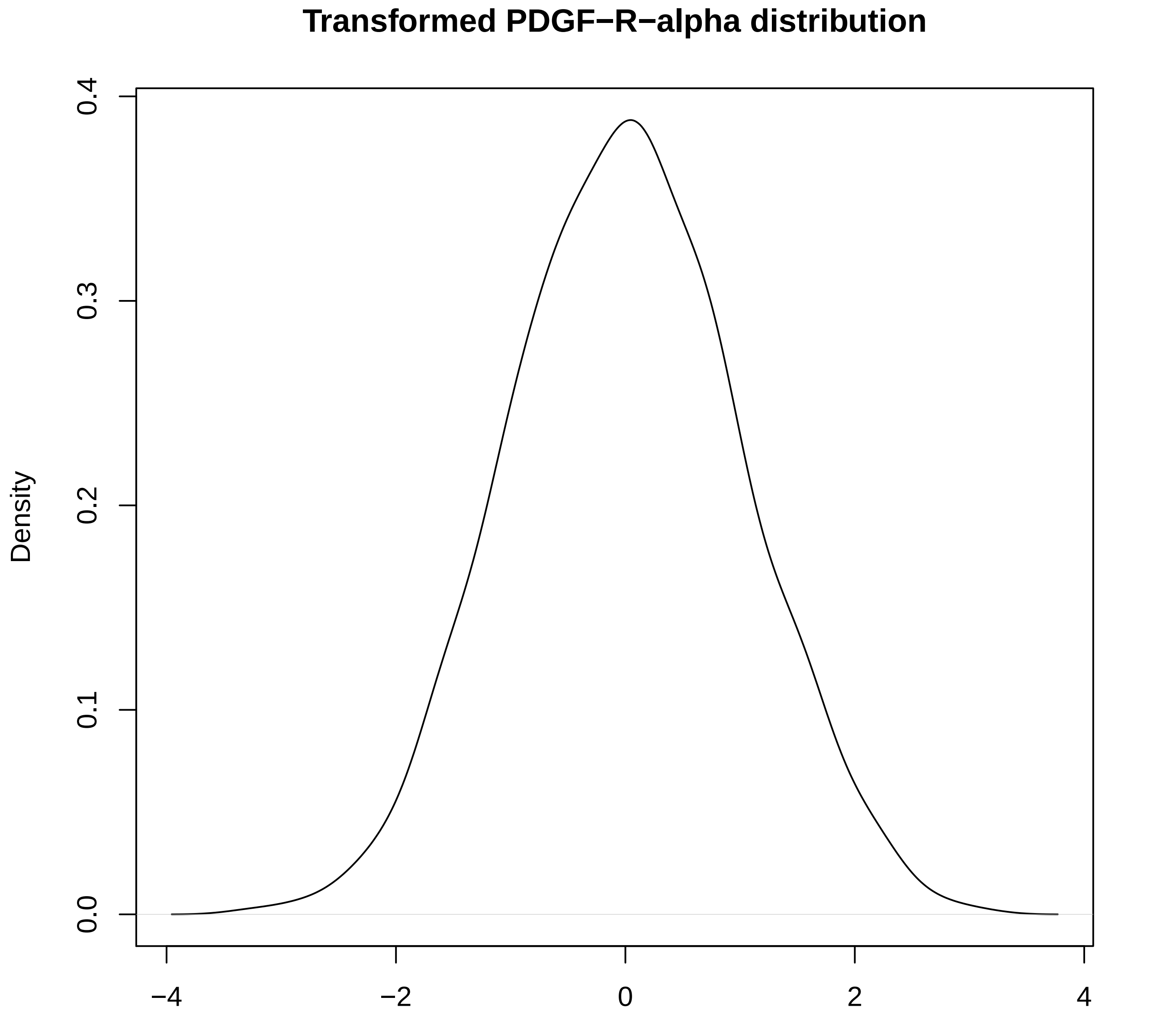
Transformed PDGF−R−alpha distribution
Density
0.0
0.1
0.2
0.3
0.4
−4
−2
0
2
4

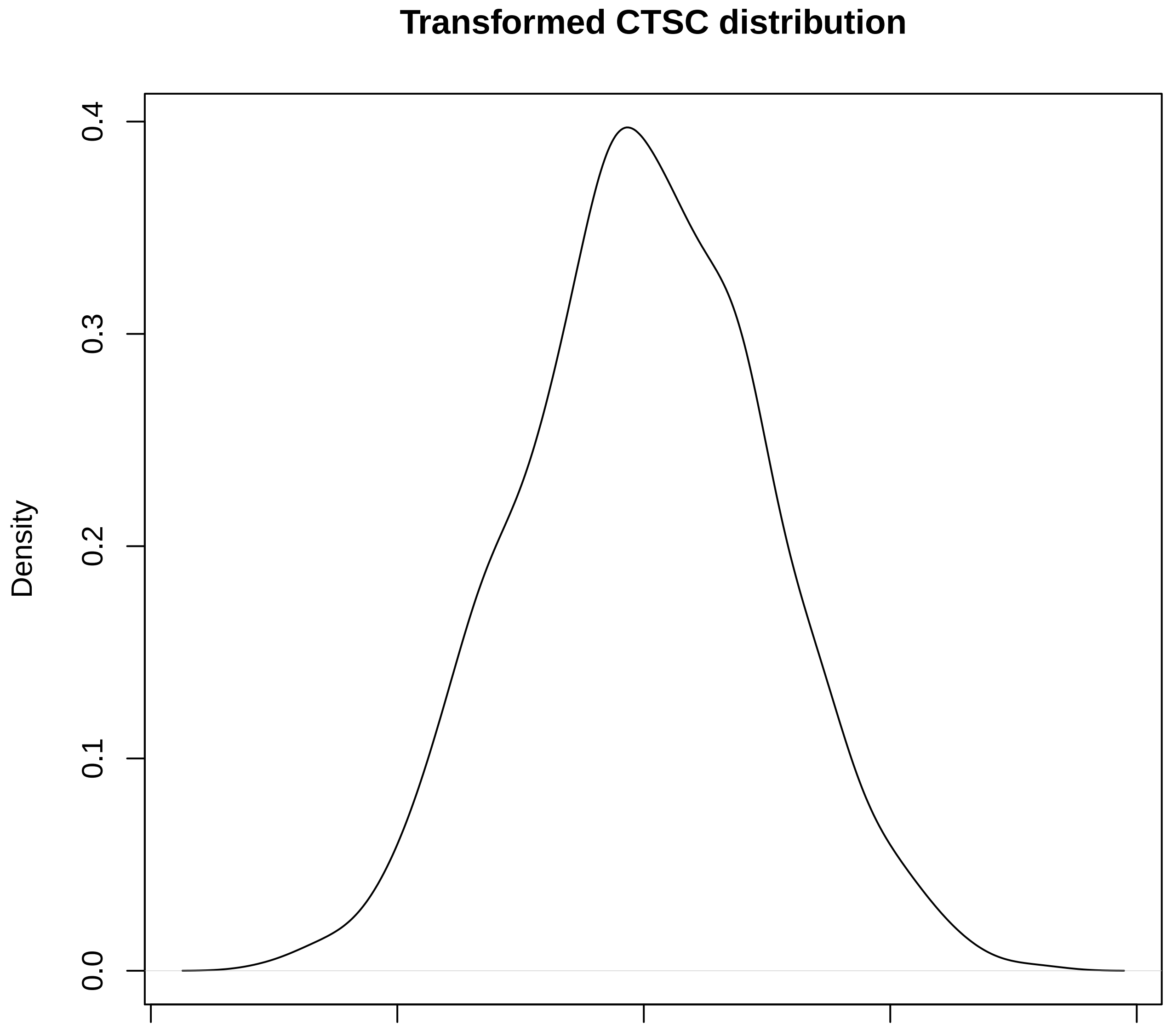
Transformed CTSC distribution
Density
0.0
0.1
0.2
0.3
0.4
−4
−2
0
2
4

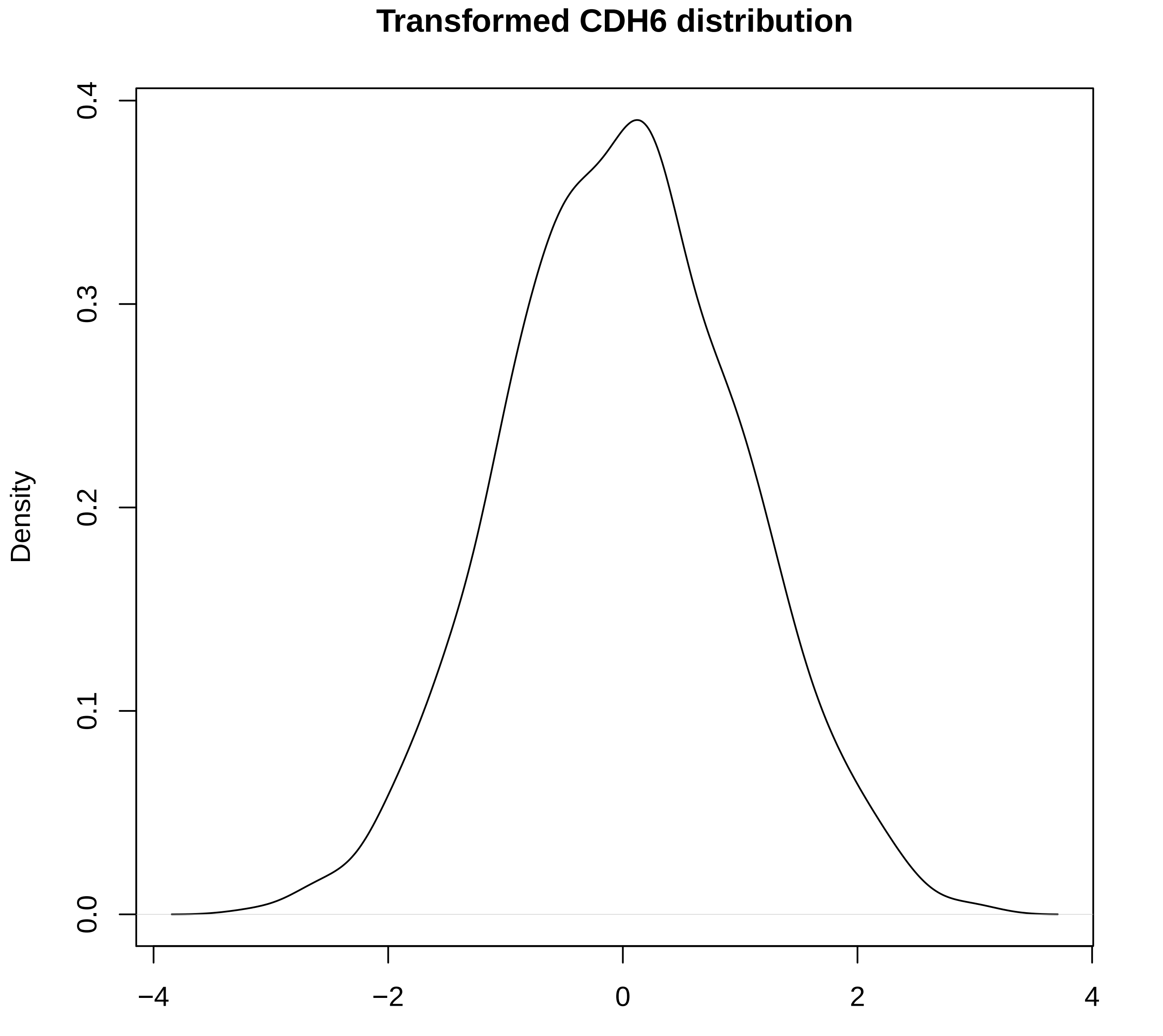

Transformed CDH6 distribution
Density
0.0
0.1
0.2
0.3
0.4
−4
−2
0
2
4

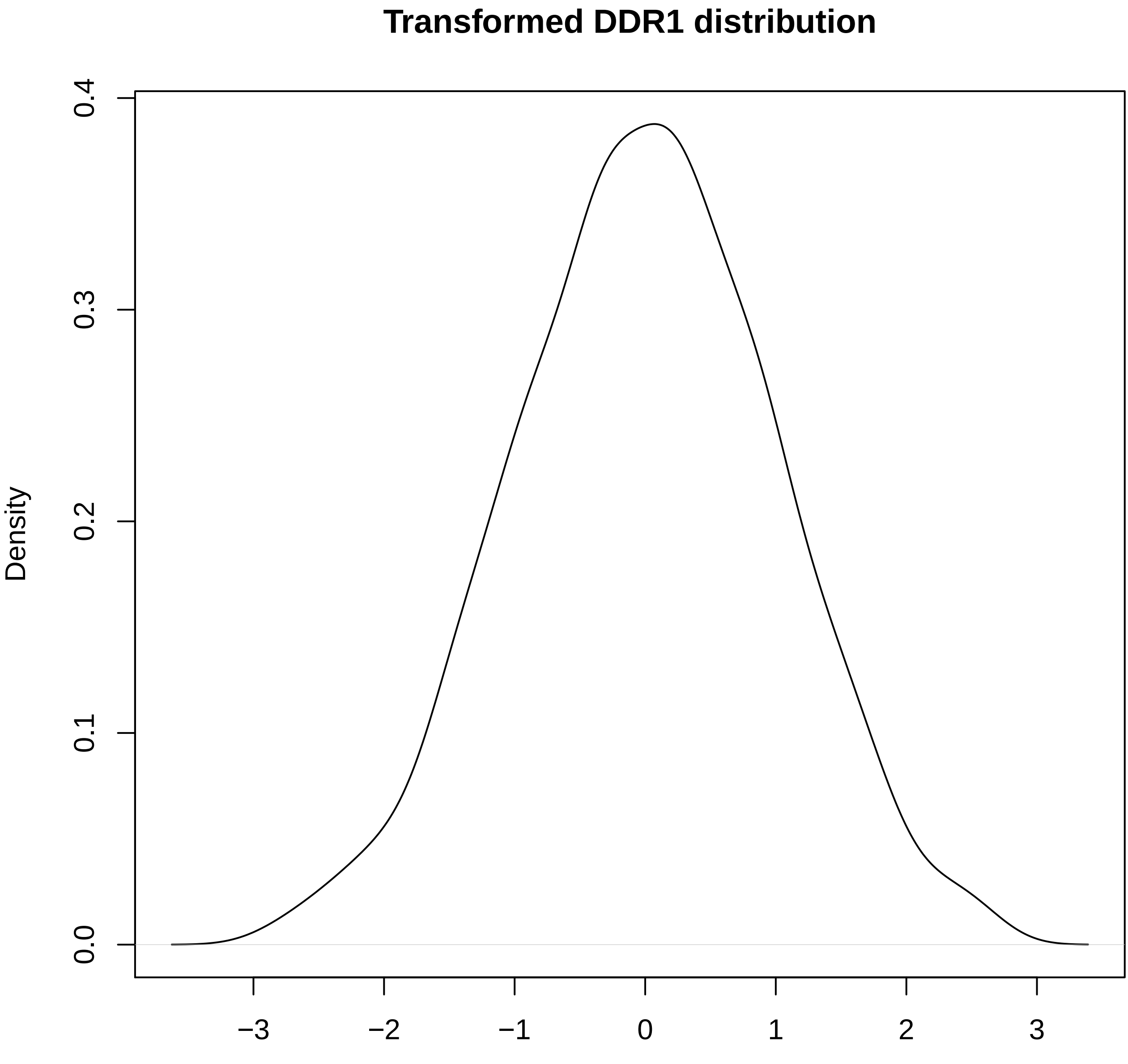

Transformed DDR1 distribution
Density
0.0
0.1
0.2
0.3
0.4
−3
−2
−1
0
1
2
3

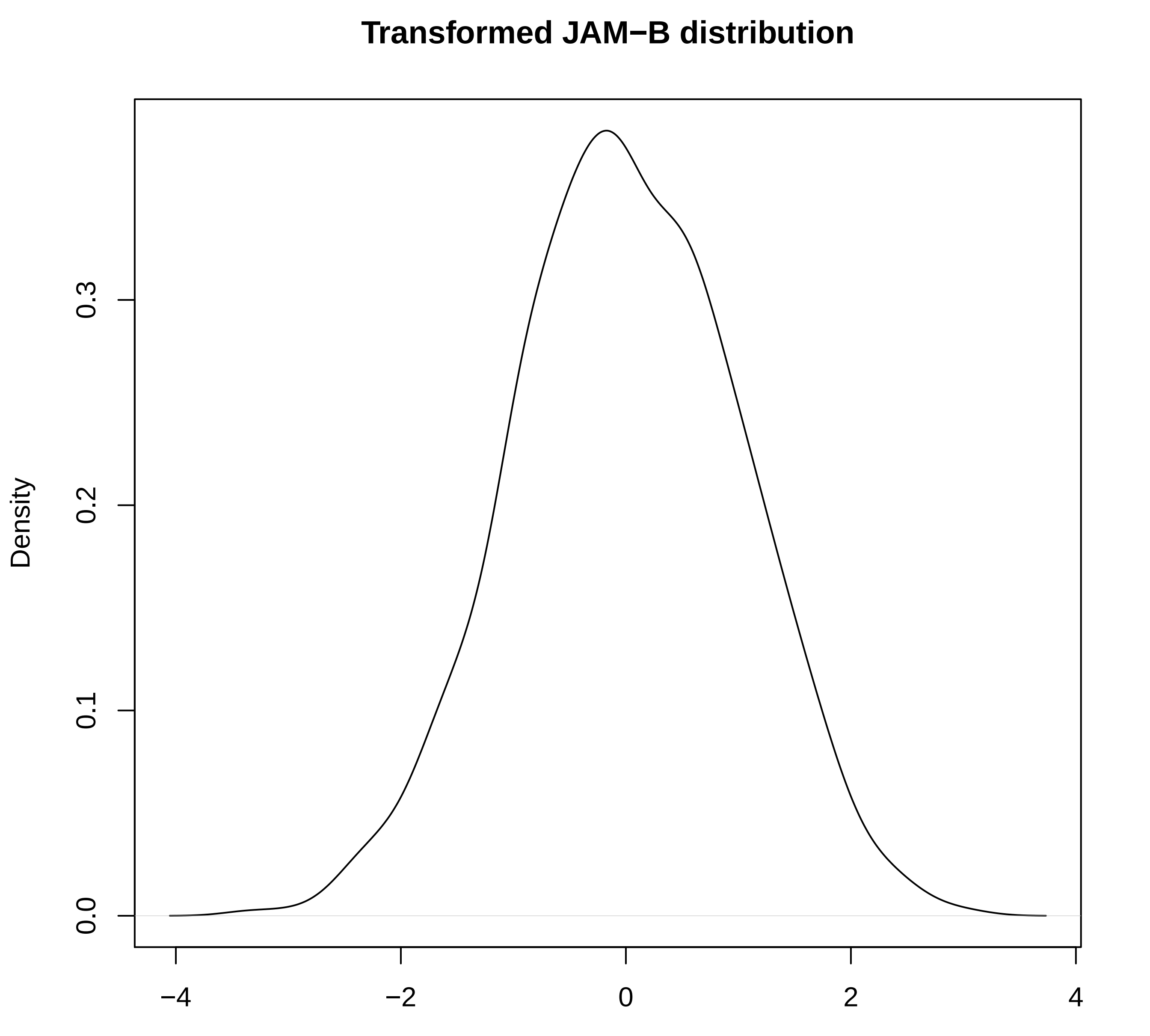
Transformed JAM−B distribution
Density
0.0
0.1
0.2
0.3
−4
−2
0
2
4

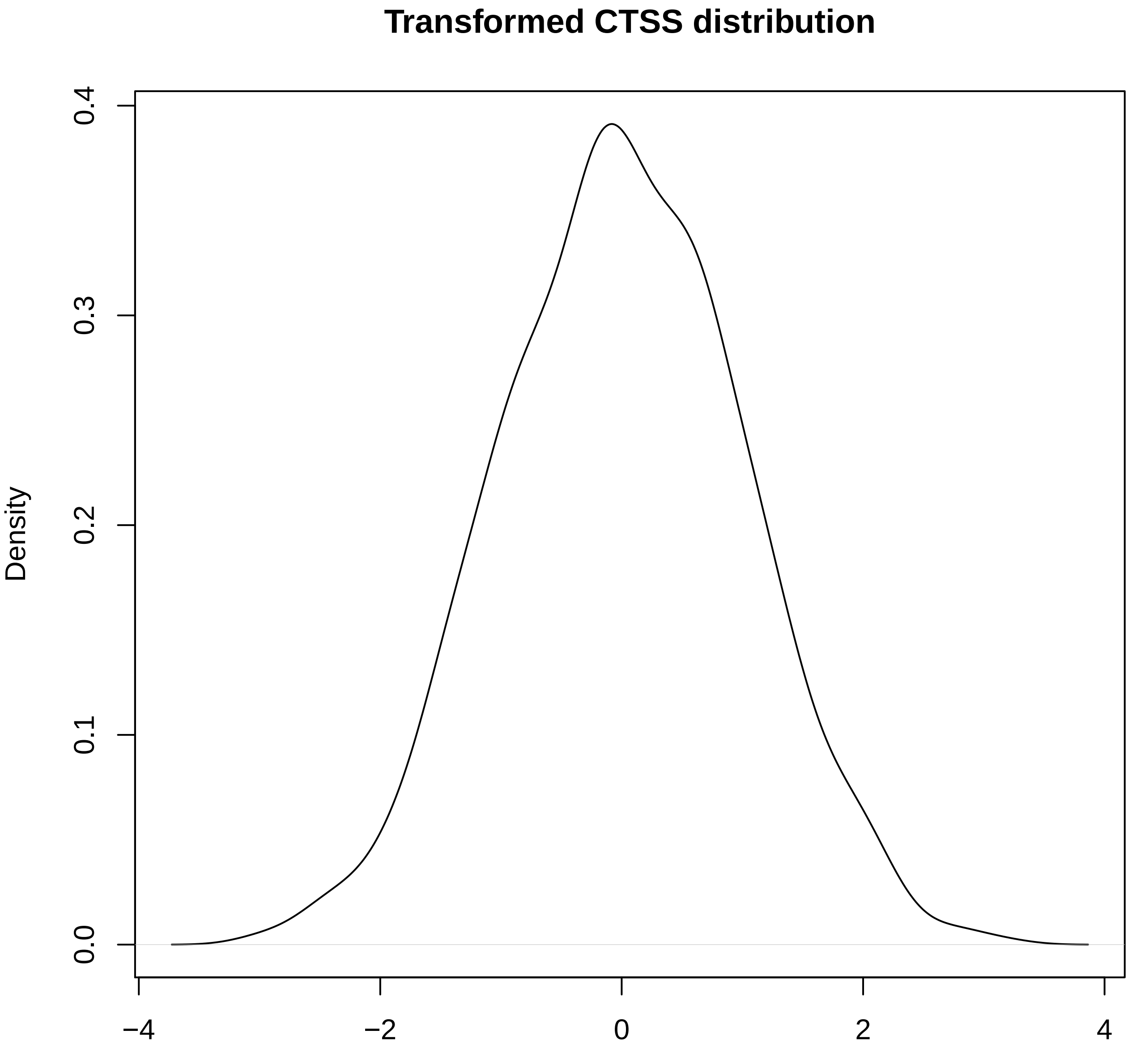
Transformed CTSS distribution
Density
0.0
0.1
0.2
0.3
0.4
−4
−2
0
2
4

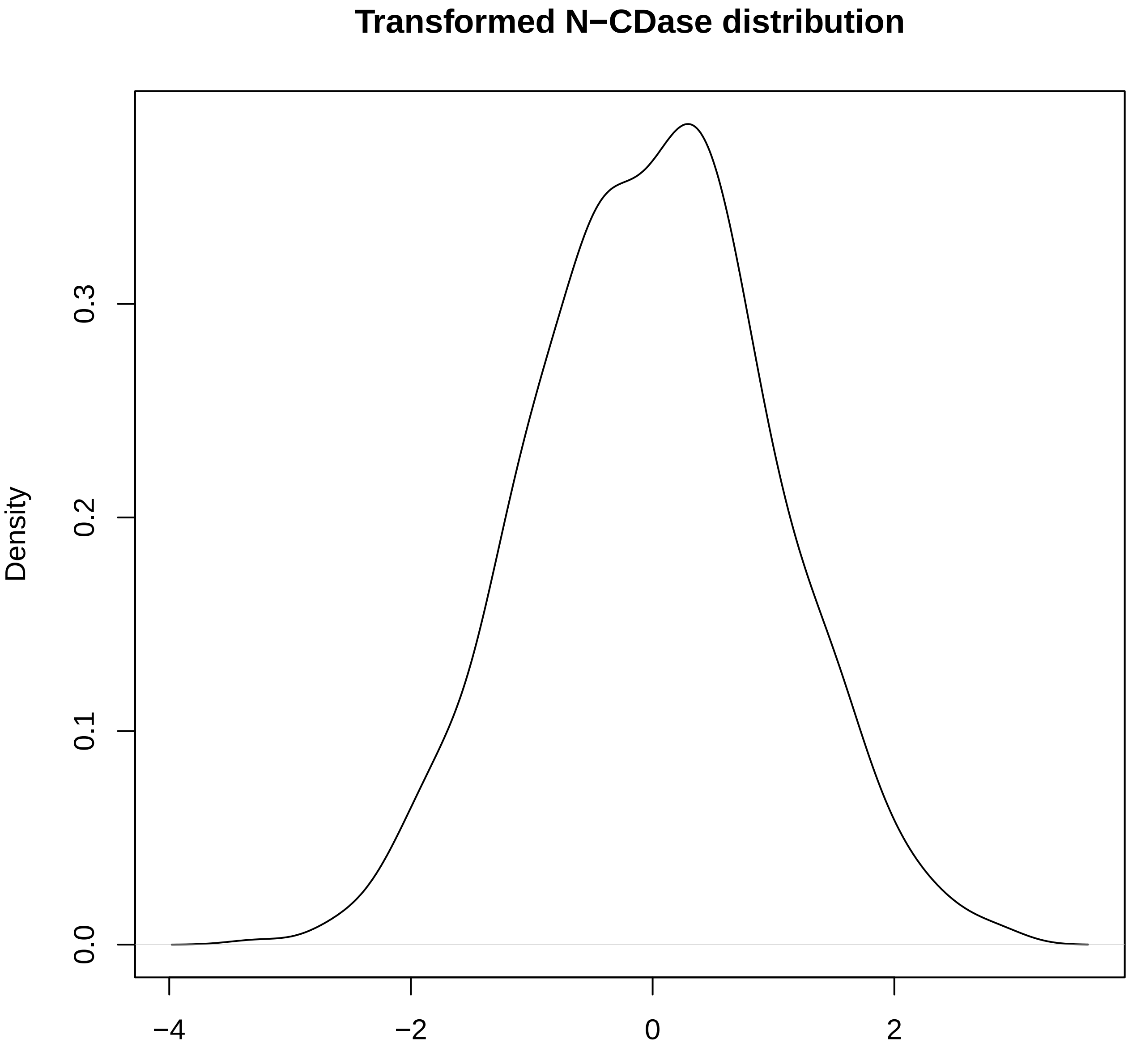
Transformed N−CDase distribution
Density
0.0
0.1
0.2
0.3
−4
−2
0
2

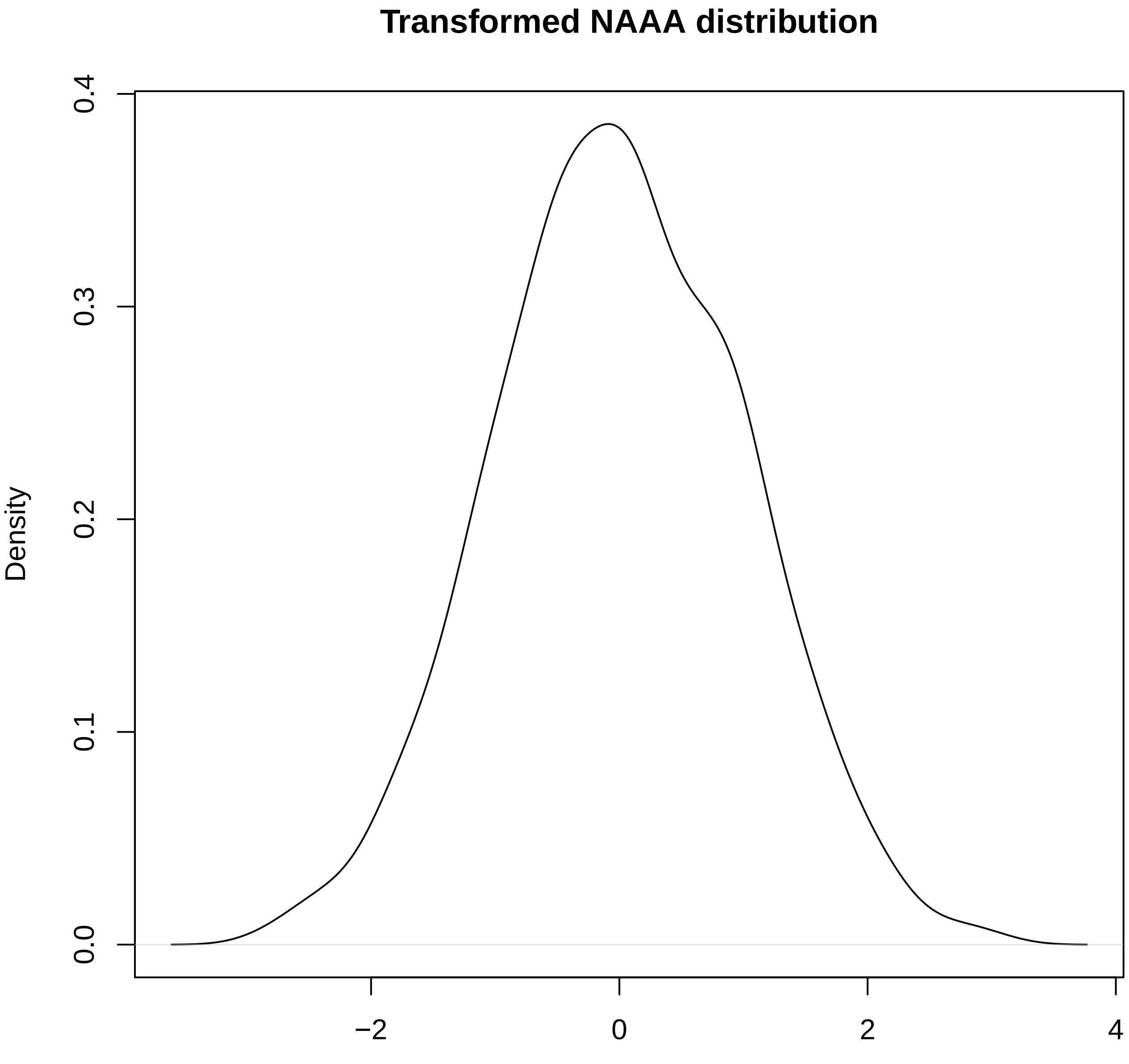

Transformed NAAA distribution
Density
0.0
0.1
0.2
0.3
0.4
−2
0
2
4

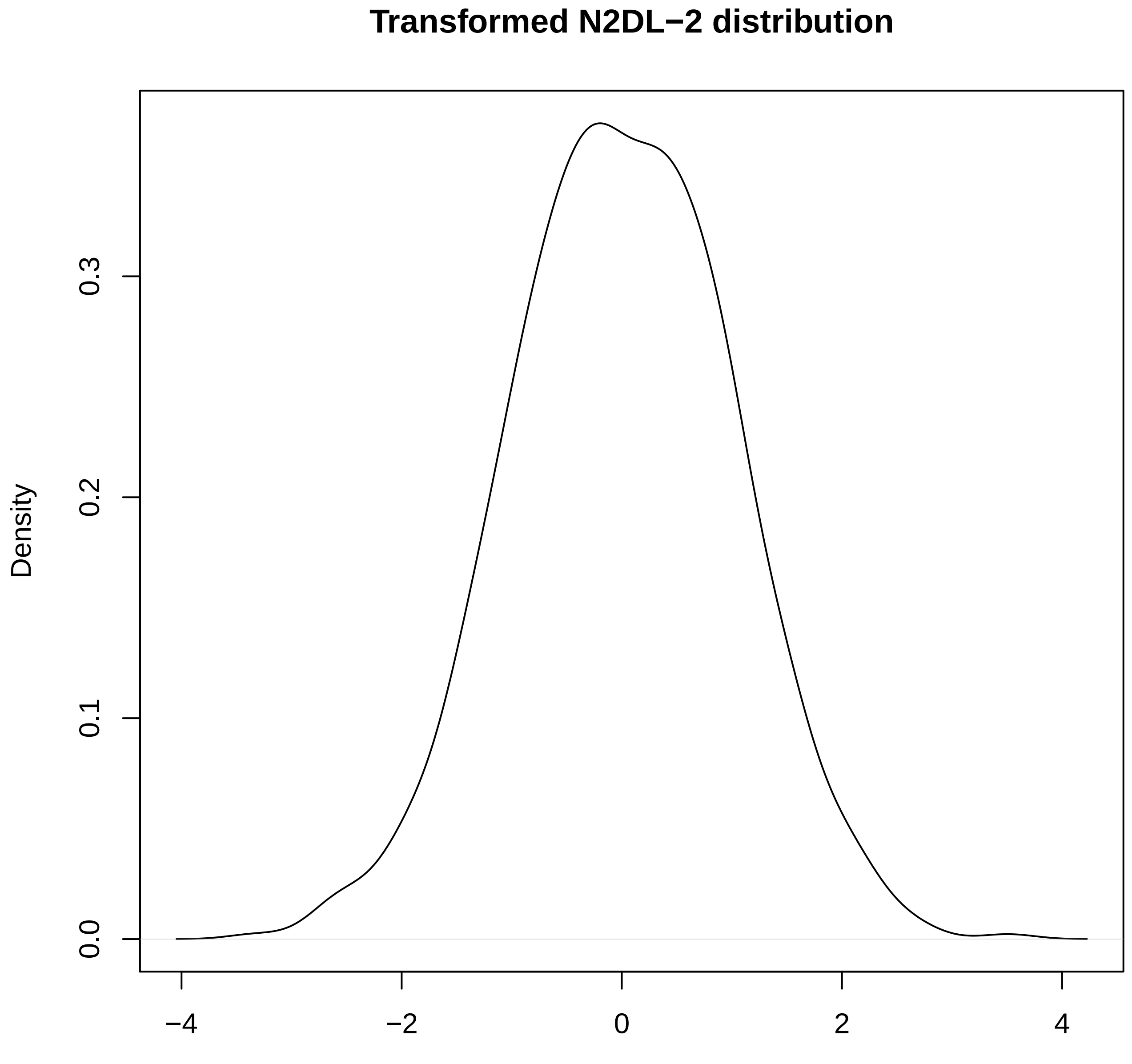

Transformed N2DL−2 distribution
Density
0.0
0.1
0.2
0.3
−4
−2
0
2
4

Transformed PLXNB1 distribution

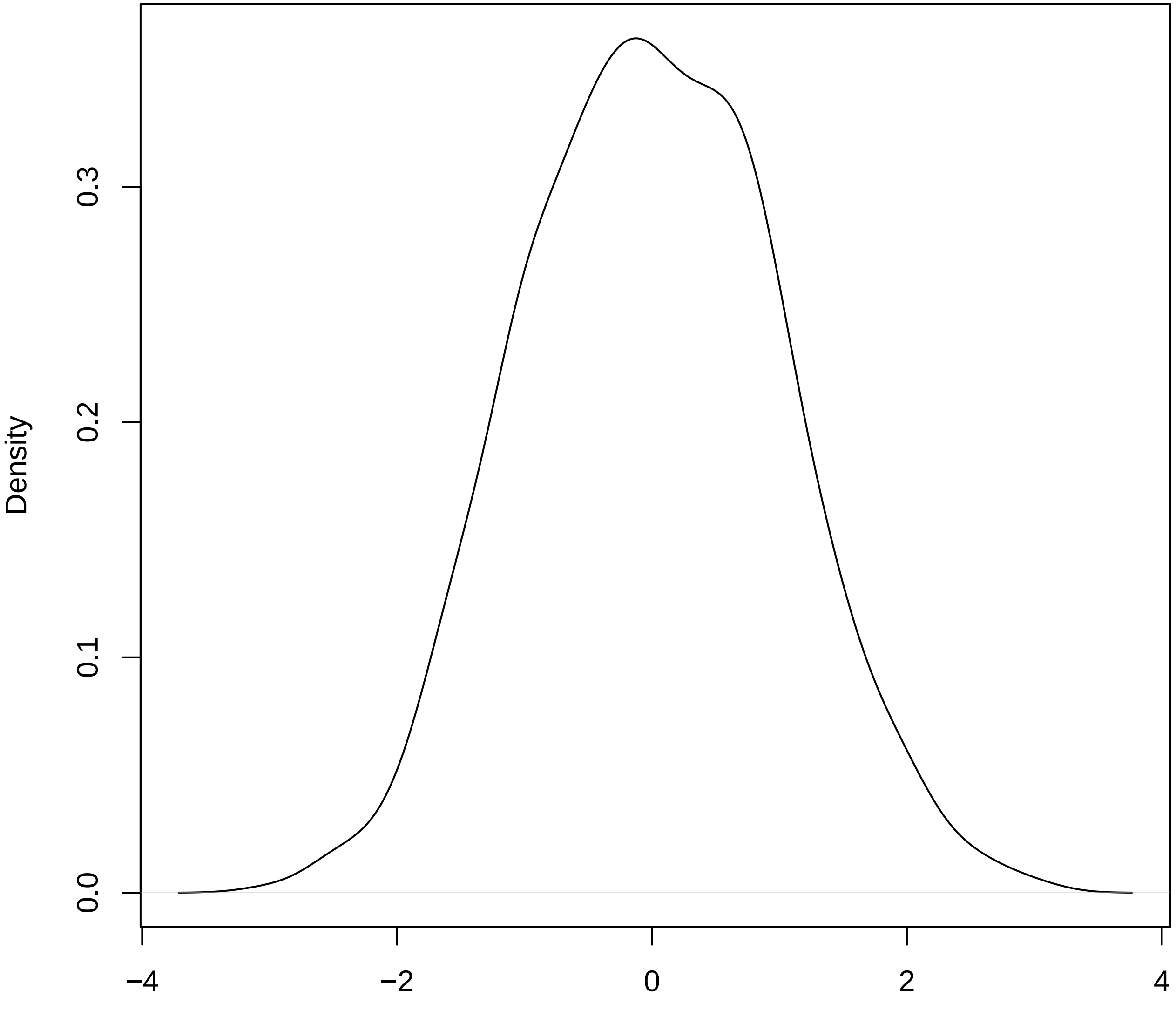

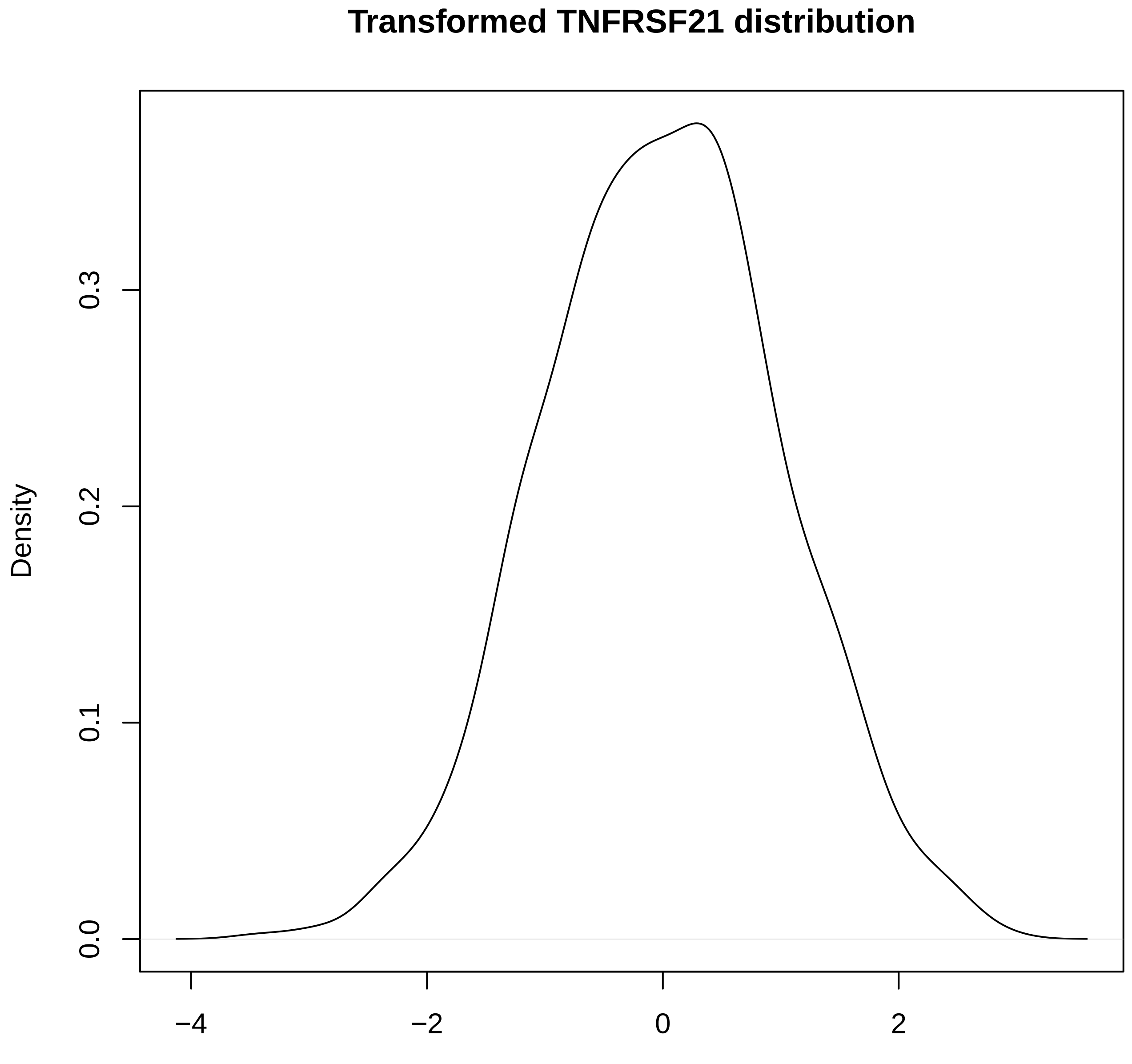

Transformed TNFRSF21 distribution
Density
0.0
0.1
0.2
0.3
−4
−2
0
2

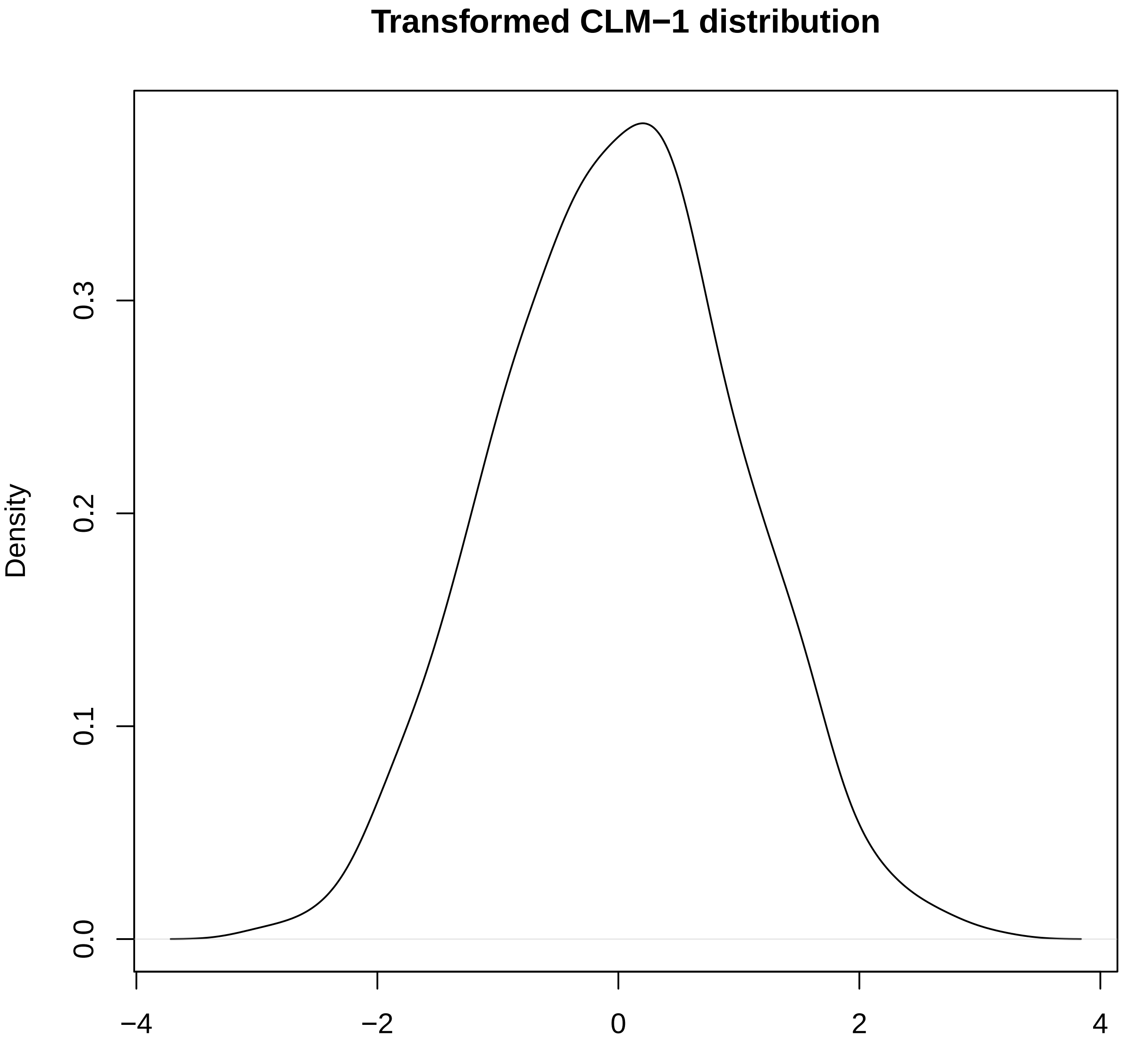
Transformed CLM−1 distribution
Density
0.0
0.1
0.2
0.3
−4
−2
0
2
4

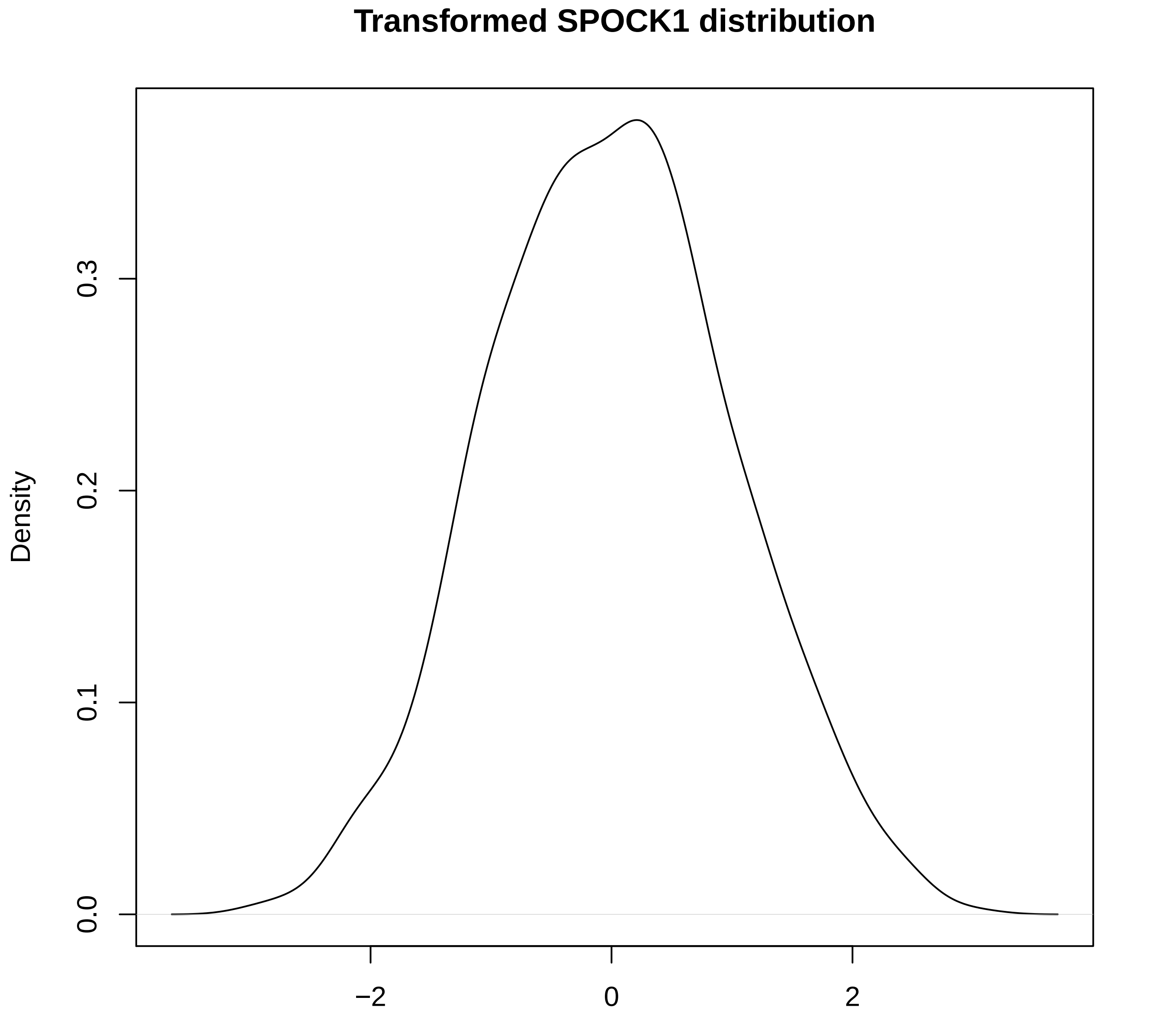
Transformed SPOCK1 distribution
Density
0.0
0.1
0.2
0.3
−2
0
2

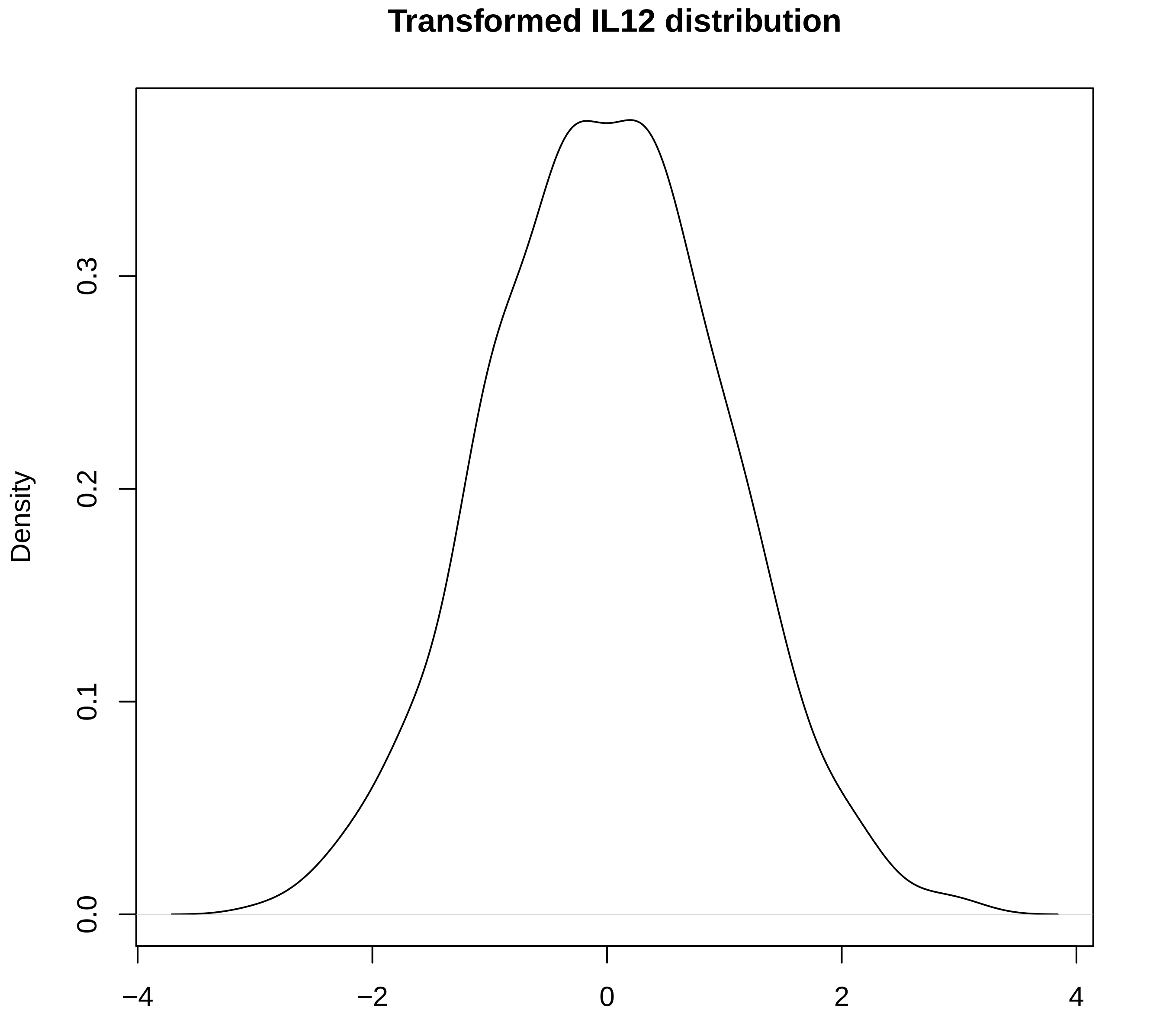

Transformed IL12 distribution
Density
0.0
0.1
0.2
0.3
−4
−2
0
2
4

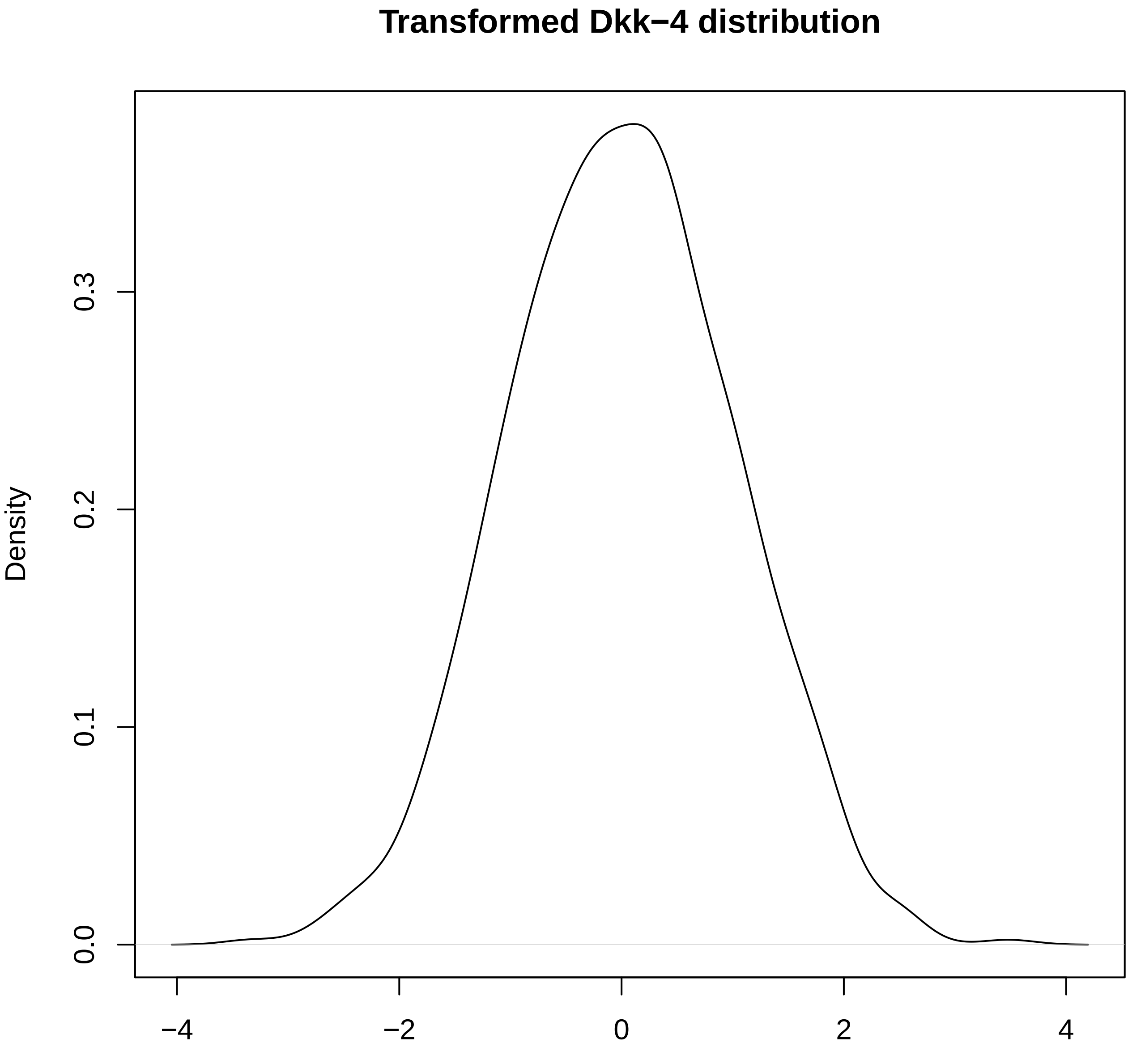

Transformed Dkk−4 distribution
Density
0.0
0.1
0.2
0.3
−4
−2
0
2
4

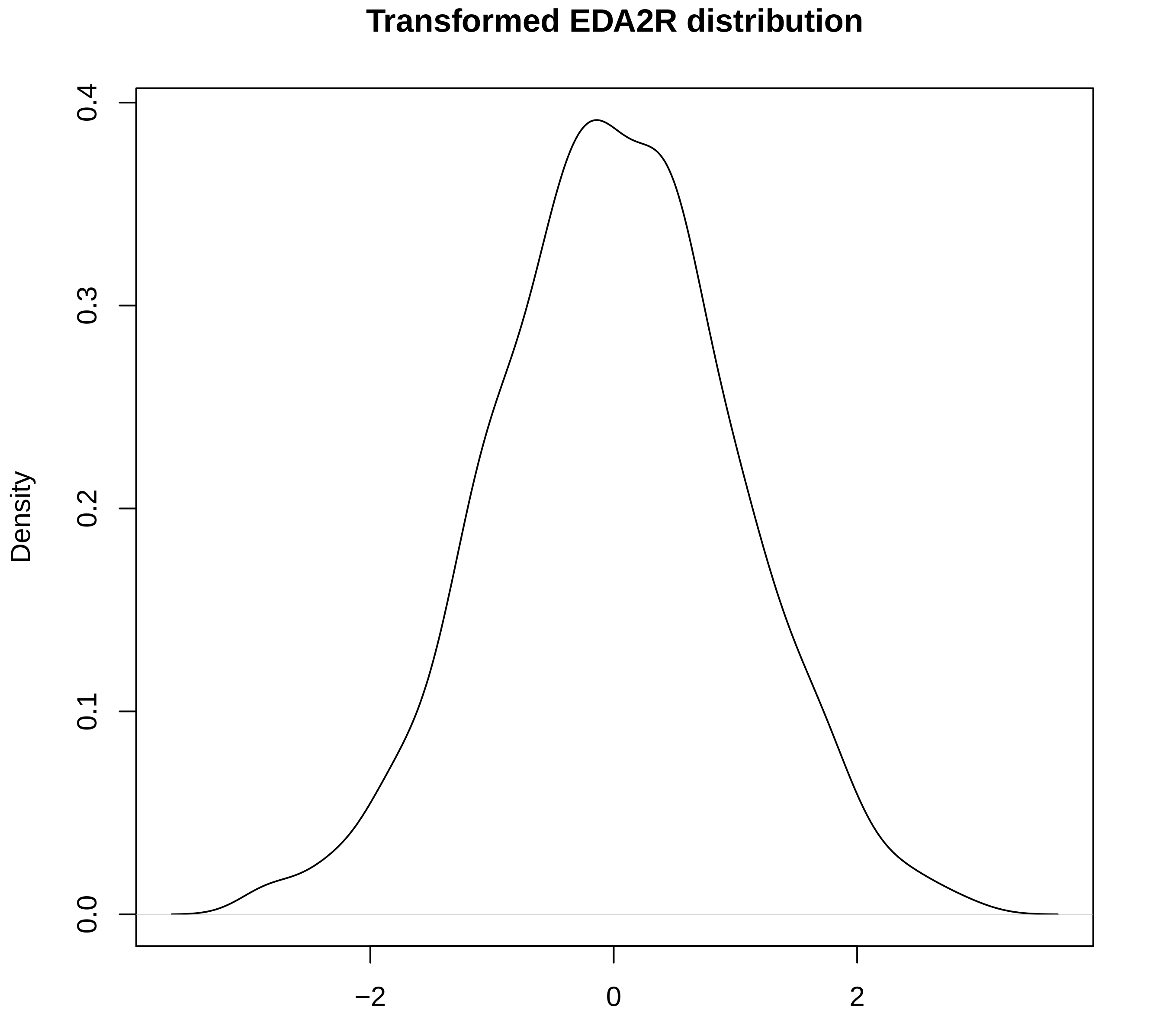
Transformed EDA2R distribution
Density
0.0
0.1
0.2
0.3
0.4
−2
0
2

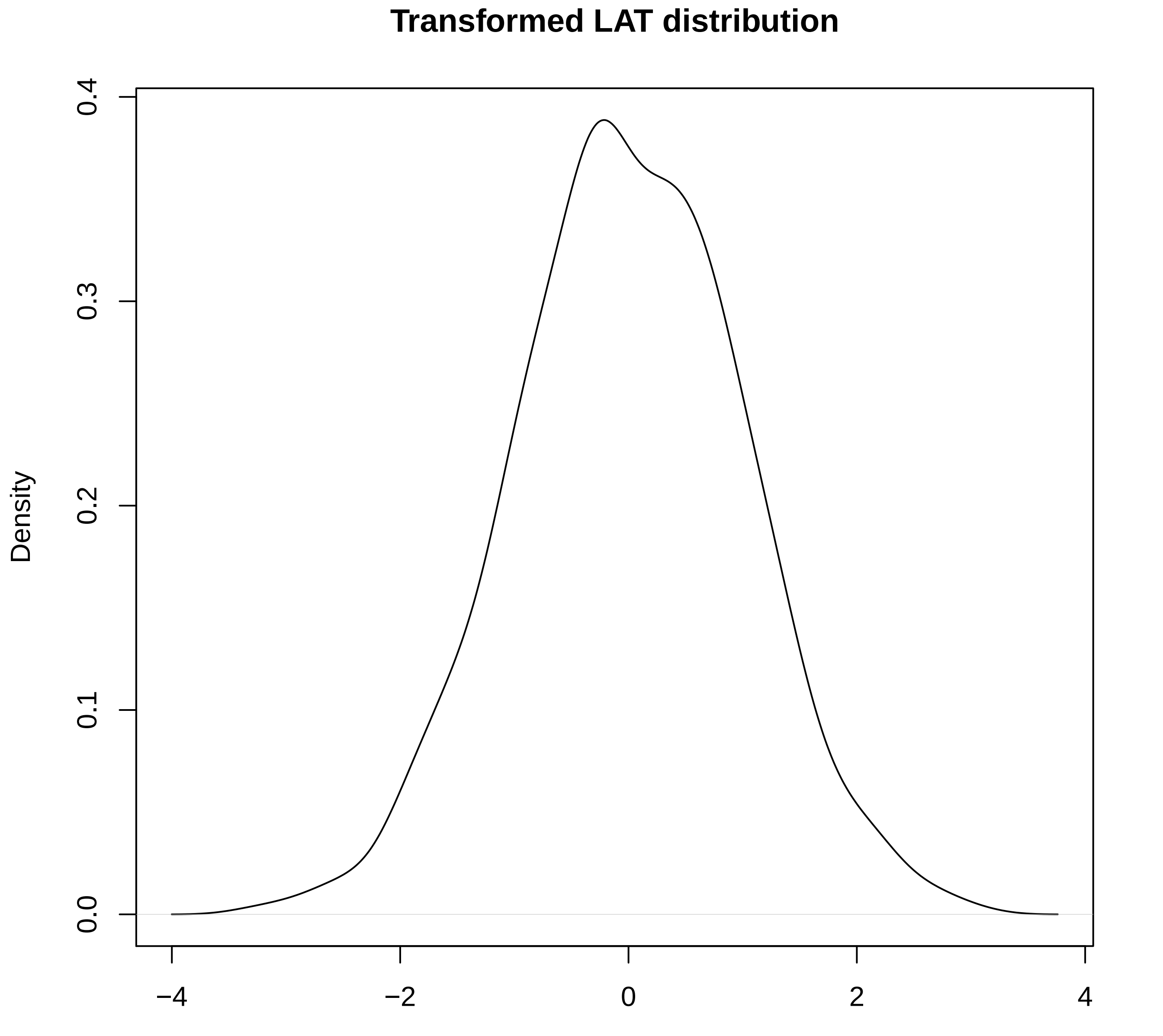

Transformed LAT distribution
Density
0.0
0.1
0.2
0.3
0.4
−4
−2
0
2
4

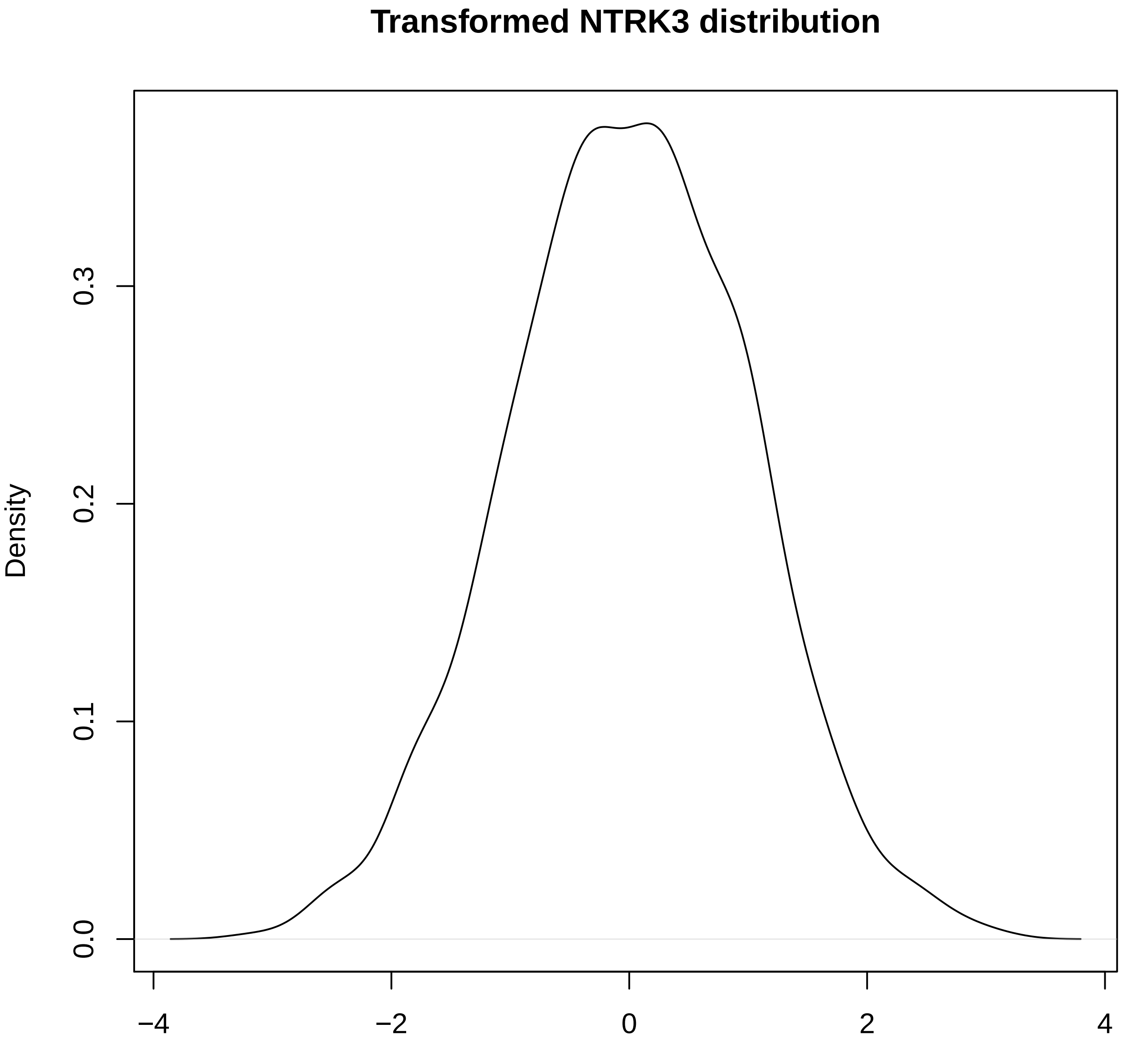
Transformed NTRK3 distribution
Density
0.0
0.1
0.2
0.3
−4
−2
0
2
4

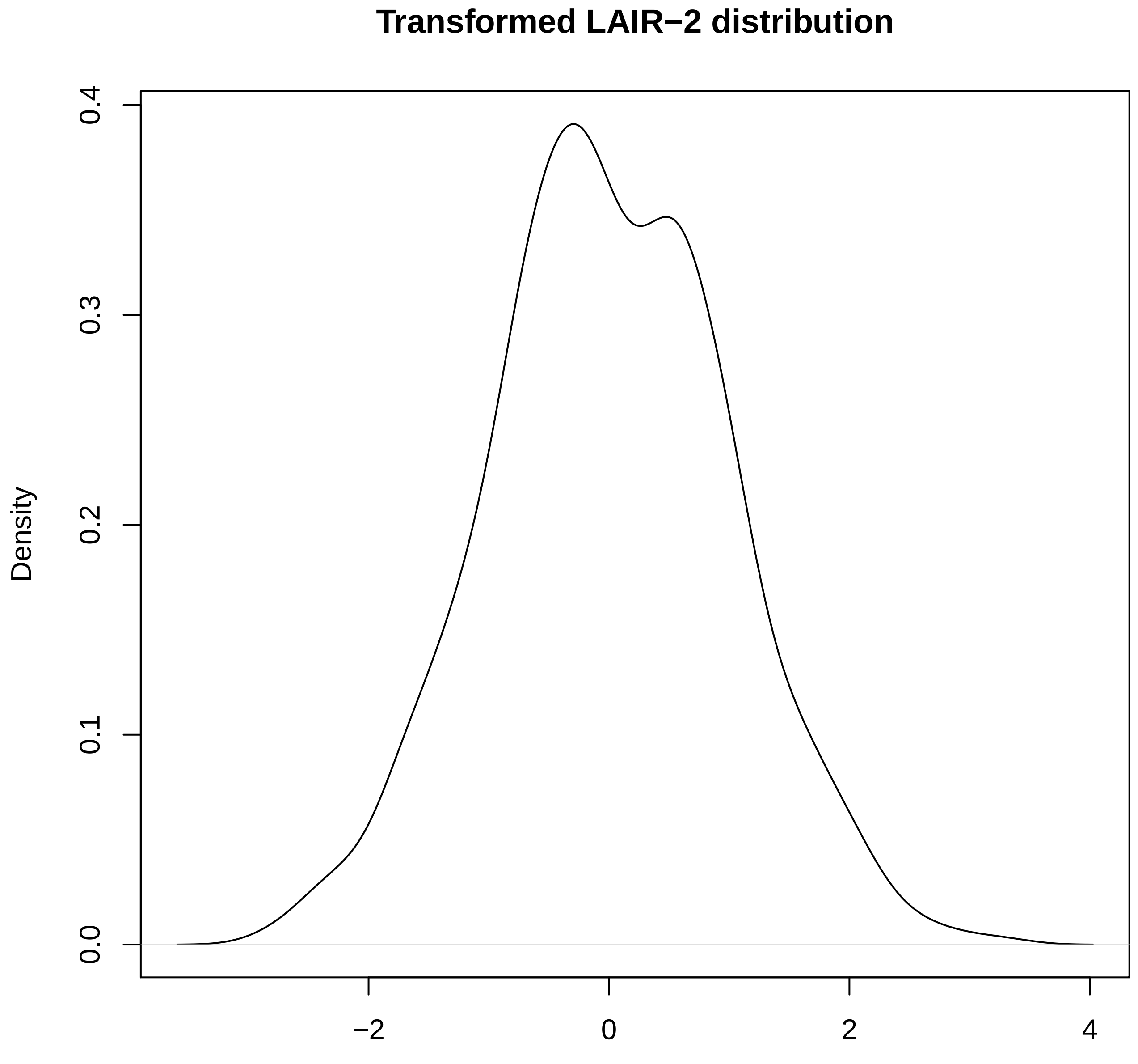

Transformed LAIR−2 distribution
Density
0.0
0.1
0.2
0.3
0.4
−2
0
2
4

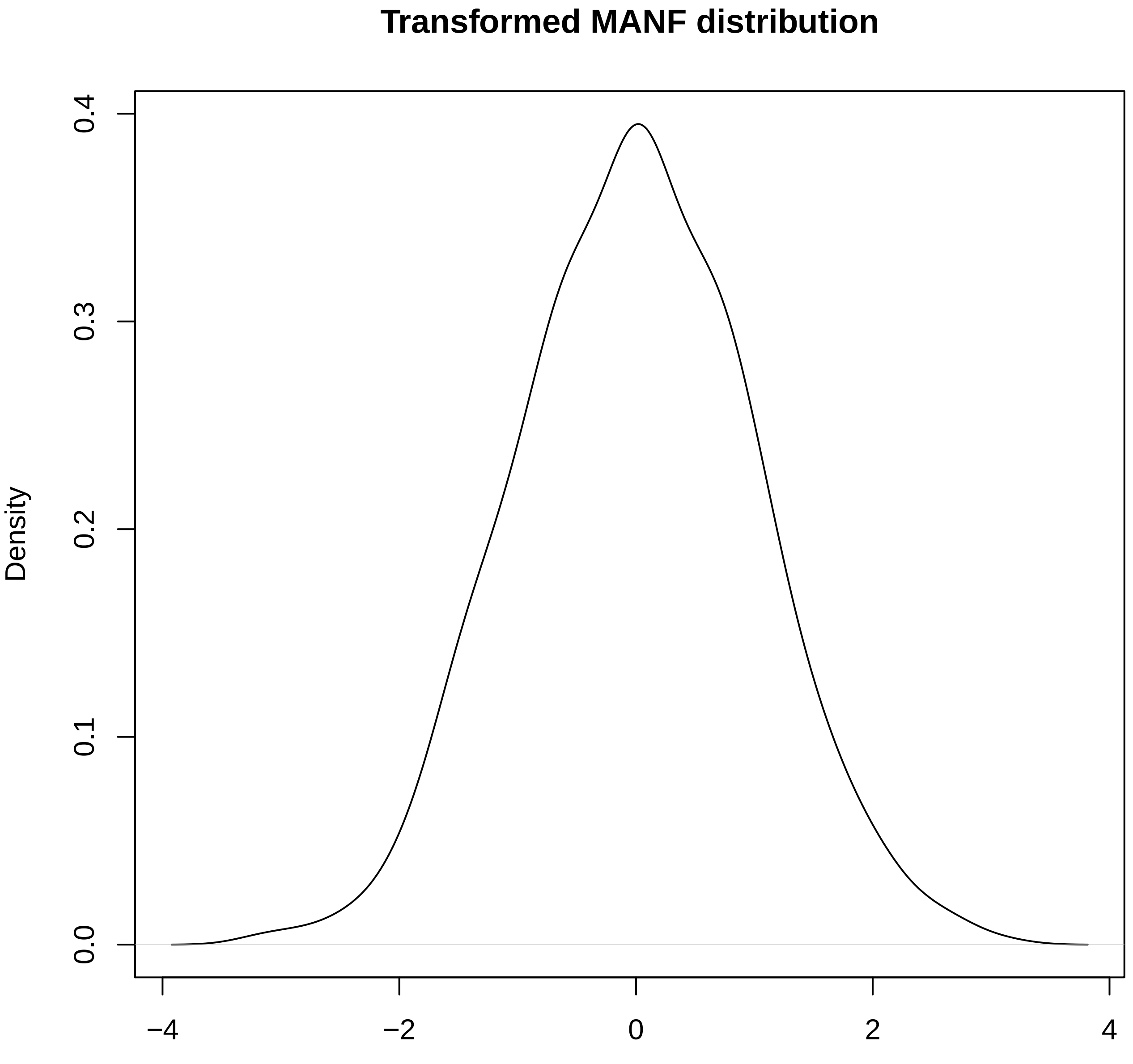
Transformed MANF distribution
Density
0.0
0.1
0.2
0.3
0.4
−4
−2
0
2
4

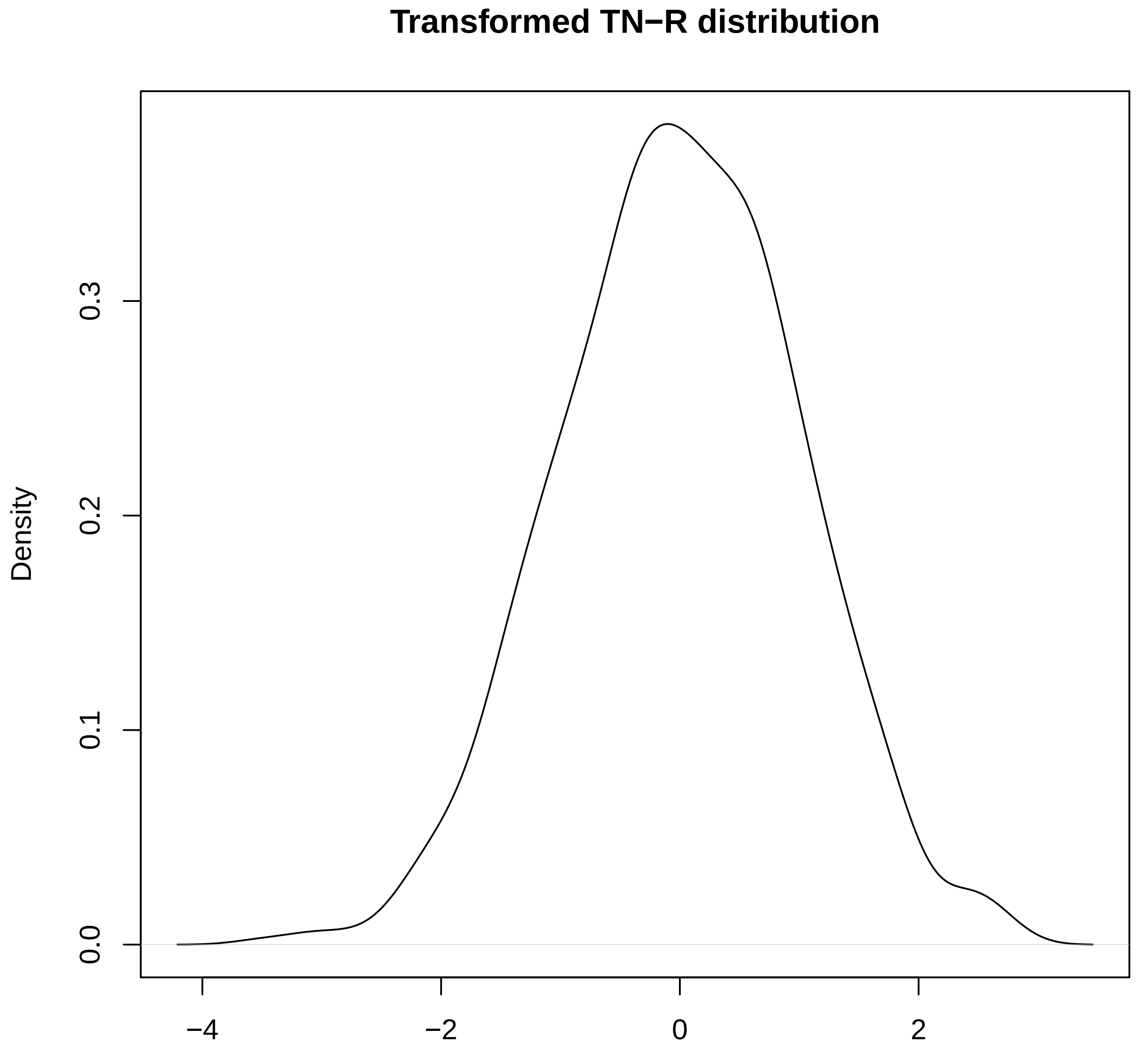
Transformed TN−R distribution
Density
0.0
0.1
0.2
0.3
−4
−2
0
2

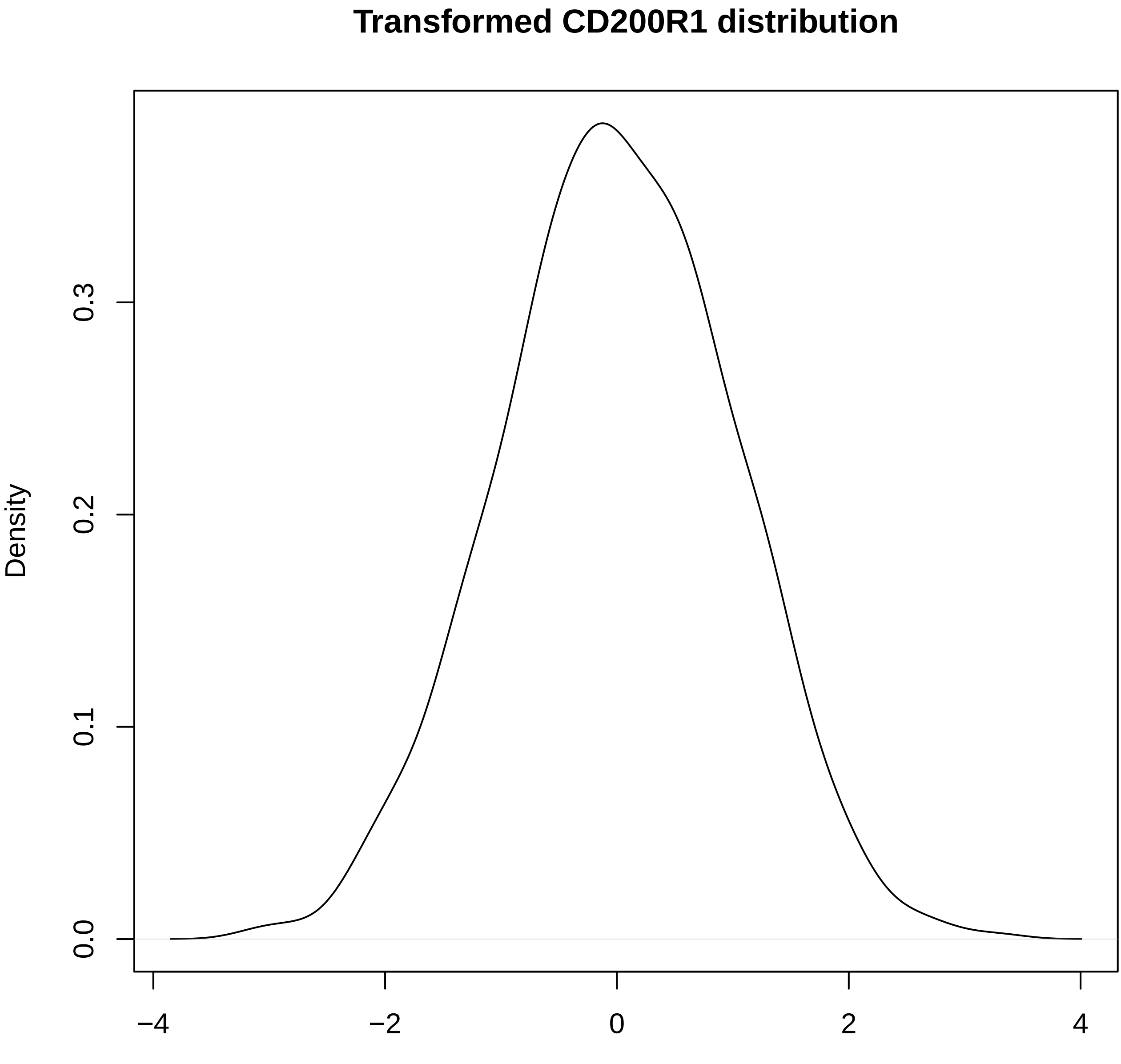

Transformed CD200R1 distribution
Density
0.0
0.1
0.2
0.3
−4
−2
0
2
4

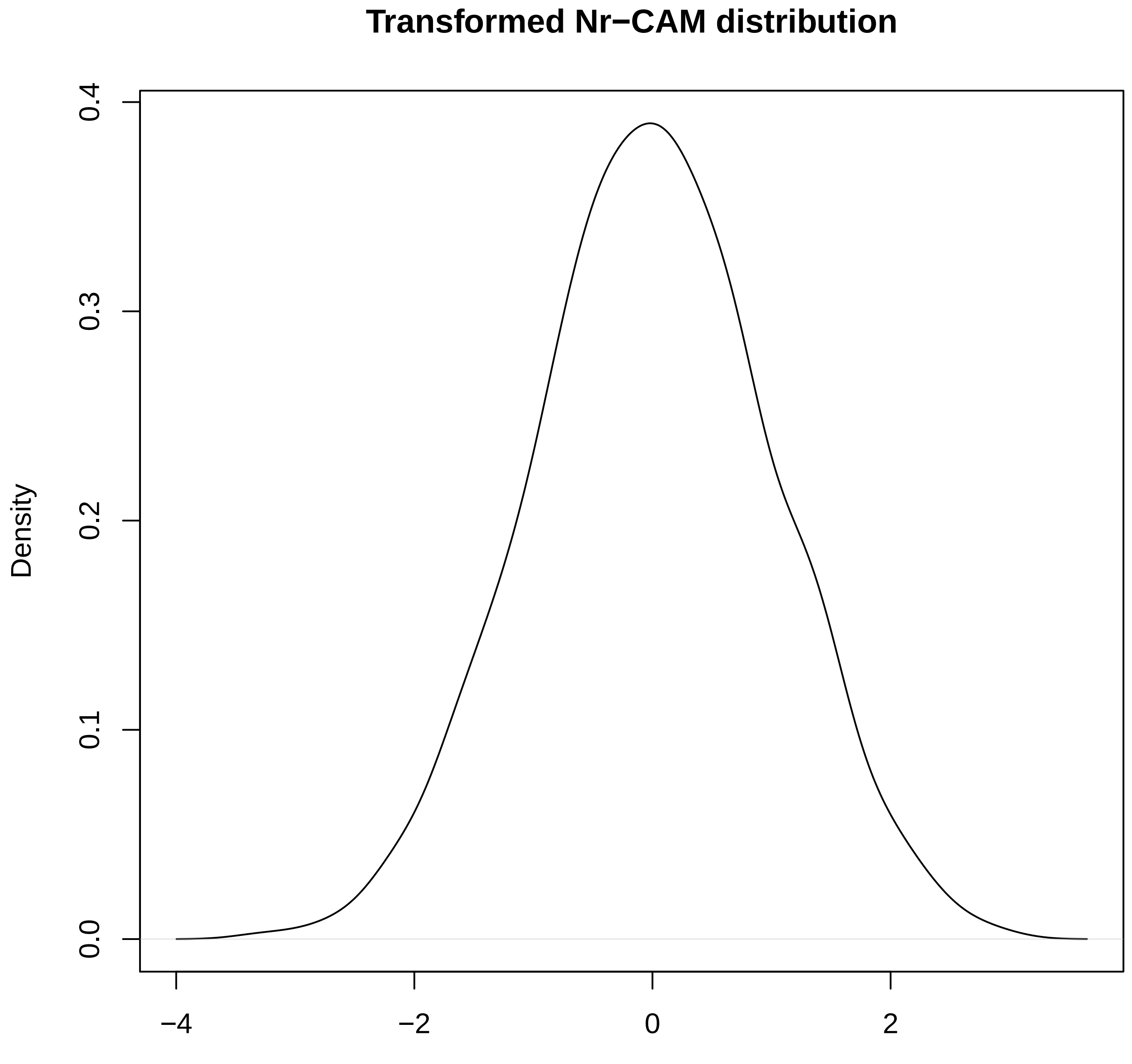

Transformed Nr−CAM distribution
Density
0.0
0.1
0.2
0.3
0.4
−4
−2
0
2

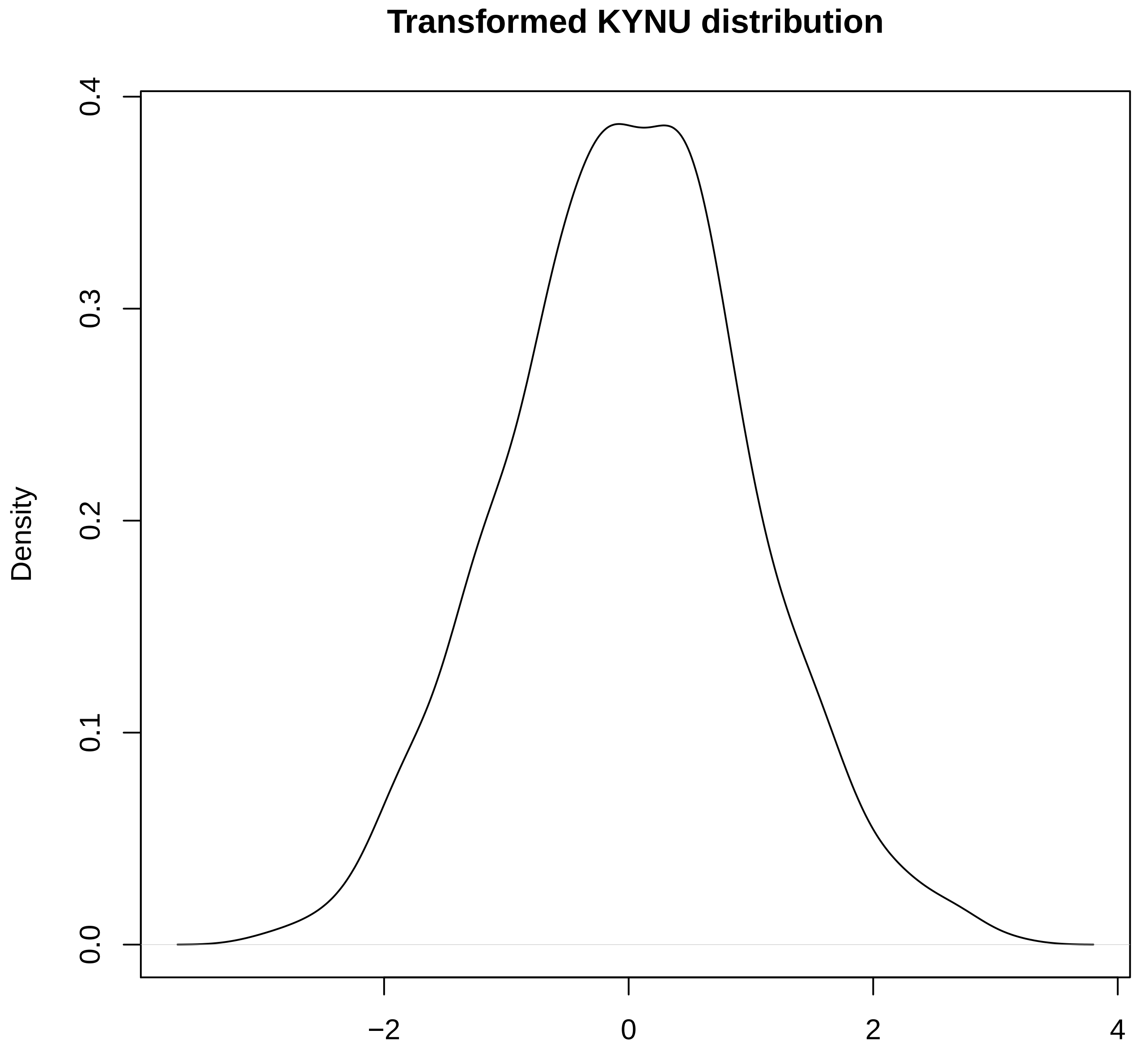
Transformed KYNU distribution
Density
0.0
0.1
0.2
0.3
0.4
−2
0
2
4
