# Supplementary material for: Genome and epigenome wide studies of neurological protein biomarkers in the Lothian Birth Cohort 1936
Source: Nat Commun. 2019 Jul 18;10:3160. doi: 10.1038/s41467-019-11177-x (PMC6639385; doi:10.1038/s41467-019-11177-x)
Supplement: Supplementary file 14 — Reporting Summary [file 41467_2019_11177_MOESM14_ESM.pdf]

nature research

Corresponding author(s): Riccardo Marioni (riccardo.marioni@ed.ac.uk)

Last updated by author(s): Jun 18, 2019

Reporting Summary

Nature Research wishes to improve the reproducibility of the work that we publish. This form provides structure for consistency and transparency in reporting. For further information on Nature Research policies, see Authors & Referees and the Editorial Policy Checklist.

Statistics

For all statistical analyses, confirm that the following items are present in the figure legend, table legend, main text, or Methods section.

n/a	Confirmed	
☐	☒	The exact sample size (n) for each experimental group/condition, given as a discrete number and unit of measurement
☐	☒	A statement on whether measurements were taken from distinct samples or whether the same sample was measured repeatedly
☐	☒	The statistical test(s) used AND whether they are one- or two-sided<br>*Only common tests should be described solely by name; describe more complex techniques in the Methods section.*
☐	☒	A description of all covariates tested
☐	☒	A description of any assumptions or corrections, such as tests of normality and adjustment for multiple comparisons
☐	☒	A full description of the statistical parameters including central tendency (e.g. means) or other basic estimates (e.g. regression coefficient) AND variation (e.g. standard deviation) or associated estimates of uncertainty (e.g. confidence intervals)
☐	☒	For null hypothesis testing, the test statistic (e.g. F, t, r) with confidence intervals, effect sizes, degrees of freedom and P value noted<br>*Give P values as exact values whenever suitable.*
☐	☒	For Bayesian analysis, information on the choice of priors and Markov chain Monte Carlo settings
☒	☐	For hierarchical and complex designs, identification of the appropriate level for tests and full reporting of outcomes
☐	☒	Estimates of effect sizes (e.g. Cohen's d, Pearson's r), indicating how they were calculated

Our web collection on statistics for biologists contains articles on many of the points above.

Software and code

Policy information about availability of computer code

Data collection	N/A
Data analysis	Code will be available from the authors on request as well as deposited on the University of Edinburgh datastore.

For manuscripts utilizing custom algorithms or software that are central to the research but not yet described in published literature, software must be made available to editors/reviewers. We strongly encourage code deposition in a community repository (e.g. GitHub). See the Nature Research guidelines for submitting code & software for further information.

Data

Policy information about availability of data

All manuscripts must include a data availability statement. This statement should provide the following information, where applicable:

- Accession codes, unique identifiers, or web links for publicly available datasets
- A list of figures that have associated raw data
- A description of any restrictions on data availability

LBC36 data are available on request from the Lothian Birth Cohort Study, Centre for Cognitive Ageing and Cognitive Epidemiology, University of Edinburgh. LBC36 data are not publicly available due to them containing information that could compromise participant consent and confidentiality. Full and openly accessible summary statistics from the association studies on Olink® neurology protein levels will be made available on the University of Edinburgh datastore.

Field-specific reporting

Please select the one below that is the best fit for your research. If you are not sure, read the appropriate sections before making your selection.

☒ Life sciences ☐ Behavioural & social sciences ☐ Ecological, evolutionary & environmental sciences

For a reference copy of the document with all sections, see nature.com/documents/nr-reporting-summary-flat.pdf

Life sciences study design

All studies must disclose on these points even when the disclosure is negative.

Sample size	The Lothian Birth Cohort 1936 (LBC1936) comprises 1,091 individuals with detailed genetic, epigenetic, proteomic, lifestyle, health and cognitive data. In this study, proteomic and genotypic data were available for 750 individuals. Furthermore, complete methylation and proteomic data were available for 692 individuals. No sample size calculations were performed.
Data exclusions	Genotyping: Individuals were excluded on the basis of sex discrepancies, relatedness, SNP call rate of less than 0.95, and evidence of non-Caucasian descent.<br>Methylation: Furthermore, samples were removed if they had a poor match between genotype and SNP control probes, or incorrect DNA methylation-predicted sex.<br>Outliers for white blood cell counts (n = 22) were also excluded prior to analyses.
Replication	Replication of the protein-genotype associations was carried out via lookup of genotype-protein summary statistics from existing protein-genotype studies (references outlined in text). Associations were considered to be replicated at P < 1.25 x 10-7 (denoting the least conservative threshold across all studies).
Randomization	N/A
Blinding	N/A

Reporting for specific materials, systems and methods

We require information from authors about some types of materials, experimental systems and methods used in many studies. Here, indicate whether each material, system or method listed is relevant to your study. If you are not sure if a list item applies to your research, read the appropriate section before selecting a response.

Materials & experimental systems

n/a	Involved in the study	
☒	☐	Antibodies
☒	☐	Eukaryotic cell lines
☒	☐	Palaeontology
☒	☐	Animals and other organisms
☐	☒	Human research participants
☒	☐	Clinical data

Methods

n/a	Involved in the study	
☒	☐	ChIP-seq
☒	☐	Flow cytometry
☒	☐	MRI-based neuroimaging

Human research participants

Policy information about studies involving human research participants

Population characteristics	The Lothian Birth Cohort 1936 (LBC1936) were approximately 70 years of age at Wave 1 of the study (mean age: 69.6 ± 0.8 years), and 50.2% male.
Recruitment	The LBC1936 comprises Scottish individuals born in 1936, most of whom took part in the Scottish Mental Survey 1947 at age 11. Participants who were living within Edinburgh and the Lothians were re-contacted approximately 60 years later, 1,091 consented and joined the LBC1936. Upon recruitment, participants were approximately 70 years of age (mean age: 69.6 ± 0.8 years). LBC36 participants have subsequently attended four waves of clinical examination every four years.
Ethics oversight	Ethical permission for the LBC1936 was obtained from the Multi-Centre Research Ethics Committee for Scotland (MREC/01/0/56) and the Lothian Research Ethics Committee (LREC/2003/2/29). Written informed consent was obtained from all participants.

Note that full information on the approval of the study protocol must also be provided in the manuscript.
