# Supplementary material for: Genome and epigenome wide studies of neurological protein biomarkers in the Lothian Birth Cohort 1936
Source: Nat Commun. 2019 Jul 18;10:3160. doi: 10.1038/s41467-019-11177-x (PMC6639385; doi:10.1038/s41467-019-11177-x)
Supplement: Supplementary file 15 — Description of Additional Supplementary Files [file 41467_2019_11177_MOESM15_ESM.doc]

**Title: Supplementary Data 1:**
**Description:** All pQTLs associated with neurological biomarker levels.

**Title:** **Supplementary Data 2:
Description:** Independent pQTL variants identified by conditional and joint analysis.

**Title: Supplementary Data 3:**
**Description:** Independent pQTL variants identified using FUMA with predicted function and mapped to nearest gene.

**Title: Supplementary Data 4:
Description:** Tissue-specific patterns of expression for genes associated with Olink® neurology proteins exhibiting genome-wide significant CpG sites.

**Title: Supplementary Data 5:
Description:** Gene Ontology terms in which genes mapped to CpG sites associated with SIGLEC1 levels are enriched.

**Title: Supplementary Data 6:
Description:** Gene Ontology terms in which genes mapped to CpG sites associated with G-CSF levels are enriched.

**Title: Supplementary Data 7:
Description:** Gene Ontology terms in which genes mapped to CpG sites associated with NEP levels are enriched.

**Title: Supplementary Data 8:
Description:** Replication of conditionally significant pQTLs (GCTA-COJO) from previous studies for which testing was possible.

**Title: Supplementary Data 9:**
**Description:** Replication of pQTLs (FUMA) from previous studies for which testing was possible.

**Title: Supplementary Data 10:**
**Description:** Pre-adjusted protein level distributions.

**Title: Supplementary Data 11:
Description:** Transformed protein level distributions.

**Title: Supplementary Data 12:
Description:** The association of pre-adjusted protein levels with biological and technical covariates.
